# Gene gain facilitated endosymbiotic evolution of Chlamydiae

**DOI:** 10.1038/s41564-022-01284-9

**Published:** 2023-01-05

**Authors:** Jennah E. Dharamshi, Stephan Köstlbacher, Max E. Schön, Astrid Collingro, Thijs J. G. Ettema, Matthias Horn

**Affiliations:** 1 Department of Cell and Molecular Biology, Science for Life Laboratory, Uppsala University, Uppsala, Sweden; 2 University of Vienna, Centre for Microbiology and Environmental Systems Science, Vienna, Austria; 3 University of Vienna, Doctoral School in Microbiology and Environmental Science, Vienna, Austria; 4 Laboratory of Microbiology, Wageningen University and Research, Wageningen, The Netherlands

**Keywords:** Bacterial evolution, Symbiosis

## Abstract

Chlamydiae is a bacterial phylum composed of obligate animal and protist endosymbionts. However, other members of the Planctomycetes–Verrucomicrobia–Chlamydiae superphylum are primarily free living. How Chlamydiae transitioned to an endosymbiotic lifestyle is still largely unresolved. Here we reconstructed Planctomycetes–Verrucomicrobia–Chlamydiae species relationships and modelled superphylum genome evolution. Gene content reconstruction from 11,996 gene families suggests a motile and facultatively anaerobic last common Chlamydiae ancestor that had already gained characteristic endosymbiont genes. Counter to expectations for genome streamlining in strict endosymbionts, we detected substantial gene gain within Chlamydiae. We found that divergence in energy metabolism and aerobiosis observed in extant lineages emerged later during chlamydial evolution. In particular, metabolic and aerobic genes characteristic of the more metabolically versatile protist-infecting chlamydiae were gained, such as respiratory chain complexes. Our results show that metabolic complexity can increase during endosymbiont evolution, adding an additional perspective for understanding symbiont evolutionary trajectories across the tree of life.

## Abstract (web summary)

Extensive gene gain via horizontal gene transfer facilitated the evolution of obligate intracellular Chlamydiae.

## Main

Symbioses are sustained interactions between different organisms that span the mutualism–parasitism spectrum^1,2^. Symbiotic associations between bacterial symbionts and both microbial (that is, protists) and multicellular eukaryotic hosts are ubiquitous^3,4^ and play essential roles, from ecosystem functioning to the evolution of biological complexity^5–8^. Driven by small population sizes, lack of recombination and host dependence, obligate intracellular symbionts—that is, endosymbionts—tend to undergo genome reduction and metabolic streamlining^9–13^. Studying the origins of ancient endosymbiotic groups is necessary to unravel symbiont evolutionary trajectories and underlying evolutionary processes. Host association has evolved multiple times in the Planctomycetes–Verrucomicrobia–Chlamydiae (PVC) superphylum, a group of bacteria consisting of the aforementioned phyla alongside Lentisphaerae, Kirimatiellaeota and other potential members^14,15^. PVC bacteria represent an ideal case for investigation of symbiont evolution because they are ubiquitous, have large variations in lifestyle and metabolism and include members of ecological, medical and industrial importance^14,16,17^. While most PVC bacteria are free living, all described Chlamydiae are obligate endosymbionts of eukaryotes^16^.

Chlamydiae are well known for the medically important human pathogen *Chlamydia trachomatis* and other Chlamydiaceae family members, which are animal pathogens with a high health burden and zoonotic potential^18–20^. Chlamydiae are also ubiquitous in environmental samples^21,22^ as endosymbionts of a wide range of both protist and animal hosts^15,23,24^. Nevertheless, apart from roles as pathogens (for example, Chlamydiaceae), chlamydial host effects are understudied despite host interactions spanning the mutualism–parasitism spectrum. Protist-infecting chlamydiae (for example, Parachlamydiaceae) can act as mutualists that protect against host co-infection with *Legionella* and giant viruses^25,26^. Parachlamydiaceae have larger genomes and greater metabolic capacity than the animal-pathogenic Chlamydiaceae^27,28^. Despite contrasting genomic features, all described chlamydiae share a biphasic lifestyle with an intracellular replicative phase as reticulate bodies (RBs) and a nondividing extracellular phase as elementary bodies (EBs)^19^. Chlamydiae diverged from other PVC bacteria 1–2 billion years ago (Ga)^29,30^, and their endosymbiotic lifestyle is proposed to have evolved early^29,31–33^. It was also thought that the chlamydial ancestor resembled extant protist-infecting lineages and had greater coding potential and metabolic versatility while other chlamydial groups underwent genome reduction. However, because initial studies included only the minimal chlamydial genomic diversity available from cultured representatives, little is known about the evolution of endosymbiosis in Chlamydiae. Through culture-independent genomics, numerous chlamydial lineages with unknown hosts have now been retrieved from various environments^34–39^. These groups fundamentally changed our understanding of chlamydial physiology by revealing genetic potential for motility^37,38^ and anaerobic metabolism^39,40^. However, all isolated chlamydiae are still obligate endosymbionts.

We leveraged the culture-independent expansion in PVC bacteria genomic diversity to investigate endosymbiont evolution exemplified by Chlamydiae. We performed in-depth phylogenomic analyses to reconstruct PVC bacteria evolutionary relationships and gene-tree-aware ancestral state reconstruction. We reconstructed key endosymbiont genomic features in the Chlamydiae ancestor, suggesting an ancient capability to infect eukaryotic hosts. The Chlamydiae ancestor was inferred to have been a motile facultative anaerobe, indicating a lifestyle involving transitions between oxic and anoxic environments. Major shifts in chlamydial energy metabolism and oxygen tolerance were later mainly driven by gene gain. Counter to our expectations for genome streamlining during endosymbiont evolution, gene gain led to expanded metabolic potential in protist-infecting chlamydial groups.

## Results

### Establishment of a resolved Chlamydiae species phylogeny

**Fig. 1:**
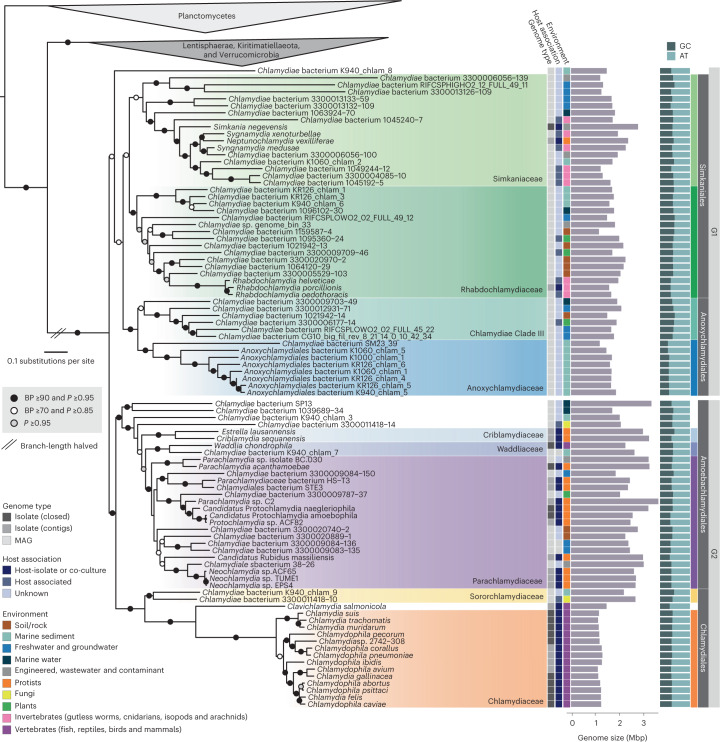
Robust species phylogeny of PVC bacteria. Concatenated (74 marker genes) Bayesian phylogeny of 180 PVC bacteria with compositionally heterogeneous sites removed (8,151 amino acid sites). Circles indicate bipartition support from posterior probability (*P*) (CAT + GTR + Γ4 model), and nonparametric bootstraps (BP) (LG + C60 + F + Γ4-derived PMSF approximation). The tree is rooted by Planctomycetes. Reduced branch lengths are indicated by parallel lines, and substitutions per site by the scale bar. Genome type, evidence for host association and the environment from which the genome was obtained are indicated by coloured squares according to the legend. Genome size (Mbp in purple) and GC content (%GC in dark blue and %AT in light blue) are indicated by bars. Higher-level taxonomic classifications are indicated and chlamydial families outlined by coloured boxes. See also Supplementary Data 2 for genome characteristics and Supplementary Data 6 for the uncollapsed species phylogeny.

**Extended Data Fig. 1:**
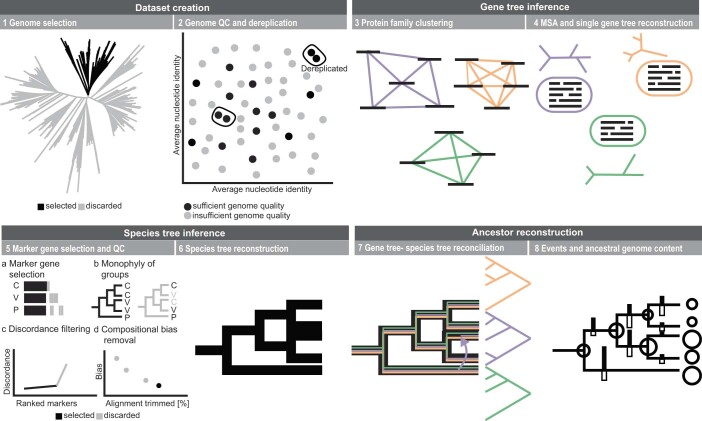
Workflow for ancestral state reconstruction of Chlamydiae. Dataset creation: PVC bacteria representatives from public repositories were selected with completeness ≥90% and redundancy ≤2%. Species and genus-level representatives were selected for Chlamydiae and non-Chlamydiae PVC members, respectively. Gene tree inference: Protein sequences from the selected dataset were clustered into NOG gene families at the last universal common ancestor level. Unmapped protein sequences were de novo clustered. Protein sequences from each resulting gene family were aligned into a multiple sequence alignment (MSA) and ML single-gene trees inferred. Species tree inference: Gene families found in a single-copy in at least 95% of dataset taxa were selected as potential marker genes. ML single-gene trees were inferred and manually curated, with marker genes that well-resolved PVC phyla retained; further marker genes were removed through discordance filtering, while distant homologs, paralogs, and redundant sequences were removed for each retained marker gene. Individually aligned protein sequences from each marker gene were then concatenated into a supermatrix alignment that was used for both ML and Bayesian phylogenetic inference, with compositionally heterogeneous sites sequentially removed to reduce bias. Ancestor reconstruction: ancestral states were reconstructed using gene-tree species-tree reconciliation. See Methods for details.

To accurately resolve PVC bacteria species relationships we selected high-quality genomes of species (Chlamydiae) and genus (other PVC bacteria) representatives (Extended Data Fig. 1, Supplementary Fig. 1 and Supplementary Data 1 and 2). Maximum-likelihood (ML) and Bayesian species trees were inferred using 74 concatenated single-copy marker genes, using methods to account for compositional bias and long branch attraction (Fig. 1, Supplementary Figs. 2–5, Supplementary Data 3–5 and Supplementary Discussion 1). Chlamydiae monophyly was fully supported, and chlamydial families were consistently resolved and compatible with a 16S ribosomal RNA gene phylogeny (Fig. 1 and Supplementary Figs. 3–6). Two long-branching lineages consisting of four chlamydial genomes were removed due to unstable positions (Supplementary Figs. 3 and 4 and Supplementary Discussion 1). In the final dataset, deep evolutionary relationships were consistently resolved in both Bayesian and ML analyses when compositional bias was taken into account (Fig. 1 and Supplementary Fig. 5). These final 180 PVC representatives included 91 chlamydial species sequenced from nine environments with genome size and GC content ranging from 1.05 to 3.42 Mbp and 26.2 to 49.1%, respectively. With a robust species tree and comprehensive sampling, we here propose revision of chlamydial taxonomy (Fig. 1 and Supplementary Discussion 2). Consistent with recent work^37,39–41^, an early divergence of Chlamydiae into two major groups, Group 1 (G1) and Group 2 (G2), is well supported. We can further subdivide G1 into two putative orders: Simkaniales (families Simkaniaceae and Rhabdochlamydiaceae) and Anoxychlamydiales (Anoxychlamydiaceae, formerly Anoxychlamydiales^37^, and Chlamydiae Clade III). G1 members are primarily represented by metagenome-assembled genomes (MAGs) sequenced from diverse environments and with unknown hosts, although several distinct groups were obtained from invertebrate animal metagenomes. The G2 subdivision includes the classical chlamydial animal pathogens, Chlamydiaceae and *Clavichlamydia*, alongside Sororchlamydiaceae^42^ in the previously established Chlamydiales order^43^. Other G2 families (Parachlamydiaceae, Criblamydiaceae, Waddliaceae and orphan lineages) comprise the here-defined order Amoebachlamydiales, which primarily infect protists.

### The chlamydial endosymbiotic lifestyle is ancestral

To reconstruct PVC genome evolution we used the gene-tree-aware amalgamated likelihood estimation (ALE) approach^44^ (Supplementary Figs. 7–10 and Supplementary Data 7–9). Protein-coding genes from PVC genomes were clustered into homologous gene families and phylogenies inferred for the 11,996 clusters with more than three sequences. Gene tree samples were then reconciled with the species tree to infer evolutionary events (speciations, originations, duplications, transfers and losses) and proteome size (that is, the sum of inferred gene copies corrected by gene extinction probability) across ancestral nodes (Extended Data Fig. 1). Genes inferred as present with high confidence were used to reconstruct ancestral gene content, and event frequencies to assess overall patterns in gene content evolution. Of note, we can reconstruct only those genes present in extant genomes and in the dataset. Thus, ancestral reconstructions are incomplete because we miss gene families that have gone extinct or were not sampled. ALE compensates for this by taking into account estimated gene extinction rates and genome completeness for inferring gene copy numbers. Originations can derive from either de novo gene birth or horizontal gene transfer (HGT) from outside the PVC genome dataset. For Chlamydiae originations, to differentiate between these we searched for homologues in protein databases. Where identified, phylogenetic trees were inferred to discern with which taxonomic groups chlamydial genes affiliated, indicating putative donor lineages of the horizontally transferred gene families.

**Fig. 2:**
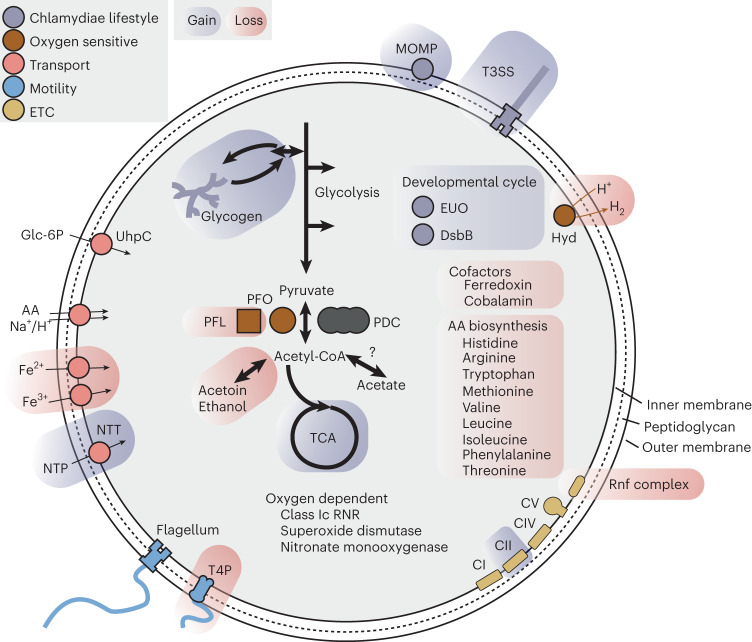
Schematic of gene content reconstructed in LCCA. Gains and losses in LCCA relative to LVCCA are indicated by blue and red backgrounds, respectively. The reconstructed presence of a peptidoglycan-based cell wall is indicated by the dashed line between inner and outer membranes. ADI, arginine deiminase pathway; CI–V, electron transport chain complexes I–V; Hyd, [FeFe]-hydrogenase; PDC, pyruvate dehydrogenase; T4P, type IV pilus. See also Extended Data Fig. 2 for a more detailed summary and Supplementary Data 9 for gene content reconstructed in LCCA and LVCCA.

**Fig. 3:**
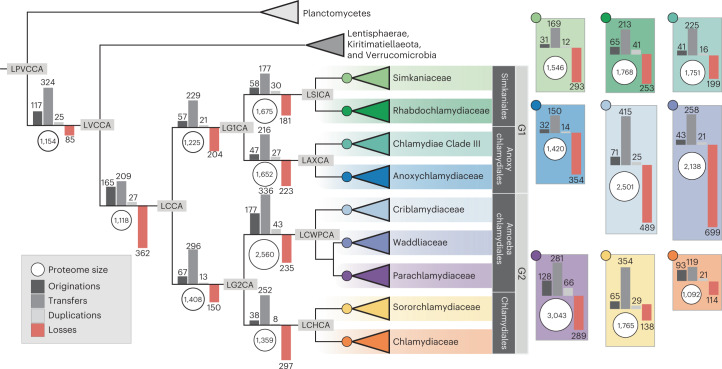
Ancestral reconstruction of Chlamydiae gene content evolution. Schematic Chlamydiae tree indicating ancestral proteome size by white circles to the left of each node. Branches are annotated with bars representing the inferred sum of origination, transfer and duplication events leading to each node (see legend). The sum of loss events is indicated negatively by a red bar. Terminal nodes represent chlamydial family ancestors, with corresponding events shown to the right. Orphan lineages are excluded from the schematic. Abbreviations of ancestors not defined in the text: all PVC bacteria (LPVCCA), Anoxychlamydiales (LAXCA) and Chlamydiales (LCHCA). See also Supplementary Data 7 for event sums across all nodes and event numbers with different reconciliation frequency cutoffs. See Supplementary Fig. 11 for events inferred at reconciliation frequencies ≥0.3.

**Extended Data Fig. 2:**
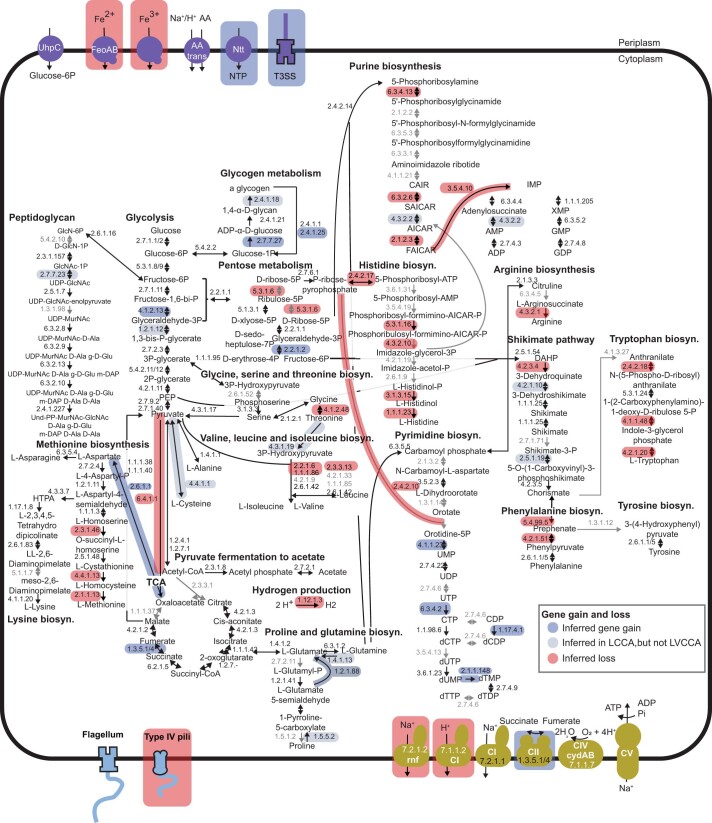
Chlamydiae last common ancestor reconstructed central metabolism. Genes were reconstructed as present if they had an inferred relative frequency ≥0.3 in ALE. Enzyme complexes were inferred as present if at least half of the necessary genes were present. Enzymes are annotated with enzyme commission numbers, arrows indicate the directionality of reactions and are coloured in black or grey if inferred as present or absent, respectively. Highlights show changes in inferred gene content between LVCCA and LCCA. Purple, light purple and red highlights indicate inferred gains, presence (while not inferred in LVCCA), and losses LVCCA, respectively. See Data S9 for gene families inferred in LCCA and LVCCA and corresponding annotations.

**Extended Data Fig. 3:**
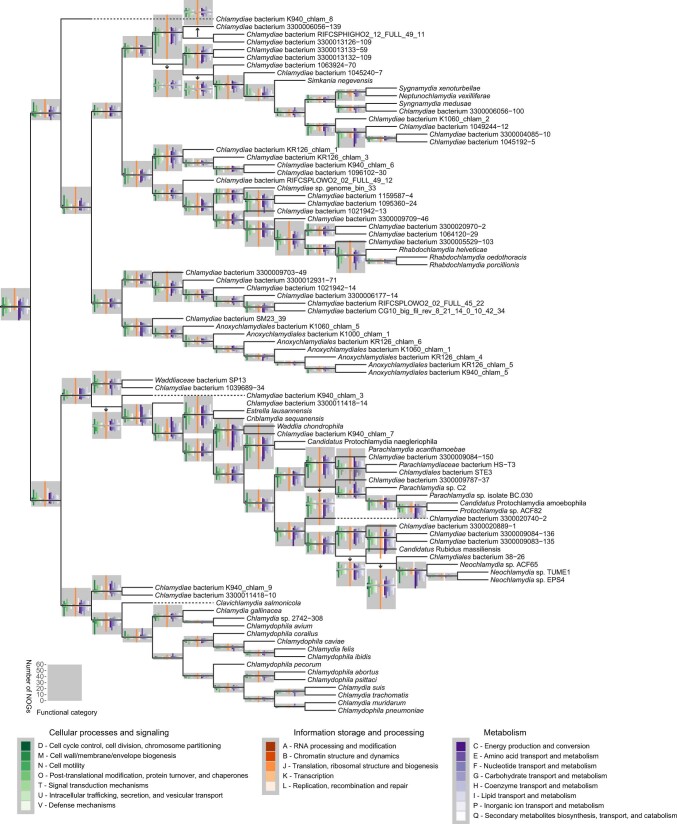
Gain and loss events per functional category across Chlamydiae. Cladogram of Chlamydiae phylogenetic relationships showing gain (originations and transfers) and loss events of eggNOG NOG gene families with reconciliation frequencies ≥0.3 (see Methods) across COG categories. The barplots mapped onto each branch indicate the events that have occurred leading to the ancestral node to their right. The number of NOGs gained are indicated in the positive direction, and those lost in the negative direction, with the number corresponding to the bar height (see grey box scale). Bars are sorted and coloured according to the COG category. NOGs assigned to poorly characterized COG categories (R: general function prediction only, S: function unknown, X: Mobilome), multiple categories, and de novo gene families were excluded. See Data S7 for gene gains and losses inferred per functional category.

**Extended Data Fig. 4:**
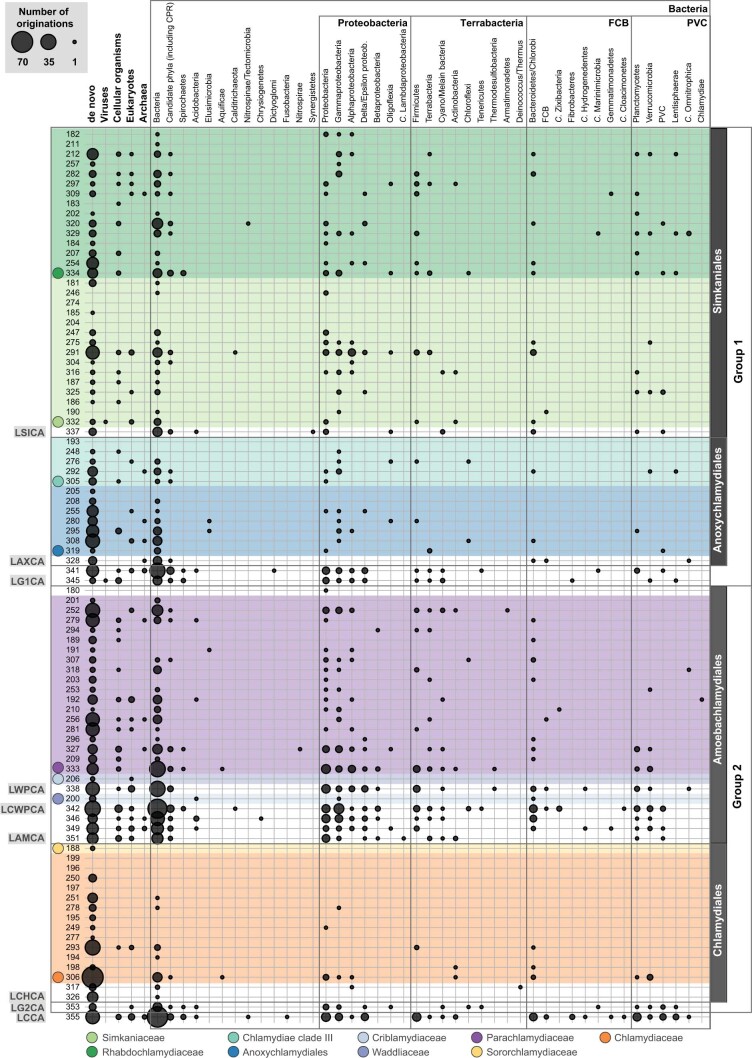
Taxonomic affiliation of inferred gene originations across Chlamydiae. Number of gene originations affiliated with different taxonomic groups across Chlamydiae ancestral nodes. For all chlamydial originations (that is, transfers from outside the PVC bacteria dataset used), gene trees were inferred with sequences from public databases and the affiliated taxonomic group identified (consensus of 75% of taxa in the sister clade), which represents the putative donor group. Chlamydiae taxonomy and ancestors are indicated to the right and left, respectively; see Data S7 for ancestor abbreviations and Figure S10 for node numbers mapped to chlamydial phylogeny. See Data S11 for chlamydial originations and affiliated taxonomic groups.

**Extended Data Fig. 5:**
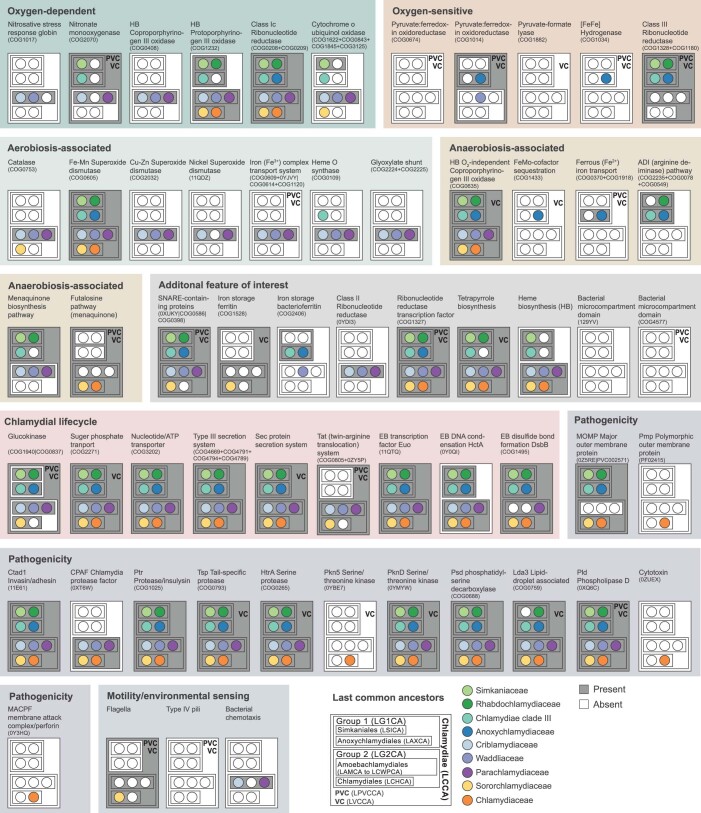
Reconstructed presence of genes and pathways of interest across Chlamydiae ancestors. Gene family IDs (COG, NOG, or de novo identifier) are indicated for genes and complexes, and for pathways can be found in Data S10. See Data S10 for corresponding gene family annotations and inferred presence across all Chlamydiae ancestors.

The last common Chlamydiae ancestor (LCCA) was reconstructed with ~1,118 protein-coding genes (Figs. 2 and 3, Extended Data Fig. 2 and Supplementary Data 9). Of these, 401 were inferred as gene gains, many associated with metabolism (*n* = 99) and obtained through HGT (Extended Data Figs. 3 and 4). Hallmark endosymbiont genomic features for host interaction, energy parasitism and the chlamydial biphasic lifecycle were gained before LCCA. These genes included a type III secretion system (T3SS) (for example, genes *sctJ*, *sctT*, *sctS*, *sctV* and *sctW*), the adhesin Ctad1 and major outer membrane protein (MOMP), two nucleotide transporters (NTTs), DsbB, glycogen biosynthesis and degradation (for example, *glgC*, *glgP*, *malQ*) and the transcriptional regulator early upstream ORF (EUO) (Fig. 2, Extended Data Figs. 2 and 5 and Supplementary Data 9 and 10). The T3SS facilitates host cell entry through effector secretion^45^, and Ctad1 and MOMP are pathogenicity factors in Chlamydiaceae involved in host invasion^19^. In the RB stage, NTTs facilitate energy parasitism and metabolite scavenging by importing ATP, nucleotides and NAD^+^ from the host cytosol^28^. In the EB stage, DNA is condensed by histone-like proteins (for example, HctA), and the cell envelope rigidified to protect against osmotic and physical stress through disulfide crosslinking of outer membrane proteins by DsbB^46^. HctA was not reconstructed in LCCA, but in all early chlamydial ancestors (Extended Data Fig. 5 and Supplementary Data 10). Glycogen is used as a carbon source by EBs and enhances extracellular survival^47^. EUO is a master regulator that represses T3SS, DNA condensation and cell surface modification genes before RB-to-EB conversion^48,49^, and is a putative chlamydial gene invention.

Many gene losses (31%, *n* = 362) were inferred between LCCA and the last common ancestor of Chlamydiae, Verrucomicrobia, Lentisphaerae and Kiritimatiellaeota (LVCCA) (Fig. 3), and were predominantly associated with metabolism (49%, Extended Data Fig. 3). Relative to LVCCA, de novo amino acid and nucleotide biosynthesis capabilities were strongly reduced in LCCA through loss of genes for histidine, arginine, tryptophan, methionine, valine, leucine, isoleucine, phenylalanine, threonine and purine (for example, *purC*, *purD* and *purH*) biosynthesis (Fig. 2, Extended Data Fig. 2 and Supplementary Data 9). However, a suite of amino acid and oligopeptide transporters was already inferred as being present in LVCCA and maintained in LCCA, with both able to acquire amino acids from external sources. NAD and NADP biosynthesis genes were inferred in LCCA. Pathways for biosynthesis of other cofactors, such as ferredoxin and cobalamin, were inferred as lost and LCCA probably depended on their uptake. Several Chlamydiaceae virulence factors associated with host metabolite degradation (for example, proteases and lipases) were inferred as present already in LVCCA and retained throughout chlamydial evolution (Extended Data Fig. 5 and Supplementary Data 10). LVCCA also already had the potential to import glucose-6-phosphate with the transporter UhpC, which is used to scavenge host glucose by chlamydiae and other endosymbionts^50^ (Fig. 2 and Supplementary Data 9). While peptidoglycan biosynthesis genes are absent in some Planctomycetes^51^, we reconstructed most key genes (for example, *murACDEFGJ*, *mreB* and *mraY*) in both LVCCA and LCCA (Fig. 2, Extended Data Fig. 2 and Supplementary Data 9 and 10).

Earlier hypotheses for Chlamydiae genome evolution were based only on gene presence patterns or included limited genomic diversity^32,33^. Our results provide the previously missing support that LCCA already had the genetic toolkit for an endosymbiotic and biphasic lifestyle. Furthermore, we have shown that key genes were gained before LVCCA, and that LCCA also evolved through a reduction in pathways involved in de novo biosynthesis and hence a dependence on uptake of essential metabolites. Chlamydiae diverged from other PVC bacteria between 1 and 2 Ga, coinciding with estimates for the evolution of eukaryotes (1.2–2.1 Ga)^29,30,32,33^. Our reconstruction demonstrates that LCCA was already an obligate endosymbiont, indicating a billion-year-old history of chlamydiae infecting eukaryotic hosts as they evolved.

### A facultative anaerobic origin of Chlamydiae

**Fig. 4:**
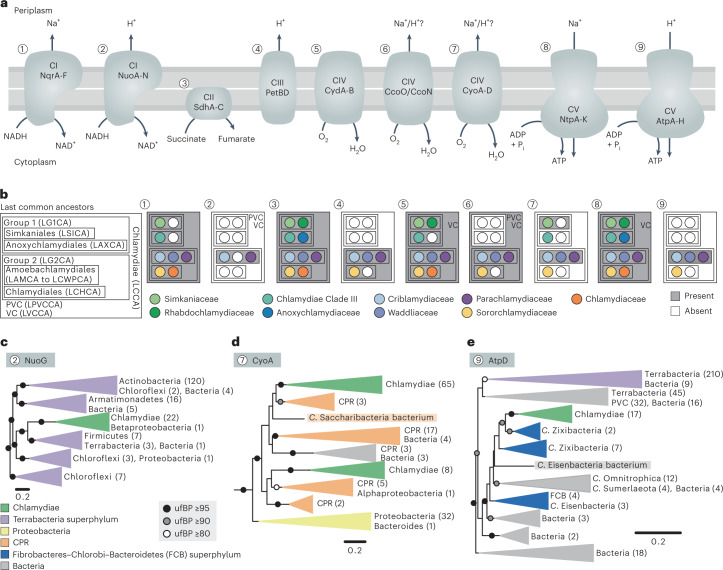
The ancestral chlamydial respiratory chain has undergone expansion in Amoebachlamydiales. **a**, Schematic of respiratory complexes found in Chlamydiae, with substrates and complexes. **b**, Presence and absence of respiratory complexes across chlamydial ancestors. Presence is defined as ≥50% of complex subunits. **c**–**e**, ML phylogenies of the NuoG subunit of proton-transporting NADH dehydrogenase (NuoA-N) (**c**), the CyoA subunit of cytochrome *o* ubiquinol oxidase (CyoA-D) (**d**) and the AtpD subunit of proton-driven ATP synthase (AtpA-H) (**e**), inferring with the LG model of evolution. Circles indicate ufBP bipartition support. Collapsed clades are annotated and coloured if most sequences have the same taxonomy. Scale bars indicate the number of substitutions per amino acid position in the alignment. See also Supplementary Data 6 for trees of all complex subunits and Supplementary Data 9 for inferred presence across all Chlamydiae ancestors.

**Extended Data Fig. 6:**
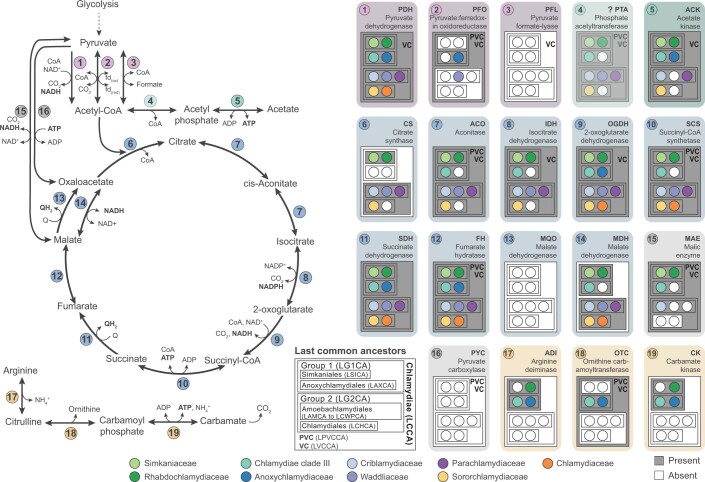
Reconstructed presence of TCA cycle components and fermentative metabolism across Chlamydiae ancestors. Presence and absence of genes encoding enzymes involved in converting pyruvate to acetyl-CoA (pink), pyruvate fermentation to acetate (green), the TCA cycle (blue), pyruvate to TCA intermediate interconversion (grey), and the fermentative arginine deiminase pathway (yellow). A schematic overview of reactions performed by each numbered enzyme is shown to the left, alongside key metabolites (water, bicarbonate and protons are excluded). To the right the presence and absence of each component across key ancestors is indicated according to the legend. See Data S10 for gene annotations and inferred presence across all Chlamydiae ancestors.

Although most Chlamydiae are aerobes, groups with anaerobic metabolism (for example, Anoxychlamydiaceae) were recently identified^39,40^. To unravel the evolutionary history of aerobiosis in Chlamydiae, we investigated metabolic genes reconstructed in LCCA and LVCCA. Core metabolic genes conserved in most extant chlamydiae^28^ were inferred alongside genes indicating a facultatively anaerobic lifestyle (Figs. 2 and 4, Extended Data Figs. 5 and 6 and Supplementary Data 9 and 10). LCCA had the potential to use glycolysis to generate ATP with both glucose (glucokinase) and glucose-6-phosphate (UhpC). The resulting pyruvate could be converted to acetyl-CoA using pyruvate dehydrogenase or the oxygen-sensitive pyruvate:ferredoxin oxidoreductase (PFO) under oxic and anoxic conditions, respectively. Acetyl-CoA could then be directed into the tricarboxylic acid (TCA) cycle or fermented. The TCA cycle was reconstructed as missing citrate synthase and malate dehydrogenase in LCCA, but as complete in many early chlamydial ancestors (Extended Data Fig. 6 and Supplementary Data 10). LCCA could probably perform oxidative phosphorylation, because we inferred a complete respiratory electron transport chain (ETC) including sodium-transporting NADH dehydrogenase (Nqr; Complex I, CI), succinate dehydrogenase (Sdh; CII), terminal oxidases (CIV) cytochrome *bd* ubiquinol oxidase (CydA-B) and cytochrome *c* oxidase *cbb*_*3*_-type (CcoO/N), and sodium-driven ATP synthase (Ntp; CV) (Fig. 4a,b and Suplementary Data 9 and 10). These terminal oxidases have high oxygen affinity and could have been used to respire oxygen under micro-aerophilic conditions, or to provide oxidative stress protection for oxygen-sensitive enzymes like PFO^52,53^. The LCCA ETC was probably used to generate a sodium motive force (SMF), as demonstrated in *C. trachomatis*^54^. The same central metabolism was reconstructed in LVCCA, except for succinate dehydrogenase (Extended Data Fig. 6). LVCCA could also oxidize pyruvate using the oxygen-sensitive pyruvate formate lyase (PFL), and couple pyruvate oxidation to H_2_ production with the oxygen-sensitive [FeFe]-hydrogenase (HydA) (Fig. 2, Extended Data Fig. 2 and Supplementary Data 9). More extensive fermentative capabilities were also reconstructed in LVCCA, which could ferment pyruvate to acetate, acetoin and, potentially, ethanol. An Rnf complex (sodium ion-translocating ferredoxin:NAD^+^ oxidoreductase), which is strictly linked to sodium energetics and strongly associated with anaerobes^55^, was also reconstructed as part of the LVCCA ETC. PFL, HydA, the Rnf complex and some fermentative capabilities were lost between LVCCA and LCCA.

Additional genes encoding proteins with central functions and varying oxygen tolerances were reconstructed in LCCA and LVCCA, and differentially retained across early chlamydial ancestors (Fig. 2, Extended Data Figs. 2 and 5 and Supplementary Data 9 and 10). Complementary copies of ribonucleotide reductase (RNR), the key enzyme for ribonucleotide-to-deoxyribonucleotide interconversion, were reconstructed in LCCA. Under anoxic conditions LCCA and LVCCA could use a class III RNR, which is highly oxygen sensitive^56^, and under oxic conditions LCCA could use a class Ic RNR, which is oxygen dependent^57^. Similarily, haem biosynthesis can occur through both anaerobic and aerobic routes. The anaerobic-route, oxygen-independent coproporphyrinogen III oxidase was reconstructed in LCCA and LVCCA and retained in all chlamydial family ancestors. The aerobic-route, oxygen-dependent protoporphyrinogen III oxidase was reconstructed in most early chlamydial ancestors, but not in LCCA and LVCCA. The oxygen-dependent superoxide dismutase and nitronate monooxygenase, which detoxify oxygen radicals and oxidize alkyl nitronates, were also reconstructed in LCCA and LVCCA. Transport systems for both primary iron species under anoxic (Fe^2+^) and oxic (Fe^3+^) conditions^58,59^ were reconstructed in LVCCA but not in LCCA. LVCCA and LCCA were both probably motile, based on an inferred flagellar apparatus and an additional type IV pilus in LVCCA. Contrary to our expectations and previous hypotheses^32,33^, in LCCA we did not reconstruct the extensive aerobic and energy metabolism found in modern Amoebachlamydiales.

LCCA and LVCCA were reconstructed as facultative anaerobes that encoded oxygen-sensitive and -dependent metabolic genes and pathways associated with both anaerobic and aerobic lifestyles. LVCCA would have lived 2 Ga, soon after the great oxidation event (2.1–2.4 Ga)^30^, when environments with transient oxygen and oxic microclines would have been common. Extant facultative anaerobes in analogous environments (for example, tidal zones, sediments and animal tissues) regulate aerobic and anaerobic gene expression with oxygen exposure^60^. LVCCA may have used motility to transit oxic microclines and adjusted metabolism accordingly, and potentially had a biphasic lifestyle based on oxic–anoxic transitions rather than host invasion as in extant chlamydiae. The eukaryotic intracellular environment can provide a refuge from oxygen, and strict anaerobes can survive and divide within amoeba vacuoles when exposed to high oxygen^61^. A possible scenario that drove the evolution of chlamydial endosymbiosis was a coinciding increase in oxygen and the emergence of a niche suited to a facultative anaerobe within early eukaryotic hosts. Thus, Chlamydiae evolution may have been facilitated by both endosymbiosis-related gene gains and a facultatively anaerobic ancestor.

### Gene gain facilitated oxygen tolerance and respiratory chain expansion in Chlamydiae

Chlamydiae later diversified into two major groups, G1 and G2, divergent in oxygen tolerance. Genes reconstructed in the last common ancestors of G1 (LG1CA) and G2 (LG2CA) and their descendents are indicative of lifestages in anoxic and oxic environments, respectively. The arginine deiminase pathway, known as anaerobic substrate-level phosphorylation, was reconstructed in LG1CA (Extended Data Fig. 6 and Supplementary Data 9 and 10). Similarly, the iron transporter FeoAB for the primary species under anoxic conditions (Fe^2+^) and the hydrogen-producing and oxygen-sensitive HydA were reconstructed in Anoxychlamydiales and Anoxychlamydiaceae ancestors, respectively (Extended Data Fig. 5). Oxygen-utilizing enzymes, such as cytochrome *bd* ubiquinol oxidase, were also lost before the Anoxychlamydiaceae ancestor (Fig. 4a,b and Supplementary Data 9 and 10). In contrast, the oxygen-sensitive PFO was lost before LG2CA (Extended Data Fig. 6). Unexpected in G2 was the gain of an extensive suite of aerobiosis-associated pathways and oxygen-dependent enzymes between the last common ancestors of Amoebachlamydiales (LAMCA) and Criblamydiaceae, Waddliaceae and Parachlamydiaceae (LCWPCA), which indicates adaptation to higher-oxygen environments during early Amoebachlamydiales evolution (Extended Data Fig. 5 and Supplementary Data 10). These included genes encoding coproporphyrinogen III oxidase for aerobic-route haem biosynthesis, iron complex transporters (for example, siderophores) for the primary species under oxic conditions (Fe^3+^), catalase and superoxide dismutases for oxidative stress response, a bacterial globin for nitric oxide detoxification, and the glyoxylate shunt, a TCA cycle bypass almost exclusive to aerobes^62^. Overall, early Amoebachlamydiales ancestors were probably better adapted to oxic environments, suggesting oxygen tolerance as a driving force in chlamydial evolution.

Amoebachlamydiales also expanded energy metabolism by gaining complexes for generation of a proton motive force (PMF) alongside the ancestral SMF. PMF has a larger redox gap than SMF and can result in greater ATP generation^63^. We reconstructed several PMF-associated complexes in LCWPCA, including a proton-transporting NADH dehydrogenase (NuoA-N; CI), cytochrome *bc* complex (PetBD; CIII), cytochrome *o* ubiquinol oxidase (CyoA-D) and proton-driven F-type ATP synthase (AtpA-H; CV) (Fig. 4a,b). In phylogenetic trees of NuoA-H subunits, chlamydial sequences consistently branch with members of the Terrabacteria superphylum (NuoG; Fig. 4c and Supplementary Data 6), supporting gain before LCWPCA. Such physiologically coupled proteins from multisubunit complexes are often gained as a functional unit^64^. PetBD was reconstructed in LG2CA and retained in most descendants, but lost in Chlamydiaceae (Fig. 4a,b). The evolutionary history of CyoA-D in Chlamydiae is unclear. It was reconstructed in Simkaniales (LSICA) and LAMCA order ancestors, but also in Chlamydiae Clade III and Sororchlamydiaceae family ancestors (Fig. 4a,b). CyoA-D gene trees show an affiliation with Candidate Phyla Radiation (CPR) bacteria members and suggest at least one HGT event (CyoA; Fig. 4d and Supplementary Data 6). CyoA-D has lower oxygen affinity and is associated with higher oxygen levels than other terminal oxidases in Chlamydiae^65^. Haem O synthase (CyoE), which generates the CyoA-D haem O cluster, was also reconstructed in LCWPCA and the Chlamydiae Clade III ancestor but not in other early ancestors (Extended Data Fig. 5). In phylogenetic trees of AtpA-H subunits, chlamydial sequences affiliate with *Candidatus* Zixibacteria and the complex was probably gained before LCWPCA in a single HGT event (AtpD; Fig. 4e and Supplementary Data 6). Thus, an extended ETC with mosaic origins was gained before LCWPCA through additive HGT of several complexes from different bacterial groups (Fig. 4). The more extensive metabolic capabilities in protist-infecting Amoebachlamydiales compared with animal pathogen Chlamydiaceae had previously been noted^27,28,66^. However, it had been presumed that differences were a result of gene loss in Chlamydiaceae and other lineages, with LCCA having had the more flexible and branched ETC^33,36^. Our analyses instead indicate that the extended Amoebachlamydiales ETC was gained after divergence from LCCA.

### Gene content expansion as a mode of evolution in endosymbionts

**Fig. 5:**
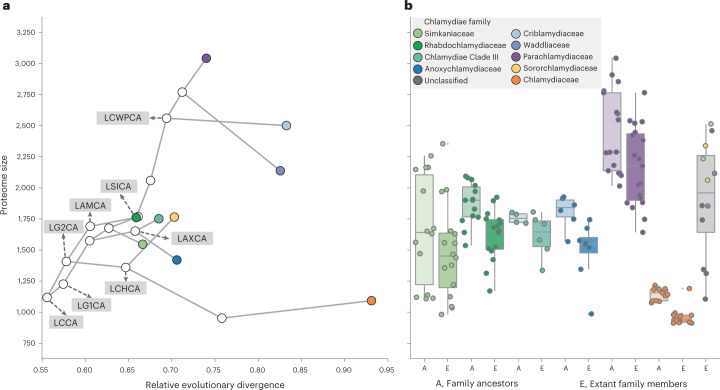
The proteome size of Amoebachlamydiales ancestors increased relative to other chlamydiae. **a**,**b**, Proteome size inferred for chlamydial ancestors, and protein-coding gene copies present in extant chlamydial genomes. **a**, Inferred proteome size of early chlamydial ancestors scaled to relative evolutionary divergence, from LCCA to extant taxa. **b**, Proteome size comparison between inferred ancestors and extant members within each chlamydial family. Chlamydiae without in-family ancestors are grouped together in the grey boxplot (that is, Criblamydiaceae, Waddliaceae, Sororchlamydiaceae and unclassified). Points and boxplots are coloured according to the legend. Centre lines in the box-and-whisker plot represent median values, box limits represent upper and lower quartile values and whiskers represent 1.5 times the interquartile range above the upper quartile and below the lower quartile. Number of ancestors and extant members per family depicted from left to right: *n* = 14, 16, 14, 16, 4, 6, 6, 8, 17, 19, 12, 14 and 12. Abbreviations of key ancestors are labelled as in Fig. 3. See also Supplementary Data 7 for proteome sizes.

Amoebachlamydiales aerobiosis-associated gene expansion was accompanied by additional metabolic gene gains (Extended Data Fig. 3), in line with the extended metabolic capabilities and larger gene repertoires of extant members relative to other chlamydiae^27,28,66^. Our results provide evidence that these key genes were not present in LCCA as expected, but were instead gained later through HGT leading to the characteristic Amoebachlamydiales metabolic complexity. Although we cannot accurately reconstruct the evolution of all genes, such as those gone extinct or rapidly evolving, it is possible to investigate general patterns in relative reconstructed proteome sizes. Our results indicate a shift towards larger proteome sizes between LAMCA and LCWPCA relative to other early chlamydial ancestors (Fig. 5a). The upward trend in reconstructed Amoebachlamydiales proteome sizes was corroborated using a gene presence/absence method (Supplementary Fig. 12). However, proteome sizes of extant taxa and in-family ancestors were consistent only when using the gene-tree-aware method (Fig. 5b and Supplementary Fig. 12b). Proteome size was reconstructed as having expanded from 1,691 in LAMCA to 2,560 in LCWPCA, nearly double that inferred for LG2CA (*n* = 1,408) and indicating genome expansion in Amoebachlamydiales. In contrast, the reconstructed proteome size of the Chlamydiaceae ancestor (*n* = 1,092) suggests genome reduction. In other early chlamydial ancestors, reconstructed proteome sizes are consistent with genome maintenance (Fig. 5).

Despite a conserved endosymbiotic lifestyle, divergent patterns in genome evolution were found across Chlamydiae. Genome reduction in obligate endosymbionts is associated with vertical transmission and a strict host range. The resulting small intracellular population sizes and genetic isolation lead to gene loss through genetic drift, the accumulation of slightly deleterious mutations and a lack of recombination^9–12^. We observed genome reduction leading to the pathogenic Chlamydiaceae, which are typically horizontally transmitted but have strict animal hosts. Genome maintenance in obligate endosymbionts is associated with horizontal transmission and a wider host range. For example, marine bivalve endosymbionts maintain intermediate genome sizes due to horizontal transmission and recombination^67^. Protists have been referred to as ‘melting pots’ of evolution, because their endosymbionts tend to undergo less genome reduction due to HGT with host prey and co-infecting endosymbionts^68–70^. In most chlamydial lineages we observe patterns consistent with genome maintenance, although we lack information about host ranges and transmission. Genome reduction and maintenance are in line with previous work on the effects of transmission mode and host variability on endosymbiont genome evolution^71,72^. While genome expansion has been shown in facultative symbionts with free-living lifestages^73^, it has not previously been shown in obligate endosymbionts. We observed extensive gene gain as having led to the larger proteome sizes and increased metabolic complexity characteristic of extant protist-infecting Amoebachlamydiales. Our findings challenge existing paradigms by providing evidence that obligate endosymbionts can counter genome reduction processes and undergo genome expansion. Given that Chlamydiae is an ancient endosymbiotic phylum, we suggest that endosymbiont genomic and metabolic complexity can increase over long evolutionary time scales.

### Gene exchange is common among chlamydiae

**Extended Data Fig. 7:**
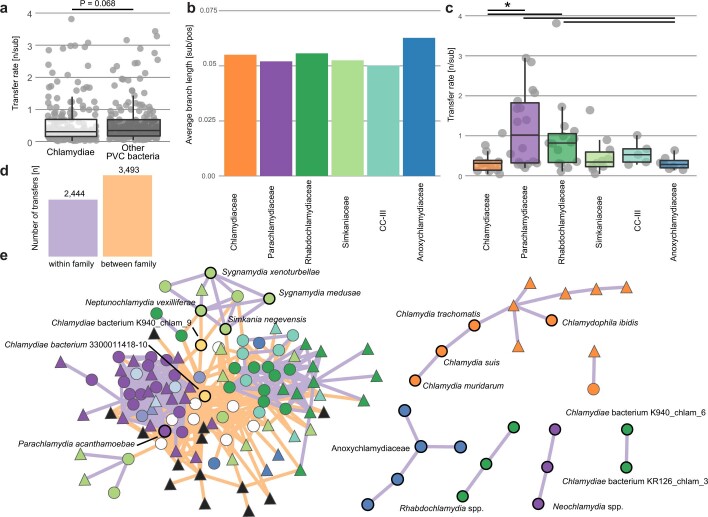
Transfer rates in Chlamydiae and other PVC bacteria, and network of enriched transfer routes in Chlamydiae. **a**. Boxplot depicting median lower transfer rates (one-sided Wilcoxon signed rank test, P = 6.8 × 10^−2^) in Chlamydiae (n = 181, median 0.31, IQR 0.16–0.69) than in other PVC bacteria (n = 172, median 0.34, IQR 0.18–0.68). **b**. The average branch length per family without terminal leaves. **c**. Transfers per substitution in the species tree per chlamydial node for each family. To account for heterogeneous genome sampling in our dataset, we only evaluated transfer rates of chlamydial families with at least two ancestors reconstructed in our analysis, excluding terminal nodes. From left to right, *n* = (14, 18, 15, 10, 5, 7). Transfer rates are significantly higher in ancestors of the Parachlamydiaceae and Rhabdochlamydiaceae than in the Chlamydiaceae (P = 4.8 × 10^−3^ and 8.5 × 10^−3^) and in Anoxychlamydiaceae (P = 7.1 × 10^−3^ and 1.1 × 10^−3^), respectively. Asterisk and lines indicate families with P ≤0.05 based on a one-sided Wilcoxon signed rank test with Bonferroni correction. **d**. Barplot showing the number of significant transfer events (binomial test, significance P ≤0.05; see Methods) in gene highways within (purple) and between (orange) chlamydial families and **e**. corresponding networks depicting chlamydiae (nodes, colour-coded by family; families represented by only one genome are white, see Fig. 1) sharing significant gene highways (edges). Edges are coloured based on gene highways within and between families in purple and orange, respectively. Center lines in the box-and-whisker plots represent median values, box limits represent upper and lower quartile values, and whiskers represent 1.5 times the interquartile range above the upper quartile and below the lower quartile. See also Figure S10 for events across all PVC nodes, and the repository for raw data.

In our analysis, 70% of PVC gene families were found to have evolved vertically, closely mirroring previous reconstructions of bacterial evolution^74^. The remaining 30% represent horizontal gene transmission from within (that is, transfers) or outside the PVC dataset (that is, HGT-derived originations). HGT is known to occur in both horizontally and vertically transmitted endosymbionts (for example, *Wolbachia*), although it is more prevalent in the former^67,71,75–79^. Nevertheless, obligate endosymbionts are expected to be limited in HGT relative to free-living bacteria^9^. A large number of gene originations were reconstructed in Chlamydiae (*n* = 1,458), many of which were probably HGTs from diverse bacterial groups (Extended Data Fig. 4 and Supplementary Data 11). For example, chlamydial sequences affiliate with bacterial groups for 94% of LCCA HGT-derived originations. For many gene originations, chlamydial sequences affiliate with taxonomic groups known for host association, such as Proteobacteria, Bacteroides and CPR bacteria^80^. This pattern is suggestive of HGT facilitated by co-occurring symbionts or phagocytosed prey bacteria, which is common in protists^68–70,81^. We further examined putative HGTs within the PVC dataset (that is, transfer events) and approximated gene transfer rates between ancestors. In Chlamydiae we found lower, but not statistically significant (*P* = 0.068), transfer rates than in other PVC bacteria (Extended Data Fig. 7). Between chlamydial families, Parachlamydiaceae and Rhabdochlamydiaceae had significantly higher transfer rates than Chlamydiaceae (*P* = 4.8 × 10^−3^ and 8.5 × 10^−3^) and Anoxychlamydiaceae (*P* = 7.1 × 10^−3^ and 1.1 × 10^−3^). Overall, gene exchange rates in some chlamydial groups do not differ from free-living PVC bacteria.

Interchlamydial HGT was visualized by testing for statistically over-represented gene transfers between chlamydial nodes where donor and acceptor lineages could be assigned (*n* = 5,937, *P* ≤0.05). The resulting network reveals gene transfer highways indicating probable shared environmental niches, such as shared hosts (Extended Data Fig. 7). Genome sequence divergence is a major barrier to HGT^82^. Despite this, 59% (*n* = 3,493, *P* ≤0.05) of significant transfers occured between members of different chlamydial families. Elevated HGT frequency between more distantly related chlamydiae could be explained by ecological overlap in host or environment^82,83^. Chlamydiaceae, Anoxychlamydiaceae and *Neochlamydia* were under-represented in the network and have isolated transfer highways. These groups have convergently lost central metabolic pathways, including TCA cycle and ETC components (Fig. 4, Extended Data Figs. 5 and 6 and Supplementary Data 10), suggesting adaptation to specialized environments. Extensive gene transfer highways between Amoebachlamydiales members also support divergent genome evolution in this group (Extended Data Fig. 7). Chlamydial gene exchange could be facilitated by the presence of ancestral plasmids, which encode conjugative elements in some lineages including several Parachlamydiaceae^41^. While HGT is well recognized in endosymbiotic bacteria^67,71,75–79^, our study provides a systematic view on the pervasiveness of HGT and the role of intersymbiont transfers in endosymbiont genome evolution.

## Conclusions

In our study we present a comprehensive view of evolution in the Chlamydiae phylum. We found that the Chlamydiae ancestor was already adapted to an endosymbiotic lifestyle and probably infected eukaryotic hosts. We also found that Chlamydiae did not evolve from a metabolically versatile aerobe as expected but rather from a facultative anaerobe. Energy metabolism and oxygen tolerance gene gain later shaped diversification within the phylum. Counter to expectations for obligate endosymbionts, our results show that the protist-infecting Amoebachlamydiales underwent genome expansion and only later gained their characteristic aerobic and metabolic versatility. Together, our results lay a foundation for further investigation of the complex, and perhaps varied, evolutionary trajectories of bacterial endosymbionts.

## Methods

See Extended Data Fig. 1 for an overview of key steps for the reconstruction of gene content evolution in PVC bacteria.

### Selection of representative genomes

A representative dataset of PVC bacteria genomes was selected using genome quality to obtain species-level Chlamydiae representatives and genus-level representatives of other PVC bacteria from the genome taxonomy database (GTDB) and the National Center for Biotechnology Information (NCBI). GTDB is continually updated as genomes are released on NCBI and thus naming structures are nonstationary^84^. Here Chlamydiae were initially classified as a phylum, but in the version used were classified as a class of Verrucomicrobiota (that is, Chlamydiia). All genomes from GTDB v.86 (2018 database) classified as Planctomycetota and Verrucomicrobiota were selected (*n* = 1,183). Non-chlamydial PVC genomes (*n* = 773; Supplementary Data 1) with completeness ≥90% and contamination ≤2%, based on GTDB metadata, were downloaded from NCBI (*n* = 182; 3 April 2019). For Chlamydiae, genomes from GTDB class ‘c__Chlamydiia’ were downloaded from NCBI (*n* = 410; 3 April 2019) and supplemented with recently acquired MAGs and isolate genomes (*n* = 216) for a total of 626 chlamydial genomes. We used miComplete^85^ v.1.1.1 to estimate the quality of chlamydiae genomes using a specific marker gene set^37^ and selected those with completeness ≥0.9 and redundancy ≤1.02 for downstream analysis (*n* = 460; Supplementary Data 1).

To reduce dataset redundancy, all genomes were dereplicated with dRep^86^ v.1.4.3 using previously proposed cutoffs for strain-level delineation^87^—that is, an average nucleotide identity of 96.5% and genome alignment fraction of at least 60%, resulting in 224 genomes (Supplementary Fig. 1 and Supplementary Data 1). Non-chlamydial PVC genomes were further dereplicated by comparing genome quality scores (GQS) per GTDB genus level (Supplementary Data 1). GQS was calculated as described in ref. 88—that is, GQS = completeness (%) – 5 × contamination (%). The highest GQS-scoring genome per genus was selected as a representative and, when two genomes had an equal score one was manually selected (Supplementary Fig. 1). The final dataset included 184 PVC genomes with 95 species-level Chlamydiae representatives and 89 genus-level non-Chlamydiae PVC representatives (47 Planctomycetes, 34 Verrucomicrobia, 5 Lentisphaerae and 3 Kiritimatiellaeota) (Supplementary Fig. 1 and Supplementary Data 2). Genome characteristics were calculated using miComplete^85^ v.1.1.1 (Fig. 1 and Supplementary Data 2). Putative uncharacterized PVC phyla were not included, such as *Candidatus* Omnitrophica^16^ due to its conflicting position in some large-scale species trees of Bacteria^15,88^.

### Phylogenomic analyses

PVC species relationships were inferred using phylogenomic datasets of concatenated single-copy marker genes (Supplementary Fig. 2 and Supplementary Data 3) for the initial 184 selected taxa (Supplementary Fig. 3 and Supplementary Data 2). Additional species phylogenies were inferred after removal of genomes with unresolved phylogenetic positions, resulting in datasets with 183 taxa (removal of *Chlamydiae* bacterium 1070360-7; Supplementary Fig. 4) and 180 taxa (further removal of the 3 Parilichlamydiaceae genomes; Supplementary Fig. 5). ML and Bayesian phylogenies were inferred with and without the removal of compositionally heterogeneous sites for all three datasets (184, 183 and 180 taxa) as outlined below (Supplementary Data 4–6). Species phylogenies were rooted with Planctomycetes based on its phylogenetic position in recent large-scale phylogenomic analyses of bacterial species relationships^88–90^. The 180-taxa dataset was selected for further analyses and the converged Bayesian species phylogeny (consensus of chains 1 and 3), with compositionally heterogeneous sites removed, was used for ancestral state reconstruction (Fig. 1 and Supplementary Discussion 1).

#### Identification of single-copy marker genes

Candidate single-copy marker genes were identified using nonsupervised orthologous groups (NOGs) from eggNOG^91^ v.4.5.1. Protein-coding gene sequences from all PVC bacteria genomes were mapped to NOGs at the last universal common ancestor level (that is, root-level ‘-d NOG’) using emapper^92^ v.1.0.1. Resulting NOGs where 95% of taxa were found in a single copy were identified as candidate markers for further investigation (*n* = 116; Supplementary Data 3).

Sequences from each gene family NOG were aligned using MAFFT L-INS-i^93^ v.7.427 and manually inspected, with poorly aligned and short sequences removed. Alignments were trimmed using BMGE^94^ v.1.12 (entropy score cutoff or ‘-h’ of 0.6). IQ-TREE^95^ v.1.6.11 was used to infer phylogenetic trees, with model selection by ModelFinder^96^ from empirical profile mixture models^97^ combined with the LG exchangeability matrix^98^ (that is, LG + C10 to LG + C60), and with 1,000 ultrafast bootstraps (ufBP)^99^. Resulting trees (available in repository) were manually examined for patterns indicative of vertical inheritance and sufficient phylogenetic signal, and markers were removed that did not generally resolve PVC phyla (Supplementary Data 3). Sequences were removed that could represent HGT events, distant paralogues or contamination (Supplementary Data 3). Where multiple sequences per taxon were present, if they overlapped both were removed (duplicates) and, if they were partial and nonoverlapping, the longer sequence was retained (Supplementary Data 3). A second round of sequence alignment and tree inference was performed as above, with further markers removed resulting in 79 marker genes (Supplementary Data 3).

Discordance filtering^100^ was then performed to remove markers with the most anomalous phylogenetic signal relative to the majority (that is, the most discordant trees). NOGs (all of which were clusters of orthologous groups, that is, COGs) were ranked by discordance score and the top-scoring fraction was removed, leaving 74 single-copy marker genes for phylogenomic analyses (Supplementary Fig. 2). Amino acid sequences for each selected marker gene were realigned and trimmed, as above, after removal of taxa with unresolved phylogenetic positions (that is, datasets with 183 and 180 taxa). Trimmed amino acid alignments were concatenated into a supermatrix for each of the three datasets.

#### Phylogenomic inferences

Heterogenous site removal was performed using *χ*^2^-trimming^101^, with the most compositionally heterogeneous sites removed from each concatenated alignment in incremental steps of 1% of alignment sites. Site removal continued until no taxa significantly heterogeneous in their amino acid composition remained (based on the *χ*^2^ test score statistic; significance *P* ≤0.05; Supplementary Figs. 3–5 and Supplementary Data 4).

Using IQ-TREE^95^ v.1.6.10 with model selection^96^, ML phylogenies were inferred for the initial unrefined alignment, for alignments in 10% increments of total sites removed based on *χ*^2^-trimming (up to 50%; Supplementary Data 4), and for the alignment with no significantly heterogeneous taxa (fully refined alignment). ML trees were then reconstructed using the posterior mean site frequency (PMSF) approximation of the LG + C60 + F + Γ4 model (selected in all initial trees) with 100 nonparametric bootstraps. Transfer bootstrap expectation^102^ bipartition support was also inferred for the initial unrefined alignment and for the fully refined alignment using IQ-TREE^103^ v.2.0.

Bayesian phylogenies were reconstructed for these two alignments for all three taxa datasets. In each case, four independent Markov chain Monte Carlo chains were run using PhyloBayes-MPI v.1.7b^104^ with the CAT + GTR + Γ4 model^97,105^, for at least 10,000 iterations. CAT, a site-heterogeneous model, performs more robustly against long-branch attraction artefacts^106^. If at least 10,000 iterations had been run but no chains had begun to converge (maximum difference <1), all chains were stopped. The number of generations, burn-in and any chain convergence (maximum difference <0.3) can be found in Supplementary Figs. 3–5 alongside a consensus tree of all four chains with posterior probability (*P*) indicating branch support. Posterior predictive checks were also performed with PhyloBayes-MPI v.1.7b^104^, with configurations sampled every ten generations after burn-in. The resulting range of *z*-scores for maximum heterogeneity and diversity across chains can be found in Supplementary Figs. 3–5. See Supplementary Data 6 for all uncollapsed species phylogenies and Supplementary Data 5 for a summary of the number of taxa, alignment lengths, inference methods, bootstrap supports and model of evolution for each phylogeny.

### 16S rRNA gene species phylogeny

Near-full-length 16S rRNA gene sequences from chlamydiae (*n* = 233) and other PVC members (*n* = 205) were downloaded from SILVA^107^ v.138 SSU Ref NR 99, 79 near-full-length chlamydial 16S rRNA gene sequences (97% identity operational taxonomic unit representatives) retrieved from Schulz et al.^22^ and 142 sequences from our reference genome dataset. Sequences (*n* = 659) were clustered at 90% sequence identity to reduce redundancy using USEARCH^108^ v.11.0.667 with ‘-cluster_smallmem’. The resulting family-level sequence representatives (*n* = 177) were aligned with SINA^109^ and the alignment trimmed with trimAl^110^ v.1.4.1 ‘-gappyout’ (1,533 aligned positions). Bayesian tree samples with four Markov chain Monte Carlo chains in parallel (*n* = 100,000 each) were inferred under the CAT + GTR + Γ4 model^97,105^ in PhyloBayes v.4.1c^111^ (Supplementary Fig. 6). Convergence was assumed once maximum difference dropped below 0.3 and effective sample sizes for continuous parameters were >100 (according to the commands 'bpcomp' and 'tracecomp' in PhyloBayes, respectively) after burn-in (*n* = 25,000).

### Generation of gene families and trees

#### NOG clustering

PVC gene sequences from the 180-taxa dataset (*n* = 445,591) were mapped against eggNOG^91^ v.4.5.1 using emapper^92^ v.1.0.1 at root-level ‘-d NOG’. Of these, 326,083 (73%) gene sequences were assigned to 17,935 NOGs (Supplementary Fig. 7).

#### De novo clustering

For the remaining 119,508 gene sequences in the 180-taxa dataset with no homologue in eggNOG v.4.5.1, we performed pairwise sequence alignment in an all-against-all fashion with DIAMOND^112^ v.0.9.21 using the parameter ‘–more-sensitive’. Subsequently, de novo clustering with SiLiX^113^ v.1.2.9 was performed with default overlap of 80% and identity thresholds ranging from 5 to 40% in 5% increments (Supplementary Fig. 7). To select an appropriate identity threshold we (1) inspected the number of singleton clusters per threshold and (2) assigned TIGRFAM^114^ v.15.0 domains with InterProScan^115^ v.5.36-75.0 to gene sequences. Using the assigned TIGRFAMs, true positive rate (sensitivity) and true negative rate (specificity) were calculated for clusters, with different clusterings evaluated using the balanced accuracy measure ((specificity + sensitivity)/2) as suggested^113^. A 25% identity cutoff performed best, yielding 10,548 de novo gene families with at least two members (75,218 singletons).

#### Gene family phylogenetic trees

We performed phylogenetic analysis on all gene families (both NOG and de novo clusters) with at least four members (*n* = 11,996). Sequences were aligned with MAFFT^116^ v.7.427 using the strategy ‘–localpair’. Alignments were then trimmed using BMGE^94^ v.1.12 with default parameters and an entropy cutoff of 0.6. The permitted gap rate for alignment positions was increased to 0.5 for 94 gene families with <50 informative aligned positions using the initial parameters. Gene trees were then inferred with IQ-TREE^95^ v.1.6.11 using the best-fit model identified by ModelFinder^96^, with ‘-m TESTNEW’, ‘-madd LG + C10, LG + C20, LG + C30, LG + C40, LG + C50, LG + C60’ and 1,000 improved ufBPs^99^ ‘-bnni’. Two specific gene families were later excluded (COG3119 and COG0457) due to poor alignment (probably caused by repeat regions) and subsequent difficulties inferring a ML tree, bootstraps from which are required for ALE. These gene families primarily occur in non-Chlamydiae PVC members and thus should not impact chlamydial ancestor proteome sizes (Supplementary Data 7). The remaining 91,705 gene families had three or fewer sequences. Thus, phylogenetic trees could not be inferred. However, because ALE requires gene families in a tree format we thus provided mock trees for those with two or three sequences.

#### Annotation of gene families

We assigned protein domain annotations to gene families using InterProScan^115^ v.5.36-75.0 to identify the domains of Protein Families (Pfam)^117^, TIGRFAM^114^ and InterPro (IPR)^118^. We assigned Kyoto Encyclopedia of Genes and Genomes orthology and enzyme commision numbers using GhostKOALA^119^ and inferred eggNOG^91^ functional annotation as described above, and also at the bacterial level (‘-d BACT’).

### Ancestral state reconstruction

To gain a more complete perspective on PVC genome evolution we used the complete genome dataset outlined above, which includes MAGs from uncultured lineages that would otherwise be missed. However, this was restricted to high-quality MAGs as outlined above (completeness ≥90% and contamination ≤2%).

#### Gene-tree-unaware method, Count

For gene-tree-unaware ancestral gene content reconstruction, we ran Count^120^ v.10.04 with the gain–loss–duplication model of evolution with Poisson distribution to model gene family size at the root. We used the same gain–loss and duplication–loss ratios for all lineages and inferred ancestral gene content using the Wagner maximum parsimony framework with default costs.

#### Gene-tree-aware method, ALE

ML tree bootstrap samples of gene families identified in the PVC dataset were reconciled with the species tree to reconstruct their gene family histories. We computed conditional clade probabilities from bootstrap samples (ALEobserve) and sampled 100 reconciliations with the species tree (ALEml_undated) using ALE^121^ v.0.449, implemented as a computational pipeline (https://github.com/maxemil/ALE-pipeline). We added singleton gene families as originations at the corresponding species node to the reconstructions. Furthermore, the estimated fraction of missing gene content per genome was provided to ALE because it uses this to correct for potentially missing data—that is, in the MAGs included. In addition, the specific implementation of ALE corrects ancestor gene copy number estimates using modelled gene extinction probability rates^44^, which has previously been employed to estimate ancestral proteome sizes^122^.

#### Comparison of ancestral state reconstruction methods and selection of ALE cutoff

ALE improves on earlier methods by direct estimation of rates of gene duplication, transfer and loss from data, as well as incorporating the uncertainty in gene trees while exploring a larger gene tree space^121^. The accuracy of reconstructions can be negatively influenced by an inaccurate species tree and imbalanced taxon sampling^44,121^. Here, these risks are minimized due to our extensive taxon sampling and species tree reconstruction efforts (Supplementary Figs. 1–6 and Supplementary Data 1–6).

ALE reports relative frequencies for ancestral events and gene family copy frequencies that express their statistical support. This support accumulates the uncertainty introduced by alignment, tree reconstruction and reconciliation and should therefore not be set at a standard-level cutoff. We therefore aimed to identify a suitable threshold by investigating density distribution per inferred event type and transfer ratio per gene family (Supplementary Fig. 8), which indicated a cutoff of 0.3 for a candidate with high signal-to-noise ratio. The identified cutoff is in accordance with recent similar analyses that selected 0.3 as a frequency cutoff^123,124^. The transfer ratio represents the proportion of horizontal events over all events per gene tree. We further compared the tree-aware reconstructions generated by ALEml with the thresholds 0.3 (sensitive), 0.5 (specific) and 0.7 (very specific) using gene-content-only-aware Count reconstructions (Supplementary Fig. 9). The highest number of consensus gene families obtained with gene-tree-aware and -unaware methods was reached with a threshold of 0.3 (Supplementary Fig. 9a). Based on this analysis and event density distributions, we selected a frequency cutoff of 0.3 for inferring evolutionary events in our ancestral state reconstruction analysis (Supplementary Figs. 8 and 9). Gene families were thus inferred as present when reconstructing ancestor gene content if they had a copy frequency of at least 0.3. In addition, we used this cutoff to calculate confident-event frequencies. This meant that an event frequency ≥0.3 and <1.3 was counted as 1, a frequency ≥1.3 and <2.3 was counted as 2 and so on. These confident-event frequencies correspond to the gene content and events used in our ancestral reconstructions (Figs. 2 and 4, Supplementary Figs. 10–12 and Extended Data Figs. 2–6). However, for estimation of genome content evolution dynamics and ancestral proteome size, raw reconciliation frequencies were used to avoid potential underestimation of transfers and losses (Figs. 3 and 5 and Extended Data Fig. 7).

### Inference of transfer rates and gene transfer highways

We approximated the rate of intra-PVC HGT (that is, transfer rate) for nodes in the species tree by calculating the inferred gene transfers in our reconstructions divided by the number of substitutions in the species tree along the given branch. Based on shared gene families between two extant or ancestral genomes, we tested whether more HGT events occured between genomes than the median of transferred gene families within chlamydiae members. We used a one-sided binomial test (‘binom.test’) with ‘alternative = greater’ in the R base package^125^, and false discovery rate corrected *P* values for multiple testing with ‘p.adjust’ to identify enriched transfer routes (‘gene transfer highways’) with *P* ≤0.05. Significant gene transfer highways were visualized with Cytoscape^126^ v.3.7.0.

### Identification of non-PVC gene transfer donors

For genes inferred as originations within Chlamydiae, to distinguish bona fide HGTs from outside the PVC dataset and candidate de novo gene families, we performed a homology search against the NCBI nonredundant database. If no homologous protein sequences could be identified, gene families were referred to as de novo candidates, otherwise we inferred gene trees to identify affiliated taxonomic groups and thus potential donor lineages of the horizontally transferred gene (workflow: https://github.com/jennahd/HGT_trees). For each gene family a DIAMOND v.0.9.36.137 blastp^112^ search (with ‘max-target-seqs 2000’ and ‘more-sensitive’) was performed using all sequences against NCBI’s nonredundant database^127^ (v.5, accessed 8 October 2020). Unique hits per gene family were compiled and clustered using CD-HIT^128^ v.4.8.1 at 80% sequence identity. NCBI’s taxonomy database^129^ was used for taxonomic classification. Protein sequences from each gene family and any database hits were aligned with MAFFT^93^ v.7.471 (‘–auto’) and trimmed with trimAl^110^ v.1.4.rev15 (‘gappyout’). Sequences that covered <40% of the trimmed alignment were removed, followed by inference of an initial phylogenetic tree using FastTree^130^ 2 v.2.1.11. Long-branching taxa were identified as having outlier terminal branch lengths (third quartile + 1.5× interquartile range) relative to others in each tree, and were removed before reinferring trees as above.

These initial trees were prohibitively large for performing ML analyses and smaller subtrees were therefore selected using the above workflow. Here, clades comprising ≥25% chlamydiae with at least two chlamydial sequences were identified. To account for multiple HGT events, per gene family up to three clades with the largest number of chlamydial sequences were identified although, in the majority of cases, only one was found. Subtrees including these clades were selected by finding nodes at least three further up the tree hierarchy and that included at least 150 additional taxa and up to 400 additional taxa with bipartition support ≥0.7. Where a subtree fulfilling these conditions was identified, but with a larger number of taxa, the number of taxa was reduced to ≤400 by removal of more distant sequences (support ≥0.7, at least five sequences and at least six steps until a common ancestor with the chlamydial clade). Between 20 and 50 outgroup sequences were randomly selected from the clade with a position sister to the selected subtree (moving to the next subtending clade when there were <20 initial outgroup sequences). Selected subtrees were subsequently aligned and trimmed and an initial phylogenetic tree was inferred as above. ML trees were then inferred for each subtree using the trimmed alignment with IQ-TREE^95^ v.1.6.12 under the LG model of evolution^98^, with 1,000 ufBP. The clade sister to chlamydial sequences, and that subtending this clade (‘nested’) with ufBP ≥80, were identified. Taxonomic labels of sister and nested taxa were each compared at domain, superphylum and phylum levels. The lowest-level shared taxonomy at cutoffs of 75% (Extended Data Fig. 7), 90% and 100% of taxa was selected as the affiliated taxonomic group and hence putative gene donor (Supplementary Data 11). Originations were identified as de novo in the case where no non-chlamydial hits were found or when no sister group to chlamydial sequences was inferred.

### Reconstruction of metabolic pathways

The evolutionary history of ETC components was investigated using the workflow described above. We reconstructed ancestral gene family repertoires from ALE by selecting all families predicted to be present at a given node with a relative frequency ≥0.3. We assessed the metabolic capabilities of ancestral genomes using either the Kyoto Encyclopedia of Genes and Genomes Module tool^131^ or MetaCyc pathways^132^.

### Statistics and data visualization

Phylogenetic trees and protein domains were visualized using Figtree v.1.4.4 (http://tree.bio.ed.ac.uk/software/figtree), iTOL^132^ and the ETE3 Toolkit^133^ v.3.1.2. Relative evolutionary divergence of chlamydiae ancestors in the species tree was calculated using PhyloRank^84^ v.0.1.10 (https://github.com/dparks1134/PhyloRank). Plots were generated using Cytoscape^126^ v.3.7.0 and the R v.3.6.2 base package^125^ alongside the packages ggplot2 (ref. 134) v.3.3.3, ggtree^135^ v.2.5.0.991 and treeio^136^ v.1.10.0.

### Reporting summary

Further information on research design is available in the Nature Portfolio Reporting Summary linked to this article.

## Supplementary information

Supplementary Information Supplementary Discussions 1 and 2, Figs. 1–12 and Data 1–11.

Reporting Summary

Peer Review File

Supplementary Data Supplementary Data 1–5 and 7–11, as outlined in Supplementary Information.

Supplementary Data Supplementary Data 6 as outlined in Supplementary Information.

## Data Availability

Genome data was obtained either from NCBI Genbank (https://www.ncbi.nlm.nih.gov/nucleotide), the JGI portal (https://portal.nersc.gov/GEM) or a zenodo repository (10.5281/zenodo.4318714). 16S rRNA gene data used in this study are available via the SILVA database (https://www.arb-silva.de). Genbank accessions and database links for genomes used in the ancestral state reconstruction are provided in Supplementary Data 2. Additional raw data files are hosted on the online repository figshare (10.6084/m9.figshare.17033417). These include sequences, alignments, trimmed alignments and trees for single-copy marker genes used for species phylogenies (both those selected and not selected), the 16S rRNA gene alignment and tree, as well as concatenated alignments and trees for all three species datasets (of 184, 183 and 180 taxa). Both NOG and de novo gene families used for the ancestral state reconstruction are also provided along with alignments, trimmed alignments, trees and bootstrap trees (ufboot) provided to ALE. The raw ALE results with all events are also included, along with gene annotations and events, and events for each gene family mapped to the species tree. Protein sequence datasets, alignments and trees inferred as part of the analysis to determine HGT donors for chlamydiae gene originations are provided. In addition, PDFs of metabolic reconstructions of LVCCA, LG1CA and LG2CA can be found in the repository files.

## Code availability

The Nextflow pipeline for running the ALE ancestral state reconstruction (https://github.com/maxemil/ALE-pipeline) and the Snakemake pipeline for identification of putative HGT donor groups (https://github.com/jennahd/HGT_trees) are available on GitHub.
