# Supplementary material for: Gene gain facilitated endosymbiotic evolution of Chlamydiae
Source: Nat Microbiol. 2023 Jan 5;8(1):40–54. doi: 10.1038/s41564-022-01284-9 (PMC9816063; doi:10.1038/s41564-022-01284-9)
Supplement: Supplementary file 1 — Supplementary Discussions 1 and 2, Figs. 1–12 and Data 1–11. [file 41564_2022_1284_MOESM1_ESM.pdf]

nature microbiology

Article https://doi.org/10.1038/s41564-022-01284-9

Gene gain facilitated endosymbiotic evolution of Chlamydiae

In the format provided by the authors and unedited

SUPPLEMENTARY INFORMATION

Gene gain facilitated endosymbiotic evolution of Chlamydiae

Jennah E. Dharamshi†[1], Stephan Köstlbacher†[2,3,4], Max E. Schön[1], Astrid Collingro[2], Thijs J. G. Ettema*[1,4], Matthias Horn*[2]

[1] Department of Cell and Molecular Biology, Science for Life Laboratory, Uppsala University, SE-75123 Uppsala, Sweden
[2] University of Vienna, Centre for Microbiology and Environmental Systems Science, 1030 Vienna, Austria
[3] University of Vienna, Doctoral School in Microbiology and Environmental Science, 1030 Vienna, Austria.
[4] Laboratory of Microbiology, Wageningen University, 6708 WE Wageningen, The Netherlands

† Equal contribution

* Correspondence to: thijs.ettema@wur.nl and matthias.horn@univie.ac.at

SUPPLEMENTARY DISCUSSIONS 2

Supplementary Discussion 1 - Inconsistencies in Chlamydiae phylogenetic relationships 2

Supplementary Discussion 2 - Taxonomic descriptions and adjustments 2

Reclassification of the order *Chlamydiales* ord. corrig. (Kuo, Horn and Stephens, 2011) 3

Description of *Amoebachlamydiales* ord. nov. 3

Description of *Simkaniales* ord. nov. 3

Description of *Anoxychlamydiales* ord. nov. 3

Description of *Anoxychlamydiaceae* fam. nov. 4

SUPPLEMENTARY FIGURES 5

SUPPLEMENTARY DATA 22

SUPPLEMENTARY REFERENCES 23

SUPPLEMENTARY DISCUSSIONS

Supplementary Discussion 1 - Inconsistencies in Chlamydiae phylogenetic relationships

The Parilichlamydiaceae are fish pathogens with reduced genomes that resemble the animal pathogen Chlamydiaceae[1], and yet they have been placed sister to all other Chlamydiae in species reconstructions[1–4]. However, in our analyses of concatenated marker genes the phylogenetic position of Parilichlamydiaceae was unstable, indicating possible long-branch attraction (LBA) artifacts[5] (Figures S3-S4). Counter to prior suggestions of convergent evolution, Parilichlamydiaceae and Chlamydiaceae formed a monophyletic group in a Bayesian phylogeny of PVC 16S rRNA genes (Figure S6). Despite their interesting biology and likely importance in Chlamydiae evolution, we were unable to confidently resolve the position of Parilichlamydiaceae and thus chose to remove them. Likewise, the phylogenetic placement of the recently described orphan lineage *Chlamydiae* bacterium 1070360-7[4] was inconsistent and removed from further analyses (Figure S3).

Several long-branching chlamydial lineages formed a clade sister to other chlamydiae in initial species trees, but were found to be well-supported together with Simkaniaceae in Bayesian inferences with the CAT model of evolution (Figures 1 and S3-S5), which is known to alleviate LBA artifacts caused by fast-evolving sequences[6,7]. In maximum-likelihood (ML) phylogenies the position of these Simkaniaceae-like lineages was also reconstructed as forming a monophyletic group with Simkaniaceae, but only with the removal of compositionally heterogeneous sites. The removal of such sites from sequence alignments reduces systematic error in phylogenomic analyses by alleviating the artifact of species grouping together based on shared biases in amino acid composition[8]. Based on these results, we thus classified this Simkaniaceae-like group as putatively belonging to the Simkaniaceae family and included it as such for our ancestral reconstruction (Figures 1 and S3-S5). A similar pattern was seen for *Chlamydiae* bacterium 3300009703-49 which initially affiliated with Anoxychlamydiaceae (formerly Anoxychlamydiales[9]), but was well-supported together with Chlamydiae Clade III (CC-III) once accounting for compositional heterogeneity (Figures 1 and S3-S5). CC-III and Anoxychlamydiaceae form a well-supported clade we refer to here as the order Anoxychlamydiales. Likewise, the position of *Chlamydiae* bacterium K940_chlam_8, another long-branching lineage, is supported with methods accounting for LBA and compositional bias artifacts (Figures 1 and S3-S5).

In the final dataset of 180 PVC bacteria genomes, all deep evolutionary relationships were consistently resolved in both Bayesian and ML inferences when compositionally heterogeneous sites were removed. However, in the Bayesian analysis the topology within Chlamydiaceae differed (chains 1 and 3 converged, and chains 2 and 4 converged). For the ancestral reconstruction we used the consensus Bayesian phylogeny (chains 1 and 3) with stronger convergence and where the topology in Chlamydiaceae was consistent with ML inferences.

Supplementary Discussion 2 - Taxonomic descriptions and adjustments

Previous suggestions to divide the phylum Chlamydiae into several orders have not been accepted by the Subcommittee on the Taxonomy of Chlamydiae of the International Committee on Systematics of Prokaryotes (ICSP) due to the lack of data[10,11], but high quality data for many diverse chlamydiae have become available within the past years[2–4]. By including this additional data and a large number of non-chlamydial PVC genomes (n=89), a stable chlamydial species phylogeny could be inferred with Bayesian and ML tree inference methods (see Methods, Figures

1, S1 and S3-S6, Extended Data Figure 1, Data S1-S6). However, this was only possible after removing two family-level lineages with unstable branching patterns (*Candidatus* Parilichlamydiaceae and *Candidatus* MCF-D; see Supplementary Discussion 1) from the final dataset. Based on the single-copy marker gene species tree inferred in this study and lately published chlamydial phylogenies for marker genes and the 16S rRNA gene, we propose the reclassification of the order levels within the phylum Chlamydiae[3,4,12].

Reclassification of the order *Chlamydiales* ord. corrig. (Kuo, Horn and Stephens, 2011)
The order *Chlamydiales* is so far the only officially accepted one within the *Chlamydiae* and includes all previously described family-level lineages[11,13]. Based on the stable monophyletic branching in concatenated marker gene and 16S rRNA gene tree inferences, we propose to reclassify the *Chlamydiales* and to only include members of the *Chlamydiaceae*[14], *Candidatus* Clavichlamydiaceae[15], and *Candidatus* Sororchlamydiaceae (formerly Chlamydiae Clade IV or CC-IV)[3,16] in this order.

Description of *Amoebachlamydiales* ord. nov.
(A.moe.ba.chla.my.di.a'les. N.L. fem. n. *Amoeba* derived from Gr. amoibe; N.L. fem. *Chlamydia* taxonomic name of a bacterial genus; L. suff. *-ales* ending to denote an order; *Amoebachlamydiales* N.L. fem. pl. n. referring to Amoebozoa as the major known hosts of members of the order)

The order *Amoebachlamydiales* represents a distinct monophyletic lineage as supported by concatenated marker genes and 16S rRNA gene trees. The order includes members of the families *Criblamydiaceae*[17], *Waddliaceae*[18] and *Parachlamydiaceae*[19]. All of these families have cultivated representatives that thrive in Amoebozoa hosts. Additional families whose representatives have so far only been recovered from genomic data, including *Candidatus* MCF-F, *Candidatus* MCF-G, *Candidatus* K940_chlam_3, and *Candidatus* GCA-270938 should be included in this order[3,4]. Members of the *Amoebachlamydiales* often have extended genetic repertoires for aerobic respiration and other metabolic pathways compared to most other chlamydiae. Their genome sizes range between 2-4 Mb.

Description of *Simkaniales* ord. nov.
(Sim.ka.ni.a'les. N.L. fem. n. *Simkania* type genus of the order; L. suff. *-ales* ending to denote an order; N.L. fem. pl. n. *Simkaniales* referring to the order that includes the type genus *Simkania*)

The order *Simkaniales* represents a distinct monophyletic lineage as supported by concatenated marker gene and 16S rRNA gene trees. It includes members of the family-level lineages *Simkaniaceae* and *Rhabdochlamydiaceae*[19,20].

Description of *Anoxychlamydiales* ord. nov.
(An.oxy.chla.my.di.a'les. Gr. pref. *An*- not; N.L. neut. n. *oxygenium* chemical element oxygen; N.L. fem. *Chlamydia* taxonomic name of a bacterial genus; L. suff. *-ales* ending to denote an order; *Anoxychlamydiales* referring to the potential anoxic lifestyle of some members of this order)

The order *Candidatus* Anoxychlamydiales represents a distinct monophyletic lineage as supported by concatenated marker gene and 16S rRNA gene trees. It includes members of the family-level lineages *Candidatus* Anoxychlamydiaceae and *Candidatus* Chlamydiae Clade III (CC-III)[3].

Description of *Anoxychlamydiaceae* fam. nov.
(An.oxy.chla.my.di.a.ce'ae. Gr. pref. *An*- not; N.L. neut. n. *oxygenium* chemical element oxygen; N.L. fem. *Chlamydia* taxonomic name of a bacterial genus; L. suff. *-aceae* ending to denote a family; *Anoxychlamydiaceae* referring to the potential anoxic lifestyle of members of this family)

The family *Anoxychlamydiaceae* represents a distinct monophyletic lineage as supported by concatenated marker gene and 16S rRNA gene trees. Members of this family so far are only represented by metagenome-assembled genomes[3,4]. Members of this family encode the arginine deiminase pathway, [FeFe]-hydrogenase, and a pyruvate:ferredoxin oxidoreductase indicating an obligate anoxic lifestyle for these organisms. In addition many oxygen-dependent genes are missing in the genomes of the *Anoxychlamydiaceae*.

SUPPLEMENTARY FIGURES

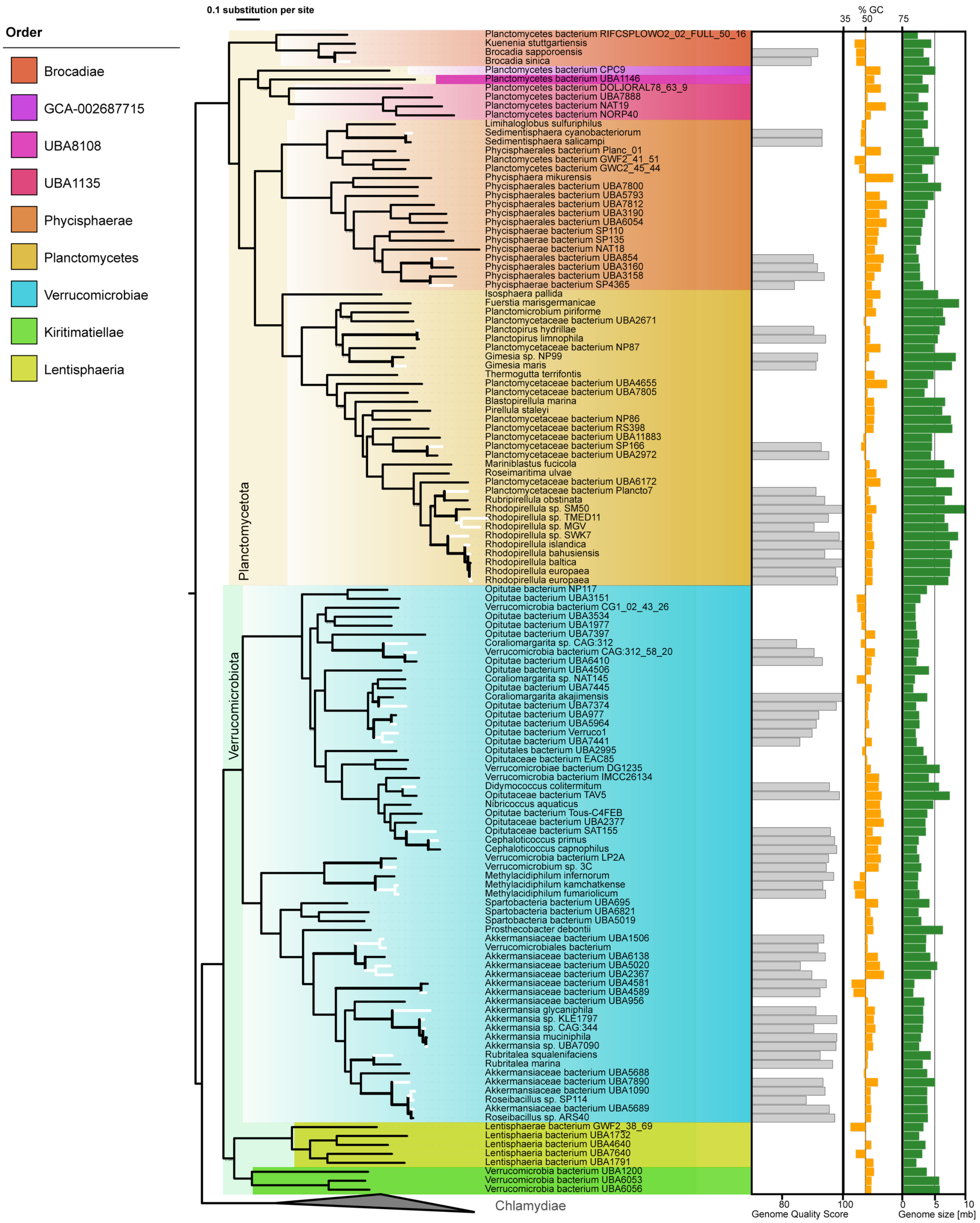

Figure S1. Genus-level dereplication of non-Chlamydiae PVC genomes based on genome quality score. The phylogenetic tree, for representation purposes, is based on 120 bacterial single-copy marker genes[21] inferred with FastTree v2.1[22]. Clades are coloured by GTDB[21] assigned order rank. Black leafs in the species tree represent the selected genomes for downstream analysis, while white leafs were discarded because of higher quality genomes in the same genus. The inner bar chart depicts the genome quality score of genomes in genera with more than one member. The middle chart represents % GC deviation from 50, the outer chart depicts the genome size. See also Data S1-S2.

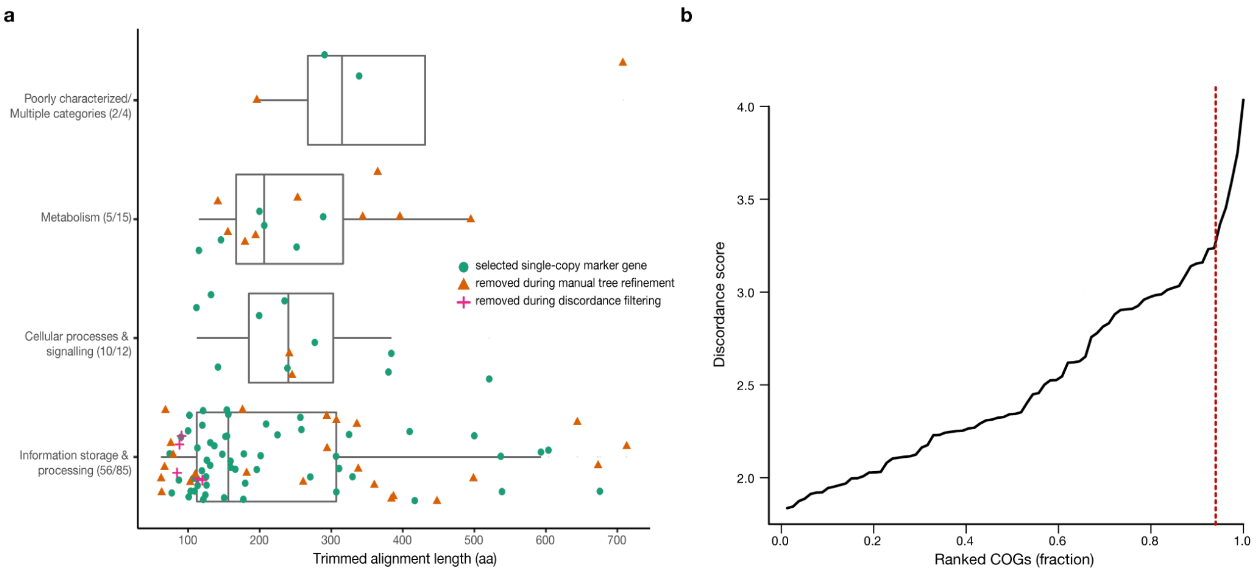

Figure S2. a. Boxplot of putative marker genes for inferring species trees and the length of the corresponding trimmed protein alignment. Marker genes are split into larger COG categories with symbols indicating whether it was removed during manual tree refinement (orange triangles), removed during discordance filtering (pink crosses), or selected for use in concatenated species phylogenies (green circles). Center lines in the box-and-whisker plot represent median values, box limits represent upper and lower quartile values, and whiskers represent 1.5 times the interquartile range above the upper quartile and below the lower quartile. Number of genes included in the box-and-whisker plots from top to bottom n=(4, 15, 12, 85). **b**. Marker gene COGs that passed manual tree refinement are ranked according to discordance score[23]. The red line indicates the fraction of marker genes (n=5) that were removed based on having the largest discordance from other trees. See also Data S3.

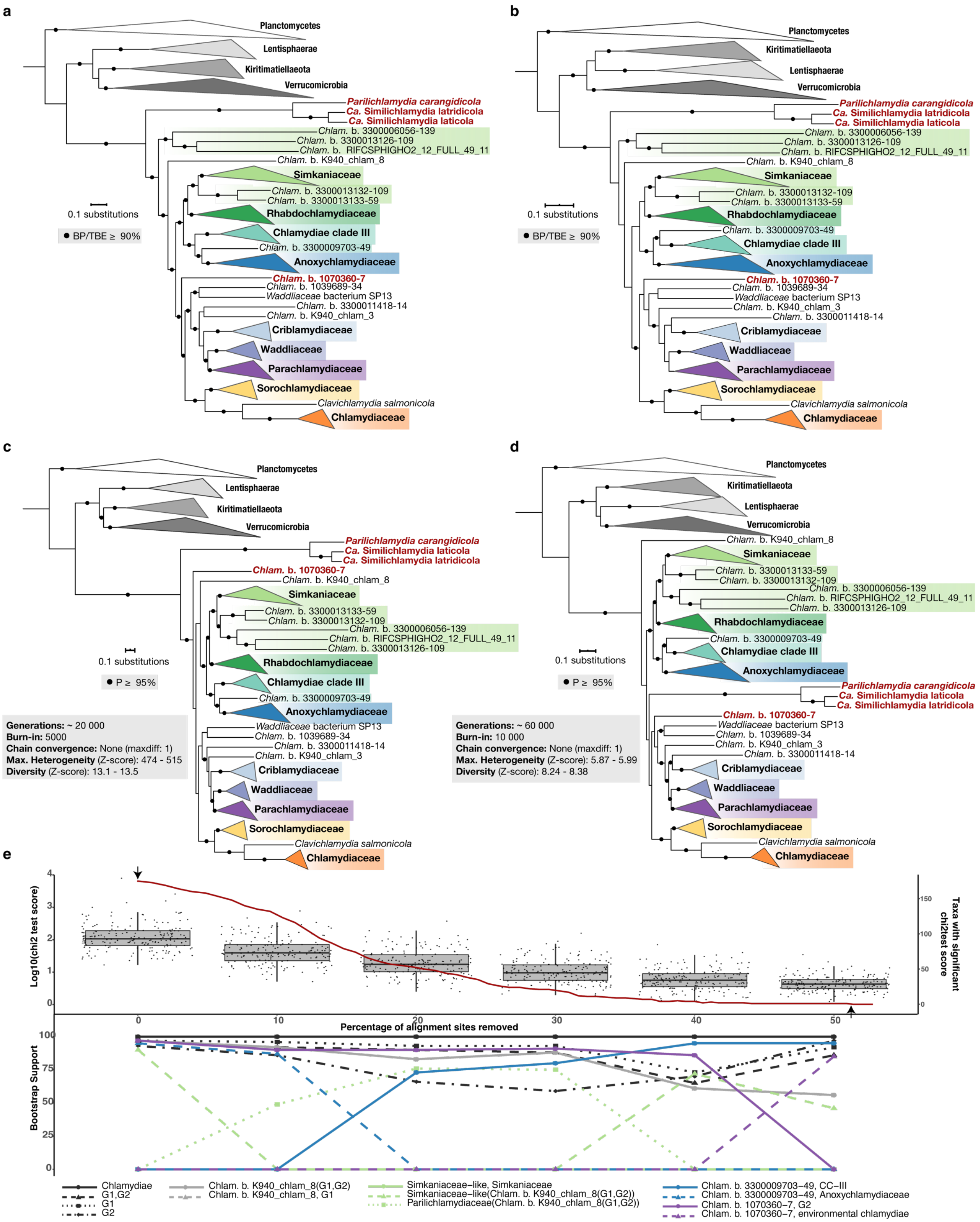

Figure S3. Maximum-likelihood (**a,b**) and Bayesian consensus (**c,d**) species phylogenies of concatenated single-copy marker genes from all PVC bacteria representatives (n=184 taxa) were inferred with the complete alignment (**a,c**) and with compositionally heterogeneous sites removed (**b,d**). High support for bipartitions is indicated by a black circle, with support measured by non-parametric bootstraps (BP) and the transfer bootstrap expectation (TBE) inferred using IQ-TREE with the LG+C60+F+Γ4-derived PMSF approximation, or posterior probability (P) inferred with Phylobayes using the CAT+GTR+Γ4 model of evolution. Consistent clades are collapsed with taxonomy indicated and chlamydial families coloured. Taxa with unclear phylogenetic affiliation are coloured red. Run characteristics and

converged chains are indicated for Bayesian phylogenies in a grey box (**c,d**). Scale bars indicate the number of substitutions per site. **e.** The 1% most compositionally heterogeneous sites were removed incrementally starting from the initial alignment. Boxplots show the log range of $\chi 2$ test scores (scale on the left) across taxa from alignments with the corresponding percentage of sites removed. The red line (scale to the right) indicates the number of taxa with significant $\chi 2$ test scores, and hence deviation from patterns of amino acid composition found in other taxa. Center lines in the box-and-whisker plot represent median values, box limits represent upper and lower quartile values, and whiskers represent 1.5 times the interquartile range above the upper quartile and below the lower quartile. Arrows indicate the initial alignment (**a,c**) and the percentage of alignment sites removed for no remaining taxa with significant deviations in composition (**b,d**). Changes in BP support for the monophyly of different groups of interest is shown in the bottom line plots, at different intervals of percentage sites removed. Abbreviations and short forms include: *Chlam* b. (*Chlamydiae* bacterium), G1 (Group 1), G2 (Group 2), and CC-III (Chlamydiae Clade III). See also Data S4-S6.

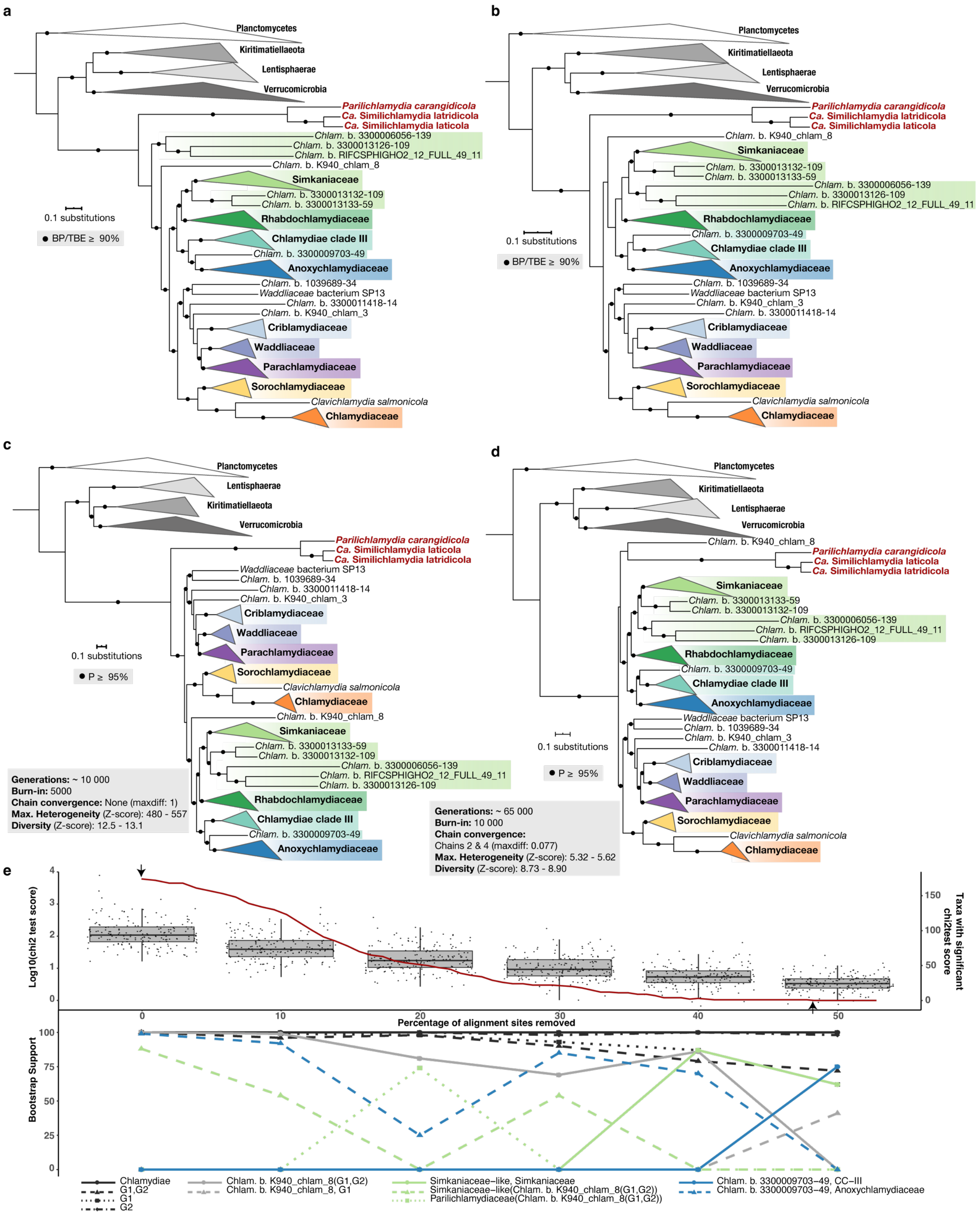

Figure S4. Maximum-likelihood (**a,b**) and Bayesian consensus (**c,d**) species phylogenies of concatenated single-copy marker genes from PVC representatives with the removal of *Chlamydiae* bacterium 1070360-7 (n=183 taxa) were inferred with the complete alignment (**a,c**) and with compositionally heterogeneous sites removed (**b,d**). High support for bipartitions is indicated by a black circle, with support measured by non-parametric bootstraps (BP) and the transfer bootstrap expectation (TBE) inferred using IQ-TREE with the LG+C60+F+Γ4-derived PMSF approximation, or posterior probability (P) inferred with Phylobayes using the CAT+GTR+Γ4 model of evolution. Consistent clades are collapsed with their taxonomy indicated and chlamydial families coloured. Taxa with unclear phylogenetic affiliation

are coloured red. Run characteristics and converged chains are indicated for Bayesian phylogenies in a grey box (**c,d**). Scale bars indicate the number of substitutions per site. **e.** The 1% most compositionally heterogeneous sites were removed incrementally starting from the initial alignment. Boxplots show the log range of $\chi2$ test scores (scale on the left) across taxa from alignments with the corresponding percentage of sites removed. The red line (scale to the right) indicates the number of taxa with significant $\chi2$ test scores, and hence deviation from patterns of amino acid composition found in other taxa. Center lines in the box-and-whisker plot represent median values, box limits represent upper and lower quartile values, and whiskers represent 1.5 times the interquartile range above the upper quartile and below the lower quartile. Arrows indicate the initial alignment (**a,c**) and the percentage of alignment sites removed for no remaining taxa with significant deviations in composition (**b,d**). Changes in BP support for the monophyly of different groups of interest is shown in the bottom line plots, at different intervals of percentage sites removed. Abbreviations and short forms include: *Chlam* b. (*Chlamydiae* bacterium), G1 (Group 1), G2 (Group 2), and CC-III (Chlamydiae Clade III). See also Data S4-S6.

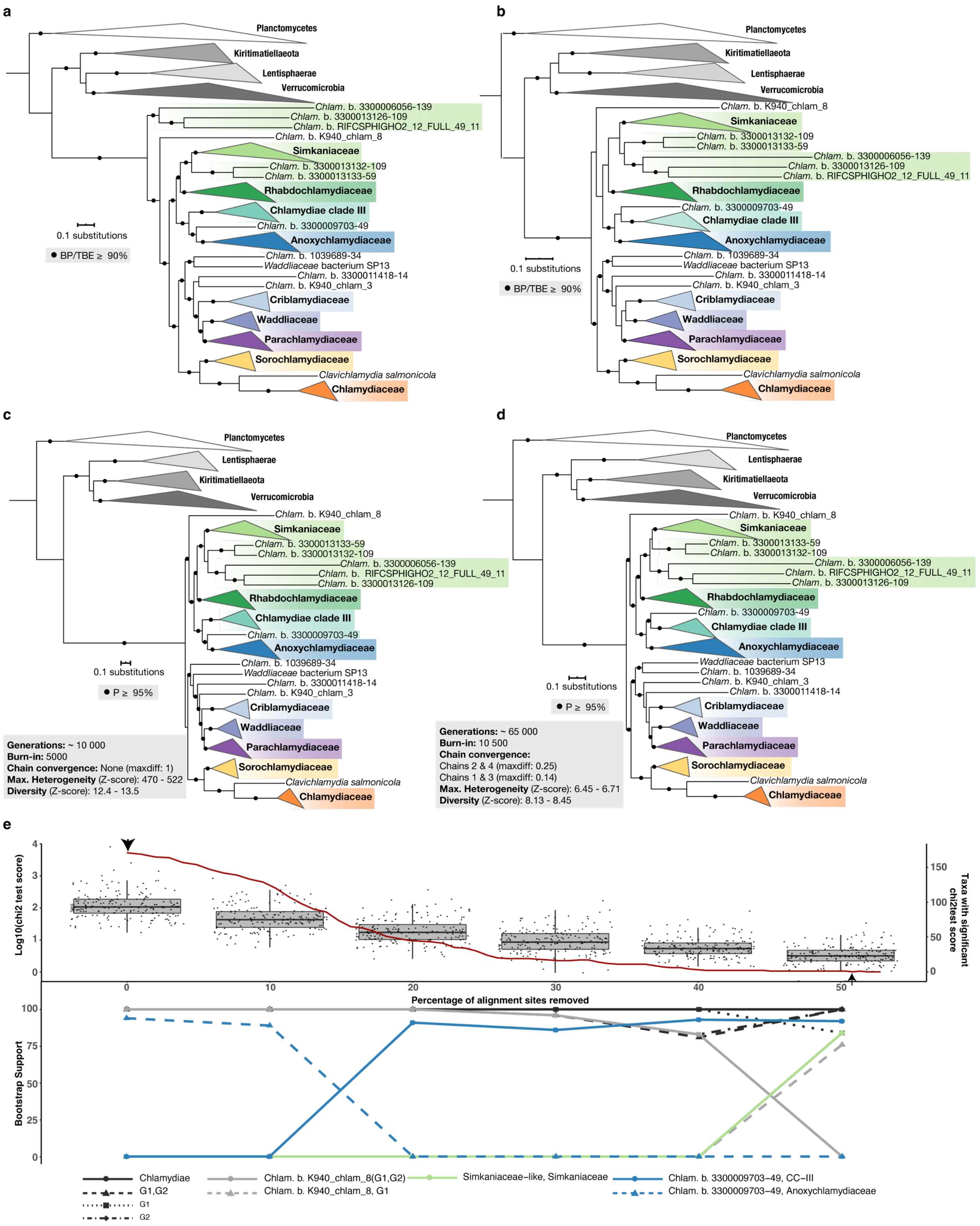

Figure S5. Maximum-likelihood (**a,b**) and Bayesian consensus (**c,d**) species phylogenies of concatenated single-copy marker genes from PVC representatives with the removal of *Chlamydiae* bacterium 1070360-7 and members of the Parilichlamydiaceae family (n=180 taxa) were inferred with the complete alignment (**a,c**) and with compositionally heterogeneous sites removed (**b,d**). High support for bipartitions is indicated by a black circle, with support measured by non-parametric bootstraps (BP) and the transfer bootstrap expectation (TBE) inferred using IQ-TREE with the LG+C60+F+Γ4-derived PMSF approximation, or posterior probability (P) inferred with Phylobayes using the CAT+GTR+Γ4 model of evolution. Consistent clades are collapsed with their taxonomy indicated and chlamydial

families coloured. Run characteristics and converged chains are indicated for Bayesian phylogenies in a grey box (**c,d**). Scale bars indicate the number of substitutions per site. **e.** The 1% most compositionally heterogeneous sites were removed incrementally starting from the initial alignment. Boxplots show the log range of $\chi 2$ test scores (scale on the left) across taxa from alignments with the corresponding percentage of sites removed. The red line (scale to the right) indicates the number of taxa with significant $\chi 2$ test scores, and hence deviation from patterns of amino acid composition found in other taxa. Center lines in the box-and-whisker plot represent median values, box limits represent upper and lower quartile values, and whiskers represent 1.5 times the interquartile range above the upper quartile and below the lower quartile. Arrows indicate the initial alignment (**a,c**) and the percentage of alignment sites removed for no remaining taxa with significant deviations in composition (**b,d**). Changes in BP support for the monophyly of different groups of interest is shown in the bottom line plots, at different intervals of percentage sites removed. Abbreviations and short forms include: *Chlam* b. (*Chlamydiae* bacterium), G1 (Group 1), G2 (Group 2), and CC-III (Chlamydiae Clade III). See also Figure 1 and Data S4-S6.

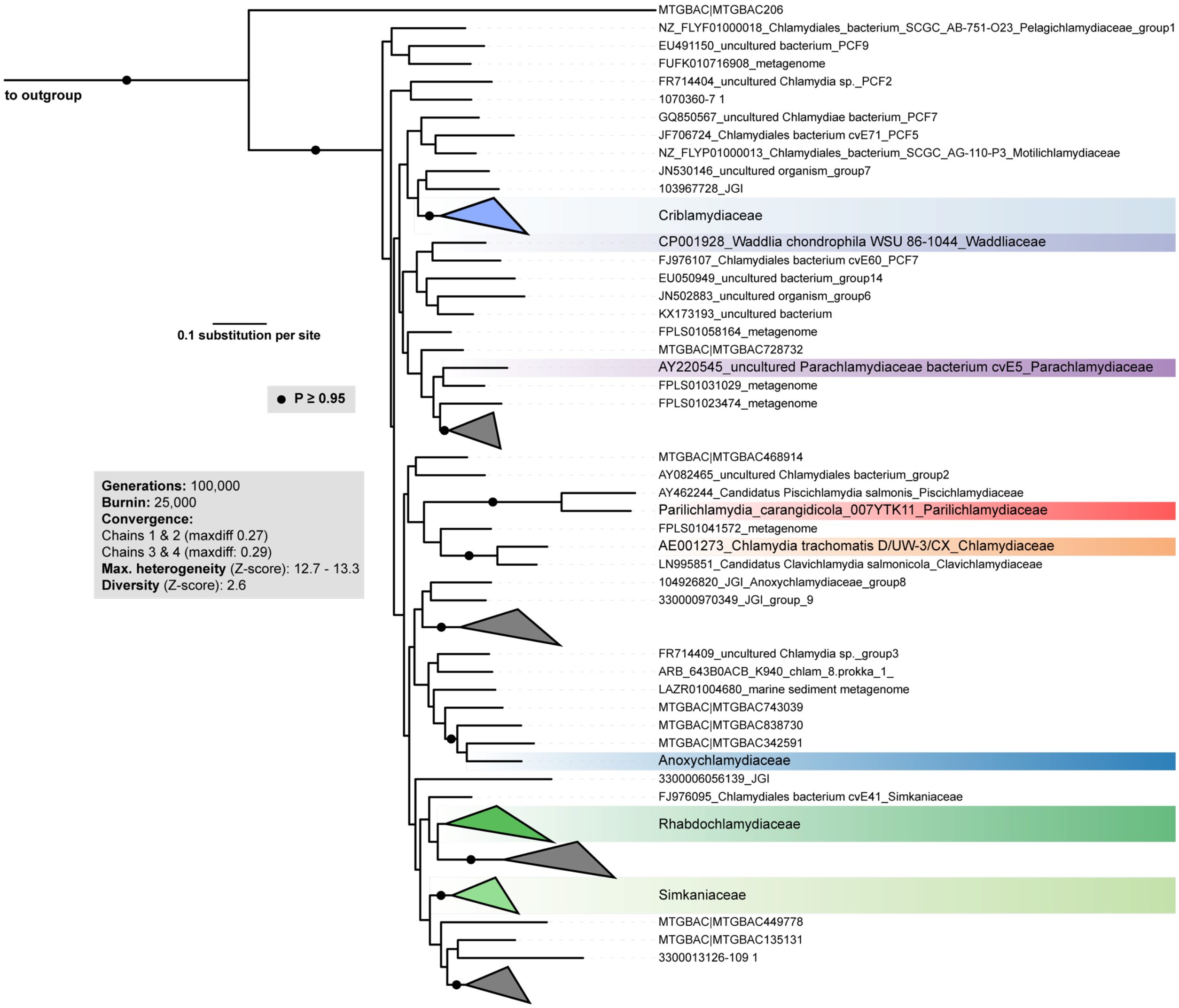

Figure S6. Bayesian 16S rRNA gene phylogenies of family level representatives. Species tree (CAT+GTR+Γ4 model) inferred from an alignment (1,533 aligned positions) of 177 approximately family level representative 16S rRNA gene sequences (> 1,200 nt). Well supported clades (P ≥ 0.95), indicated by filled circles at the nodes, with more than two members were collapsed. Family clades with more than one genome representative are highlighted. See repository files for uncollapsed phylogeny.

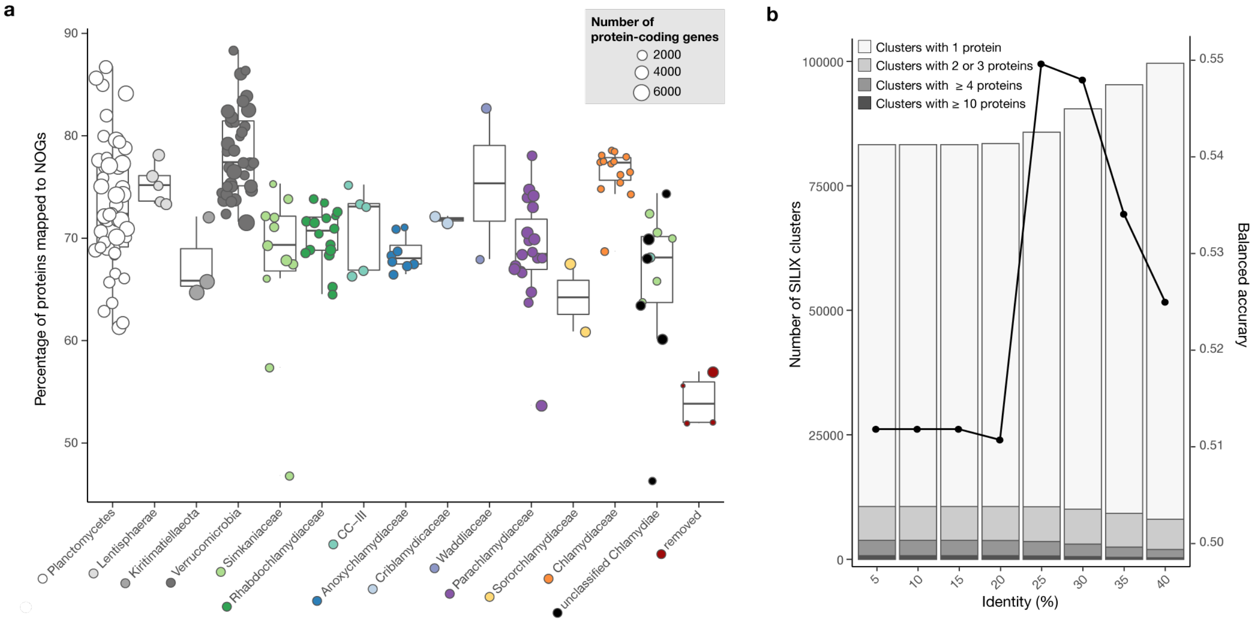

Figure S7. a. Boxplots showing the percentage of each genome mapped to eggNOG NOG gene families from different PVC phyla and chlamydial families (coloured accordingly). Circles represent individual genomes with size indicating the total number of protein-coding genes from that genome. Simkaniaceae-like lineages are coloured in green though they are included under unclassified Chlamydiae. Removed lineages include those excluded from further analyses based on their inconsistent positions in species trees (*i.e.*, members of the Parilichlamydiaceae family and *Chlamydiae* bacterium 1070360-7). Center lines in the box-and-whisker plot represent median values, box limits represent upper and lower quartile values, and whiskers represent 1.5 times the interquartile range above the upper quartile and below the lower quartile. Number of genomes depicted in box-and-whisker plots from left to right n=(47, 5, 3, 34, 11, 16, 5, 8, 2, 2, 19, 2, 14, 12, 4). **b.** Protein-coding genes not mapped to NOGs were *de-novo* clustered into gene families. Barplots show the number of gene families (left axis) generated based on different % identity cutoffs, with the number of member genes indicated by the stacked bars (see legend). Balanced accuracy at different cutoffs is shown by the line plot (right scale) (see Methods). Percentage identity of 25% maximized the balanced accuracy and was thus selected. See also Data S7 for percentage of genes mapped to NOGs and repository files for SILIX clusters across cutoffs and balanced accuracy.

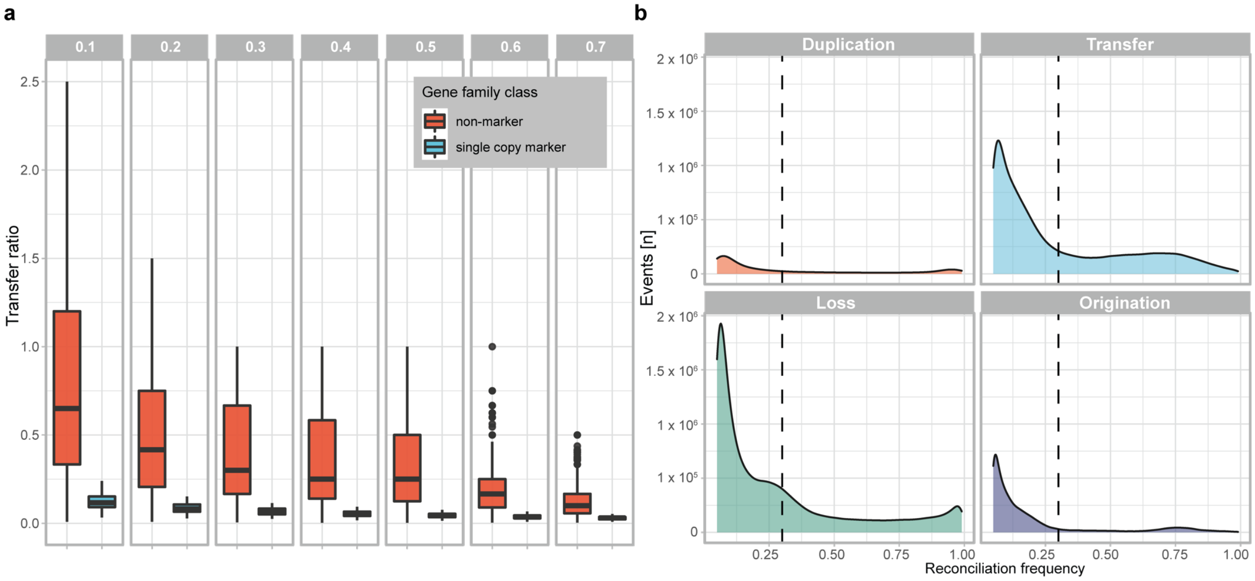

Figure S8. a. Boxplots indicating the ratio of transfers (proportion of horizontal events over all events per gene tree reconciliation) inferred for all non-marker genes with two or more members (red; n=23,330), and single-copy marker genes (blue; n=74; used for species phylogeny inference), at different reconciliation frequency cutoffs for transfer events. At a cutoff of 0.3 the median transfer ratio is likewise 0.3 for non-marker genes, equaling 70% vertical transmission events. Center lines in the box-and-whisker plot represent median values, box limits represent upper and lower quartile values, and whiskers represent 1.5 times the interquartile range above the upper quartile and below the lower quartile. **b**. Density distributions of the number of events inferred to have occurred across reconciliation frequencies for each event type. The selected cutoff of 0.3 is indicated by a dashed line with events to the right of the line used for further analyses. See repository files for raw event data.

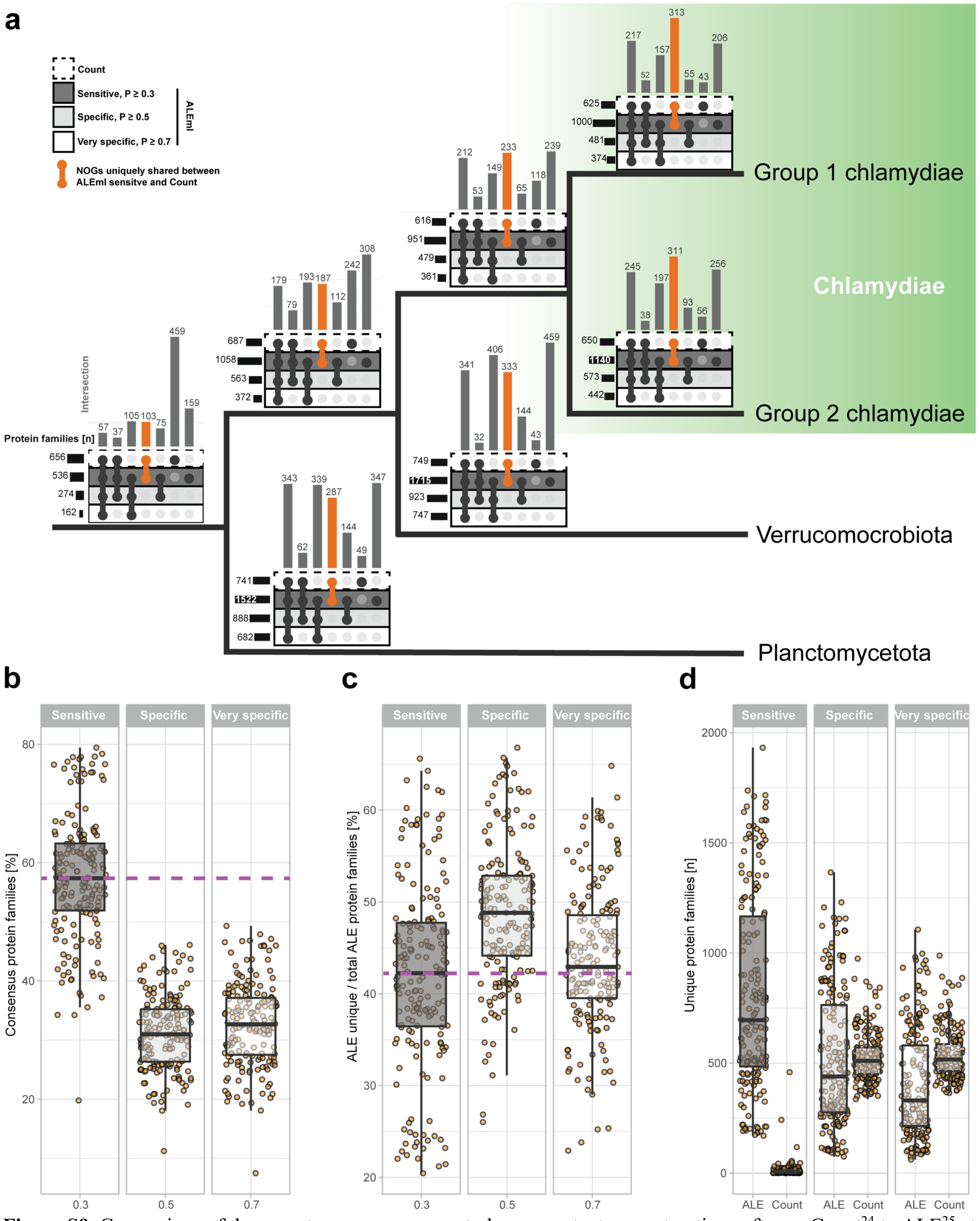

Figure S9. Comparison of the gene-tree unaware ancestral gene content reconstruction software Count[24] to ALE[25] at different frequency cutoffs. For comparison, we defined the ALE frequency cutoffs at P ≤ 0.3 ≤ 0.5 ≤ 0.7 as sensitive, specific, and very specific, respectively. **a.** Plots showing intersections of gene families (y-axis) inferred to be present in early PVC ancestors using different reconstruction methods and mapped to a schematic phylogenetic tree based on Figure 1. The X-axis depicts the total inferred gene families per method and ancestor, respectively. Comparisons (**b-d**) of inferred gene content in PVC ancestors (n=179). **b.** Boxplots depict the percentage of gene families inferred using both Count and the respective ALE cutoffs relative to all inferred gene families per PVC ancestors with both

methods. Purple line indicates the median value of the sensitive cutoff. **c.** Percentage of uniquely inferred gene families in all PVC ancestors with the respective ALE cutoff in comparison to Count. The purple line indicates the median value of the sensitive cutoff. **d.** Total unique gene families inferred with ALE and Count with the respective ALE cutoffs. Center lines in the box-and-whisker plot represent median values, box limits represent upper and lower quartile values, and whiskers represent 1.5 times the interquartile range above the upper quartile and below the lower quartile. See Data S7-S8 and repository files for raw ALE and Count output.

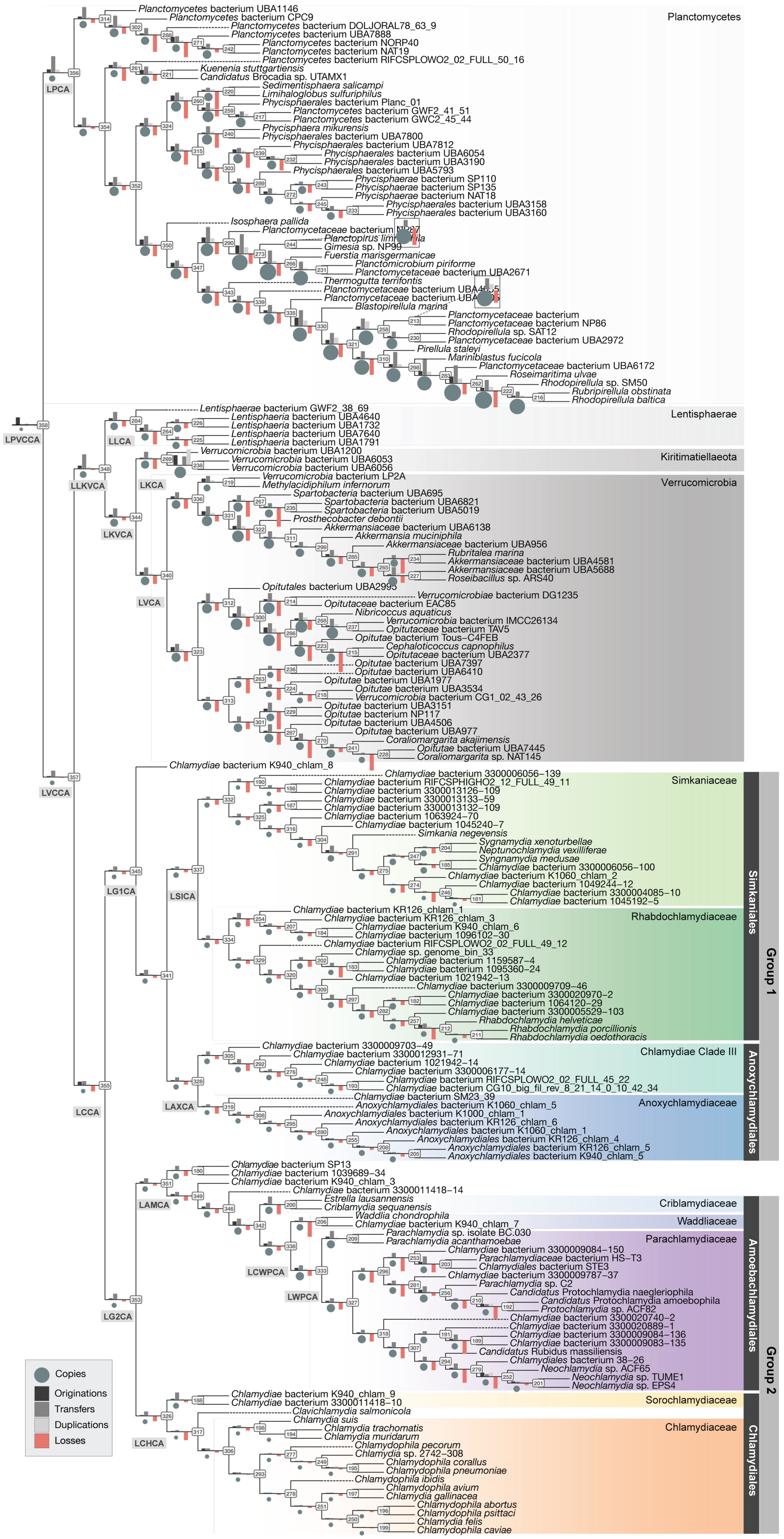

Figure S10. Overview of ancestral events and inferred proteome sizes across all PVC nodes, alongside node numbers. Only gene copy and event numbers with reconciliation frequencies ≥ 0.3 were considered (see Methods). Barplots at

each branch indicate origination, transfer, and duplication events in the positive direction (grey bars; see legend), and loss events in the negative direction (red bars), while circle size represents inferred ancestral proteome size (*i.e.*, number of protein-coding gene copies) for each node to the right. The maximum bar size is 1000, several cases with larger numbers of events are capped at this size (in non-Chlamydiae PVC nodes). Taxonomic groups are indicated and chlamydial families coloured. Key ancestors are indicated and abbreviations can be found in Data S7 alongside event counts for each node.

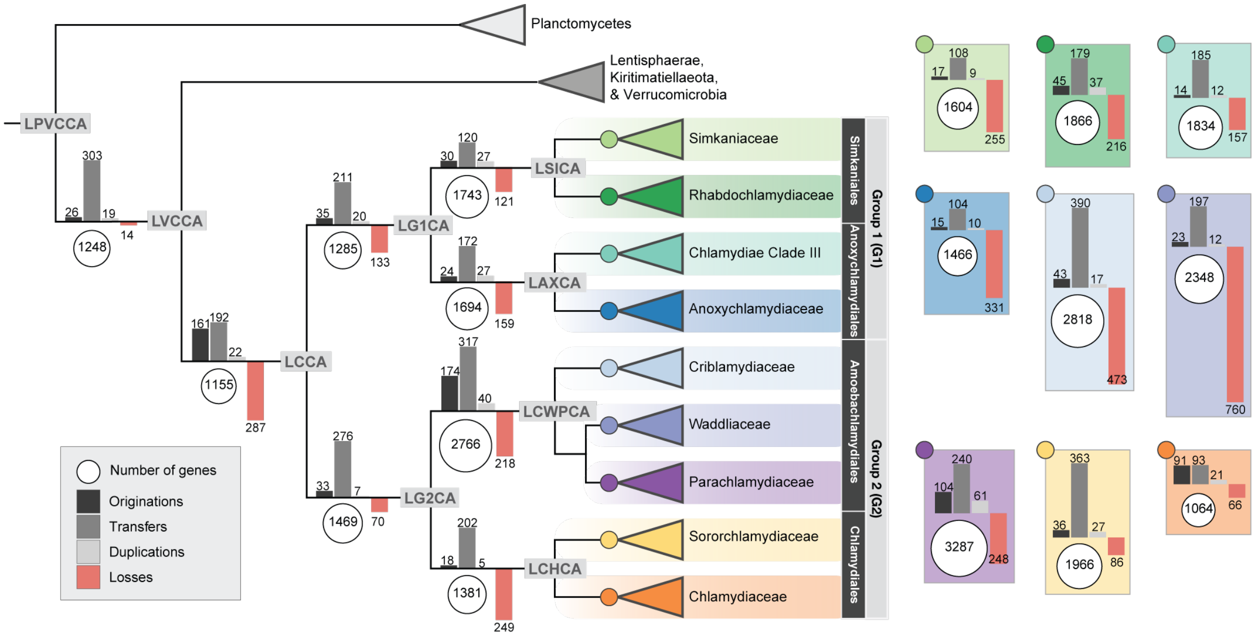

Figure S11. Schematic phylogenetic tree corresponds to the Bayesian consensus tree depicted in Figure 1. Nodes are annotated with proteome size in white circles, while branches are annotated with bars representing the number of originations, transfer, and duplication events in greyscale (see legend). The number of loss events are indicated negatively by a red bar. Only gene copy and event numbers with reconciliation frequencies ≥ 0.3 were considered (see Methods). Terminal nodes represent chlamydial family ancestors, collapsed into triangles, with the corresponding node events shown to the right. Orphan lineages are excluded. Abbreviations in the figure refer to: ancestors of all PVC bacteria (LPVCCA); Chlamydiae, Lentisphaerae, Kiritimatiellaeota, & Verrucomicrobia (LVCCA); Chlamydiae (LCCA); Group 1 chlamydiae (LG1CA); Simkaniales (LSICA); Anoxychlamydiales (LAXCA); Group 2 chlamydiae (LG2CA); Criblamydiaceae, Waddliaceae, & Parachlamydiaceae (LCWPCA); Amoebachlamydiales (LAMCA); Chlamydiales (LCHCA). See Figure S10 for events across all PVC bacteria ancestor nodes and Figure 3 for gene copy and event numbers based on raw reconciliation frequencies.

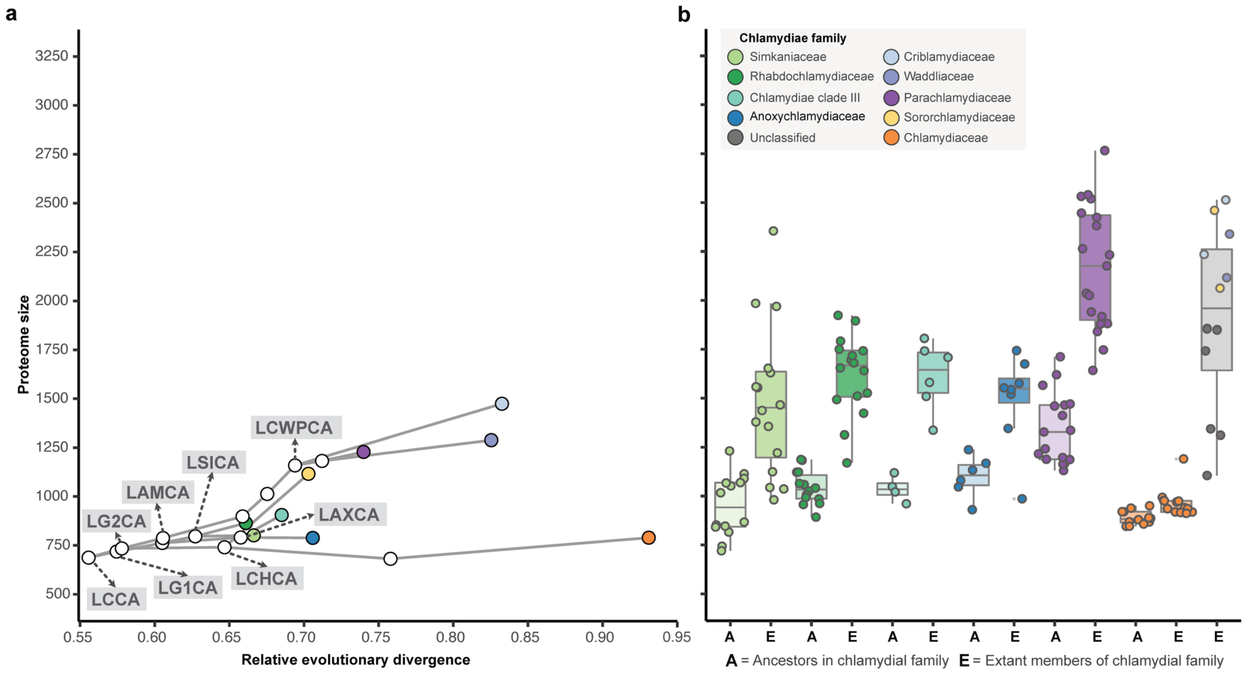

Figure S12. Ancestor proteome size is underestimated when gene trees and extinction probabilities are not taken into account. Proteome size inferred across chlamydial ancestors using a gene-tree unaware method (see Methods), and protein-coding gene copies present in extant chlamydial genomes. **a**. Inferred proteome size of early chlamydial ancestors scaled to the relative evolutionary divergence (RED) metric[21]. The RED metric provides an approximation of relative time from a given common ancestor (LPVCCA, RED=0) to extant taxa (RED=1). **b**. Comparison of proteome size between ancestors and extant members from each chlamydial family. Chlamydiae from families without in-family ancestors are grouped together in the grey boxplot (*i.e.*, Criblamydiaceae, Waddliaceae, Sororchlamydiaceae, and unclassified). Center lines in the box-and-whisker plot represent median values, box limits represent upper and lower quartile values, and whiskers represent 1.5 times the interquartile range above the upper quartile and below the lower quartile. Number of ancestors and extant members per family depicted from left to right: n=(14, 16, 14, 16, 4, 6, 6, 8, 17, 19, 12, 14, 12). Nodes and branches are coloured by family assignment according to the legend. Abbreviations of key ancestors are labeled as in Figure 3 (see Data S7). See also Data S8 and repository.

SUPPLEMENTARY DATA

Data S1. Overview of initial prefiltered PVC bacteria dataset and genome representative selection. Includes genome quality and if genomes were selected for downstream analysis for both non-chlamydial PVC bacteria (a) and Chlamydiae (b), representative genomes after species-level dereplication (c), and subsequent selection of non-Chlamydiae PVC bacteria genus representatives (d).

Data S2. Overview of PVC genome representatives selected for use. Includes taxonomy, source information (*e.g.*, Genbank identifier), species and strain names, genome identifiers used in species phylogenies and for ALE analyses, and genome characteristics.

Data S3. Selection of single-copy marker genes. NOG identifiers and annotations, alongside trimmed alignment length are given for the initial dataset of 116 marker genes. NOGs removed and retained from the marker gene set are indicated after each of the two rounds of tree refinement. The protein sequence identifiers are listed for sequences removed based on tree refinement due to either being duplicates or partial sequences, and those that could represent HGT events, contamination, or distant paralogs. Discordance scores for each of the 79 gene markers that passed tree refinement are given, and those subsequently removed alongside the 74 marker genes selected as the final dataset are indicated.

Data S4. Summary of the stepwise removal of the most compositionally heterogeneous sites from alignments with 184 (a), 183 (b), and 180 (c) taxa in 1% increments. The corresponding alignment length, percentage of the alignment removed, and number of taxa significantly divergent in composition is given for each step, alongside the $\chi 2$ test score for each taxon. Dark green indicates alignments corresponding to trees shown in Figures 1 and S3-S5, while light green indicates alignments where ML phylogenies were also inferred and used to assess the monophyly of different groups in Figures S3-S5.

Data S5. Overview of all species phylogenies inferred, with the number of taxa, percentage of the total alignment pruned, alignment length, model of evolution, inference method and supports, and where the phylogeny can be found, with corresponding page numbers for Data S6.

Data S6. Uncollapsed trees for all species phylogenies inferred including both ML (both PMSF non-parametric bootstrap and TBE supports) and Bayesian trees (all chains, and relevant consensus trees with posterior probabilities). Uncollapsed ML trees for subunits of proton-transporting NADH dehydrogenase (NuoA-N), cytochrome o ubiquinol oxidase (CyoA-D), and proton-driven ATP synthase (AtpA-H).

Data S7. Summary of key ancestor nodes and the corresponding ancestor abbreviation and included taxonomic groups (a) alongside the percentage of genes mapped to NOGs, the number of genes per taxon included in the reconciliation, singletons, and those excluded per genome (b). Overview of the number of events at each node inferred using raw summed events (c) and different cutoffs (0.1 to 0.9, in 0.05 increments; 0.3 selected) for duplications (d), transfers (e), losses (f), originations (g), and copies (*i.e.*, ancestral proteome size) (h). The sum of gains and losses across COG functional categories at a reconciliation frequency of 0.3 (i)

Data S8. Ancestral genome content reconstructions based on the gene-tree unaware method Count. Includes inferred copy number per gene family per node in the species phylogeny, if the inferred copy number was larger than 0.

Data S9. Summary of annotations of gene content of selected PVC ancestors: all annotations (a), LPVCCA (b), LVCCA (c), LVCA (d), LCCA (e), LG1CA (f), LG2CA (g). Includes annotation information from eggNOG, PFAM, TIGRFAM, and Interpro databases per gene family in addition to noted manual curation for important genes per ancestor referred to in Figure 2 and Extended Data Figure 2.

Data S10. An overview of gene annotations presented in Figures 2-3 and Extended Data Figures S5-S6 is also outlined for genes and complexes related to: the electron transport chain (a), the TCA cycle and fermentation (b), and other key genes and pathways of interest (c), alongside inferred copy number (hence presence) across all Chlamydiae ancestors and major PVC ancestors.

Data S11. Taxonomic groups affiliated with chlamydiae in gene trees for inferred originations, and representing the putative HGT donor lineage (taxonomy of ≥ 75% of sequences in sister clade) and visualized in Extended Data Figure 4 (a). Domain, superphylum, and phylum affiliation of each chlamydial gene family origination based on different cutoffs of percentage taxa (75, 90, and 100%) in a supported monophyletic clade sister to chlamydial sequences, and in the clade sister to this group (nested) (b).

SUPPLEMENTARY REFERENCES

1. Taylor-Brown, A. *et al.* Metagenomic analysis of fish-associated Ca. Parilichlamydiaceae reveals striking metabolic similarities to the terrestrial Chlamydiaceae. *Genome Biol. Evol.* **10**, 2587–2595 (2018).
2. Pillonel, T., Bertelli, C. & Greub, G. Environmental metagenomic assemblies reveal seven new highly divergent chlamydial lineages and hallmarks of a conserved intracellular lifestyle. *Front. Microbiol.* **9**, 79 (2018).
3. Dharamshi, J. E. *et al.* Marine sediments illuminate Chlamydiae diversity and evolution. *Curr. Biol.* **30**, 1032–1048.e7 (2020).
4. Köstlbacher, S. *et al.* Pangenomics reveals alternative environmental lifestyles among chlamydiae. *Nat. Commun.* **12**, 4021 (2021).
5. Bergsten, J. A review of long-branch attraction. *Cladistics* **21**, 163–193 (2005).
6. Philippe, H. *et al.* Resolving difficult phylogenetic questions: why more sequences are not enough. *PLoS Biol.* **9**, e1000602 (2011).
7. Lartillot, N. & Philippe, H. A Bayesian mixture model for across-site heterogeneities in the amino-acid replacement process. *Mol. Biol. Evol.* **21**, 1095–1109 (2004).
8. Philippe, H., Delsuc, F., Brinkmann, H. & Lartillot, N. Phylogenomics. *Annual Review of Ecology, Evolution, and Systematics* **36**, 541–562 (2005).
9. Stairs, C. W. *et al.* Chlamydial contribution to anaerobic metabolism during eukaryotic evolution. *Sci Adv* **6**, eabb7258 (2020).
10. Borel, N. & Greub, G. International Committee on Systematics of Prokaryotes (ICSP) Subcommittee on the taxonomy of Chlamydiae. Minutes of the closed meeting, 5 July 2018, Woudschoten, Zeist, The Netherlands. *International Journal of Systematic and Evolutionary Microbiology* **69**, 2606–2608 (2019).
11. Greub, G. & Bavoil, P. International Committee on Systematics of Prokaryotes Subcommittee on the taxonomy of Chlamydiae. Minutes of the closed meeting, 7 September 2016, Oxford, UK. *International Journal of Systematic and Evolutionary Microbiology* **68**, 3683–3684 (2018).
12. Köstlbacher, S., Collingro, A., Halter, T., Domman, D. & Horn, M. Coevolving plasmids

drive gene flow and genome plasticity in host-associated intracellular bacteria. *Curr. Biol.* **31**, 346–357.e3 (2021).
13. Kuo, C., Horn, M. & Stephens, R. S. Order I. Chlamydiales. in *Bergey's Manual of Systematic Bacteriology* (ed. Krieg N, Staley J, Brown D, Hedlund B, Paster B, Ward N, Ludwig W, Whitman W) vol. 4, 2nd ed. 844–845 (Springer, 2011).
14. Kuo, C. & Stephens, R. Family I. Chlamydiaceae. in *Bergey's Manual of Systematic Bacteriology* (ed. Krieg NR, Staley JT, Brown DR, Hedlund BP, Paster BJ, Ward NL, Ludwig W, and Whitman WB) vol. 4, 2nd ed. 845 (Springer, 2011).
15. Horn, M. Family II. 'Candidatus Clavichlamydiaceae'. in *Bergey's Manual of Systematic Bacteriology* (ed. Krieg NR, Staley JT, Brown DR, Hedlund BP, Paster BJ) vol. 4, 2nd ed. 865 (Springer, 2011).
16. Dharamshi, J. E. *et al.* Genomic diversity and biosynthetic capabilities of sponge-associated chlamydiae. *bioRxiv* (2021) doi:10.1101/2021.12.21.473556.
17. Thomas, V., Casson, N. & Greub, G. *Criblamydia sequanensis*, a new intracellular Chlamydiales isolated from Seine river water using amoebal co-culture. *Environ. Microbiol.* **8**, 2125–2135 (2006).
18. Rurangirwa, F. R., Dilbeck, P. M., Crawford, T. B., McGuire, T. C. & McElwain, T. F. Analysis of the 16S rRNA gene of micro-organism WSU 86-1044 from an aborted bovine foetus reveals that it is a member of the order Chlamydiales: proposal of Waddliaceae fam. nov., *Waddlia chondrophila* gen. nov., sp. nov. *Int. J. Syst. Bacteriol.* **49 Pt 2**, 577–581 (1999).
19. Everett, K. D., Bush, R. M. & Andersen, A. A. Emended description of the order Chlamydiales, proposal of Parachlamydiaceae fam. nov. and Simkaniaceae fam. nov., each containing one monotypic genus, revised taxonomy of the family Chlamydiaceae, including a new genus and five new species, and standards for the identification of organisms. *Int. J. Syst. Bacteriol.* **49 Pt 2**, 415–440 (1999).
20. Horn, M. Family VI. Rhabdochlamydiaceae fam. nov. in *Bergey's Manual of Systematic Bacteriology* (ed. Krieg NR, Staley JT, Brown DR, Hedlund BP, Paster BJ, Ward NL, Ludwig W, and Whitman WB) vol. 4, 2nd ed. 873 (Springer, 2011).
21. Parks, D. H. *et al.* A standardized bacterial taxonomy based on genome phylogeny substantially revises the tree of life. *Nat. Biotechnol.* **36**, 996–1004 (2018).
22. Price, M. N., Dehal, P. S. & Arkin, A. P. FastTree 2--approximately maximum-likelihood trees for large alignments. *PLoS One* **5**, e9490 (2010).
23. Williams, K. P. *et al.* Phylogeny of gammaproteobacteria. *J. Bacteriol.* **192**, 2305–2314 (2010).
24. Csurös, M. Count: evolutionary analysis of phylogenetic profiles with parsimony and likelihood. *Bioinformatics* **26**, 1910–1912 (2010).
25. Szöllősi, G. J., Davín, A. A., Tannier, E., Daubin, V. & Boussau, B. Genome-scale phylogenetic analysis finds extensive gene transfer among fungi. *Philos. Trans. R. Soc. Lond. B Biol. Sci.* **370**, 20140335 (2015).
