# Supplementary material for: Gene gain facilitated endosymbiotic evolution of Chlamydiae
Source: Nat Microbiol. 2023 Jan 5;8(1):40–54. doi: 10.1038/s41564-022-01284-9 (PMC9816063; doi:10.1038/s41564-022-01284-9)
Supplement: Supplementary file 2 — Reporting Summary [file 41564_2022_1284_MOESM2_ESM.pdf]

nature portfolio

Corresponding author(s): Matthias Horn & Thijs Ettema

Last updated by author(s): October 31, 2022

Reporting Summary

Nature Portfolio wishes to improve the reproducibility of the work that we publish. This form provides structure for consistency and transparency in reporting. For further information on Nature Portfolio policies, see our Editorial Policies and the Editorial Policy Checklist.

Statistics

For all statistical analyses, confirm that the following items are present in the figure legend, table legend, main text, or Methods section.

n/a	Confirmed	
☐	☒	The exact sample size (*n*) for each experimental group/condition, given as a discrete number and unit of measurement
☒	☐	A statement on whether measurements were taken from distinct samples or whether the same sample was measured repeatedly
☐	☒	The statistical test(s) used AND whether they are one- or two-sided<br>*Only common tests should be described solely by name; describe more complex techniques in the Methods section.*
☒	☐	A description of all covariates tested
☐	☒	A description of any assumptions or corrections, such as tests of normality and adjustment for multiple comparisons
☐	☒	A full description of the statistical parameters including central tendency (e.g. means) or other basic estimates (e.g. regression coefficient) AND variation (e.g. standard deviation) or associated estimates of uncertainty (e.g. confidence intervals)
☐	☒	For null hypothesis testing, the test statistic (e.g. *F*, *t*, *r*) with confidence intervals, effect sizes, degrees of freedom and *P* value noted<br>*Give P values as exact values whenever suitable.*
☒	☐	For Bayesian analysis, information on the choice of priors and Markov chain Monte Carlo settings
☒	☐	For hierarchical and complex designs, identification of the appropriate level for tests and full reporting of outcomes
☒	☐	Estimates of effect sizes (e.g. Cohen's *d*, Pearson's *r*), indicating how they were calculated

Our web collection on statistics for biologists contains articles on many of the points above.

Software and code

Policy information about availability of computer code

Data collection	dRep v1.4.3; GTDB v86; miComplete 1.1.1;
Data analysis	emapper v1.0.1; MAFFT L-INS-i v7.427; BMGE vl.12; IQ-TREE v1.6.10, 1.6.11, 1.6.12, 2.0; PhyloBayes-MPI v1.7b; USEARCH v11.0.667; trimAl v1.4.1; PhyloBayes v4.1c; DIAMOND v0.9.21; SiliX v1.2.9; interproscan v5.36-75.0; Count v10.04; ALE v0.449; Cytoscape v3.7.0; Snakemake v5.19.3; MAFFT v7.471; FastTree 2 v2.1.11; ETE3 Toolkit v3.1.2; PhyloRank v0.1.10; R v3.6.2; ggplot2 v3.3.3; ggtree v2.5.0.991; treeio v1.10.0; Figtree v1.4.4; CD-HIT v4.8.1; trimAl v1.4.rev15<br><br>Custom code:<br>https://github.com/jennahd/HGT_trees,<br>https://github.com/maxemil/ALE-pipeline

For manuscripts utilizing custom algorithms or software that are central to the research but not yet described in published literature, software must be made available to editors and reviewers. We strongly encourage code deposition in a community repository (e.g. GitHub). See the Nature Portfolio guidelines for submitting code & software for further information.

Data

Policy information about availability of data

All manuscripts must include a data availability statement. This statement should provide the following information, where applicable:

- Accession codes, unique identifiers, or web links for publicly available datasets
- A description of any restrictions on data availability
- For clinical datasets or third party data, please ensure that the statement adheres to our policy

Genome data was obtained either from NCBI Genbank (https://www.ncbi.nlm.nih.gov/nucleotide/), the JGI portal (https://portal.nersc.gov/GEM/) or a zenodo repository (https://doi.org/l0.5281/zenodo.4318714). Small subunit rRNA gene data used in this study are available via the SILVA database (https://www.arb-silva.de/). Genbank accessions and database links for genomes used in the ancestral state reconstruction are provided in Data S2. Additional raw data files are hosted on the online repository figshare (https://doi.org/10.6084/m9.figshare.17033417). These include sequences, alignments, trimmed alignments, and trees for single-copy marker genes used for species phylogenies (both those selected and not selected), the 16S rRNA gene, and concatenated alignments and trees for all three species datasets (of 184, 183, and 180 taxa). Both NOG and de novo gene families used for the ancestral state reconstruction are also provided alongside alignments, trimmed alignments, trees, and bootstrap trees (ufboot) provided to ALE. The raw ALE results with all events are also included, alongside gene annotations together with events, and events for each gene family mapped to the species tree. Protein sequence datasets, alignments and trees inferred as part of the analysis to determine HGT donors for chlamydiae gene originations are provided. In addition, pdfs of metabolic reconstructions of LVCCA, LG1CA, and LG2CA can be found in the repository files.

Human research participants

Policy information about studies involving human research participants and Sex and Gender in Research.

Reporting on sex and gender	NA
Population characteristics	NA
Recruitment	NA
Ethics oversight	NA

Note that full information on the approval of the study protocol must also be provided in the manuscript.

Field-specific reporting

Please select the one below that is the best fit for your research. If you are not sure, read the appropriate sections before making your selection.

☐ Life sciences ☐ Behavioural & social sciences ☒ Ecological, evolutionary & environmental sciences

For a reference copy of the document with all sections, see nature.com/documents/nr-reporting-summary-flat.pdf

Ecological, evolutionary & environmental sciences study design

All studies must disclose on these points even when the disclosure is negative.

Study description	Phylogenomic analyses of Chlamydiae and other Planctomycetes-Verrucomicrobia-Chlamydiae (PVC) superphylum bacteria. Gene-tree<br>species tree reconciliation and ancestral reconstruction of the last common ancestors of PVC bacteria with a focus on Chlamydiae evolution. Analysis of the origin of gained gene content in the Chlamydiae phylum for key ancestors.
Research sample	Available genomes of cultured and uncultured PVC bacteria with a focus on recently published Chlamydiae draft genomes.
Sampling strategy	We selected genomes based on their phylogenetic affiliation with PVC bacteria. Representatives where then selected based on the highest genome quality score per taxonomic unit: approximately species for Chlamydiae, and genus (sensu GTDB) level for other PVC bacteria.
Data collection	N/A, as primary data collection (i.e., DNA sequencing, genome assembly, and quality control) was performed by other parties (sequence contributors to JGI and NCBI). PVC genomes were downloaded based on their taxonomy from GTDB, additional chlamydiae from more recent studies were additionally downloaded from the JGI.
Timing and spatial scale	Genomes were downloaded all at once on April 3rd, 2019.
Data exclusions	We excluded Chlamydiae genomes with a miComplete specific marker gene set estimated completeness smaller than 0.9 and a redundancy larger than 1.02. Quality of other PVC genomes was based on GTDB provided CheckM quality scores, we excluded genomes with an estimated completeness smaller than 90% and a contamination larger than 2%.

Reproducibility	All results of this study can be reproduced given the same original source data and the methods provided in this manuscript.
Randomization	N/A because randomization was not required for the purposes of this study, as we based all ancestral reconstructions on the complete set of highest quality available PVC genomes to infer ancestral state reconstruction.
Blinding	N/A because blinding was not required for the purposes of this study, as the taxonomic and evolutionary context was of great importance for interpretation of the findings.

Did the study involve field work? ☐ Yes ☒ No

Reporting for specific materials, systems and methods

We require information from authors about some types of materials, experimental systems and methods used in many studies. Here, indicate whether each material, system or method listed is relevant to your study. If you are not sure if a list item applies to your research, read the appropriate section before selecting a response.

Materials & experimental systems

n/a	Involved in the study	
☒	☐	Antibodies
☒	☐	Eukaryotic cell lines
☒	☐	Palaeontology and archaeology
☒	☐	Animals and other organisms
☒	☐	Clinical data
☒	☐	Dual use research of concern

Methods

n/a	Involved in the study	
☒	☐	ChIP-seq
☒	☐	Flow cytometry
☒	☐	MRI-based neuroimaging
