# Supplementary material for: Gene gain facilitated endosymbiotic evolution of Chlamydiae
Source: Nat Microbiol. 2023 Jan 5;8(1):40–54. doi: 10.1038/s41564-022-01284-9 (PMC9816063; doi:10.1038/s41564-022-01284-9)

Supplementary Data S6 – Phylogenetic trees

PVC BACTERIA SPECIES PHYLOGENIES (SEE DATA S5) **3-62**
180 TAXA DATASET **3-23**
ML (PMSF) – 0% pruned *3*
ML (TBE) – 0% pruned *4*
Bayesian (Chain 1) – 0% pruned *5*
Bayesian (Chain 2) – 0% pruned *6*
Bayesian (Chain 3) – 0% pruned *7*
Bayesian (Chain 4) – 0% pruned *8*
Bayesian (Consensus of chains 1, 2, 3, & 4) – 0% pruned *9*
ML (PMSF) – 10% pruned *10*
ML (PMSF) – 20% pruned *11*
ML (PMSF) – 30% pruned *12*
ML (PMSF) – 40% pruned *13*
ML (PMSF) – 50% pruned *14*
ML (PMSF) – 51% pruned *15*
ML (TBE) – 51% pruned *16*
Bayesian (Chain 1) – 51% pruned *17*
Bayesian (Chain 2) – 51% pruned *18*
Bayesian (Chain 3) – 51% pruned *19*
Bayesian (Chain 4) – 51% pruned *20*
Bayesian (Consensus of chains 1, 2, 3, & 4) – 51% pruned *21*
Bayesian (Consensus of chains 1 & 3) – 51% pruned *22*
Bayesian (Consensus of chains 2 & 4) – 51% pruned *23*
183 TAXA DATASET **24-43**
ML (PMSF) – 0% pruned *24*
ML (TBE) – 0% pruned *25*
Bayesian (Chain 1) – 0% pruned *26*
Bayesian (Chain 2) – 0% pruned *27*
Bayesian (Chain 3) – 0% pruned *28*
Bayesian (Chain 4) – 0% pruned *29*
Bayesian (Consensus of chains 1, 2, 3, & 4) – 0% pruned *30*
ML (PMSF) – 10% pruned *31*
ML (PMSF) – 20% pruned *32*
ML (PMSF) – 30% pruned *33*
ML (PMSF) – 40% pruned *34*
ML (PMSF) – 48% pruned *35*
ML (TBE) – 48% pruned *36*
Bayesian (Chain 1) – 48% pruned *37*
Bayesian (Chain 2) – 48% pruned *38*
Bayesian (Chain 3) – 48% pruned *39*
Bayesian (Chain 4) – 48% pruned *40*
Bayesian (Consensus of chains 1, 2, 3, & 4) – 48% pruned *41*
Bayesian (Consensus of chains 2 & 4) – 48% pruned *42*
ML (PMSF) – 50% pruned *43*
184 Taxa Dataset **44-62**
ML (PMSF) – 0% pruned *44*
ML (TBE) – 0% pruned *45*
Bayesian (Chain 1) – 0% pruned *46*
Bayesian (Chain 2) – 0% pruned *47*
Bayesian (Chain 3) – 0% pruned *48*
Bayesian (Chain 4) – 0% pruned *49*
Bayesian (Consensus of chains 1, 2, 3, & 4) – 0% pruned *50*

ML (PMSF) – 10% pruned 51
ML (PMSF) – 20% pruned 52
ML (PMSF) – 30% pruned 53
ML (PMSF) – 40% pruned 54
ML (PMSF) – 50% pruned 55
ML (PMSF) – 51% pruned 56
ML (TBE) – 51% pruned 57
Bayesian (Chain 1) – 51% pruned 58
Bayesian (Chain 2) – 51% pruned 59
Bayesian (Chain 3) – 51% pruned 60
Bayesian (Chain 4) – 51% pruned 61
Bayesian (Consensus of chains 1, 2, 3, & 4) – 51% pruned 62

ML SINGLE PROTEIN PHYLOGENIES 63-87

PROTON-DRIVEN ATP SYNTHASE 63-69
AtpA (COG0056*) 63
AtpB (COG0356) 64
AtpC (11YZG) 65
AtpD (COG0055) 66
AtpE (0ZXI6) 67
AtpF (COG0711) 68
AtpH (COG0712) 69
CYTOCHROME *O* UBIQUINOL OXIDASE 70-73
CyoA (COG1622) 70
CyoB (COG0843) 71
CyoC (COG1845) 72
CyoD (COG3125) 73
SODIUM TRANSPORTING NADH DEHDYROGENASE 74-87
NuoA (COG0838) 74
NuoB (COG0377) 75
NuoC (COG0852) 76
NuoD (COG0649) 77
NuoE (COG1905*) 78
NuoF (COG1894*) 79
NuoG (COG3383) 80
NuoH (COG1005) 81
NuoI (COG1143) 82
NuoJ (COG0839) 83
NuoK (COG0713) 84
NuoL (COG1009*) 85
NuoM (COG1008) 86
NuoN (COG1007) 87

*NOG includes additional paralogous chlamydial sequences that form distinct clusters. Only the tree encoding the functional paralog of interest is shown, which in all cases here is the largest cluster of chlamydial sequences. Trees of other NOG clusters are in the repository.

**Based on Pfam domains, chlamydial sequences mapped to the NOG including AtpG (COG0224) are a different paralog.

180taxa_0perc_pruned_original

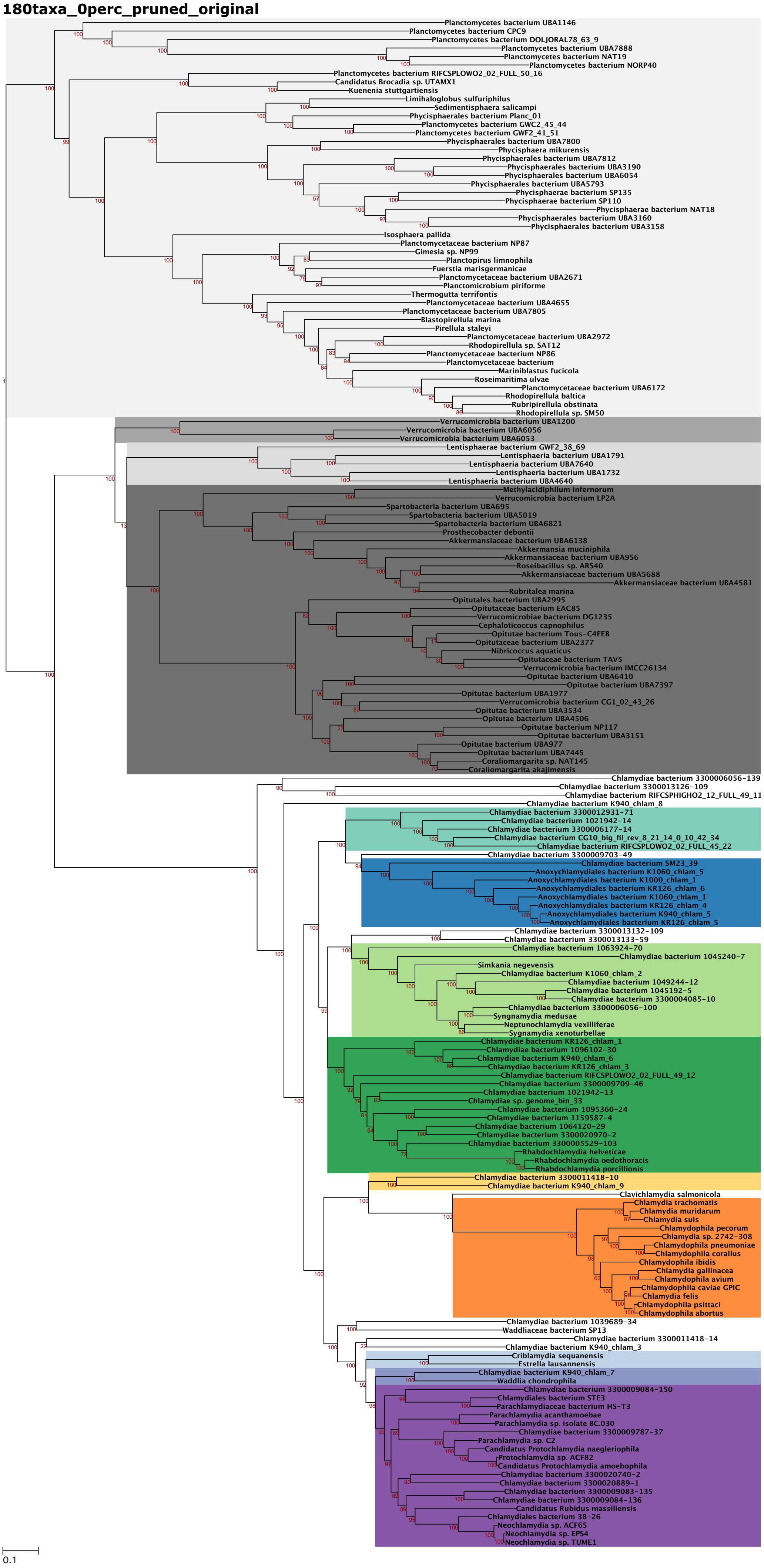

180taxa_0perc_pruned_original

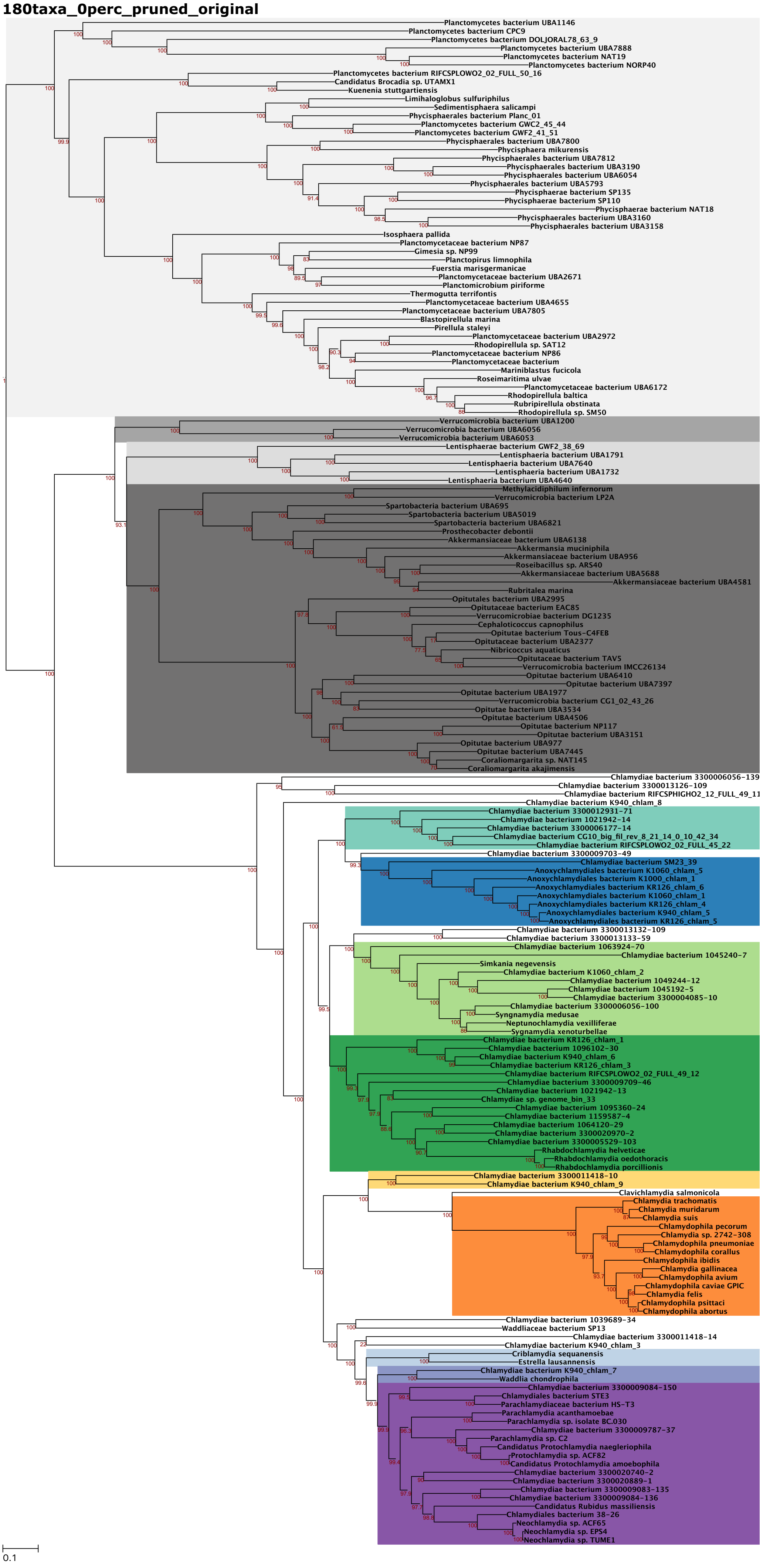

chain1

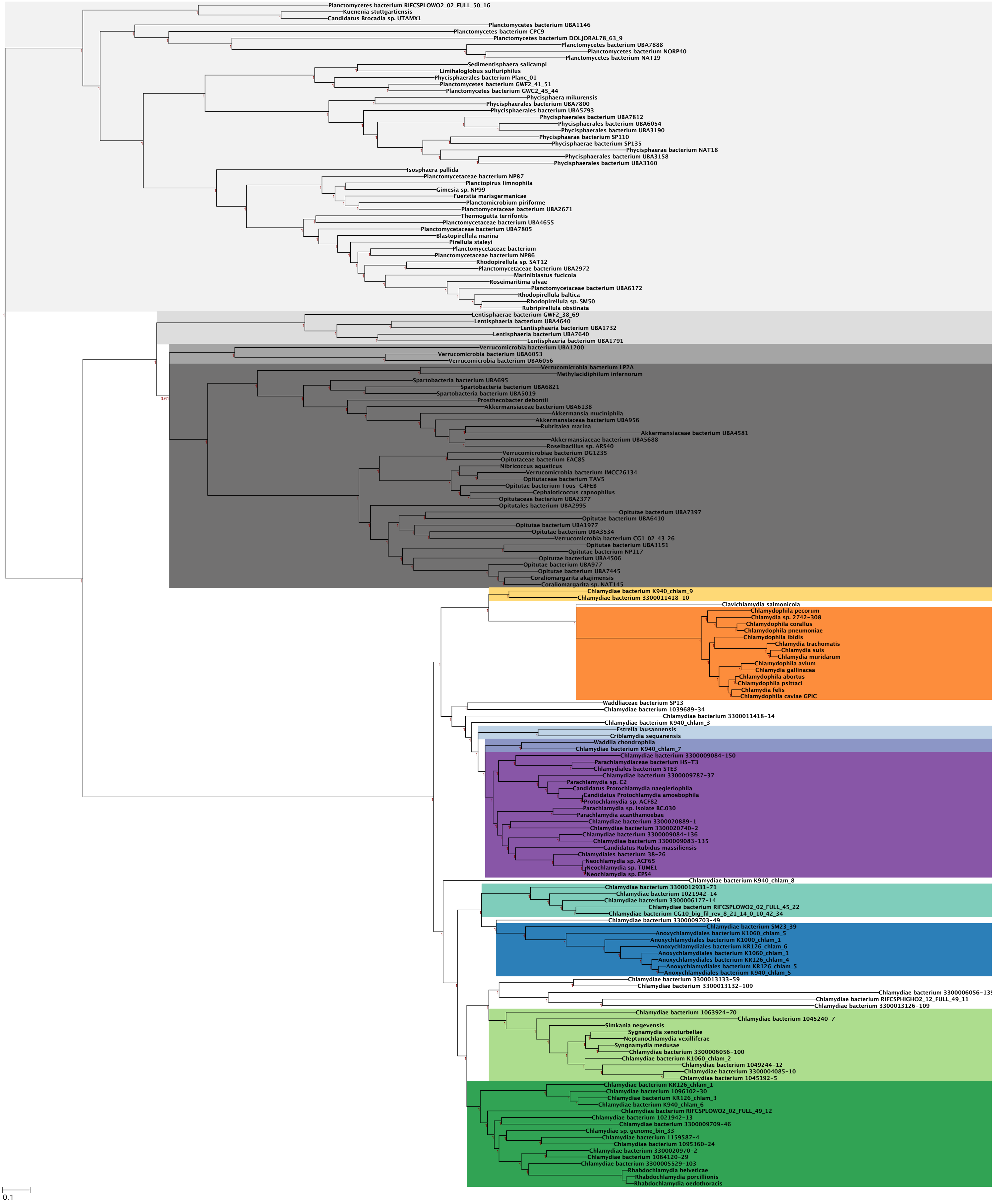

Planctomycetes bacterium RIFCSPLOWO2_02_FULL_50_16
Kuenenia stuttgartiensis
Candidatus Brocadia sp. UTAMX1
Planctomycetes bacterium UBA1146
Planctomycetes bacterium CPC9
Planctomycetes bacterium DOLJORAL78_63_9
Planctomycetes bacterium UBA7888
Planctomycetes bacterium NORP40
Planctomycetes bacterium NAT19
Sedimentisphaera salicampi
Limihaloglobus sulfuriphilus
Phycisphaerales bacterium Planc_01
Planctomycetes bacterium GWF2_41_51
Planctomycetes bacterium GWC2_45_44
Phycisphaera mikurensis
Phycisphaerales bacterium UBA7800
Phycisphaerales bacterium UBA5793
Phycisphaerales bacterium UBA7812
Phycisphaerales bacterium UBA6054
Phycisphaerales bacterium UBA3190
Phycisphaerae bacterium SP110
Phycisphaerae bacterium SP135
Phycisphaerae bacterium NAT18
Phycisphaerales bacterium UBA3158
Phycisphaerales bacterium UBA3160
Isosphaera pallida
Planctomycetaceae bacterium NP87
Planctopirus limnophila
Gimesia sp. NP99
Fuerstia marisgermanicae
Planctomicrobium piriforme
Planctomycetaceae bacterium UBA2671
Thermogutta terrifontis
Planctomycetaceae bacterium UBA4655
Planctomycetaceae bacterium UBA7805
Blastopirellula marina
Pirellula staleyi
Planctomycetaceae bacterium
Planctomycetaceae bacterium NP86
Rhodopirellula sp. SAT12
Planctomycetaceae bacterium UBA2972
Mariniblastus fucicola
Roseimaritima ulvae
Planctomycetaceae bacterium UBA6172
Rhodopirellula baltica
Rhodopirellula sp. SM50
Rubripirellula obstinata
Lentisphaerae bacterium GWF2_38_69
Lentisphaeria bacterium UBA4640
Lentisphaeria bacterium UBA1732
Lentisphaeria bacterium UBA7640
Lentisphaeria bacterium UBA1791
Verrucomicrobia bacterium UBA1200
Verrucomicrobia bacterium UBA6053
Verrucomicrobia bacterium UBA6056
Verrucomicrobia bacterium LP2A
Methylacidiphilum infernorum
Spartobacteria bacterium UBA695
Spartobacteria bacterium UBA6821
Spartobacteria bacterium UBA5019
Prosthecobacter debontii
Akkermansiaceae bacterium UBA6138
Akkermansia muciniphila
Akkermansiaceae bacterium UBA956
Rubritalea marina
Akkermansiaceae bacterium UBA4581
Akkermansiaceae bacterium UBA5688
Roseibacillus sp. ARS40
Verrucomicrobiae bacterium DG1235
Opitutaceae bacterium EAC85
Nibricoccus aquaticus
Verrucomicrobia bacterium IMCC26134
Opitutaceae bacterium TAV5
Opitutae bacterium Tous-C4FEB
Cephaloticoccus capnophilus
Opitutaceae bacterium UBA2377
Opitutales bacterium UBA2995
Opitutae bacterium UBA7397
Opitutae bacterium UBA6410
Opitutae bacterium UBA1977
Opitutae bacterium UBA3534
Verrucomicrobia bacterium CG1_02_43_26
Opitutae bacterium UBA3151
Opitutae bacterium NP117
Opitutae bacterium UBA4506
Opitutae bacterium UBA977
Opitutae bacterium UBA7445
Coraliomargarita akajimensis
Coraliomargarita sp. NAT145
Chlamydiae bacterium K940_chlam_9
Chlamydiae bacterium 3300011418-10
Clavichlamydia salmonicola
Chlamydophila pecorum
Chlamydia sp. 2742-308
Chlamydophila corallus
Chlamydophila pneumoniae
Chlamydophila ibidis
Chlamydia trachomatis
Chlamydia suis
Chlamydia muridarum
Chlamydophila avium
Chlamydia gallinacea
Chlamydophila abortus
Chlamydophila psittaci
Chlamydia felis
Chlamydophila caviae GPIC
Waddliaceae bacterium SP13
Chlamydiae bacterium 1039689-34
Chlamydiae bacterium 3300011418-14
Chlamydiae bacterium K940_chlam_3
Estrella lausannensis
Criblamydia sequanensis
Waddlia chondrophila
Chlamydiae bacterium K940_chlam_7
Chlamydiae bacterium 3300009084-150
Parachlamydiaceae bacterium HS-T3
Chlamydiales bacterium STE3
Chlamydiae bacterium 3300009787-37
Parachlamydia sp. C2
Candidatus Protochlamydia naegleriophila
Candidatus Protochlamydia amoebophila
Protochlamydia sp. ACF82
Parachlamydia sp. isolate BC.030
Parachlamydia acanthamoebae
Chlamydiae bacterium 3300020889-1
Chlamydiae bacterium 3300020740-2
Chlamydiae bacterium 3300009084-136
Chlamydiae bacterium 3300009083-135
Candidatus Rubidus massiliensis
Chlamydiales bacterium 38-26
Neochlamydia sp. ACF65
Neochlamydia sp. TUME1
Neochlamydia sp. EPS4
Chlamydiae bacterium K940_chlam_8
Chlamydiae bacterium 3300012931-71
Chlamydiae bacterium 1021942-14
Chlamydiae bacterium 3300006177-14
Chlamydiae bacterium RIFCSPLOWO2_02_FULL_45_22
Chlamydiae bacterium CG10_big_fil_rev_8_21_14_0_10_42_34
Chlamydiae bacterium 3300009703-49
Chlamydiae bacterium SM23_39
Anoxychlamydiales bacterium K1060_chlam_5
Anoxychlamydiales bacterium K1000_chlam_1
Anoxychlamydiales bacterium KR126_chlam_6
Anoxychlamydiales bacterium K1060_chlam_1
Anoxychlamydiales bacterium KR126_chlam_4
Anoxychlamydiales bacterium KR126_chlam_5
Anoxychlamydiales bacterium K940_chlam_5
Chlamydiae bacterium 3300013133-59
Chlamydiae bacterium 3300013132-109
Chlamydiae bacterium 3300006056-139
Chlamydiae bacterium RIFCSPHIGHO2_12_FULL_49_11
Chlamydiae bacterium 3300013126-109
Chlamydiae bacterium 1063924-70
Chlamydiae bacterium 1045240-7
Simkania negevensis
Sygnamydia xenoturbellae
Neptunochlamydia vexilliferae
Syngnamydia medusae
Chlamydiae bacterium 3300006056-100
Chlamydiae bacterium K1060_chlam_2
Chlamydiae bacterium 1049244-12
Chlamydiae bacterium 3300004085-10
Chlamydiae bacterium 1045192-5
Chlamydiae bacterium KR126_chlam_1
Chlamydiae bacterium 1096102-30
Chlamydiae bacterium KR126_chlam_3
Chlamydiae bacterium K940_chlam_6
Chlamydiae bacterium RIFCSPLOWO2_02_FULL_49_12
Chlamydiae bacterium 1021942-13
Chlamydiae bacterium 3300009709-46
Chlamydiae sp. genome_bin_33
Chlamydiae bacterium 1159587-4
Chlamydiae bacterium 1095360-24
Chlamydiae bacterium 3300020970-2
Chlamydiae bacterium 1064120-29
Chlamydiae bacterium 3300005529-103
Rhabdochlamydia helveticae
Rhabdochlamydia porcellionis
Rhabdochlamydia oedothoracis
0.1

chain2

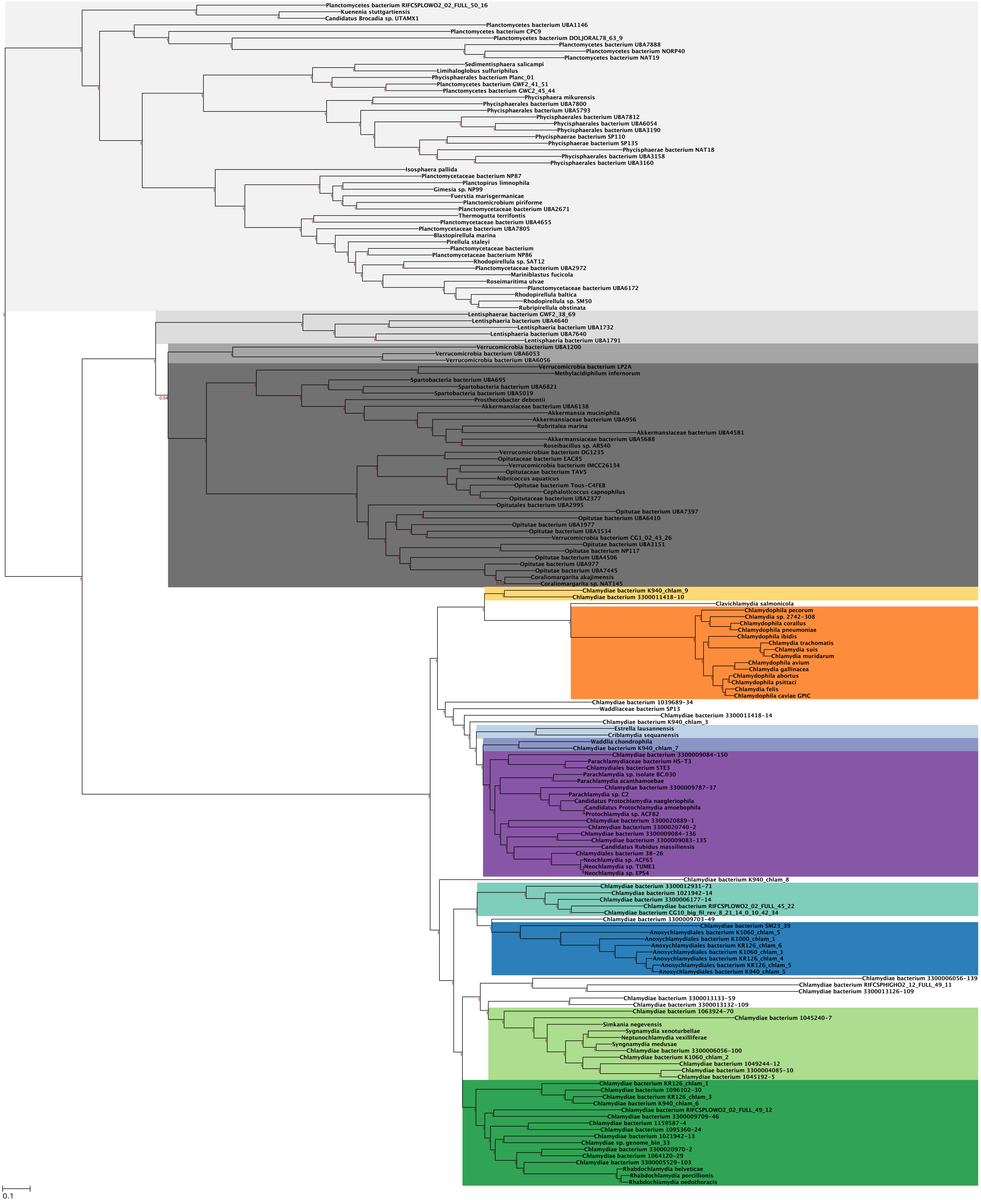
Planctomycetes bacterium RIFCSPLOWO2_02_FULL_50_16
Kuenenia stuttgartiensis
Candidatus Brocadia sp. UTAMX1
Planctomycetes bacterium UBA1146
Planctomycetes bacterium CPC9
Planctomycetes bacterium DOLJORAL78_63_9
Planctomycetes bacterium UBA7888
Planctomycetes bacterium NORP40
Planctomycetes bacterium NAT19
Sedimentisphaera salicampi
Limihaloglobus sulfuriphilus
Phycisphaerales bacterium Planc_01
Planctomycetes bacterium GWF2_41_51
Planctomycetes bacterium GWC2_45_44
Phycisphaera mikurensis
Phycisphaerales bacterium UBA7800
Phycisphaerales bacterium UBA5793
Phycisphaerales bacterium UBA7812
Phycisphaerales bacterium UBA6054
Phycisphaerales bacterium UBA3190
Phycisphaerae bacterium SP110
Phycisphaerae bacterium SP135
Phycisphaerae bacterium NAT18
Phycisphaerales bacterium UBA3158
Phycisphaerales bacterium UBA3160
Isosphaera pallida
Planctomycetaceae bacterium NP87
Planctopirus limnophila
Gimesia sp. NP99
Fuerstia marisgermanicae
Planctomicrobium piriforme
Planctomycetaceae bacterium UBA2671
Thermogutta terrifontis
Planctomycetaceae bacterium UBA4655
Planctomycetaceae bacterium UBA7805
Blastopirellula marina
Pirellula staleyi
Planctomycetaceae bacterium
Planctomycetaceae bacterium NP86
Rhodopirellula sp. SAT12
Planctomycetaceae bacterium UBA2972
Mariniblastus fucicola
Roseimaritima ulvae
Planctomycetaceae bacterium UBA6172
Rhodopirellula baltica
Rhodopirellula sp. SM50
Rubripirellula obstinata
Lentisphaerae bacterium GWF2_38_69
Lentisphaeria bacterium UBA4640
Lentisphaeria bacterium UBA1732
Lentisphaeria bacterium UBA7640
Lentisphaeria bacterium UBA1791
Verrucomicrobia bacterium UBA1200
Verrucomicrobia bacterium UBA6053
Verrucomicrobia bacterium UBA6056
Verrucomicrobia bacterium LP2A
Methylacidiphilum infernorum
Spartobacteria bacterium UBA695
Spartobacteria bacterium UBA6821
Spartobacteria bacterium UBA5019
Prosthecobacter debontii
Akkermansiaceae bacterium UBA6138
Akkermansia muciniphila
Akkermansiaceae bacterium UBA956
Rubritalea marina
Akkermansiaceae bacterium UBA4581
Akkermansiaceae bacterium UBA5688
Roseibacillus sp. ARS40
Verrucomicrobiae bacterium DG1235
Opitutaceae bacterium EAC85
Verrucomicrobia bacterium IMCC26134
Opitutaceae bacterium TAV5
Nibricoccus aquaticus
Opitutae bacterium Tous-C4FEB
Cephaloticoccus capnophilus
Opitutaceae bacterium UBA2377
Opitutales bacterium UBA2995
Opitutae bacterium UBA7397
Opitutae bacterium UBA6410
Opitutae bacterium UBA1977
Opitutae bacterium UBA3534
Verrucomicrobia bacterium CG1_02_43_26
Opitutae bacterium UBA3151
Opitutae bacterium NP117
Opitutae bacterium UBA4506
Opitutae bacterium UBA977
Opitutae bacterium UBA7445
Coraliomargarita akajimensis
Coraliomargarita sp. NAT145
Chlamydiae bacterium K940_chlam_9
Chlamydiae bacterium 3300011418-10
Clavichlamydia salmonicola
Chlamydophila pecorum
Chlamydia sp. 2742-308
Chlamydophila corallus
Chlamydophila pneumoniae
Chlamydophila ibidis
Chlamydia trachomatis
Chlamydia suis
Chlamydia muridarum
Chlamydophila avium
Chlamydia gallinacea
Chlamydophila abortus
Chlamydophila psittaci
Chlamydia felis
Chlamydophila caviae GPIC
Chlamydiae bacterium 1039689-34
Waddliaceae bacterium SP13
Chlamydiae bacterium 3300011418-14
Chlamydiae bacterium K940_chlam_3
Estrella lausannensis
Criblamydia sequanensis
Waddlia chondrophila
Chlamydiae bacterium K940_chlam_7
Chlamydiae bacterium 3300009084-150
Parachlamydiaceae bacterium HS-T3
Chlamydiales bacterium STE3
Parachlamydia sp. isolate BC.030
Parachlamydia acanthamoebae
Chlamydiae bacterium 3300009787-37
Parachlamydia sp. C2
Candidatus Protochlamydia naegleriophila
Candidatus Protochlamydia amoebophila
Protochlamydia sp. ACF82
Chlamydiae bacterium 3300020889-1
Chlamydiae bacterium 3300020740-2
Chlamydiae bacterium 3300009084-136
Chlamydiae bacterium 3300009083-135
Candidatus Rubidus massiliensis
Chlamydiales bacterium 38-26
Neochlamydia sp. ACF65
Neochlamydia sp. TUME1
Neochlamydia sp. EPS4
Chlamydiae bacterium K940_chlam_8
Chlamydiae bacterium 3300012931-71
Chlamydiae bacterium 1021942-14
Chlamydiae bacterium 3300006177-14
Chlamydiae bacterium RIFCSPLOWO2_02_FULL_45_22
Chlamydiae bacterium CG10_big_fil_rev_8_21_14_0_10_42_34
Chlamydiae bacterium 3300009703-49
Chlamydiae bacterium SM23_39
Anoxychlamydiales bacterium K1060_chlam_5
Anoxychlamydiales bacterium K1000_chlam_1
Anoxychlamydiales bacterium KR126_chlam_6
Anoxychlamydiales bacterium K1060_chlam_1
Anoxychlamydiales bacterium KR126_chlam_4
Anoxychlamydiales bacterium KR126_chlam_5
Anoxychlamydiales bacterium K940_chlam_5
Chlamydiae bacterium 3300006056-139
Chlamydiae bacterium RIFCSPHIGHO2_12_FULL_49_11
Chlamydiae bacterium 3300013126-109
Chlamydiae bacterium 3300013133-59
Chlamydiae bacterium 3300013132-109
Chlamydiae bacterium 1063924-70
Chlamydiae bacterium 1045240-7
Simkania negevensis
Sygnamydia xenoturbellae
Neptunochlamydia vexilliferae
Syngnamydia medusae
Chlamydiae bacterium 3300006056-100
Chlamydiae bacterium K1060_chlam_2
Chlamydiae bacterium 1049244-12
Chlamydiae bacterium 3300004085-10
Chlamydiae bacterium 1045192-5
Chlamydiae bacterium KR126_chlam_1
Chlamydiae bacterium 1096102-30
Chlamydiae bacterium KR126_chlam_3
Chlamydiae bacterium K940_chlam_6
Chlamydiae bacterium RIFCSPLOWO2_02_FULL_49_12
Chlamydiae bacterium 3300009709-46
Chlamydiae bacterium 1159587-4
Chlamydiae bacterium 1095360-24
Chlamydiae bacterium 1021942-13
Chlamydiae sp. genome_bin_33
Chlamydiae bacterium 3300020970-2
Chlamydiae bacterium 1064120-29
Chlamydiae bacterium 3300005529-103
Rhabdochlamydia helveticae
Rhabdochlamydia porcillionis
Rhabdochlamydia oedothoracis
0.54
0.53
0.1

chain3

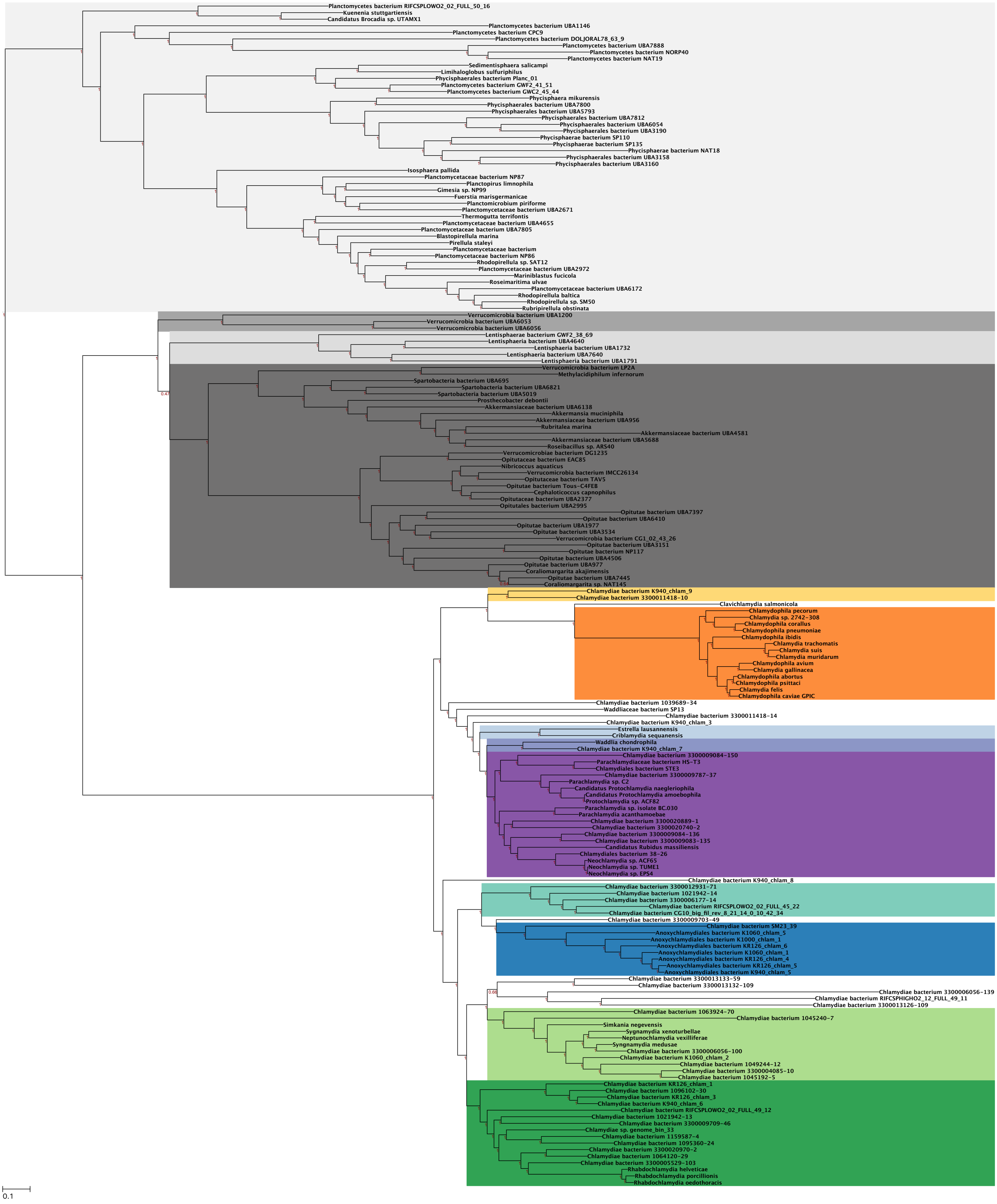

Planctomycetes bacterium RIFCSPLOWO2_02_FULL_50_16
Kuenenia stuttgartiensis
Candidatus Brocadia sp. UTAMX1
Planctomycetes bacterium UBA1146
Planctomycetes bacterium CPC9
Planctomycetes bacterium DOLJORAL78_63_9
Planctomycetes bacterium UBA7888
Planctomycetes bacterium NORP40
Planctomycetes bacterium NAT19
Sedimentisphaera salicampi
Limihaloglobus sulfuriphilus
Phycisphaerales bacterium Planc_01
Planctomycetes bacterium GWF2_41_51
Planctomycetes bacterium GWC2_45_44
Phycisphaera mikurensis
Phycisphaerales bacterium UBA7800
Phycisphaerales bacterium UBA5793
Phycisphaerales bacterium UBA7812
Phycisphaerales bacterium UBA6054
Phycisphaerales bacterium UBA3190
Phycisphaerae bacterium SP110
Phycisphaerae bacterium SP135
Phycisphaerae bacterium NAT18
Phycisphaerales bacterium UBA3158
Phycisphaerales bacterium UBA3160
Isosphaera pallida
Planctomycetaceae bacterium NP87
Planctopirus limnophila
Gimesia sp. NP99
Fuerstia marisgermanicae
Planctomicrobium piriforme
Planctomycetaceae bacterium UBA2671
Thermogutta terrifontis
Planctomycetaceae bacterium UBA4655
Planctomycetaceae bacterium UBA7805
Blastopirellula marina
Pirellula staleyi
Planctomycetaceae bacterium
Planctomycetaceae bacterium NP86
Rhodopirellula sp. SAT12
Planctomycetaceae bacterium UBA2972
Mariniblastus fucicola
Roseimaritima ulvae
Planctomycetaceae bacterium UBA6172
Rhodopirellula baltica
Rhodopirellula sp. SM50
Rubripirellula obstinata
Verrucomicrobia bacterium UBA1200
Verrucomicrobia bacterium UBA6053
Verrucomicrobia bacterium UBA6056
Lentisphaerae bacterium GWF2_38_69
Lentisphaeria bacterium UBA4640
Lentisphaeria bacterium UBA1732
Lentisphaeria bacterium UBA7640
Lentisphaeria bacterium UBA1791
Verrucomicrobia bacterium LP2A
Methylacidiphilum infernorum
Spartobacteria bacterium UBA695
Spartobacteria bacterium UBA6821
Spartobacteria bacterium UBA5019
Prosthecobacter debontii
Akkermansiaceae bacterium UBA6138
Akkermansia muciniphila
Akkermansiaceae bacterium UBA956
Rubritalea marina
Akkermansiaceae bacterium UBA4581
Akkermansiaceae bacterium UBA5688
Roseibacillus sp. ARS40
Verrucomicrobiae bacterium DG1235
Opitutaceae bacterium EAC85
Nibricoccus aquaticus
Verrucomicrobia bacterium IMCC26134
Opitutaceae bacterium TAV5
Opitutae bacterium Tous-C4FEB
Cephaloticoccus capnophilus
Opitutaceae bacterium UBA2377
Opitutales bacterium UBA2995
Opitutae bacterium UBA7397
Opitutae bacterium UBA6410
Opitutae bacterium UBA1977
Opitutae bacterium UBA3534
Verrucomicrobia bacterium CG1_02_43_26
Opitutae bacterium UBA3151
Opitutae bacterium NP117
Opitutae bacterium UBA4506
Opitutae bacterium UBA977
Coraliomargarita akajimensis
Opitutae bacterium UBA7445
Coraliomargarita sp. NAT145
Chlamydiae bacterium K940_chlam_9
Chlamydiae bacterium 3300011418-10
Clavichlamydia salmonicola
Chlamydophila pecorum
Chlamydia sp. 2742-308
Chlamydophila corallus
Chlamydophila pneumoniae
Chlamydophila ibidis
Chlamydia trachomatis
Chlamydia suis
Chlamydia muridarum
Chlamydophila avium
Chlamydia gallinacea
Chlamydophila abortus
Chlamydophila psittaci
Chlamydia felis
Chlamydophila caviae GPIC
Chlamydiae bacterium 1039689-34
Waddliaceae bacterium SP13
Chlamydiae bacterium 3300011418-14
Chlamydiae bacterium K940_chlam_3
Estrella lausannensis
Criblamydia sequanensis
Waddlia chondrophila
Chlamydiae bacterium K940_chlam_7
Chlamydiae bacterium 3300009084-150
Parachlamydiaceae bacterium HS-T3
Chlamydiales bacterium STE3
Chlamydiae bacterium 3300009787-37
Parachlamydia sp. C2
Candidatus Protochlamydia naegleriophila
Candidatus Protochlamydia amoebophila
Protochlamydia sp. ACF82
Parachlamydia sp. isolate BC.030
Parachlamydia acanthamoebae
Chlamydiae bacterium 3300020889-1
Chlamydiae bacterium 3300020740-2
Chlamydiae bacterium 3300009084-136
Chlamydiae bacterium 3300009083-135
Candidatus Rubidus massiliensis
Chlamydiales bacterium 38-26
Neochlamydia sp. ACF65
Neochlamydia sp. TUME1
Neochlamydia sp. EPS4
Chlamydiae bacterium K940_chlam_8
Chlamydiae bacterium 3300012931-71
Chlamydiae bacterium 1021942-14
Chlamydiae bacterium 3300006177-14
Chlamydiae bacterium RIFCSPLOWO2_02_FULL_45_22
Chlamydiae bacterium CG10_big_fil_rev_8_21_14_0_10_42_34
Chlamydiae bacterium 3300009703-49
Chlamydiae bacterium SM23_39
Anoxychlamydiales bacterium K1060_chlam_5
Anoxychlamydiales bacterium K1000_chlam_1
Anoxychlamydiales bacterium KR126_chlam_6
Anoxychlamydiales bacterium K1060_chlam_1
Anoxychlamydiales bacterium KR126_chlam_4
Anoxychlamydiales bacterium KR126_chlam_5
Anoxychlamydiales bacterium K940_chlam_5
Chlamydiae bacterium 3300013133-59
Chlamydiae bacterium 3300013132-109
Chlamydiae bacterium 3300006056-139
Chlamydiae bacterium RIFCSPHIGHO2_12_FULL_49_11
Chlamydiae bacterium 3300013126-109
Chlamydiae bacterium 1063924-70
Chlamydiae bacterium 1045240-7
Simkania negevensis
Sygnamydia xenoturbellae
Neptunochlamydia vexilliferae
Syngnamydia medusae
Chlamydiae bacterium 3300006056-100
Chlamydiae bacterium K1060_chlam_2
Chlamydiae bacterium 1049244-12
Chlamydiae bacterium 3300004085-10
Chlamydiae bacterium 1045192-5
Chlamydiae bacterium KR126_chlam_1
Chlamydiae bacterium 1096102-30
Chlamydiae bacterium KR126_chlam_3
Chlamydiae bacterium K940_chlam_6
Chlamydiae bacterium RIFCSPLOWO2_02_FULL_49_12
Chlamydiae bacterium 1021942-13
Chlamydiae bacterium 3300009709-46
Chlamydiae sp. genome_bin_33
Chlamydiae bacterium 1159587-4
Chlamydiae bacterium 1095360-24
Chlamydiae bacterium 3300020970-2
Chlamydiae bacterium 1064120-29
Chlamydiae bacterium 3300005529-103
Rhabdochlamydia helveticae
Rhabdochlamydia porcellionis
Rhabdochlamydia oedothoracis
0.1

chain4

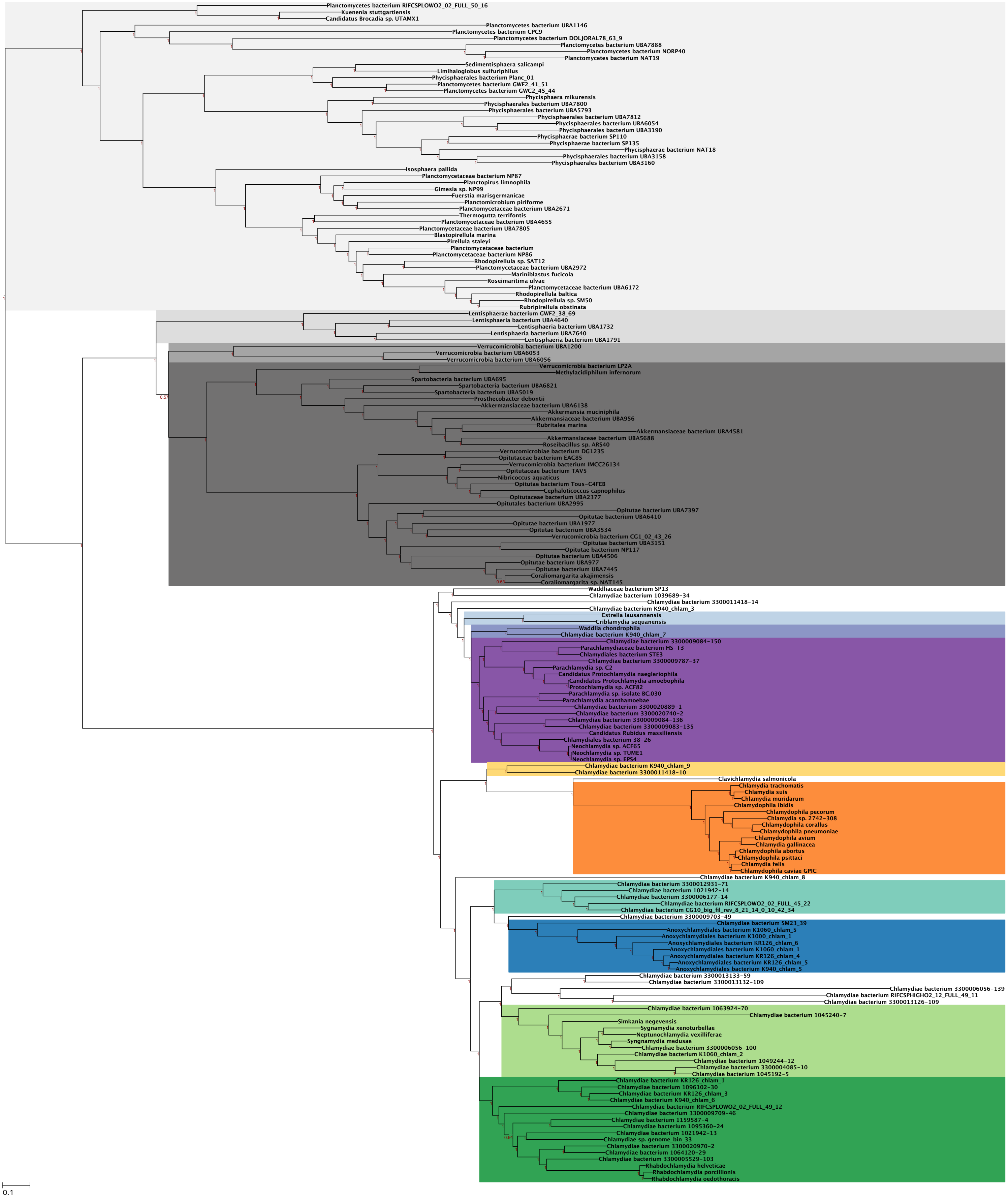
Planctomycetes bacterium RIFCSPLOWO2_02_FULL_50_16
Kuenenia stuttgartiensis
Candidatus Brocadia sp. UTAMX1
Planctomycetes bacterium UBA1146
Planctomycetes bacterium CPC9
Planctomycetes bacterium DOLJORAL78_63_9
Planctomycetes bacterium UBA7888
Planctomycetes bacterium NORP40
Planctomycetes bacterium NAT19
Sedimentisphaera salicampi
Limihaloglobus sulfuriphilus
Phycisphaerales bacterium Planc_01
Planctomycetes bacterium GWF2_41_51
Planctomycetes bacterium GWC2_45_44
Phycisphaera mikurensis
Phycisphaerales bacterium UBA7800
Phycisphaerales bacterium UBA5793
Phycisphaerales bacterium UBA7812
Phycisphaerales bacterium UBA6054
Phycisphaerales bacterium UBA3190
Phycisphaerae bacterium SP110
Phycisphaerae bacterium SP135
Phycisphaerae bacterium NAT18
Phycisphaerales bacterium UBA3158
Phycisphaerales bacterium UBA3160
Isosphaera pallida
Planctomycetaceae bacterium NP87
Planctopirus limnophila
Gimesia sp. NP99
Fuerstia marisgermanicae
Planctomicrobium piriforme
Planctomycetaceae bacterium UBA2671
Thermogutta terrifontis
Planctomycetaceae bacterium UBA4655
Planctomycetaceae bacterium UBA7805
Blastopirellula marina
Pirellula staleyi
Planctomycetaceae bacterium
Planctomycetaceae bacterium NP86
Rhodopirellula sp. SAT12
Planctomycetaceae bacterium UBA2972
Mariniblastus fucicola
Roseimaritima ulvae
Planctomycetaceae bacterium UBA6172
Rhodopirellula baltica
Rhodopirellula sp. SM50
Rubripirellula obstinata
Lentisphaerae bacterium GWF2_38_69
Lentisphaeria bacterium UBA4640
Lentisphaeria bacterium UBA1732
Lentisphaeria bacterium UBA7640
Lentisphaeria bacterium UBA1791
Verrucomicrobia bacterium UBA1200
Verrucomicrobia bacterium UBA6053
Verrucomicrobia bacterium UBA6056
Verrucomicrobia bacterium LP2A
Methylacidiphilum infernorum
Spartobacteria bacterium UBA695
Spartobacteria bacterium UBA6821
Spartobacteria bacterium UBA5019
Prosthecobacter debontii
Akkermansiaceae bacterium UBA6138
Akkermansia muciniphila
Akkermansiaceae bacterium UBA956
Rubritalea marina
Akkermansiaceae bacterium UBA4581
Akkermansiaceae bacterium UBA5688
Roseibacillus sp. ARS40
Verrucomicrobiae bacterium DG1235
Opitutaceae bacterium EAC85
Verrucomicrobia bacterium IMCC26134
Opitutaceae bacterium TAV5
Nibricoccus aquaticus
Opitutae bacterium Tous-C4FEB
Cephaloticoccus capnophilus
Opitutaceae bacterium UBA2377
Opitutales bacterium UBA2995
Opitutae bacterium UBA7397
Opitutae bacterium UBA6410
Opitutae bacterium UBA1977
Opitutae bacterium UBA3534
Verrucomicrobia bacterium CG1_02_43_26
Opitutae bacterium UBA3151
Opitutae bacterium NP117
Opitutae bacterium UBA4506
Opitutae bacterium UBA977
Opitutae bacterium UBA7445
Coraliomargarita akajimensis
Coraliomargarita sp. NAT145
0.57
0.63
Waddliaceae bacterium SP13
Chlamydiae bacterium 1039689-34
Chlamydiae bacterium 3300011418-14
Chlamydiae bacterium K940_chlam_3
Estrella lausannensis
Criblamydia sequanensis
Waddlia chondrophila
Chlamydiae bacterium K940_chlam_7
Chlamydiae bacterium 3300009084-150
Parachlamydiaceae bacterium HS-T3
Chlamydiales bacterium STE3
Chlamydiae bacterium 3300009787-37
Parachlamydia sp. C2
Candidatus Protochlamydia naegleriophila
Candidatus Protochlamydia amoebophila
Protochlamydia sp. ACF82
Parachlamydia sp. isolate BC.030
Parachlamydia acanthamoebae
Chlamydiae bacterium 3300020889-1
Chlamydiae bacterium 3300020740-2
Chlamydiae bacterium 3300009084-136
Chlamydiae bacterium 3300009083-135
Candidatus Rubidus massiliensis
Chlamydiales bacterium 38-26
Neochlamydia sp. ACF65
Neochlamydia sp. TUME1
Neochlamydia sp. EPS4
Chlamydiae bacterium K940_chlam_9
Chlamydiae bacterium 3300011418-10
Clavichlamydia salmonicola
Chlamydia trachomatis
Chlamydia suis
Chlamydia muridarum
Chlamydophila ibidis
Chlamydophila pecorum
Chlamydia sp. 2742-308
Chlamydophila corallus
Chlamydophila pneumoniae
Chlamydophila avium
Chlamydia gallinacea
Chlamydophila abortus
Chlamydophila psittaci
Chlamydia felis
Chlamydophila caviae GPIC
Chlamydiae bacterium K940_chlam_8
Chlamydiae bacterium 3300012931-71
Chlamydiae bacterium 1021942-14
Chlamydiae bacterium 3300006177-14
Chlamydiae bacterium RIFCSPLOWO2_02_FULL_45_22
Chlamydiae bacterium CG10_big_fil_rev_8_21_14_0_10_42_34
Chlamydiae bacterium 3300009703-49
Chlamydiae bacterium SM23_39
Anoxychlamydiales bacterium K1060_chlam_5
Anoxychlamydiales bacterium K1000_chlam_1
Anoxychlamydiales bacterium KR126_chlam_6
Anoxychlamydiales bacterium K1060_chlam_1
Anoxychlamydiales bacterium KR126_chlam_4
Anoxychlamydiales bacterium KR126_chlam_5
Anoxychlamydiales bacterium K940_chlam_5
Chlamydiae bacterium 3300013133-59
Chlamydiae bacterium 3300013132-109
Chlamydiae bacterium 3300006056-139
Chlamydiae bacterium RIFCSPHIGHO2_12_FULL_49_11
Chlamydiae bacterium 3300013126-109
Chlamydiae bacterium 1063924-70
Chlamydiae bacterium 1045240-7
Simkania negevensis
Sygnamydia xenoturbellae
Neptunochlamydia vexilliferae
Syngnamydia medusae
Chlamydiae bacterium 3300006056-100
Chlamydiae bacterium K1060_chlam_2
Chlamydiae bacterium 1049244-12
Chlamydiae bacterium 3300004085-10
Chlamydiae bacterium 1045192-5
Chlamydiae bacterium KR126_chlam_1
Chlamydiae bacterium 1096102-30
Chlamydiae bacterium KR126_chlam_3
Chlamydiae bacterium K940_chlam_6
Chlamydiae bacterium RIFCSPLOWO2_02_FULL_49_12
Chlamydiae bacterium 3300009709-46
Chlamydiae bacterium 1159587-4
Chlamydiae bacterium 1095360-24
Chlamydiae bacterium 1021942-13
0.54
Chlamydiae sp. genome_bin_33
Chlamydiae bacterium 3300020970-2
Chlamydiae bacterium 1064120-29
Chlamydiae bacterium 3300005529-103
Rhabdochlamydia helveticae
Rhabdochlamydia porcillionis
Rhabdochlamydia oedothoracis
0.1

convergence_chains_1234

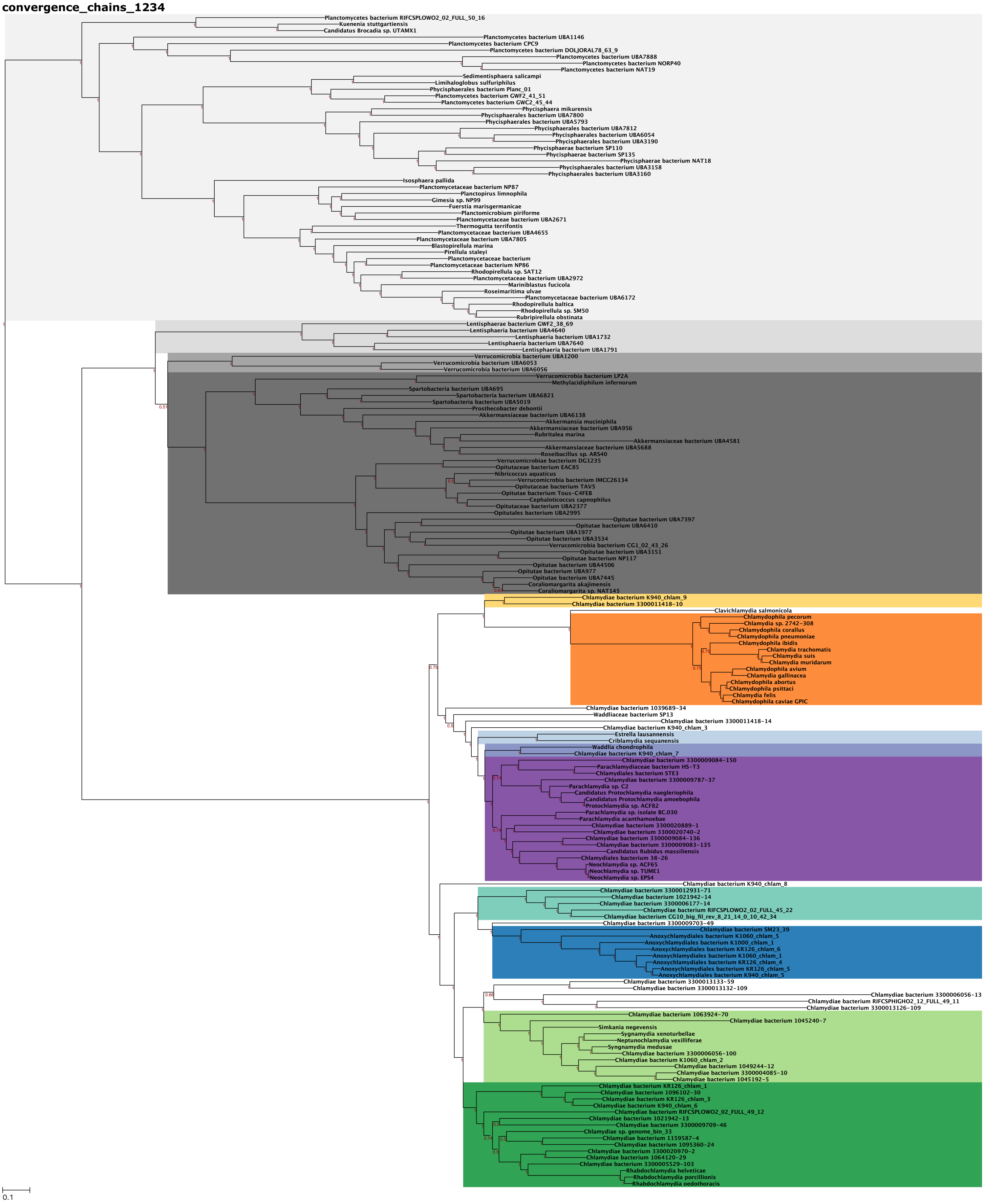

180taxa_10perc_pruned

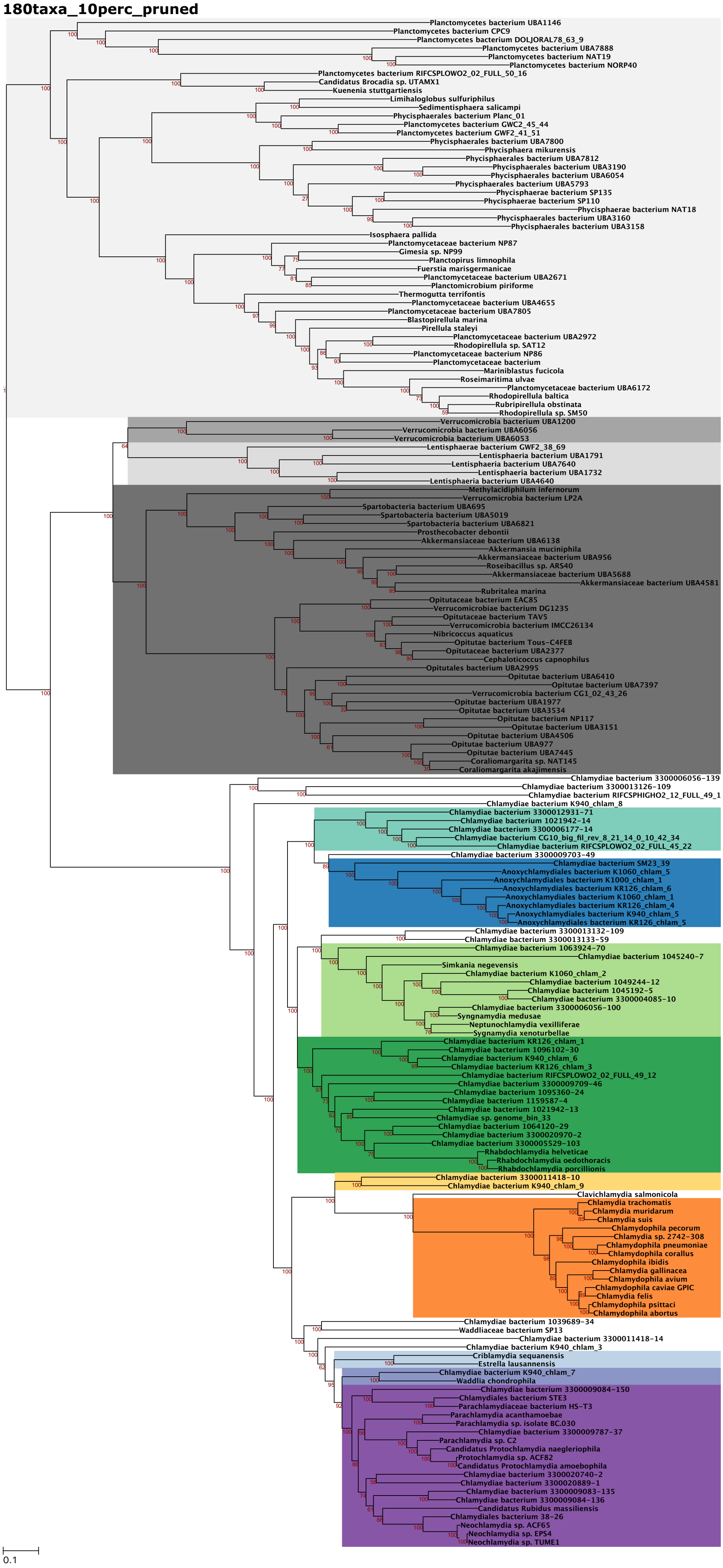

180taxa_20perc_pruned

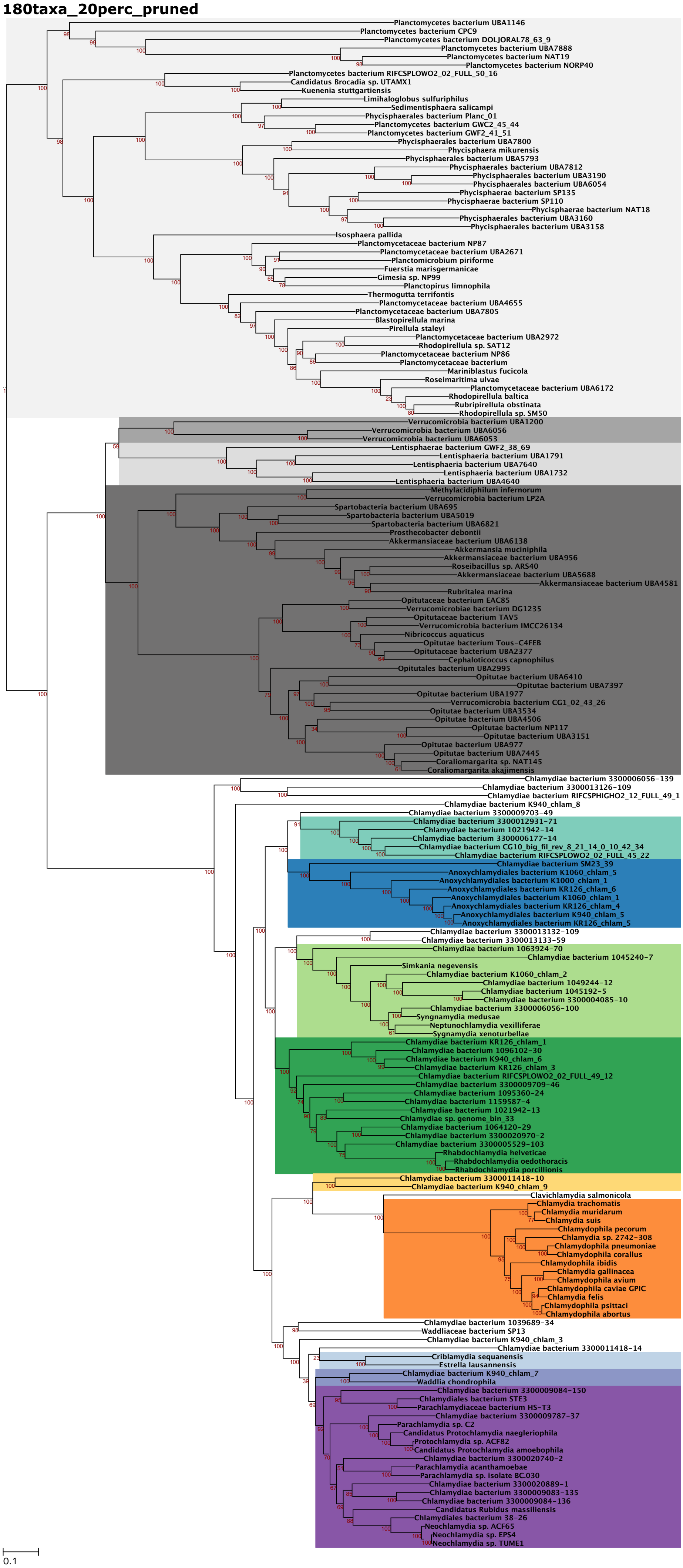

180taxa_30perc_pruned

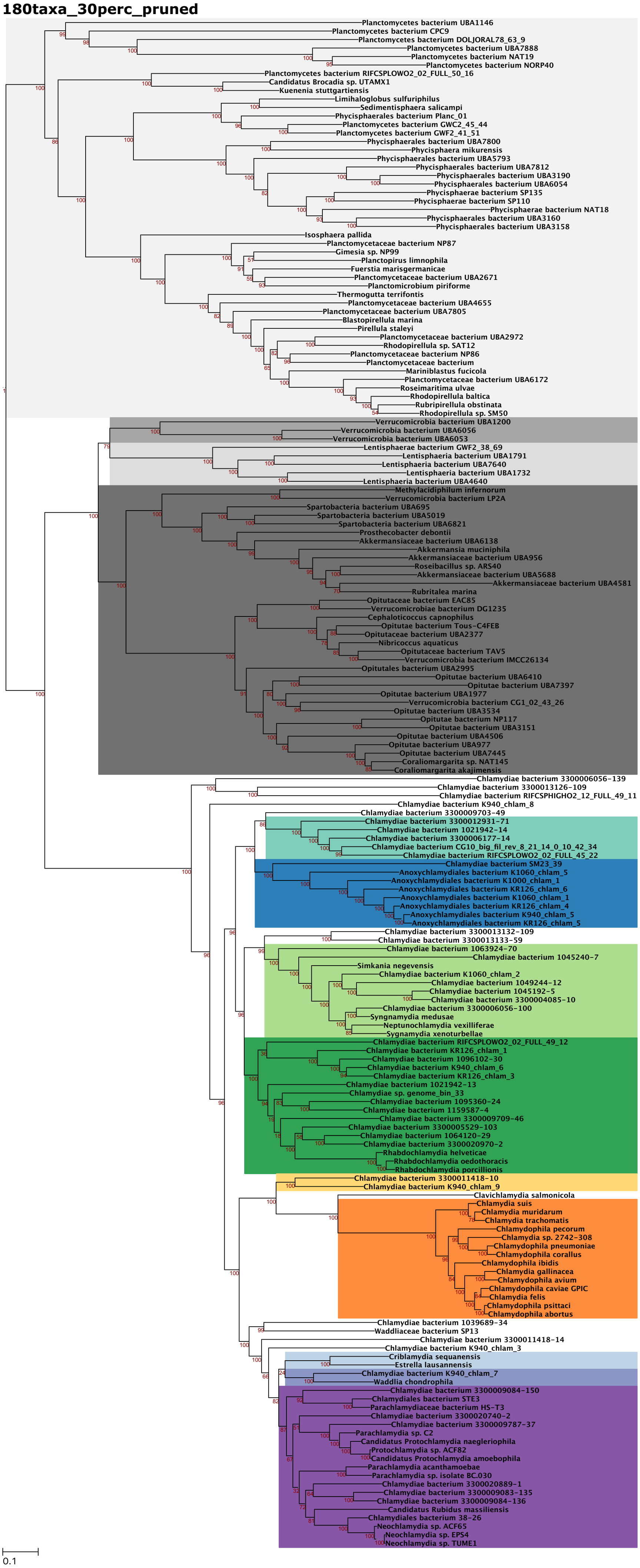

180taxa_40perc_pruned

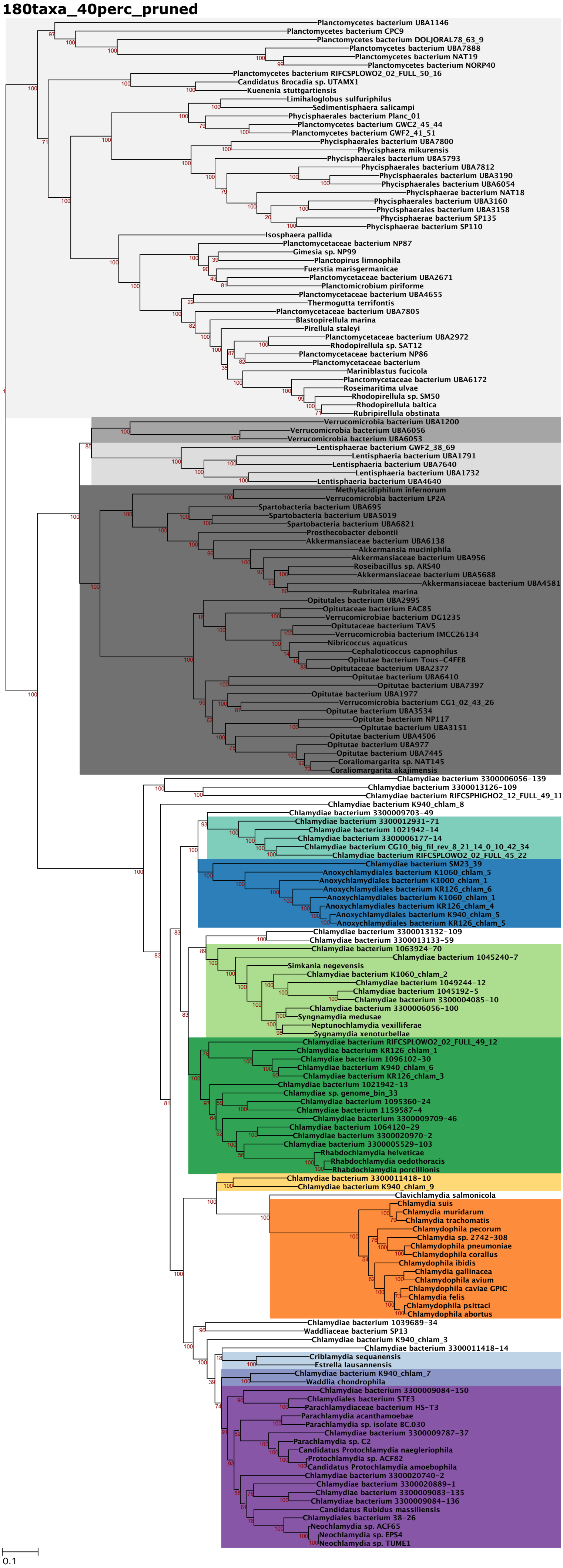

180taxa_50perc_pruned

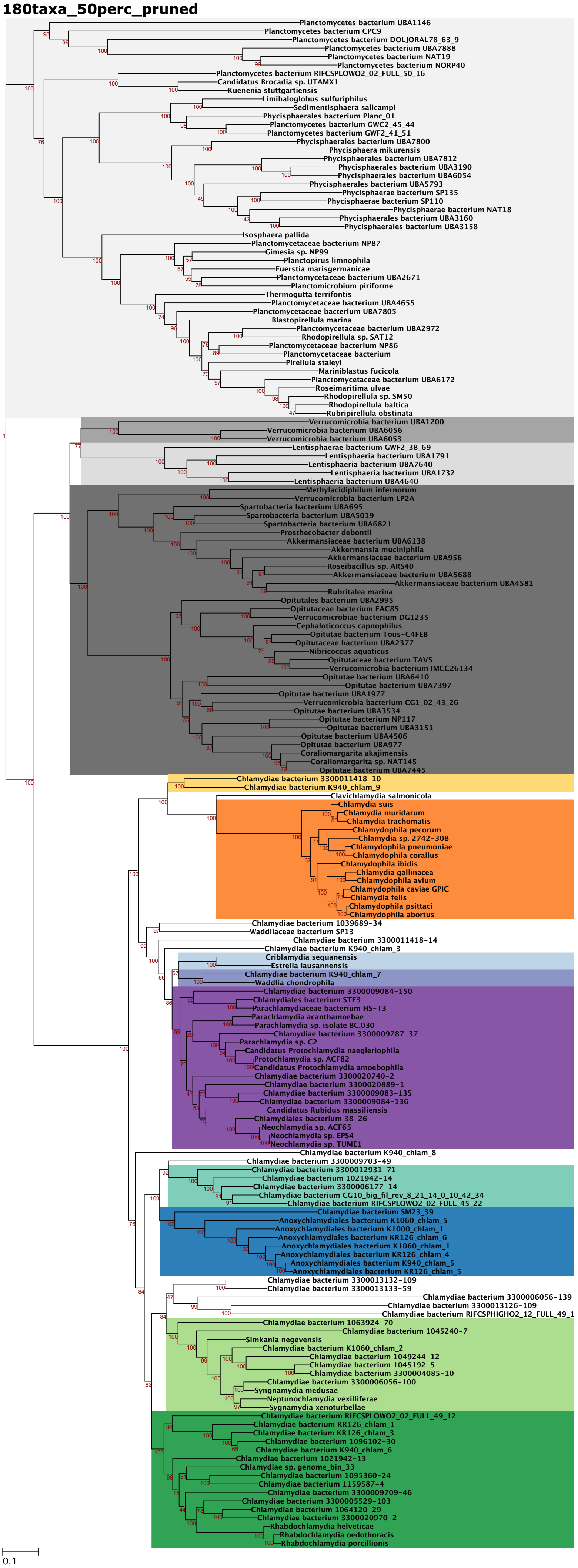

180taxa_51perc_pruned_bias_removed

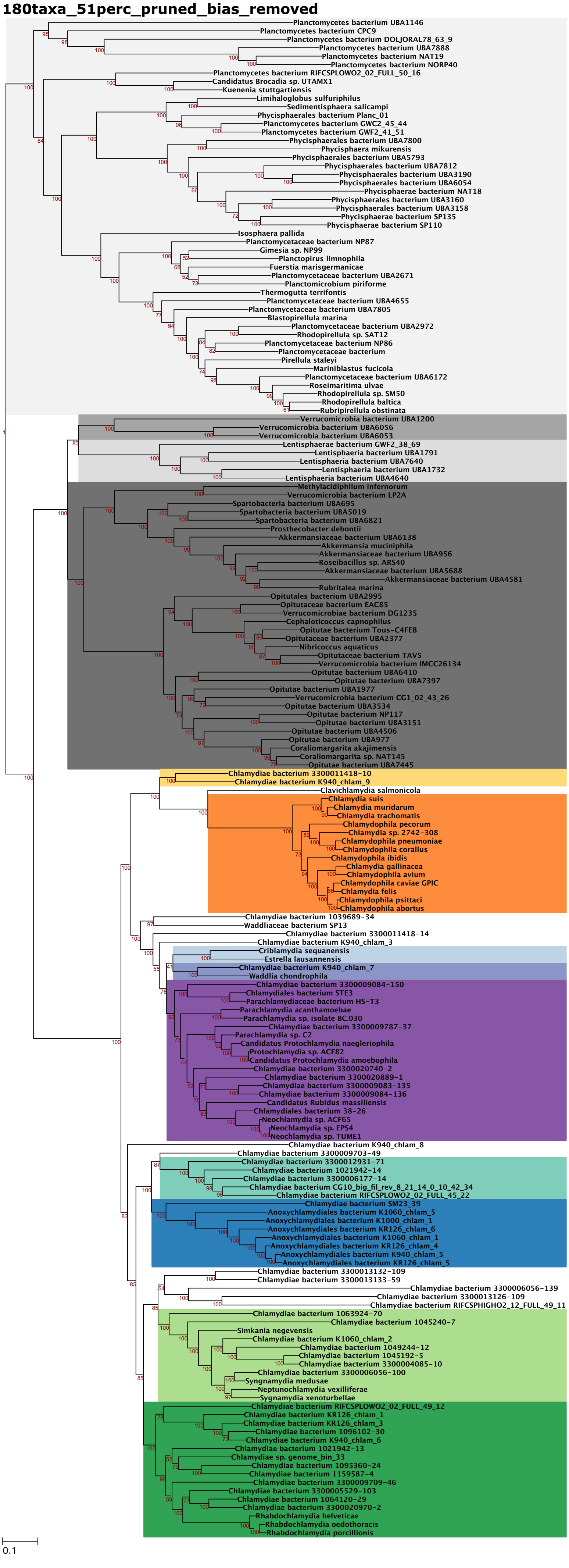

180taxa_51perc_pruned_bias_removed

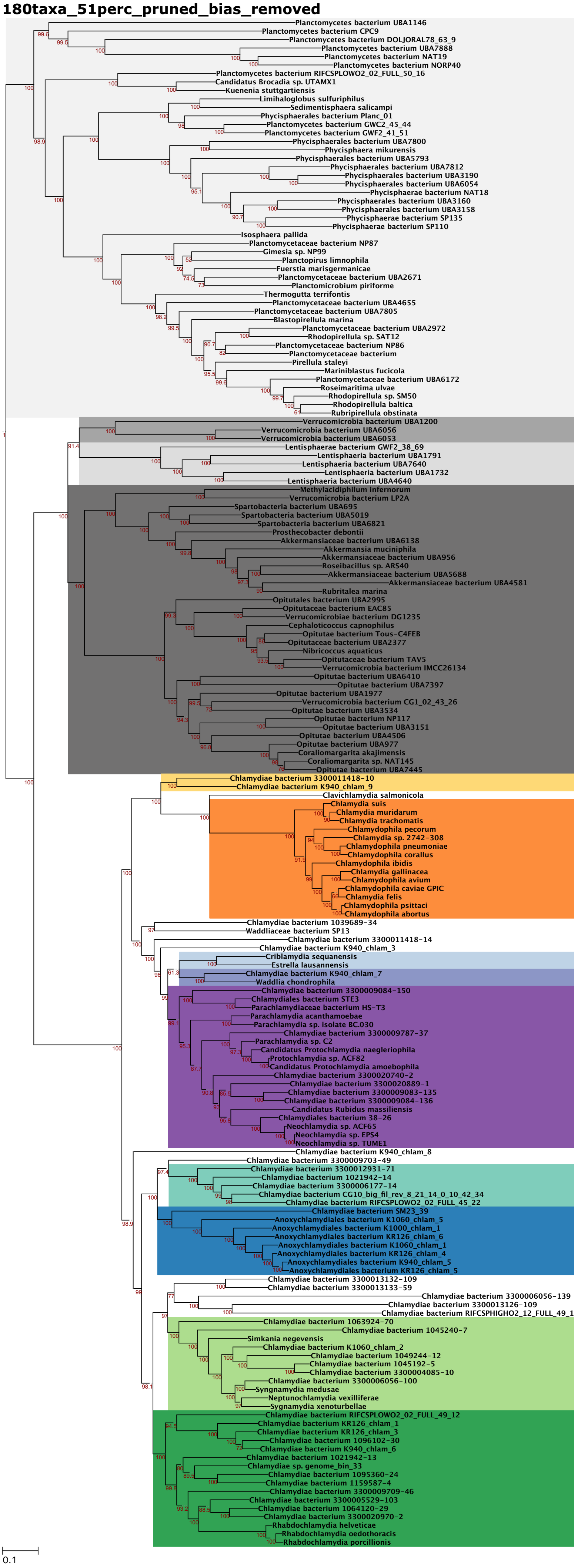

chain1

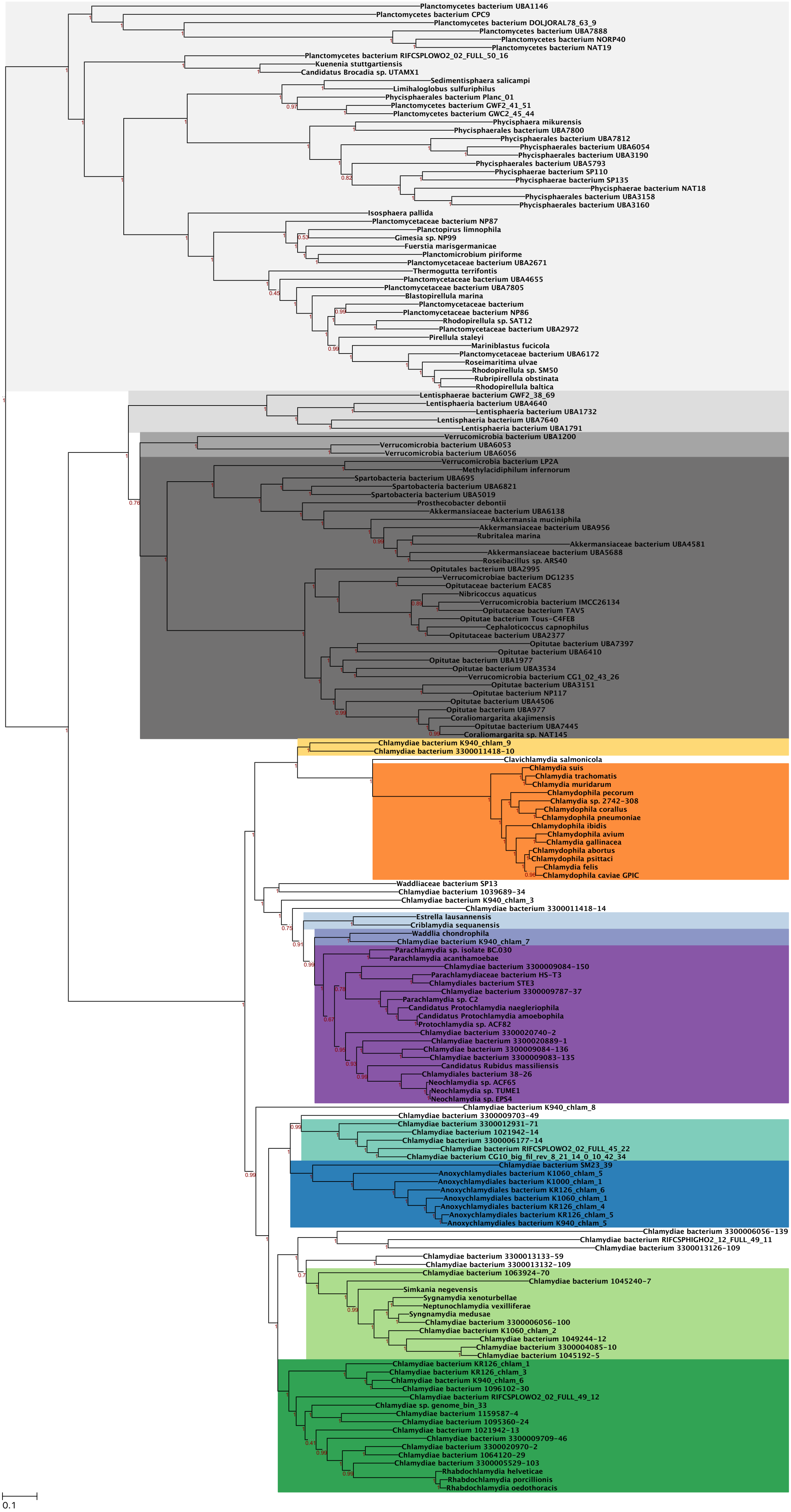

Planctomycetes bacterium UBA1146
Planctomycetes bacterium CPC9
Planctomycetes bacterium DOLJORAL78_63_9
Planctomycetes bacterium UBA7888
Planctomycetes bacterium NORP40
Planctomycetes bacterium NAT19
Planctomycetes bacterium RIFCSPLOWO2_02_FULL_50_16
Kuenenia stuttgartiensis
Candidatus Brocadia sp. UTAMX1
Sedimentisphaera salicampi
Limihaloglobus sulfuriphilus
Phycisphaerales bacterium Planc_01
Planctomycetes bacterium GWF2_41_51
Planctomycetes bacterium GWC2_45_44
Phycisphaera mikurensis
Phycisphaerales bacterium UBA7800
Phycisphaerales bacterium UBA7812
Phycisphaerales bacterium UBA6054
Phycisphaerales bacterium UBA3190
Phycisphaerales bacterium UBA5793
Phycisphaerae bacterium SP110
Phycisphaerae bacterium SP135
Phycisphaerae bacterium NAT18
Phycisphaerales bacterium UBA3158
Phycisphaerales bacterium UBA3160
Isosphaera pallida
Planctomycetaceae bacterium NP87
Planctopirus limnophila
Gimesia sp. NP99
Fuerstia marisgermanicae
Planctomicrobium piriforme
Planctomycetaceae bacterium UBA2671
Thermogutta terrifontis
Planctomycetaceae bacterium UBA4655
Planctomycetaceae bacterium UBA7805
Blastopirellula marina
Planctomycetaceae bacterium
Planctomycetaceae bacterium NP86
Rhodopirellula sp. SAT12
Planctomycetaceae bacterium UBA2972
Pirellula staleyi
Mariniblastus fucicola
Planctomycetaceae bacterium UBA6172
Roseimaritima ulvae
Rhodopirellula sp. SM50
Rubripirellula obstinata
Rhodopirellula baltica
Lentisphaerae bacterium GWF2_38_69
Lentisphaeria bacterium UBA4640
Lentisphaeria bacterium UBA1732
Lentisphaeria bacterium UBA7640
Lentisphaeria bacterium UBA1791
Verrucomicrobia bacterium UBA1200
Verrucomicrobia bacterium UBA6053
Verrucomicrobia bacterium UBA6056
Verrucomicrobia bacterium LP2A
Methylacidiphilum infernorum
Spartobacteria bacterium UBA695
Spartobacteria bacterium UBA6821
Spartobacteria bacterium UBA5019
Prosthecobacter debontii
Akkermansiaceae bacterium UBA6138
Akkermansia muciniphila
Akkermansiaceae bacterium UBA956
Rubritalea marina
Akkermansiaceae bacterium UBA4581
Akkermansiaceae bacterium UBA5688
Roseibacillus sp. ARS40
Opitutales bacterium UBA2995
Verrucomicrobiae bacterium DG1235
Opitutaceae bacterium EAC85
Nibricoccus aquaticus
Verrucomicrobia bacterium IMCC26134
Opitutaceae bacterium TAV5
Opitutae bacterium Tous-C4FEB
Cephaloticoccus capnophilus
Opitutaceae bacterium UBA2377
Opitutae bacterium UBA7397
Opitutae bacterium UBA6410
Opitutae bacterium UBA1977
Opitutae bacterium UBA3534
Verrucomicrobia bacterium CG1_02_43_26
Opitutae bacterium UBA3151
Opitutae bacterium NP117
Opitutae bacterium UBA4506
Opitutae bacterium UBA977
Coraliomargarita akajimensis
Opitutae bacterium UBA7445
Coraliomargarita sp. NAT145
Chlamydiae bacterium K940_chlam_9
Chlamydiae bacterium 3300011418-10
Clavichlamydia salmonicola
Chlamydia suis
Chlamydia trachomatis
Chlamydia muridarum
Chlamydophila pecorum
Chlamydia sp. 2742-308
Chlamydophila corallus
Chlamydophila pneumoniae
Chlamydophila ibidis
Chlamydophila avium
Chlamydia gallinacea
Chlamydophila abortus
Chlamydophila psittaci
Chlamydia felis
Chlamydophila caviae GPIC
Waddliaceae bacterium SP13
Chlamydiae bacterium 1039689-34
Chlamydiae bacterium K940_chlam_3
Chlamydiae bacterium 3300011418-14
Estrella lausannensis
Criblamydia sequanensis
Waddlia chondrophila
Chlamydiae bacterium K940_chlam_7
Parachlamydia sp. isolate BC.030
Parachlamydia acanthamoebae
Chlamydiae bacterium 3300009084-150
Parachlamydiaceae bacterium HS-T3
Chlamydiales bacterium STE3
Chlamydiae bacterium 3300009787-37
Parachlamydia sp. C2
Candidatus Protochlamydia naegleriophila
Candidatus Protochlamydia amoebophila
Protochlamydia sp. ACF82
Chlamydiae bacterium 3300020740-2
Chlamydiae bacterium 3300020889-1
Chlamydiae bacterium 3300009084-136
Chlamydiae bacterium 3300009083-135
Candidatus Rubidus massiliensis
Chlamydiales bacterium 38-26
Neochlamydia sp. ACF65
Neochlamydia sp. TUME1
Neochlamydia sp. EPS4
Chlamydiae bacterium K940_chlam_8
Chlamydiae bacterium 3300009703-49
Chlamydiae bacterium 3300012931-71
Chlamydiae bacterium 1021942-14
Chlamydiae bacterium 3300006177-14
Chlamydiae bacterium RIFCSPLOWO2_02_FULL_45_22
Chlamydiae bacterium CG10_big_fil_rev_8_21_14_0_10_42_34
Chlamydiae bacterium SM23_39
Anoxychlamydiales bacterium K1060_chlam_5
Anoxychlamydiales bacterium K1000_chlam_1
Anoxychlamydiales bacterium KR126_chlam_6
Anoxychlamydiales bacterium K1060_chlam_1
Anoxychlamydiales bacterium KR126_chlam_4
Anoxychlamydiales bacterium KR126_chlam_5
Anoxychlamydiales bacterium K940_chlam_5
Chlamydiae bacterium 3300006056-139
Chlamydiae bacterium RIFCSPHIGHO2_12_FULL_49_11
Chlamydiae bacterium 3300013126-109
Chlamydiae bacterium 3300013133-59
Chlamydiae bacterium 3300013132-109
Chlamydiae bacterium 1063924-70
Chlamydiae bacterium 1045240-7
Simkania negevensis
Sygnamydia xenoturbellae
Neptunochlamydia vexilliferae
Syngnamydia medusae
Chlamydiae bacterium 3300006056-100
Chlamydiae bacterium K1060_chlam_2
Chlamydiae bacterium 1049244-12
Chlamydiae bacterium 3300004085-10
Chlamydiae bacterium 1045192-5
Chlamydiae bacterium KR126_chlam_1
Chlamydiae bacterium KR126_chlam_3
Chlamydiae bacterium K940_chlam_6
Chlamydiae bacterium 1096102-30
Chlamydiae bacterium RIFCSPLOWO2_02_FULL_49_12
Chlamydiae sp. genome_bin_33
Chlamydiae bacterium 1159587-4
Chlamydiae bacterium 1095360-24
Chlamydiae bacterium 1021942-13
Chlamydiae bacterium 3300009709-46
Chlamydiae bacterium 3300020970-2
Chlamydiae bacterium 1064120-29
Chlamydiae bacterium 3300005529-103
Rhabdochlamydia helveticae
Rhabdochlamydia porcillionis
Rhabdochlamydia oedothoracis
0.1

chain2

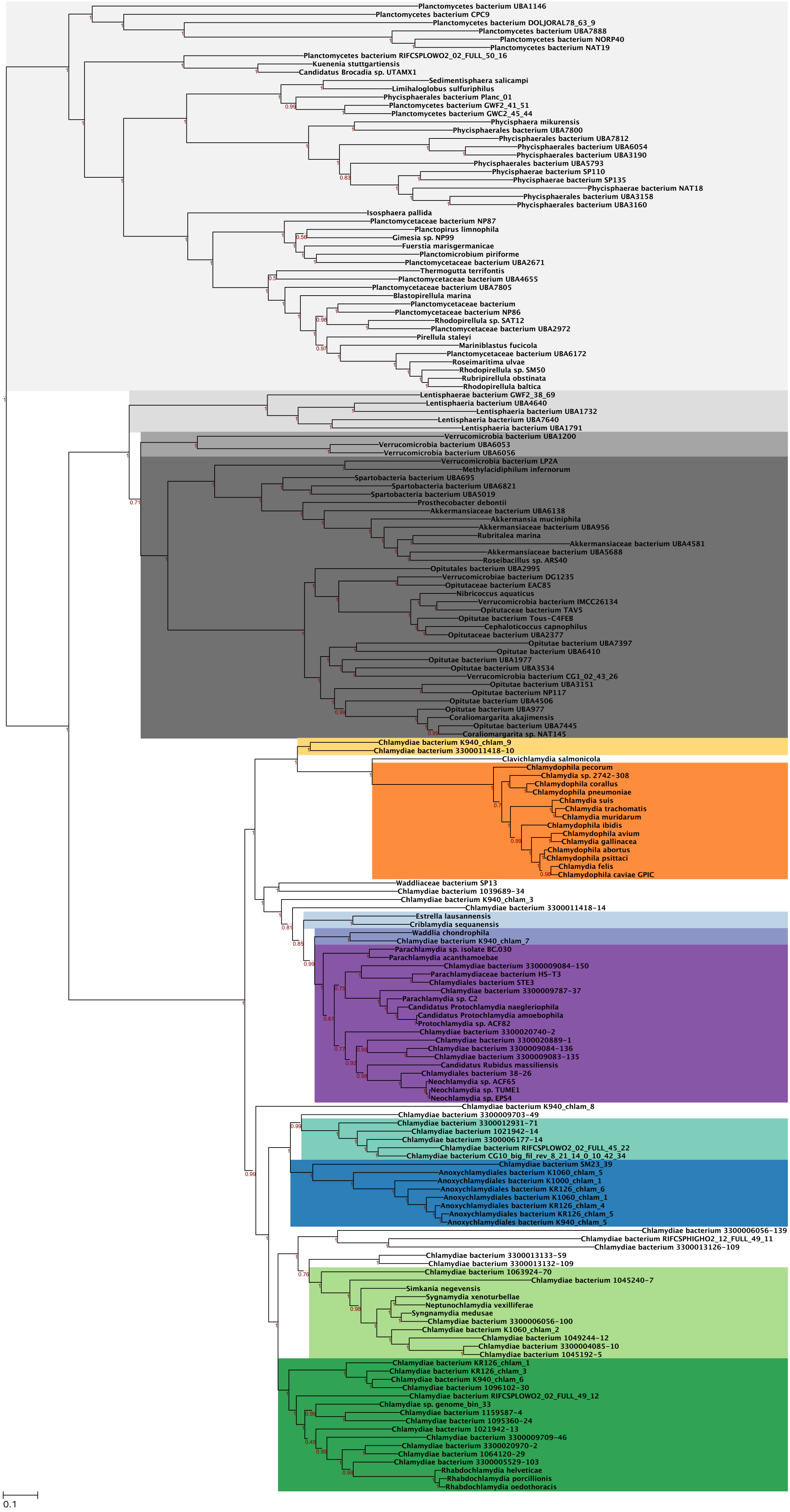
Planctomycetes bacterium UBA1146
Planctomycetes bacterium CPC9
Planctomycetes bacterium DOLJORAL78_63_9
Planctomycetes bacterium UBA7888
Planctomycetes bacterium NORP40
Planctomycetes bacterium NAT19
Planctomycetes bacterium RIFCSPLOWO2_02_FULL_50_16
Kuenenia stuttgartiensis
Candidatus Brocadia sp. UTAMX1
Sedimentisphaera salicampi
Limihaloglobus sulfuriphilus
Phycisphaerales bacterium Planc_01
Planctomycetes bacterium GWF2_41_51
Planctomycetes bacterium GWC2_45_44
Phycisphaera mikurensis
Phycisphaerales bacterium UBA7800
Phycisphaerales bacterium UBA7812
Phycisphaerales bacterium UBA6054
Phycisphaerales bacterium UBA3190
Phycisphaerales bacterium UBA5793
Phycisphaerae bacterium SP110
Phycisphaerae bacterium SP135
Phycisphaerae bacterium NAT18
Phycisphaerales bacterium UBA3158
Phycisphaerales bacterium UBA3160
Isosphaera pallida
Planctomycetaceae bacterium NP87
Planctopirus limnophila
Gimesia sp. NP99
Fuerstia marisgermanicae
Planctomicrobium piriforme
Planctomycetaceae bacterium UBA2671
Thermogutta terrifontis
Planctomycetaceae bacterium UBA4655
Planctomycetaceae bacterium UBA7805
Blastopirellula marina
Planctomycetaceae bacterium
Planctomycetaceae bacterium NP86
Rhodopirellula sp. SAT12
Planctomycetaceae bacterium UBA2972
Pirellula staleyi
Mariniblastus fucicola
Planctomycetaceae bacterium UBA6172
Roseimaritima ulvae
Rhodopirellula sp. SM50
Rubripirellula obstinata
Rhodopirellula baltica
Lentisphaerae bacterium GWF2_38_69
Lentisphaeria bacterium UBA4640
Lentisphaeria bacterium UBA1732
Lentisphaeria bacterium UBA7640
Lentisphaeria bacterium UBA1791
Verrucomicrobia bacterium UBA1200
Verrucomicrobia bacterium UBA6053
Verrucomicrobia bacterium UBA6056
Verrucomicrobia bacterium LP2A
Methylacidiphilum infernorum
Spartobacteria bacterium UBA695
Spartobacteria bacterium UBA6821
Spartobacteria bacterium UBA5019
Prosthecobacter debontii
Akkermansiaceae bacterium UBA6138
Akkermansia muciniphila
Akkermansiaceae bacterium UBA956
Rubritalea marina
Akkermansiaceae bacterium UBA4581
Akkermansiaceae bacterium UBA5688
Roseibacillus sp. ARS40
Opitutales bacterium UBA2995
Verrucomicrobiae bacterium DG1235
Opitutaceae bacterium EAC85
Nibricoccus aquaticus
Verrucomicrobia bacterium IMCC26134
Opitutaceae bacterium TAV5
Opitutae bacterium Tous-C4FEB
Cephaloticoccus capnophilus
Opitutaceae bacterium UBA2377
Opitutae bacterium UBA7397
Opitutae bacterium UBA6410
Opitutae bacterium UBA1977
Opitutae bacterium UBA3534
Verrucomicrobia bacterium CG1_02_43_26
Opitutae bacterium UBA3151
Opitutae bacterium NP117
Opitutae bacterium UBA4506
Opitutae bacterium UBA977
Coraliomargarita akajimensis
Opitutae bacterium UBA7445
Coraliomargarita sp. NAT145
Chlamydiae bacterium K940_chlam_9
Chlamydiae bacterium 3300011418-10
Clavichlamydia salmonicola
Chlamydophila pecorum
Chlamydia sp. 2742-308
Chlamydophila corallus
Chlamydophila pneumoniae
Chlamydia suis
Chlamydia trachomatis
Chlamydia muridarum
Chlamydophila ibidis
Chlamydophila avium
Chlamydia gallinacea
Chlamydophila abortus
Chlamydophila psittaci
Chlamydia felis
Chlamydophila caviae GPIC
Waddliaceae bacterium SP13
Chlamydiae bacterium 1039689-34
Chlamydiae bacterium K940_chlam_3
Chlamydiae bacterium 3300011418-14
Estrella lausannensis
Criblamydia sequanensis
Waddlia chondrophila
Chlamydiae bacterium K940_chlam_7
Parachlamydia sp. isolate BC.030
Parachlamydia acanthamoebae
Chlamydiae bacterium 3300009084-150
Parachlamydiaceae bacterium HS-T3
Chlamydiales bacterium STE3
Chlamydiae bacterium 3300009787-37
Parachlamydia sp. C2
Candidatus Protochlamydia naegleriophila
Candidatus Protochlamydia amoebophila
Protochlamydia sp. ACF82
Chlamydiae bacterium 3300020740-2
Chlamydiae bacterium 3300020889-1
Chlamydiae bacterium 3300009084-136
Chlamydiae bacterium 3300009083-135
Candidatus Rubidus massiliensis
Chlamydiales bacterium 38-26
Neochlamydia sp. ACF65
Neochlamydia sp. TUME1
Neochlamydia sp. EPS4
Chlamydiae bacterium K940_chlam_8
Chlamydiae bacterium 3300009703-49
Chlamydiae bacterium 3300012931-71
Chlamydiae bacterium 1021942-14
Chlamydiae bacterium 3300006177-14
Chlamydiae bacterium RIFCSPLOWO2_02_FULL_45_22
Chlamydiae bacterium CG10_big_fil_rev_8_21_14_0_10_42_34
Chlamydiae bacterium SM23_39
Anoxychlamydiales bacterium K1060_chlam_5
Anoxychlamydiales bacterium K1000_chlam_1
Anoxychlamydiales bacterium KR126_chlam_6
Anoxychlamydiales bacterium K1060_chlam_1
Anoxychlamydiales bacterium KR126_chlam_4
Anoxychlamydiales bacterium KR126_chlam_5
Anoxychlamydiales bacterium K940_chlam_5
Chlamydiae bacterium 3300006056-139
Chlamydiae bacterium RIFCSPHIGHO2_12_FULL_49_11
Chlamydiae bacterium 3300013126-109
Chlamydiae bacterium 3300013133-59
Chlamydiae bacterium 3300013132-109
Chlamydiae bacterium 1063924-70
Chlamydiae bacterium 1045240-7
Simkania negevensis
Sygnamydia xenoturbellae
Neptunochlamydia vexilliferae
Syngnamydia medusae
Chlamydiae bacterium 3300006056-100
Chlamydiae bacterium K1060_chlam_2
Chlamydiae bacterium 1049244-12
Chlamydiae bacterium 3300004085-10
Chlamydiae bacterium 1045192-5
Chlamydiae bacterium KR126_chlam_1
Chlamydiae bacterium KR126_chlam_3
Chlamydiae bacterium K940_chlam_6
Chlamydiae bacterium 1096102-30
Chlamydiae bacterium RIFCSPLOWO2_02_FULL_49_12
Chlamydia sp. genome_bin_33
Chlamydiae bacterium 1159587-4
Chlamydiae bacterium 1095360-24
Chlamydiae bacterium 1021942-13
Chlamydiae bacterium 3300009709-46
Chlamydiae bacterium 3300020970-2
Chlamydiae bacterium 1064120-29
Chlamydiae bacterium 3300005529-103
Rhabdochlamydia helveticae
Rhabdochlamydia porcillionis
Rhabdochlamydia oedothoracis
0.1

chain3

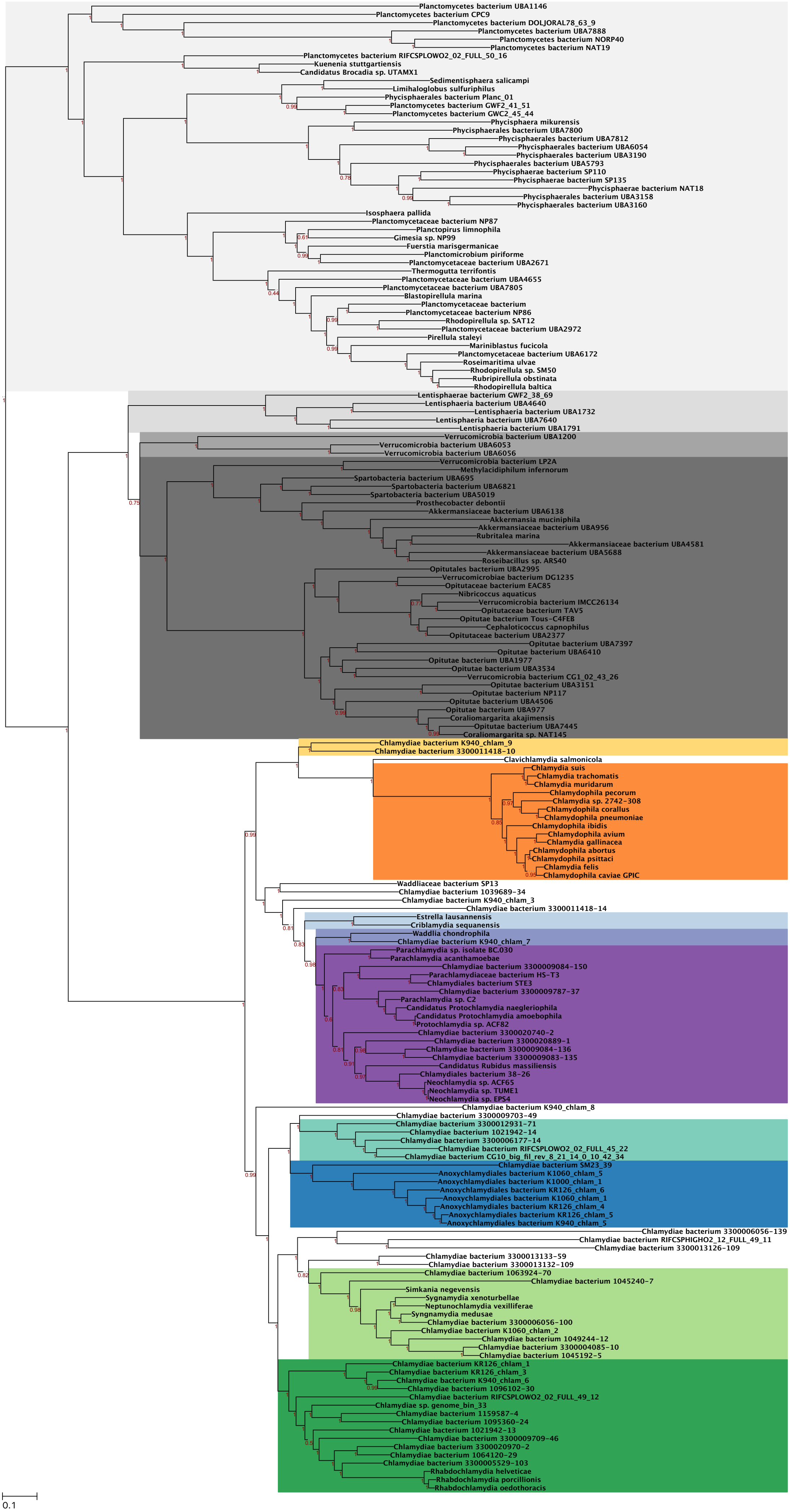
Planctomycetes bacterium UBA1146
Planctomycetes bacterium CPC9
Planctomycetes bacterium DOLJORAL78_63_9
Planctomycetes bacterium UBA7888
Planctomycetes bacterium NORP40
Planctomycetes bacterium NAT19
Planctomycetes bacterium RIFCSPLOWO2_02_FULL_50_16
Kuenenia stuttgartiensis
Candidatus Brocadia sp. UTAMX1
Sedimentisphaera salicampi
Limihaloglobus sulfuriphilus
Phycisphaerales bacterium Planc_01
Planctomycetes bacterium GWF2_41_51
Planctomycetes bacterium GWC2_45_44
Phycisphaera mikurensis
Phycisphaerales bacterium UBA7800
Phycisphaerales bacterium UBA7812
Phycisphaerales bacterium UBA6054
Phycisphaerales bacterium UBA3190
Phycisphaerales bacterium UBA5793
Phycisphaerae bacterium SP110
Phycisphaerae bacterium SP135
Phycisphaerae bacterium NAT18
Phycisphaerales bacterium UBA3158
Phycisphaerales bacterium UBA3160
Isosphaera pallida
Planctomycetaceae bacterium NP87
Planctopirus limnophila
Gimesia sp. NP99
Fuerstia marisgermanicae
Planctomicrobium piriforme
Planctomycetaceae bacterium UBA2671
Thermogutta terrifontis
Planctomycetaceae bacterium UBA4655
Planctomycetaceae bacterium UBA7805
Blastopirellula marina
Planctomycetaceae bacterium
Planctomycetaceae bacterium NP86
Rhodopirellula sp. SAT12
Planctomycetaceae bacterium UBA2972
Pirellula staleyi
Mariniblastus fucicola
Planctomycetaceae bacterium UBA6172
Roseimaritima ulvae
Rhodopirellula sp. SM50
Rubripirellula obstinata
Rhodopirellula baltica
Lentisphaerae bacterium GWF2_38_69
Lentisphaeria bacterium UBA4640
Lentisphaeria bacterium UBA1732
Lentisphaeria bacterium UBA7640
Lentisphaeria bacterium UBA1791
Verrucomicrobia bacterium UBA1200
Verrucomicrobia bacterium UBA6053
Verrucomicrobia bacterium UBA6056
Verrucomicrobia bacterium LP2A
Methylacidiphilum infernorum
Spartobacteria bacterium UBA695
Spartobacteria bacterium UBA6821
Spartobacteria bacterium UBA5019
Prosthecobacter debontii
Akkermansiaceae bacterium UBA6138
Akkermansia muciniphila
Akkermansiaceae bacterium UBA956
Rubritalea marina
Akkermansiaceae bacterium UBA4581
Akkermansiaceae bacterium UBA5688
Roseibacillus sp. ARS40
Opitutales bacterium UBA2995
Verrucomicrobiae bacterium DG1235
Opitutaceae bacterium EAC85
Nibricoccus aquaticus
Verrucomicrobia bacterium IMCC26134
Opitutaceae bacterium TAV5
Opitutae bacterium Tous-C4FEB
Cephaloticoccus capnophilus
Opitutaceae bacterium UBA2377
Opitutae bacterium UBA7397
Opitutae bacterium UBA6410
Opitutae bacterium UBA1977
Opitutae bacterium UBA3534
Verrucomicrobia bacterium CG1_02_43_26
Opitutae bacterium UBA3151
Opitutae bacterium NP117
Opitutae bacterium UBA4506
Opitutae bacterium UBA977
Coraliomargarita akajimensis
Opitutae bacterium UBA7445
Coraliomargarita sp. NAT145
Chlamydiae bacterium K940_chlam_9
Chlamydiae bacterium 3300011418-10
Clavichlamydia salmonicola
Chlamydia suis
Chlamydia trachomatis
Chlamydia muridarum
Chlamydophila pecorum
Chlamydia sp. 2742-308
Chlamydophila corallus
Chlamydophila pneumoniae
Chlamydophila ibidis
Chlamydophila avium
Chlamydia gallinacea
Chlamydophila abortus
Chlamydophila psittaci
Chlamydia felis
Chlamydophila caviae GPIC
Waddliaceae bacterium SP13
Chlamydiae bacterium 1039689-34
Chlamydiae bacterium K940_chlam_3
Chlamydiae bacterium 3300011418-14
Estrella lausannensis
Criblamydia sequanensis
Waddlia chondrophila
Chlamydiae bacterium K940_chlam_7
Parachlamydia sp. isolate BC.030
Parachlamydia acanthamoebae
Chlamydiae bacterium 3300009084-150
Parachlamydiaceae bacterium HS-T3
Chlamydiales bacterium STE3
Chlamydiae bacterium 3300009787-37
Parachlamydia sp. C2
Candidatus Protochlamydia naegleriophila
Candidatus Protochlamydia amoebophila
Protochlamydia sp. ACF82
Chlamydiae bacterium 3300020740-2
Chlamydiae bacterium 3300020889-1
Chlamydiae bacterium 3300009084-136
Chlamydiae bacterium 3300009083-135
Candidatus Rubidus massiliensis
Chlamydiales bacterium 38-26
Neochlamydia sp. ACF65
Neochlamydia sp. TUME1
Neochlamydia sp. EPS4
Chlamydiae bacterium K940_chlam_8
Chlamydiae bacterium 3300009703-49
Chlamydiae bacterium 3300012931-71
Chlamydiae bacterium 1021942-14
Chlamydiae bacterium 3300006177-14
Chlamydiae bacterium RIFCSPLOWO2_02_FULL_45_22
Chlamydiae bacterium CG10_big_fil_rev_8_21_14_0_10_42_34
Chlamydiae bacterium SM23_39
Anoxychlamydiales bacterium K1060_chlam_5
Anoxychlamydiales bacterium K1000_chlam_1
Anoxychlamydiales bacterium KR126_chlam_6
Anoxychlamydiales bacterium K1060_chlam_1
Anoxychlamydiales bacterium KR126_chlam_4
Anoxychlamydiales bacterium KR126_chlam_5
Anoxychlamydiales bacterium K940_chlam_5
Chlamydiae bacterium 3300006056-139
Chlamydiae bacterium RIFCSPHIGHO2_12_FULL_49_11
Chlamydiae bacterium 3300013126-109
Chlamydiae bacterium 3300013133-59
Chlamydiae bacterium 3300013132-109
Chlamydiae bacterium 1063924-70
Chlamydiae bacterium 1045240-7
Simkania negevensis
Sygnamydia xenoturbellae
Neptunochlamydia vexilliferae
Syngnamydia medusae
Chlamydiae bacterium 3300006056-100
Chlamydiae bacterium K1060_chlam_2
Chlamydiae bacterium 1049244-12
Chlamydiae bacterium 3300004085-10
Chlamydiae bacterium 1045192-5
Chlamydiae bacterium KR126_chlam_1
Chlamydiae bacterium KR126_chlam_3
Chlamydiae bacterium K940_chlam_6
Chlamydiae bacterium 1096102-30
Chlamydiae bacterium RIFCSPLOWO2_02_FULL_49_12
Chlamydiae sp. genome_bin_33
Chlamydiae bacterium 1159587-4
Chlamydiae bacterium 1095360-24
Chlamydiae bacterium 1021942-13
Chlamydiae bacterium 3300009709-46
Chlamydiae bacterium 3300020970-2
Chlamydiae bacterium 1064120-29
Chlamydiae bacterium 3300005529-103
Rhabdochlamydia helveticae
Rhabdochlamydia porcillionis
Rhabdochlamydia oedothoracis
0.1

chain4

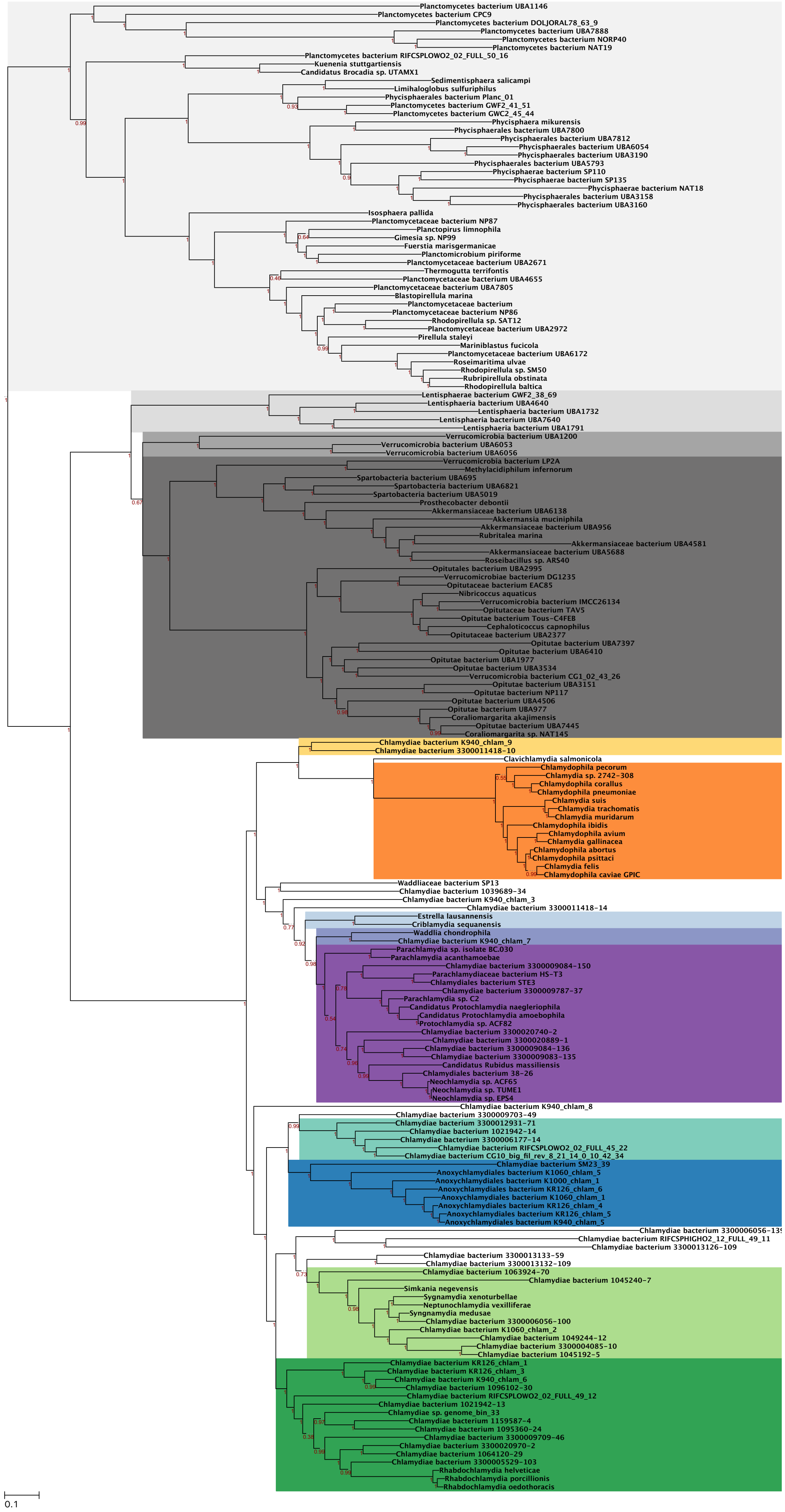
Planctomycetes bacterium UBA1146
Planctomycetes bacterium CPC9
Planctomycetes bacterium DOLJORAL78_63_9
Planctomycetes bacterium UBA7888
Planctomycetes bacterium NORP40
Planctomycetes bacterium NAT19
Planctomycetes bacterium RIFCSPLOWO2_02_FULL_50_16
Kuenenia stuttgartiensis
Candidatus Brocadia sp. UTAMX1
Sedimentisphaera salicampi
Limihaloglobus sulfuriphilus
Phycisphaerales bacterium Planc_01
Planctomycetes bacterium GWF2_41_51
Planctomycetes bacterium GWC2_45_44
Phycisphaera mikurensis
Phycisphaerales bacterium UBA7800
Phycisphaerales bacterium UBA7812
Phycisphaerales bacterium UBA6054
Phycisphaerales bacterium UBA3190
Phycisphaerales bacterium UBA5793
Phycisphaerae bacterium SP110
Phycisphaerae bacterium SP135
Phycisphaerae bacterium NAT18
Phycisphaerales bacterium UBA3158
Phycisphaerales bacterium UBA3160
Isosphaera pallida
Planctomycetaceae bacterium NP87
Planctopirus limnophila
Gimesia sp. NP99
Fuerstia marisgermanicae
Planctomicrobium piriforme
Planctomycetaceae bacterium UBA2671
Thermogutta terrifontis
Planctomycetaceae bacterium UBA4655
Planctomycetaceae bacterium UBA7805
Blastopirellula marina
Planctomycetaceae bacterium
Planctomycetaceae bacterium NP86
Rhodopirellula sp. SAT12
Planctomycetaceae bacterium UBA2972
Pirellula staleyi
Mariniblastus fucicola
Planctomycetaceae bacterium UBA6172
Roseimaritima ulvae
Rhodopirellula sp. SM50
Rubripirellula obstinata
Rhodopirellula baltica
Lentisphaerae bacterium GWF2_38_69
Lentisphaeria bacterium UBA4640
Lentisphaeria bacterium UBA1732
Lentisphaeria bacterium UBA7640
Lentisphaeria bacterium UBA1791
Verrucomicrobia bacterium UBA1200
Verrucomicrobia bacterium UBA6053
Verrucomicrobia bacterium UBA6056
Verrucomicrobia bacterium LP2A
Methylacidiphilum infernorum
Spartobacteria bacterium UBA695
Spartobacteria bacterium UBA6821
Spartobacteria bacterium UBA5019
Prosthecobacter debontii
Akkermansiaceae bacterium UBA6138
Akkermansia muciniphila
Akkermansiaceae bacterium UBA956
Rubritalea marina
Akkermansiaceae bacterium UBA4581
Akkermansiaceae bacterium UBA5688
Roseibacillus sp. ARS40
Opitutales bacterium UBA2995
Verrucomicrobiae bacterium DG1235
Opitutaceae bacterium EAC85
Nibricoccus aquaticus
Verrucomicrobia bacterium IMCC26134
Opitutaceae bacterium TAV5
Opitutae bacterium Tous-C4FEB
Cephaloticoccus capnophilus
Opitutaceae bacterium UBA2377
Opitutae bacterium UBA7397
Opitutae bacterium UBA6410
Opitutae bacterium UBA1977
Opitutae bacterium UBA3534
Verrucomicrobia bacterium CG1_02_43_26
Opitutae bacterium UBA3151
Opitutae bacterium NP117
Opitutae bacterium UBA4506
Opitutae bacterium UBA977
Coraliomargarita akajimensis
Opitutae bacterium UBA7445
Coraliomargarita sp. NAT145
Chlamydiae bacterium K940_chlam_9
Chlamydiae bacterium 3300011418-10
Clavichlamydia salmonicola
Chlamydophila pecorum
Chlamydia sp. 2742-308
Chlamydophila corallus
Chlamydophila pneumoniae
Chlamydia suis
Chlamydia trachomatis
Chlamydia muridarum
Chlamydophila ibidis
Chlamydophila avium
Chlamydia gallinacea
Chlamydophila abortus
Chlamydophila psittaci
Chlamydia felis
Chlamydophila caviae GPIC
Waddliaceae bacterium SP13
Chlamydiae bacterium 1039689-34
Chlamydiae bacterium K940_chlam_3
Chlamydiae bacterium 3300011418-14
Estrella lausannensis
Criblamydia sequanensis
Waddlia chondrophila
Chlamydiae bacterium K940_chlam_7
Parachlamydia sp. isolate BC.030
Parachlamydia acanthamoebae
Chlamydiae bacterium 3300009084-150
Parachlamydiaceae bacterium HS-T3
Chlamydiales bacterium STE3
Chlamydiae bacterium 3300009787-37
Parachlamydia sp. C2
Candidatus Protochlamydia naegleriophila
Candidatus Protochlamydia amoebophila
Protochlamydia sp. ACF82
Chlamydiae bacterium 3300020740-2
Chlamydiae bacterium 3300020889-1
Chlamydiae bacterium 3300009084-136
Chlamydiae bacterium 3300009083-135
Candidatus Rubidus massiliensis
Chlamydiales bacterium 38-26
Neochlamydia sp. ACF65
Neochlamydia sp. TUME1
Neochlamydia sp. EPS4
Chlamydiae bacterium K940_chlam_8
Chlamydiae bacterium 3300009703-49
Chlamydiae bacterium 3300012931-71
Chlamydiae bacterium 1021942-14
Chlamydiae bacterium 3300006177-14
Chlamydiae bacterium RIFCSPLOWO2_02_FULL_45_22
Chlamydiae bacterium CG10_big_fil_rev_8_21_14_0_10_42_34
Chlamydiae bacterium SM23_39
Anoxychlamydiales bacterium K1060_chlam_5
Anoxychlamydiales bacterium K1000_chlam_1
Anoxychlamydiales bacterium KR126_chlam_6
Anoxychlamydiales bacterium K1060_chlam_1
Anoxychlamydiales bacterium KR126_chlam_4
Anoxychlamydiales bacterium KR126_chlam_5
Anoxychlamydiales bacterium K940_chlam_5
Chlamydiae bacterium 3300006056-13
Chlamydiae bacterium RIFCSPHIGHO2_12_FULL_49_11
Chlamydiae bacterium 3300013126-109
Chlamydiae bacterium 3300013133-59
Chlamydiae bacterium 3300013132-109
Chlamydiae bacterium 1063924-70
Chlamydiae bacterium 1045240-7
Simkania negevensis
Sygnamydia xenoturbellae
Neptunochlamydia vexilliferae
Syngnamydia medusae
Chlamydiae bacterium 3300006056-100
Chlamydiae bacterium K1060_chlam_2
Chlamydiae bacterium 1049244-12
Chlamydiae bacterium 3300004085-10
Chlamydiae bacterium 1045192-5
Chlamydiae bacterium KR126_chlam_1
Chlamydiae bacterium KR126_chlam_3
Chlamydiae bacterium K940_chlam_6
Chlamydiae bacterium 1096102-30
Chlamydiae bacterium RIFCSPLOWO2_02_FULL_49_12
Chlamydiae bacterium 1021942-13
Chlamydiae sp. genome_bin_33
Chlamydiae bacterium 1159587-4
Chlamydiae bacterium 1095360-24
Chlamydiae bacterium 3300009709-46
Chlamydiae bacterium 3300020970-2
Chlamydiae bacterium 1064120-29
Chlamydiae bacterium 3300005529-103
Rhabdochlamydia helveticae
Rhabdochlamydia porcellionis
Rhabdochlamydia oedothoracis
0.1

convergence_chains_1234

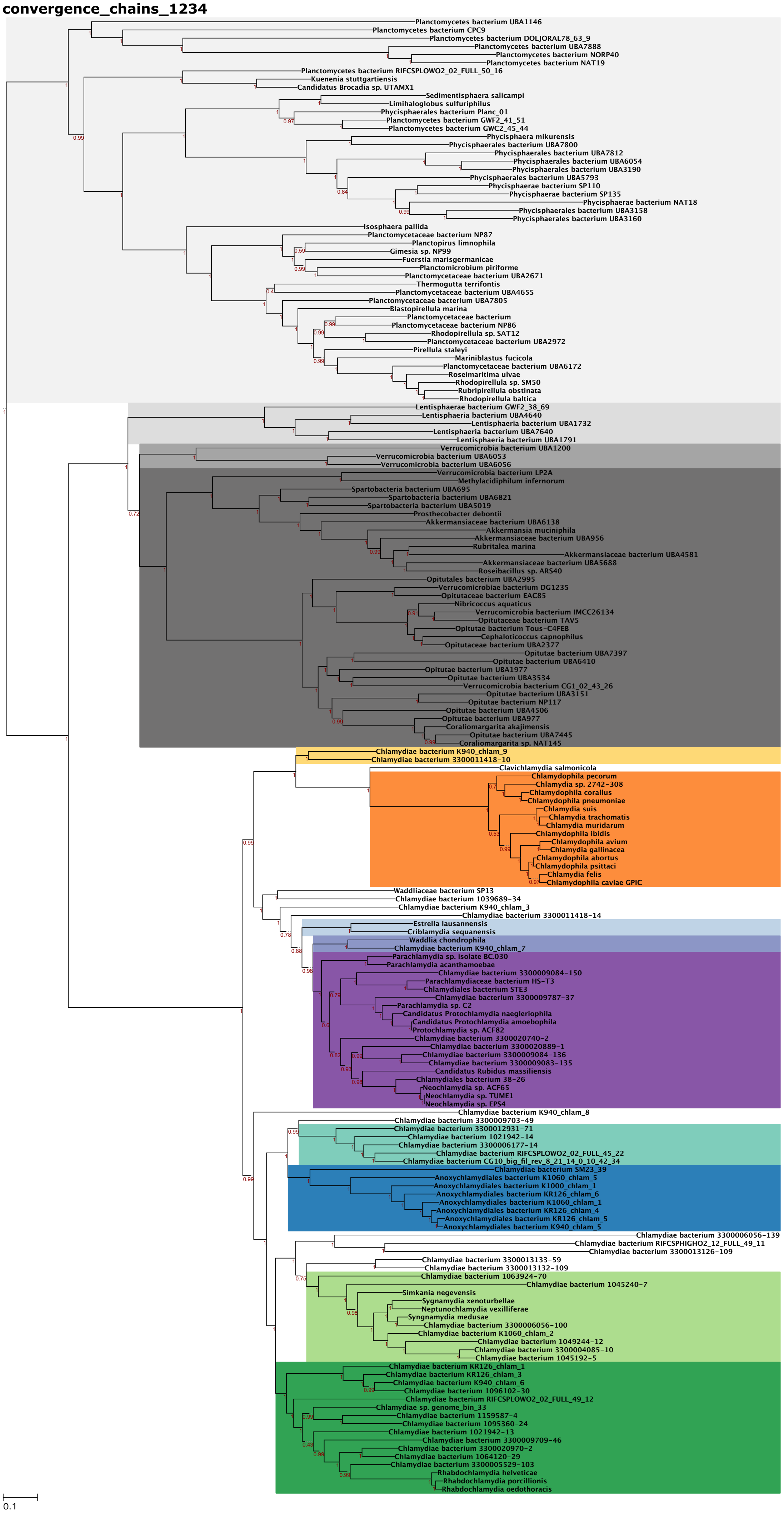

convergence_chains_13

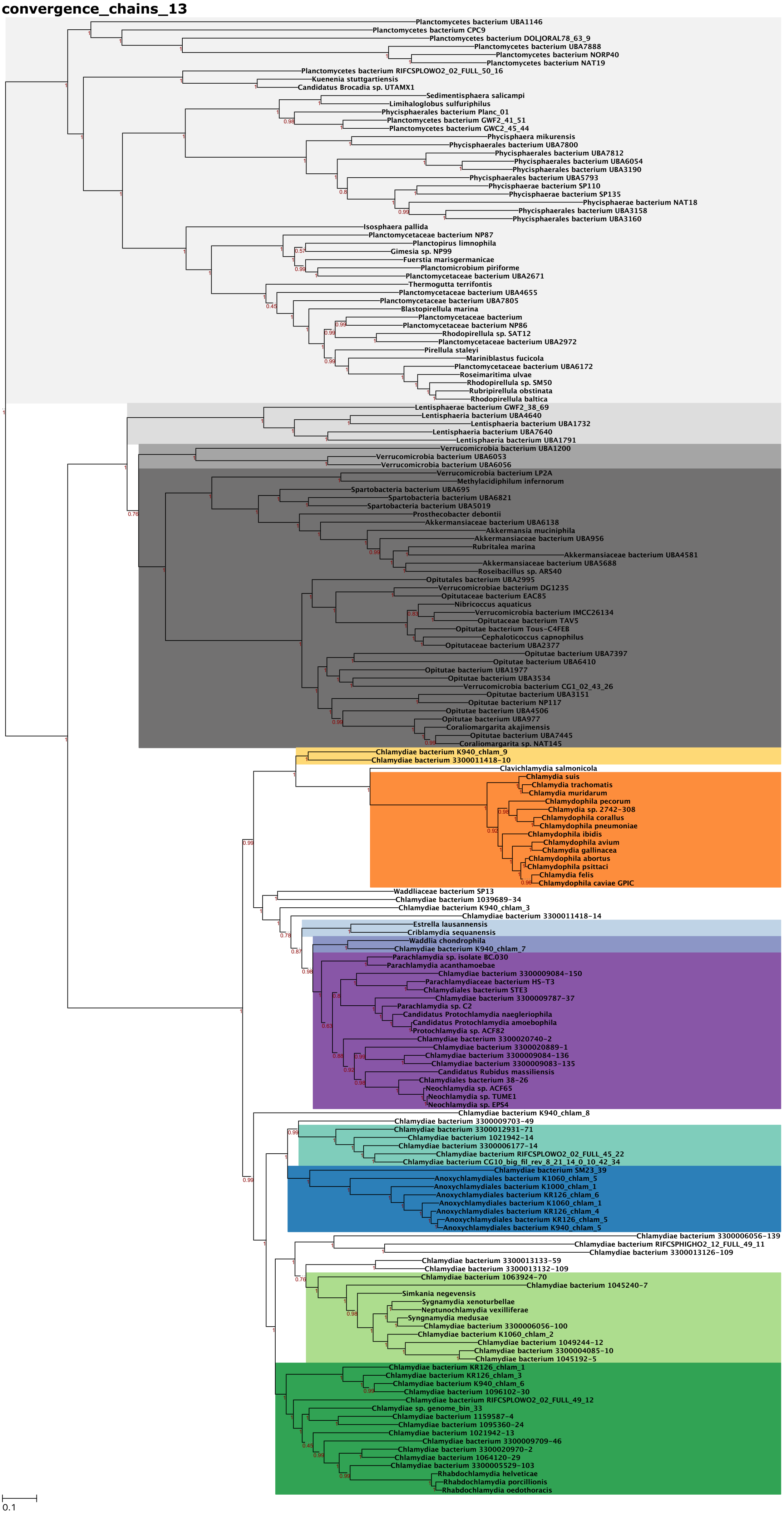

convergence_chains_24

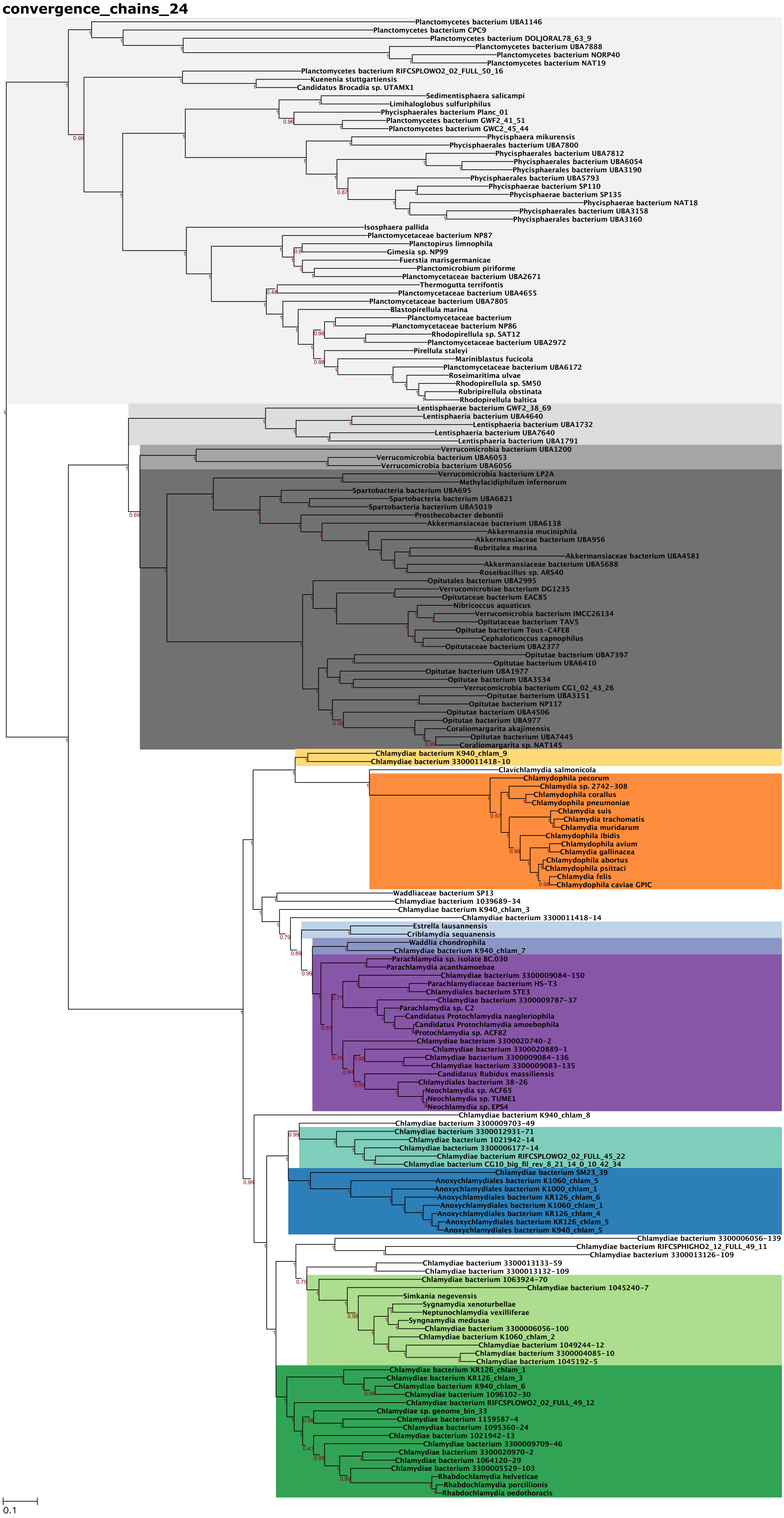

183taxa_0perc_pruned_original

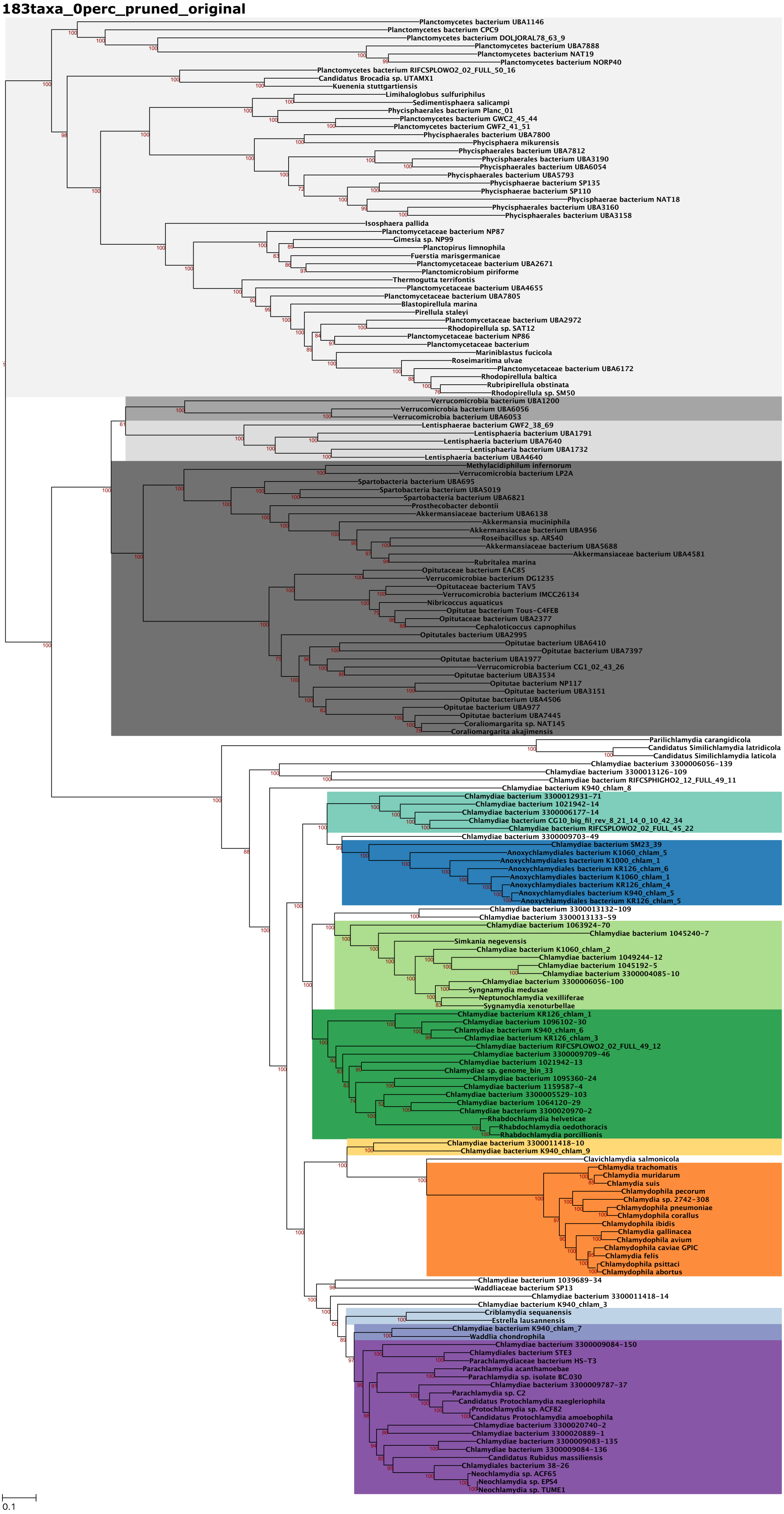

183taxa_0perc_pruned_original

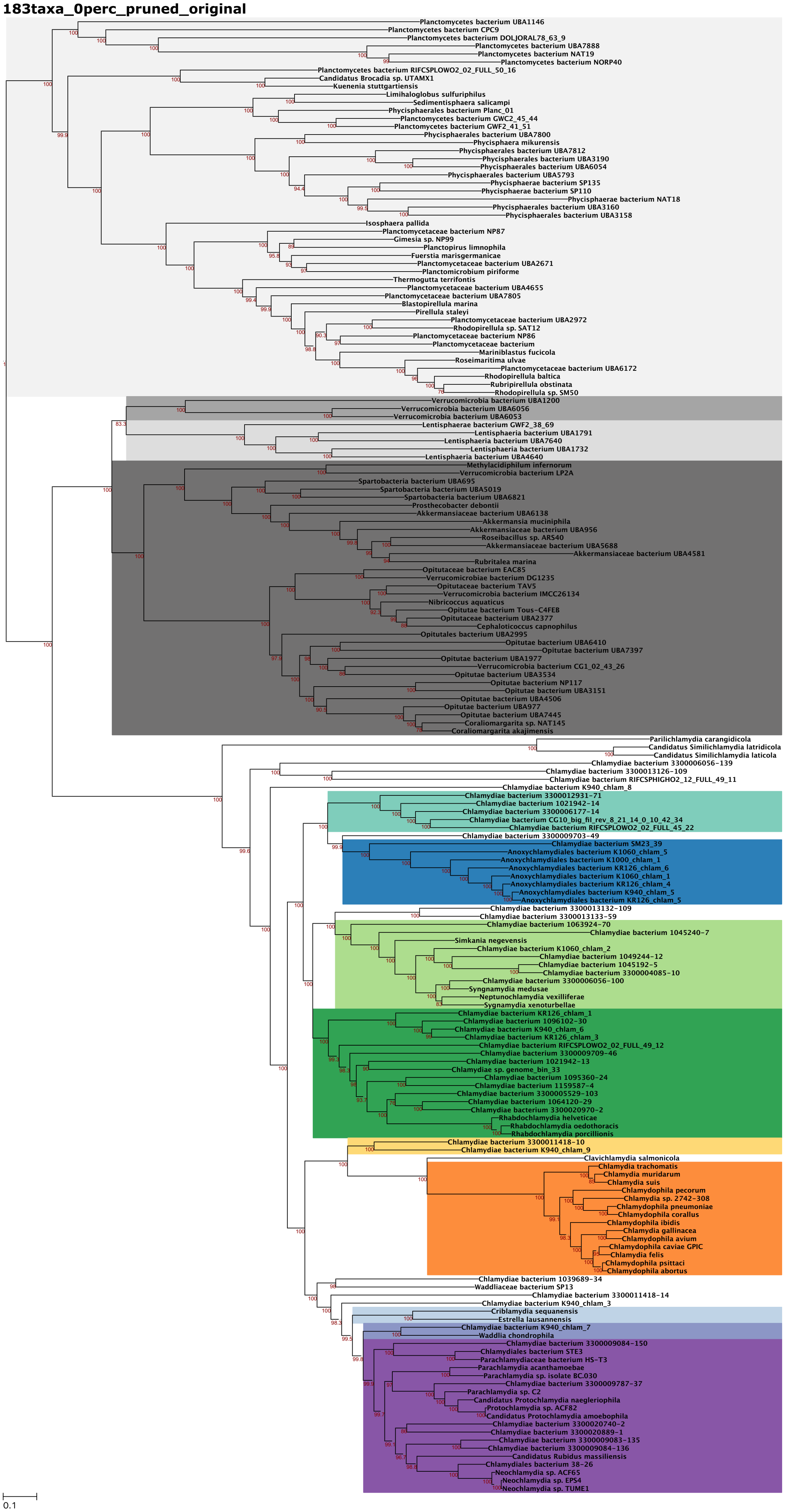

chain1

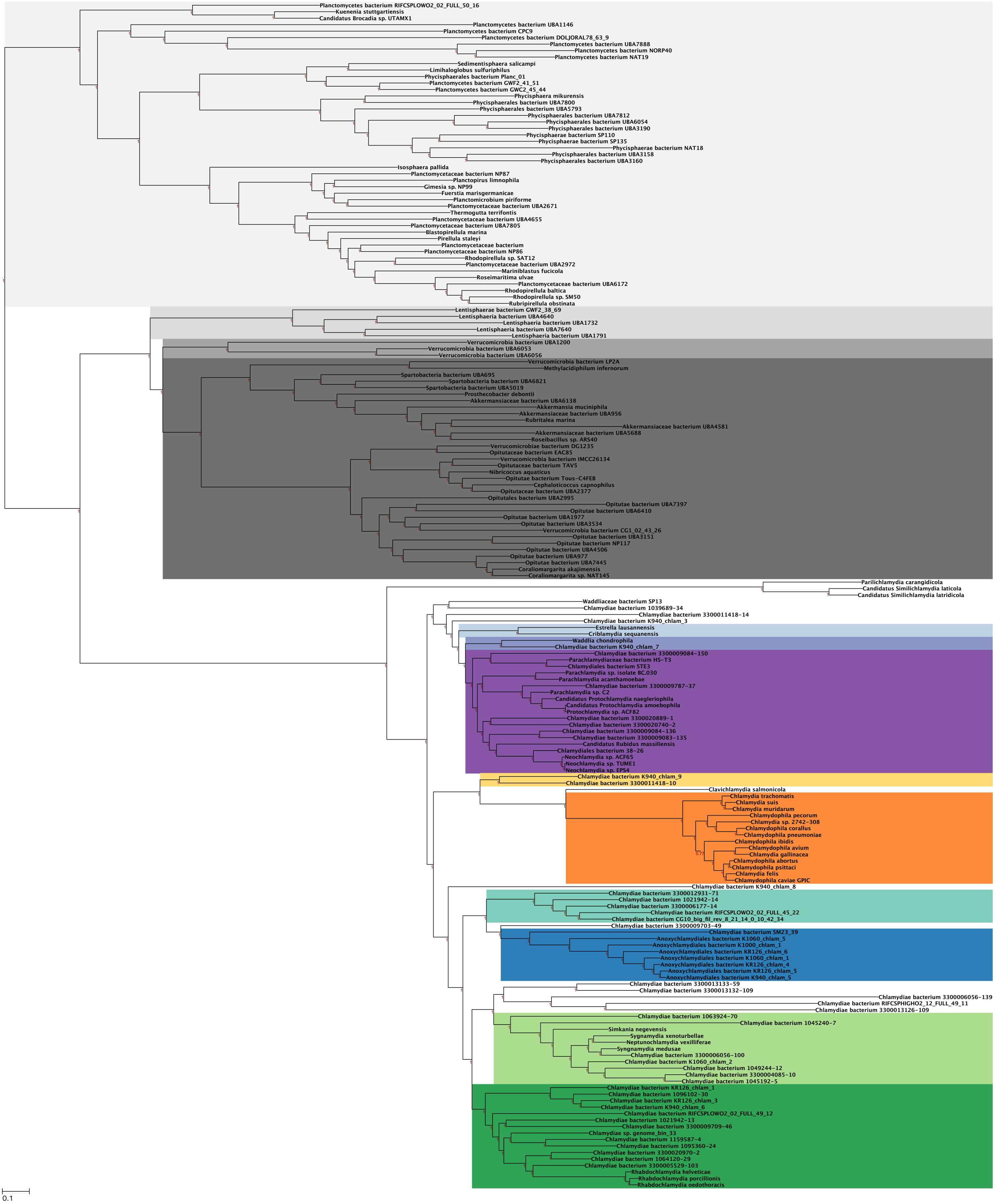
Planctomycetes bacterium RIFCSPLOWO2_02_FULL_50_16
Kuenenia stuttgartiensis
Candidatus Brocadia sp. UTAMX1
Planctomycetes bacterium UBA1146
Planctomycetes bacterium CPC9
Planctomycetes bacterium DOLJORAL78_63_9
Planctomycetes bacterium UBA7888
Planctomycetes bacterium NORP40
Planctomycetes bacterium NAT19
Sedimentisphaera salicampi
Limihaloglobus sulfuriphilus
Phycisphaerales bacterium Planc_01
Planctomycetes bacterium GWF2_41_51
Planctomycetes bacterium GWC2_45_44
Phycisphaera mikurensis
Phycisphaerales bacterium UBA7800
Phycisphaerales bacterium UBA5793
Phycisphaerales bacterium UBA7812
Phycisphaerales bacterium UBA6054
Phycisphaerales bacterium UBA3190
Phycisphaerae bacterium SP110
Phycisphaerae bacterium SP135
Phycisphaerae bacterium NAT18
Phycisphaerales bacterium UBA3158
Phycisphaerales bacterium UBA3160
Isosphaera pallida
Planctomycetaceae bacterium NP87
Planctopirus limnophila
Gimesia sp. NP99
Fuerstia marisgermanicae
Planctomicrobium piriforme
Planctomycetaceae bacterium UBA2671
Thermogutta terrifontis
Planctomycetaceae bacterium UBA4655
Planctomycetaceae bacterium UBA7805
Blastopirellula marina
Pirellula staleyi
Planctomycetaceae bacterium
Planctomycetaceae bacterium NP86
Rhodopirellula sp. SAT12
Planctomycetaceae bacterium UBA2972
Mariniblastus fucicola
Roseimaritima ulvae
Planctomycetaceae bacterium UBA6172
Rhodopirellula baltica
Rhodopirellula sp. SM50
Rubripirellula obstinata
Lentisphaerae bacterium GWF2_38_69
Lentisphaeria bacterium UBA4640
Lentisphaeria bacterium UBA1732
Lentisphaeria bacterium UBA7640
Lentisphaeria bacterium UBA1791
Verrucomicrobia bacterium UBA1200
Verrucomicrobia bacterium UBA6053
Verrucomicrobia bacterium UBA6056
Verrucomicrobia bacterium LP2A
Methylacidiphilum infernorum
Spartobacteria bacterium UBA695
Spartobacteria bacterium UBA6821
Spartobacteria bacterium UBA5019
Prosthecobacter debontii
Akkermansiaceae bacterium UBA6138
Akkermansia muciniphila
Akkermansiaceae bacterium UBA956
Rubritalea marina
Akkermansiaceae bacterium UBA4581
Akkermansiaceae bacterium UBA5688
Roseibacillus sp. ARS40
Verrucomicrobiae bacterium DG1235
Opitutaceae bacterium EAC85
Verrucomicrobia bacterium IMCC26134
Opitutaceae bacterium TAV5
Nibricoccus aquaticus
Opitutae bacterium Tous-C4FEB
Cephaloticoccus capnophilus
Opitutaceae bacterium UBA2377
Opitutales bacterium UBA2995
Opitutae bacterium UBA7397
Opitutae bacterium UBA6410
Opitutae bacterium UBA1977
Opitutae bacterium UBA3534
Verrucomicrobia bacterium CG1_02_43_26
Opitutae bacterium UBA3151
Opitutae bacterium NP117
Opitutae bacterium UBA4506
Opitutae bacterium UBA977
Opitutae bacterium UBA7445
Coraliomargarita akajimensis
Coraliomargarita sp. NAT145
Parilichlamydia carangidicola
Candidatus Similichlamydia laticola
Candidatus Similichlamydia latridicola
Waddliaceae bacterium SP13
Chlamydiae bacterium 1039689-34
Chlamydiae bacterium 3300011418-14
Chlamydiae bacterium K940_chlam_3
Estrella lausannensis
Criblamydia sequanensis
Waddlia chondrophila
Chlamydiae bacterium K940_chlam_7
Chlamydiae bacterium 3300009084-150
Parachlamydiaceae bacterium HS-T3
Chlamydiales bacterium STE3
Parachlamydia sp. isolate BC.030
Parachlamydia acanthamoebae
Chlamydiae bacterium 3300009787-37
Parachlamydia sp. C2
Candidatus Protochlamydia naegleriophila
Candidatus Protochlamydia amoebophila
Protochlamydia sp. ACF82
Chlamydiae bacterium 3300020889-1
Chlamydiae bacterium 3300020740-2
Chlamydiae bacterium 3300009084-136
Chlamydiae bacterium 3300009083-135
Candidatus Rubidus massiliensis
Chlamydiales bacterium 38-26
Neochlamydia sp. ACF65
Neochlamydia sp. TUME1
Neochlamydia sp. EPS4
Chlamydiae bacterium K940_chlam_9
Chlamydiae bacterium 3300011418-10
Clavichlamydia salmonicola
Chlamydia trachomatis
Chlamydia suis
Chlamydia muridarum
Chlamydophila pecorum
Chlamydia sp. 2742-308
Chlamydophila corallus
Chlamydophila pneumoniae
Chlamydophila ibidis
Chlamydophila avium
Chlamydia gallinacea
Chlamydophila abortus
Chlamydophila psittaci
Chlamydia felis
Chlamydophila caviae GPIC
Chlamydiae bacterium K940_chlam_8
Chlamydiae bacterium 3300012931-71
Chlamydiae bacterium 1021942-14
Chlamydiae bacterium 3300006177-14
Chlamydiae bacterium RIFCSPLOWO2_02_FULL_45_22
Chlamydiae bacterium CG10_big_fil_rev_8_21_14_0_10_42_34
Chlamydiae bacterium 3300009703-49
Chlamydiae bacterium SM23_39
Anoxychlamydiales bacterium K1060_chlam_5
Anoxychlamydiales bacterium K1000_chlam_1
Anoxychlamydiales bacterium KR126_chlam_6
Anoxychlamydiales bacterium K1060_chlam_1
Anoxychlamydiales bacterium KR126_chlam_4
Anoxychlamydiales bacterium KR126_chlam_5
Anoxychlamydiales bacterium K940_chlam_5
Chlamydiae bacterium 3300013133-59
Chlamydiae bacterium 3300013132-109
Chlamydiae bacterium 3300006056-139
Chlamydiae bacterium RIFCSPHIGHO2_12_FULL_49_11
Chlamydiae bacterium 3300013126-109
Chlamydiae bacterium 1063924-70
Chlamydiae bacterium 1045240-7
Simkania negevensis
Sygnamydia xenoturbellae
Neptunochlamydia vexilliferae
Syngnamydia medusae
Chlamydiae bacterium 3300006056-100
Chlamydiae bacterium K1060_chlam_2
Chlamydiae bacterium 1049244-12
Chlamydiae bacterium 3300004085-10
Chlamydiae bacterium 1045192-5
Chlamydiae bacterium KR126_chlam_1
Chlamydiae bacterium 1096102-30
Chlamydiae bacterium KR126_chlam_3
Chlamydiae bacterium K940_chlam_6
Chlamydiae bacterium RIFCSPLOWO2_02_FULL_49_12
Chlamydiae bacterium 1021942-13
Chlamydiae bacterium 3300009709-46
Chlamydiae sp. genome_bin_33
Chlamydiae bacterium 1159587-4
Chlamydiae bacterium 1095360-24
Chlamydiae bacterium 3300020970-2
Chlamydiae bacterium 1064120-29
Chlamydiae bacterium 3300005529-103
Rhabdochlamydia helveticae
Rhabdochlamydia porcillionis
Rhabdochlamydia oedothoracis
0.1

chain2

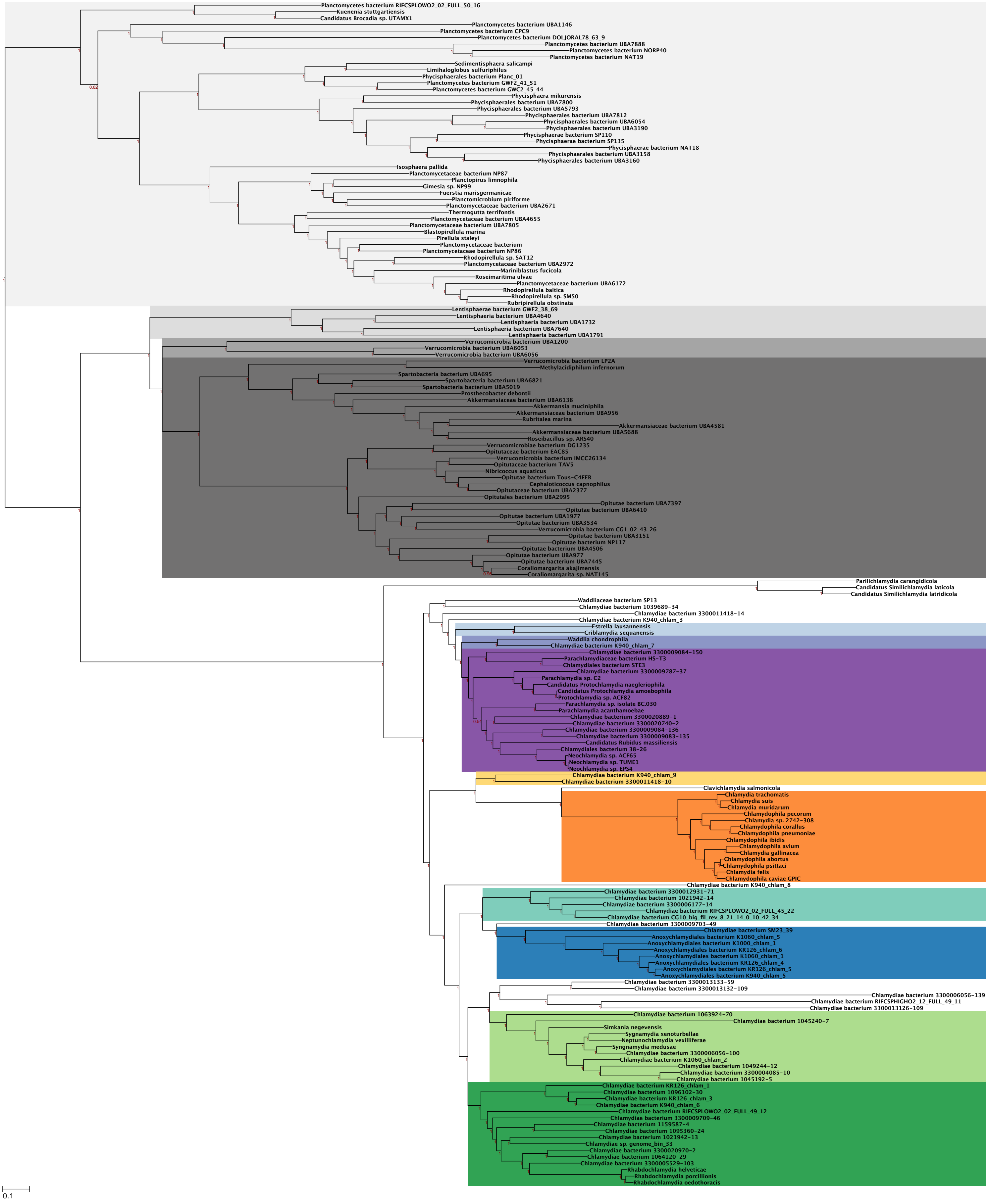

Planctomycetes bacterium RIFCSPLOWO2_02_FULL_50_16
Kuenenia stuttgartiensis
Candidatus Brocadia sp. UTAMX1
Planctomycetes bacterium UBA1146
Planctomycetes bacterium CPC9
Planctomycetes bacterium DOLJORAL78_63_9
Planctomycetes bacterium UBA7888
Planctomycetes bacterium NORP40
Planctomycetes bacterium NAT19
Sedimentisphaera salicampi
Limihaloglobus sulfuriphilus
Phycisphaerales bacterium Planc_01
Planctomycetes bacterium GWF2_41_51
Planctomycetes bacterium GWC2_45_44
Phycisphaera mikurensis
Phycisphaerales bacterium UBA7800
Phycisphaerales bacterium UBA5793
Phycisphaerales bacterium UBA7812
Phycisphaerales bacterium UBA6054
Phycisphaerales bacterium UBA3190
Phycisphaerae bacterium SP110
Phycisphaerae bacterium SP135
Phycisphaerae bacterium NAT18
Phycisphaerales bacterium UBA3158
Phycisphaerales bacterium UBA3160
Isosphaera pallida
Planctomycetaceae bacterium NP87
Planctopirus limnophila
Gimesia sp. NP99
Fuerstia marisgermanicae
Planctomicrobium piriforme
Planctomycetaceae bacterium UBA2671
Thermogutta terrifontis
Planctomycetaceae bacterium UBA4655
Planctomycetaceae bacterium UBA7805
Blastopirellula marina
Pirellula staleyi
Planctomycetaceae bacterium
Planctomycetaceae bacterium NP86
Rhodopirellula sp. SAT12
Planctomycetaceae bacterium UBA2972
Mariniblastus fucicola
Roseimaritima ulvae
Planctomycetaceae bacterium UBA6172
Rhodopirellula baltica
Rhodopirellula sp. SM50
Rubripirellula obstinata
Lentisphaerae bacterium GWF2_38_69
Lentisphaeria bacterium UBA4640
Lentisphaeria bacterium UBA1732
Lentisphaeria bacterium UBA7640
Lentisphaeria bacterium UBA1791
Verrucomicrobia bacterium UBA1200
Verrucomicrobia bacterium UBA6053
Verrucomicrobia bacterium UBA6056
Verrucomicrobia bacterium LP2A
Methylacidiphilum infernorum
Spartobacteria bacterium UBA695
Spartobacteria bacterium UBA6821
Spartobacteria bacterium UBA5019
Prosthecobacter debontii
Akkermansiaceae bacterium UBA6138
Akkermansia muciniphila
Akkermansiaceae bacterium UBA956
Rubritalea marina
Akkermansiaceae bacterium UBA4581
Akkermansiaceae bacterium UBA5688
Roseibacillus sp. ARS40
Verrucomicrobiae bacterium DG1235
Opitutaceae bacterium EAC85
Verrucomicrobia bacterium IMCC26134
Opitutaceae bacterium TAV5
Nibricoccus aquaticus
Opitutae bacterium Tous-C4FEB
Cephaloticoccus capnophilus
Opitutaceae bacterium UBA2377
Opitutales bacterium UBA2995
Opitutae bacterium UBA7397
Opitutae bacterium UBA6410
Opitutae bacterium UBA1977
Opitutae bacterium UBA3534
Verrucomicrobia bacterium CG1_02_43_26
Opitutae bacterium UBA3151
Opitutae bacterium NP117
Opitutae bacterium UBA4506
Opitutae bacterium UBA977
Opitutae bacterium UBA7445
Coraliomargarita akajimensis
Coraliomargarita sp. NAT145
Parilichlamydia carangidicola
Candidatus Similichlamydia laticola
Candidatus Similichlamydia latridicola
Waddliaceae bacterium SP13
Chlamydiae bacterium 1039689-34
Chlamydiae bacterium 3300011418-14
Chlamydiae bacterium K940_chlam_3
Estrella lausannensis
Criblamydia sequanensis
Waddlia chondrophila
Chlamydiae bacterium K940_chlam_7
Chlamydiae bacterium 3300009084-150
Parachlamydiaceae bacterium HS-T3
Chlamydiales bacterium STE3
Chlamydiae bacterium 3300009787-37
Parachlamydia sp. C2
Candidatus Protochlamydia naegleriophila
Candidatus Protochlamydia amoebophila
Protochlamydia sp. ACF82
Parachlamydia sp. isolate BC.030
Parachlamydia acanthamoebae
Chlamydiae bacterium 3300020889-1
Chlamydiae bacterium 3300020740-2
Chlamydiae bacterium 3300009084-136
Chlamydiae bacterium 3300009083-135
Candidatus Rubidus massiliensis
Chlamydiales bacterium 38-26
Neochlamydia sp. ACF65
Neochlamydia sp. TUME1
Neochlamydia sp. EPS4
Chlamydiae bacterium K940_chlam_9
Chlamydiae bacterium 3300011418-10
Clavichlamydia salmonicola
Chlamydia trachomatis
Chlamydia suis
Chlamydia muridarum
Chlamydophila pecorum
Chlamydia sp. 2742-308
Chlamydophila corallus
Chlamydophila pneumoniae
Chlamydophila ibidis
Chlamydophila avium
Chlamydia gallinacea
Chlamydophila abortus
Chlamydophila psittaci
Chlamydia felis
Chlamydophila caviae GPIC
Chlamydiae bacterium K940_chlam_8
Chlamydiae bacterium 3300012931-71
Chlamydiae bacterium 1021942-14
Chlamydiae bacterium 3300006177-14
Chlamydiae bacterium RIFCSPLOWO2_02_FULL_45_22
Chlamydiae bacterium CG10_big_fil_rev_8_21_14_0_10_42_34
Chlamydiae bacterium 3300009703-49
Chlamydiae bacterium SM23_39
Anoxychlamydiales bacterium K1060_chlam_5
Anoxychlamydiales bacterium K1000_chlam_1
Anoxychlamydiales bacterium KR126_chlam_6
Anoxychlamydiales bacterium K1060_chlam_1
Anoxychlamydiales bacterium KR126_chlam_4
Anoxychlamydiales bacterium KR126_chlam_5
Anoxychlamydiales bacterium K940_chlam_5
Chlamydiae bacterium 3300013133-59
Chlamydiae bacterium 3300013132-109
Chlamydiae bacterium 3300006056-139
Chlamydiae bacterium RIFCSPHIGHO2_12_FULL_49_11
Chlamydiae bacterium 3300013126-109
Chlamydiae bacterium 1063924-70
Chlamydiae bacterium 1045240-7
Simkania negevensis
Syngnamydia xenoturbellae
Neptunochlamydia vexilliferae
Syngnamydia medusae
Chlamydiae bacterium 3300006056-100
Chlamydiae bacterium K1060_chlam_2
Chlamydiae bacterium 1049244-12
Chlamydiae bacterium 3300004085-10
Chlamydiae bacterium 1045192-5
Chlamydiae bacterium KR126_chlam_1
Chlamydiae bacterium 1096102-30
Chlamydiae bacterium KR126_chlam_3
Chlamydiae bacterium K940_chlam_6
Chlamydiae bacterium RIFCSPLOWO2_02_FULL_49_12
Chlamydiae bacterium 3300009709-46
Chlamydiae bacterium 1159587-4
Chlamydiae bacterium 1095360-24
Chlamydiae bacterium 1021942-13
Chlamydiae sp. genome_bin_33
Chlamydiae bacterium 3300020970-2
Chlamydiae bacterium 1064120-29
Chlamydiae bacterium 3300005529-103
Rhabdochlamydia helveticae
Rhabdochlamydia porcellionis
Rhabdochlamydia oedothoracis
0.1

chain3

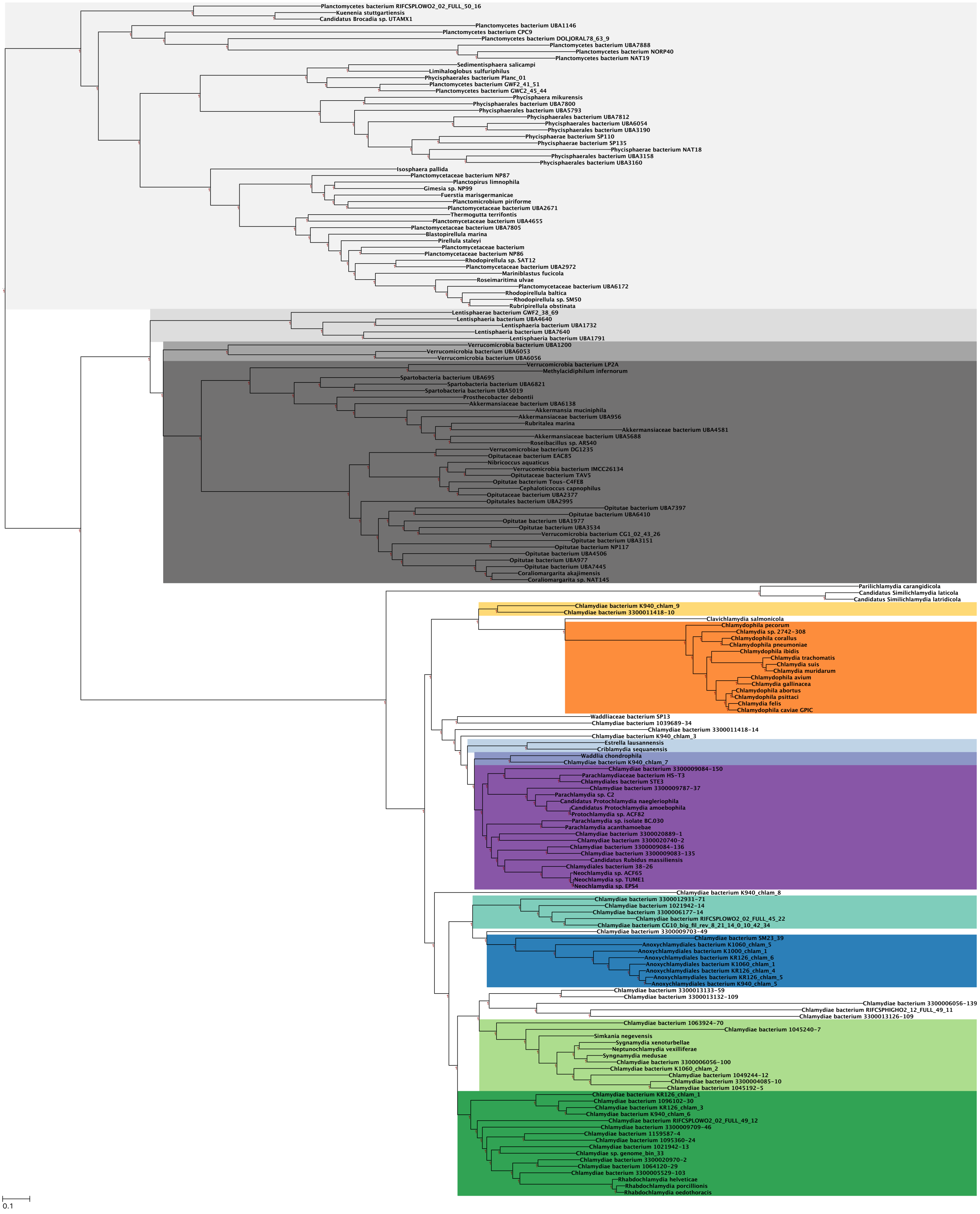

Planctomycetes bacterium RIFCSPLOWO2_02_FULL_50_16
Kuenenia stuttgartiensis
Candidatus Brocadia sp. UTAMX1
Planctomycetes bacterium UBA1146
Planctomycetes bacterium CPC9
Planctomycetes bacterium DOLJORAL78_63_9
Planctomycetes bacterium UBA7888
Planctomycetes bacterium NORP40
Planctomycetes bacterium NAT19
Sedimentisphaera salicampi
Limihaloglobus sulfuriphilus
Phycisphaerales bacterium Planc_01
Planctomycetes bacterium GWF2_41_51
Planctomycetes bacterium GWC2_45_44
Phycisphaera mikurensis
Phycisphaerales bacterium UBA7800
Phycisphaerales bacterium UBA5793
Phycisphaerales bacterium UBA7812
Phycisphaerales bacterium UBA6054
Phycisphaerales bacterium UBA3190
Phycisphaerae bacterium SP110
Phycisphaerae bacterium SP135
Phycisphaerae bacterium NAT18
Phycisphaerales bacterium UBA3158
Phycisphaerales bacterium UBA3160
Isosphaera pallida
Planctomycetaceae bacterium NP87
Planctopirus limnophila
Gimesia sp. NP99
Fuerstia marisgermanicae
Planctomicrobium piriforme
Planctomycetaceae bacterium UBA2671
Thermogutta terrifontis
Planctomycetaceae bacterium UBA4655
Planctomycetaceae bacterium UBA7805
Blastopirellula marina
Pirellula staleyi
Planctomycetaceae bacterium
Planctomycetaceae bacterium NP86
Rhodopirellula sp. SAT12
Planctomycetaceae bacterium UBA2972
Mariniblastus fucicola
Roseimaritima ulvae
Planctomycetaceae bacterium UBA6172
Rhodopirellula baltica
Rhodopirellula sp. SM50
Rubripirellula obstinata
Lentisphaerae bacterium GWF2_38_69
Lentisphaeria bacterium UBA4640
Lentisphaeria bacterium UBA1732
Lentisphaeria bacterium UBA7640
Lentisphaeria bacterium UBA1791
Verrucomicrobia bacterium UBA1200
Verrucomicrobia bacterium UBA6053
Verrucomicrobia bacterium UBA6056
Verrucomicrobia bacterium LP2A
Methylacidiphilum infernorum
Spartobacteria bacterium UBA695
Spartobacteria bacterium UBA6821
Spartobacteria bacterium UBA5019
Prosthecobacter debontii
Akkermansiaceae bacterium UBA6138
Akkermansia muciniphila
Akkermansiaceae bacterium UBA956
Rubritalea marina
Akkermansiaceae bacterium UBA4581
Akkermansiaceae bacterium UBA5688
Roseibacillus sp. ARS40
Verrucomicrobiae bacterium DG1235
Opitutaceae bacterium EAC85
Nibricoccus aquaticus
Verrucomicrobia bacterium IMCC26134
Opitutaceae bacterium TAV5
Opitutae bacterium Tous-C4FEB
Cephaloticoccus capnophilus
Opitutaceae bacterium UBA2377
Opitutales bacterium UBA2995
Opitutae bacterium UBA7397
Opitutae bacterium UBA6410
Opitutae bacterium UBA1977
Opitutae bacterium UBA3534
Verrucomicrobia bacterium CG1_02_43_26
Opitutae bacterium UBA3151
Opitutae bacterium NP117
Opitutae bacterium UBA4506
Opitutae bacterium UBA977
Opitutae bacterium UBA7445
Coraliomargarita akajimensis
Coraliomargarita sp. NAT145
Parilichlamydia carangidicola
Candidatus Similichlamydia laticola
Candidatus Similichlamydia latridicola
Chlamydiae bacterium K940_chlam_9
Chlamydiae bacterium 3300011418-10
Clavichlamydia salmonicola
Chlamydophila pecorum
Chlamydia sp. 2742-308
Chlamydophila corallus
Chlamydophila pneumoniae
Chlamydophila ibidis
Chlamydia trachomatis
Chlamydia suis
Chlamydia muridarum
Chlamydophila avium
Chlamydia gallinacea
Chlamydophila abortus
Chlamydophila psittaci
Chlamydia felis
Chlamydophila caviae GPIC
Waddliaceae bacterium SP13
Chlamydiae bacterium 1039689-34
Chlamydiae bacterium 3300011418-14
Chlamydiae bacterium K940_chlam_3
Estrella lausannensis
Criblamydia sequanensis
Waddlia chondrophila
Chlamydiae bacterium K940_chlam_7
Chlamydiae bacterium 3300009084-150
Parachlamydiaceae bacterium HS-T3
Chlamydiales bacterium STE3
Chlamydiae bacterium 3300009787-37
Parachlamydia sp. C2
Candidatus Protochlamydia naegleriophila
Candidatus Protochlamydia amoebophila
Protochlamydia sp. ACF82
Parachlamydia sp. isolate BC.030
Parachlamydia acanthamoebae
Chlamydiae bacterium 3300020889-1
Chlamydiae bacterium 3300020740-2
Chlamydiae bacterium 3300009084-136
Chlamydiae bacterium 3300009083-135
Candidatus Rubidus massiliensis
Chlamydiales bacterium 38-26
Neochlamydia sp. ACF65
Neochlamydia sp. TUME1
Neochlamydia sp. EPS4
Chlamydiae bacterium K940_chlam_8
Chlamydiae bacterium 3300012931-71
Chlamydiae bacterium 1021942-14
Chlamydiae bacterium 3300006177-14
Chlamydiae bacterium RIFCSPLOWO2_02_FULL_45_22
Chlamydiae bacterium CG10_big_fil_rev_8_21_14_0_10_42_34
Chlamydiae bacterium 3300009703-49
Chlamydiae bacterium SM23_39
Anoxychlamydiales bacterium K1060_chlam_5
Anoxychlamydiales bacterium K1000_chlam_1
Anoxychlamydiales bacterium KR126_chlam_6
Anoxychlamydiales bacterium K1060_chlam_1
Anoxychlamydiales bacterium KR126_chlam_4
Anoxychlamydiales bacterium KR126_chlam_5
Anoxychlamydiales bacterium K940_chlam_5
Chlamydiae bacterium 3300013133-59
Chlamydiae bacterium 3300013132-109
Chlamydiae bacterium 3300006056-139
Chlamydiae bacterium RIFCSPHIGHO2_12_FULL_49_11
Chlamydiae bacterium 3300013126-109
Chlamydiae bacterium 1063924-70
Chlamydiae bacterium 1045240-7
Simkania negevensis
Sygnamydia xenoturbellae
Neptunochlamydia vexilliferae
Syngnamydia medusae
Chlamydiae bacterium 3300006056-100
Chlamydiae bacterium K1060_chlam_2
Chlamydiae bacterium 1049244-12
Chlamydiae bacterium 3300004085-10
Chlamydiae bacterium 1045192-5
Chlamydiae bacterium KR126_chlam_1
Chlamydiae bacterium 1096102-30
Chlamydiae bacterium KR126_chlam_3
Chlamydiae bacterium K940_chlam_6
Chlamydiae bacterium RIFCSPLOWO2_02_FULL_49_12
Chlamydiae bacterium 3300009709-46
Chlamydiae bacterium 1159587-4
Chlamydiae bacterium 1095360-24
Chlamydiae bacterium 1021942-13
Chlamydiae sp. genome_bin_33
Chlamydiae bacterium 3300020970-2
Chlamydiae bacterium 1064120-29
Chlamydiae bacterium 3300005529-103
Rhabdochlamydia helveticae
Rhabdochlamydia porcellionis
Rhabdochlamydia oedothoracis
0.1

chain4

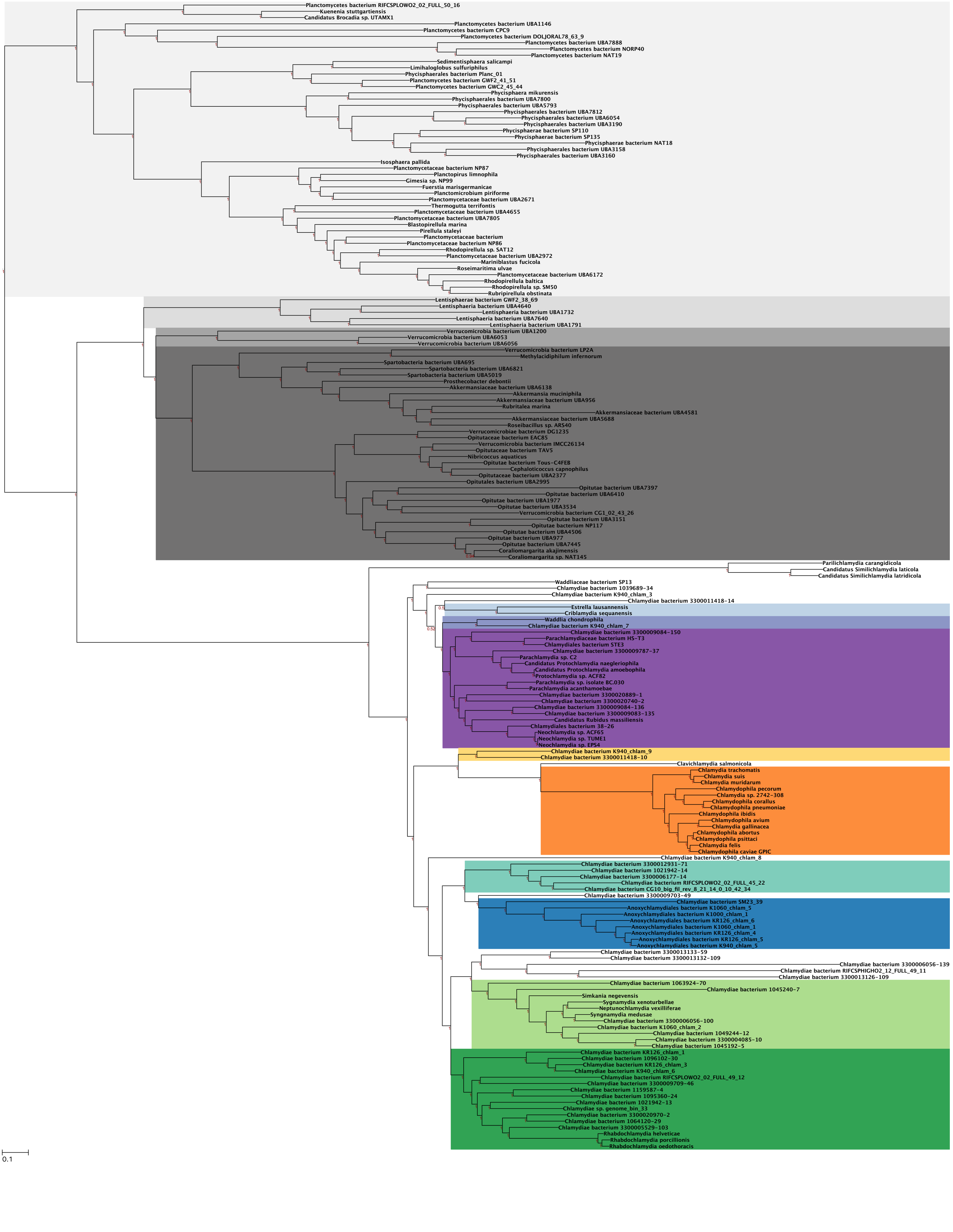

Planctomycetes bacterium RIFCSPLOWO2_02_FULL_50_16
Kuenenia stuttgartiensis
Candidatus Brocadia sp. UTAMX1
Planctomycetes bacterium UBA1146
Planctomycetes bacterium CPC9
Planctomycetes bacterium DOLJORAL78_63_9
Planctomycetes bacterium UBA7888
Planctomycetes bacterium NORP40
Planctomycetes bacterium NAT19
Sedimentisphaera salicampi
Limihaloglobus sulfuriphilus
Phycisphaerales bacterium Planc_01
Planctomycetes bacterium GWF2_41_51
Planctomycetes bacterium GWC2_45_44
Phycisphaera mikurensis
Phycisphaerales bacterium UBA7800
Phycisphaerales bacterium UBA5793
Phycisphaerales bacterium UBA7812
Phycisphaerales bacterium UBA6054
Phycisphaerales bacterium UBA3190
Phycisphaerae bacterium SP110
Phycisphaerae bacterium SP135
Phycisphaerae bacterium NAT18
Phycisphaerales bacterium UBA3158
Phycisphaerales bacterium UBA3160
Isosphaera pallida
Planctomycetaceae bacterium NP87
Planctopirus limnophila
Gimesia sp. NP99
Fuerstia marisgermanicae
Planctomicrobium piriforme
Planctomycetaceae bacterium UBA2671
Thermogutta terrifontis
Planctomycetaceae bacterium UBA4655
Planctomycetaceae bacterium UBA7805
Blastopirellula marina
Pirellula staleyi
Planctomycetaceae bacterium
Planctomycetaceae bacterium NP86
Rhodopirellula sp. SAT12
Planctomycetaceae bacterium UBA2972
Mariniblastus fucicola
Roseimaritima ulvae
Planctomycetaceae bacterium UBA6172
Rhodopirellula baltica
Rhodopirellula sp. SM50
Rubripirellula obstinata
Lentisphaerae bacterium GWF2_38_69
Lentisphaeria bacterium UBA4640
Lentisphaeria bacterium UBA1732
Lentisphaeria bacterium UBA7640
Lentisphaeria bacterium UBA1791
Verrucomicrobia bacterium UBA1200
Verrucomicrobia bacterium UBA6053
Verrucomicrobia bacterium UBA6056
Verrucomicrobia bacterium LP2A
Methylacidiphilum infernorum
Spartobacteria bacterium UBA695
Spartobacteria bacterium UBA6821
Spartobacteria bacterium UBA5019
Prosthecobacter debontii
Akkermansiaceae bacterium UBA6138
Akkermansia muciniphila
Akkermansiaceae bacterium UBA956
Rubritalea marina
Akkermansiaceae bacterium UBA4581
Akkermansiaceae bacterium UBA5688
Roseibacillus sp. ARS40
Verrucomicrobiae bacterium DG1235
Opitutaceae bacterium EAC85
Verrucomicrobia bacterium IMCC26134
Opitutaceae bacterium TAV5
Nibricoccus aquaticus
Opitutae bacterium Tous-C4FEB
Cephaloticoccus capnophilus
Opitutaceae bacterium UBA2377
Opitutales bacterium UBA2995
Opitutae bacterium UBA7397
Opitutae bacterium UBA6410
Opitutae bacterium UBA1977
Opitutae bacterium UBA3534
Verrucomicrobia bacterium CG1_02_43_26
Opitutae bacterium UBA3151
Opitutae bacterium NP117
Opitutae bacterium UBA4506
Opitutae bacterium UBA977
Opitutae bacterium UBA7445
Coraliomargarita akajimensis
Coraliomargarita sp. NAT145
Parilichlamydia carangidicola
Candidatus Similichlamydia laticola
Candidatus Similichlamydia latridicola
Waddliaceae bacterium SP13
Chlamydiae bacterium 1039689-34
Chlamydiae bacterium K940_chlam_3
Chlamydiae bacterium 3300011418-14
Estrella lausannensis
Criblamydia sequanensis
Waddlia chondrophila
Chlamydiae bacterium K940_chlam_7
Chlamydiae bacterium 3300009084-150
Parachlamydiaceae bacterium HS-T3
Chlamydiales bacterium STE3
Chlamydiae bacterium 3300009787-37
Parachlamydia sp. C2
Candidatus Protochlamydia naegleriophila
Candidatus Protochlamydia amoebophila
Protochlamydia sp. ACF82
Parachlamydia sp. isolate BC.030
Parachlamydia acanthamoebae
Chlamydiae bacterium 3300020889-1
Chlamydiae bacterium 3300020740-2
Chlamydiae bacterium 3300009084-136
Chlamydiae bacterium 3300009083-135
Candidatus Rubidus massiliensis
Chlamydiales bacterium 38-26
Neochlamydia sp. ACF65
Neochlamydia sp. TUME1
Neochlamydia sp. EPS4
Chlamydiae bacterium K940_chlam_9
Chlamydiae bacterium 3300011418-10
Clavichlamydia salmonicola
Chlamydia trachomatis
Chlamydia suis
Chlamydia muridarum
Chlamydophila pecorum
Chlamydia sp. 2742-308
Chlamydophila corallus
Chlamydophila pneumoniae
Chlamydophila ibidis
Chlamydophila avium
Chlamydia gallinacea
Chlamydophila abortus
Chlamydophila psittaci
Chlamydia felis
Chlamydophila caviae GPIC
Chlamydiae bacterium K940_chlam_8
Chlamydiae bacterium 3300012931-71
Chlamydiae bacterium 1021942-14
Chlamydiae bacterium 3300006177-14
Chlamydiae bacterium RIFCSPLOWO2_02_FULL_45_22
Chlamydiae bacterium CG10_big_fil_rev_8_21_14_0_10_42_34
Chlamydiae bacterium 3300009703-49
Chlamydiae bacterium SM23_39
Anoxychlamydiales bacterium K1060_chlam_5
Anoxychlamydiales bacterium K1000_chlam_1
Anoxychlamydiales bacterium KR126_chlam_6
Anoxychlamydiales bacterium K1060_chlam_1
Anoxychlamydiales bacterium KR126_chlam_4
Anoxychlamydiales bacterium KR126_chlam_5
Anoxychlamydiales bacterium K940_chlam_5
Chlamydiae bacterium 3300013133-59
Chlamydiae bacterium 3300013132-109
Chlamydiae bacterium 3300006056-139
Chlamydiae bacterium RIFCSPHIGHO2_12_FULL_49_11
Chlamydiae bacterium 3300013126-109
Chlamydiae bacterium 1063924-70
Chlamydiae bacterium 1045240-7
Simkania negevensis
Sygnamydia xenoturbellae
Neptunochlamydia vexilliferae
Syngnamydia medusae
Chlamydiae bacterium 3300006056-100
Chlamydiae bacterium K1060_chlam_2
Chlamydiae bacterium 1049244-12
Chlamydiae bacterium 3300004085-10
Chlamydiae bacterium 1045192-5
Chlamydiae bacterium KR126_chlam_1
Chlamydiae bacterium 1096102-30
Chlamydiae bacterium KR126_chlam_3
Chlamydiae bacterium K940_chlam_6
Chlamydiae bacterium RIFCSPLOWO2_02_FULL_49_12
Chlamydiae bacterium 3300009709-46
Chlamydiae bacterium 1159587-4
Chlamydiae bacterium 1095360-24
Chlamydiae bacterium 1021942-13
Chlamydiae sp. genome_bin_33
Chlamydiae bacterium 3300020970-2
Chlamydiae bacterium 1064120-29
Chlamydiae bacterium 3300005529-103
Rhabdochlamydia helveticae
Rhabdochlamydia porcillionis
Rhabdochlamydia oedothoracis
0.1

convergence_chains_1234

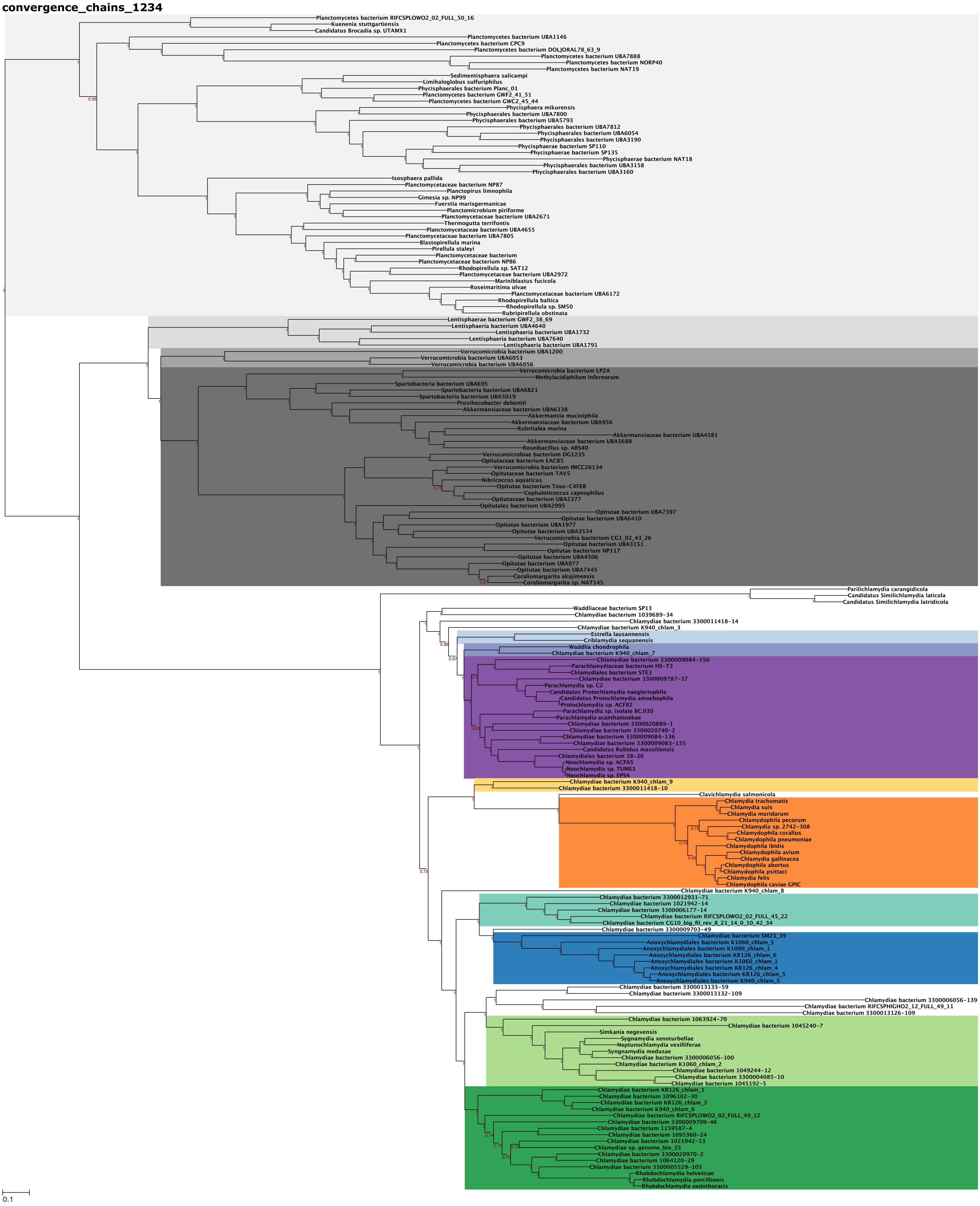

183taxa_10perc_pruned

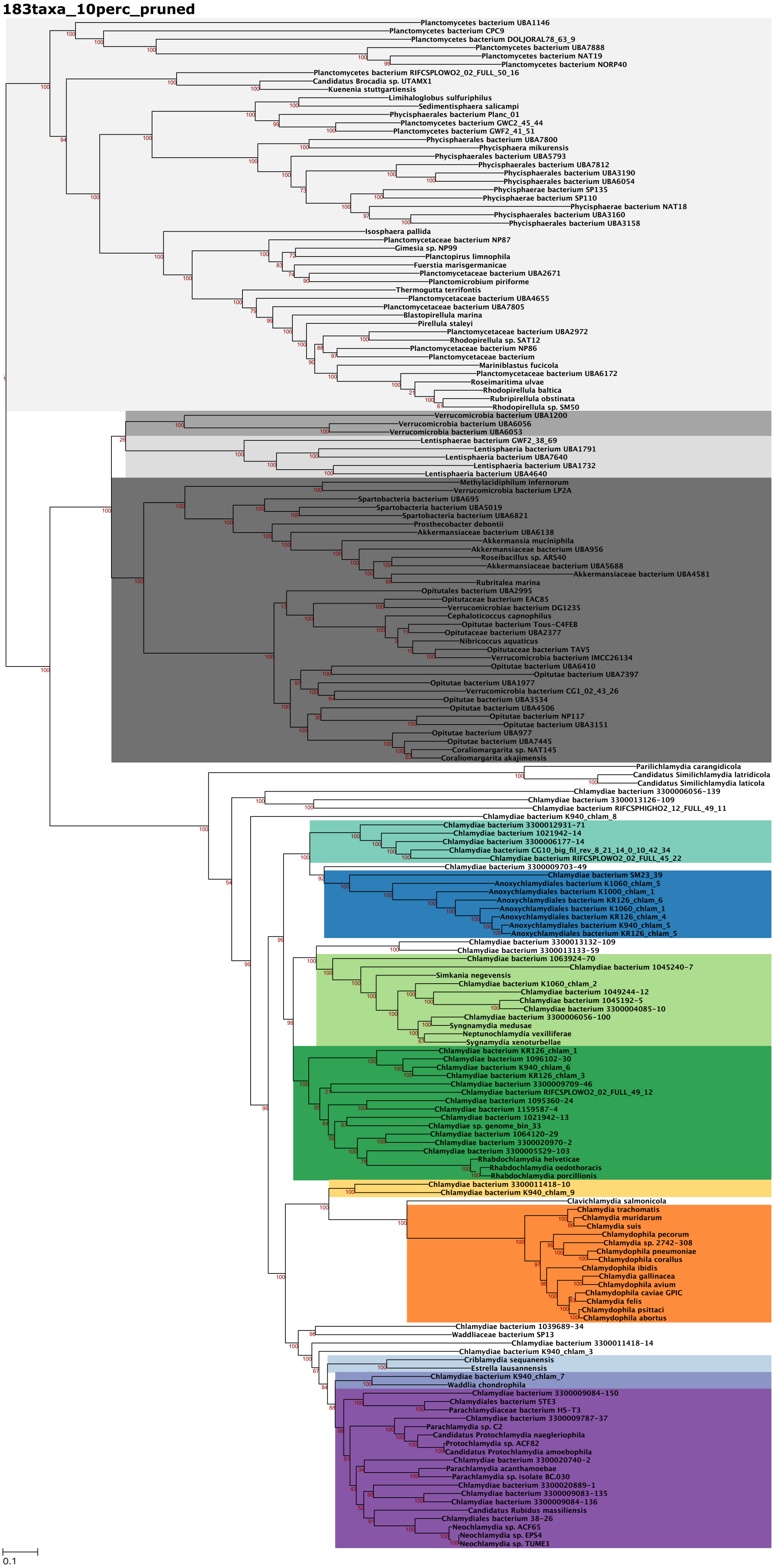

183taxa_20perc_pruned

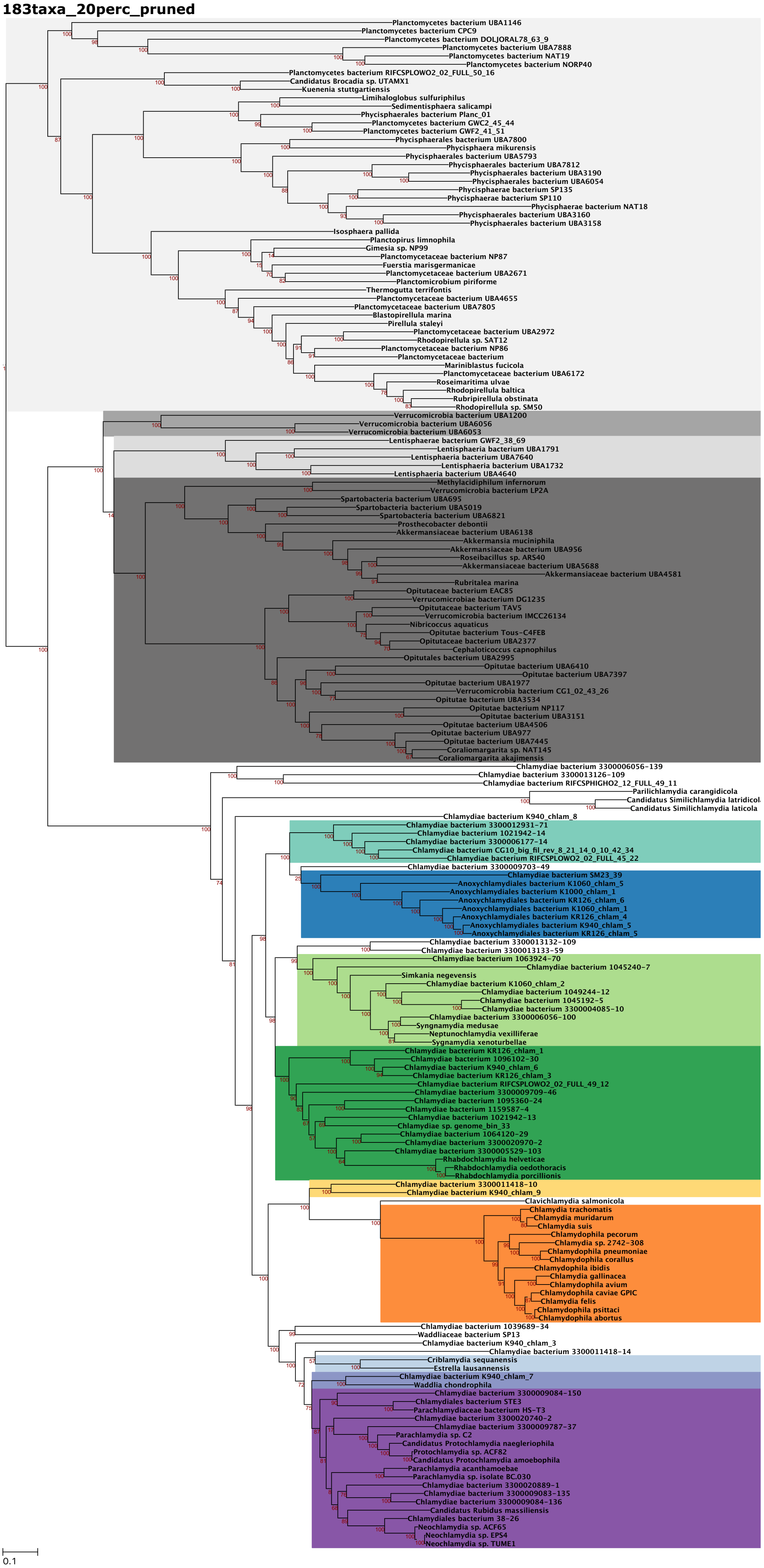

183taxa_30perc_pruned

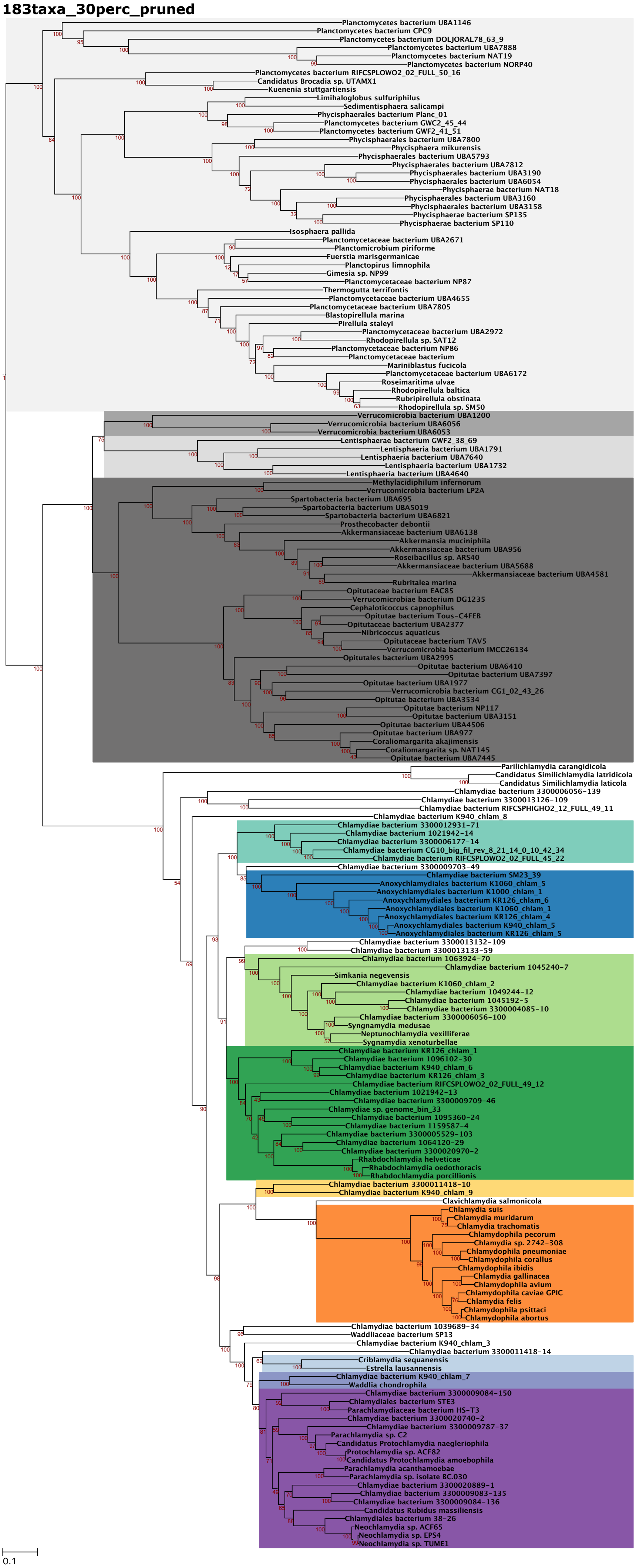

183taxa_40perc_pruned

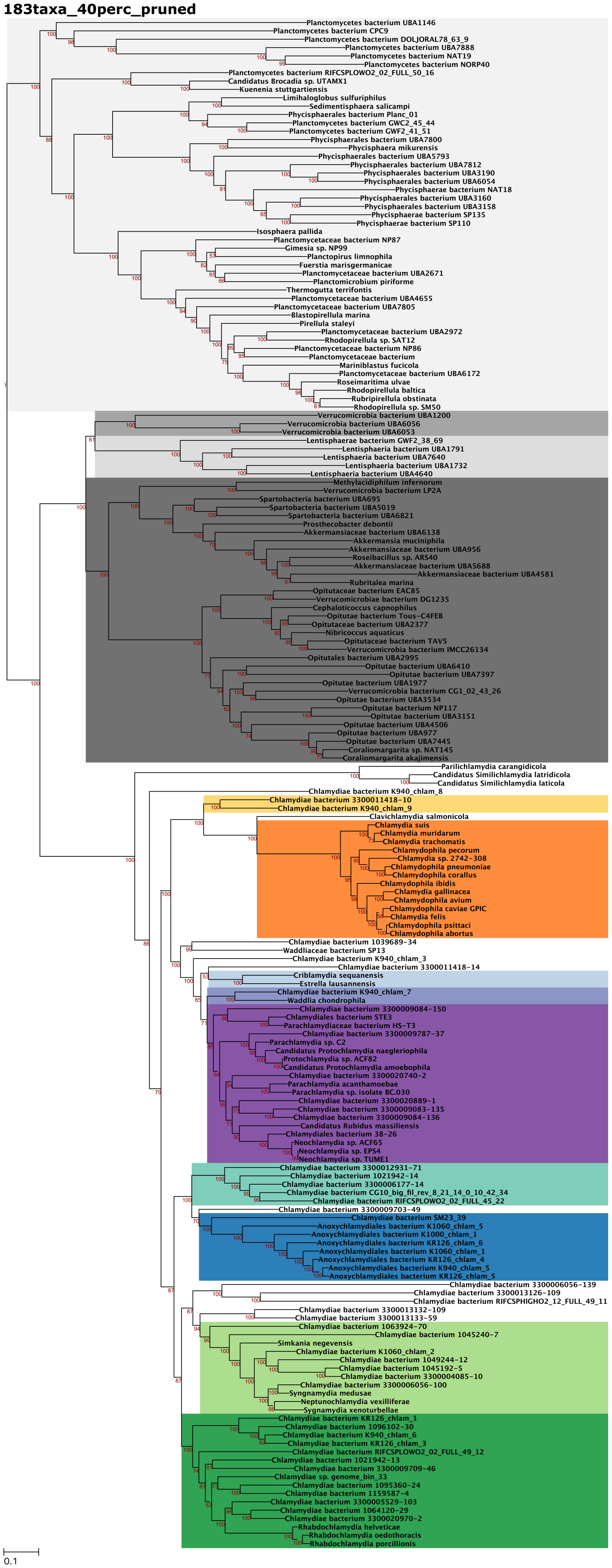

183taxa_48perc_pruned_bias_removed

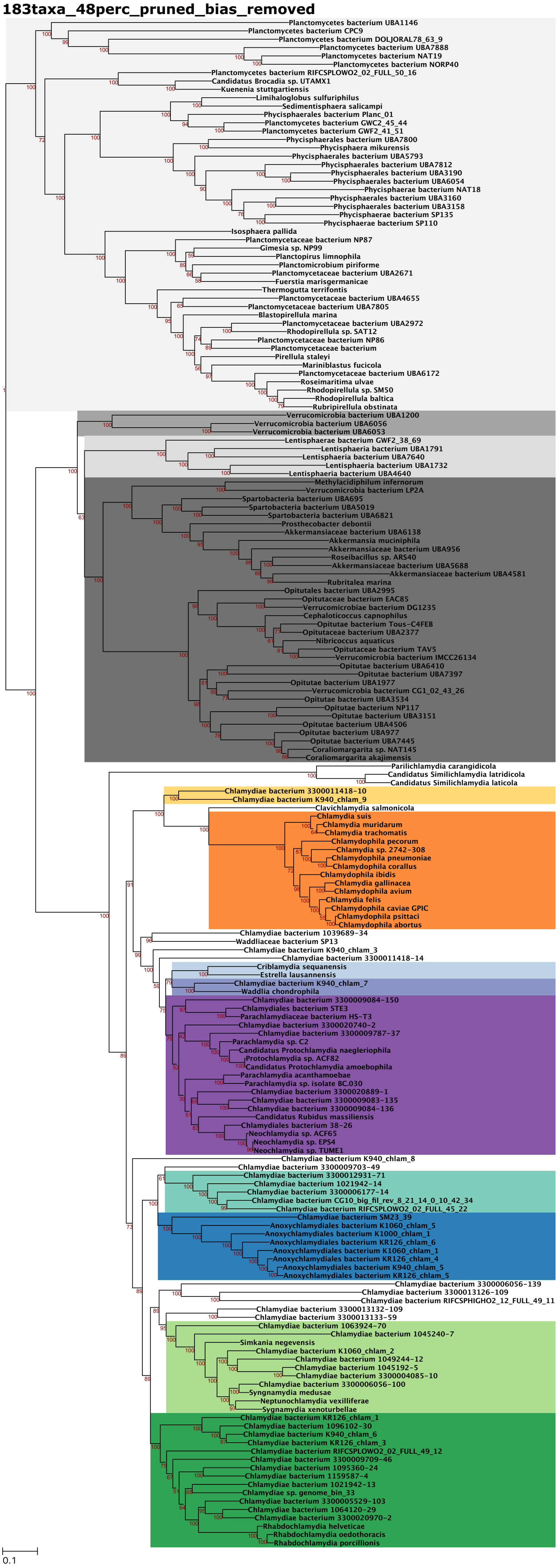

183taxa_48perc_pruned_bias_removed

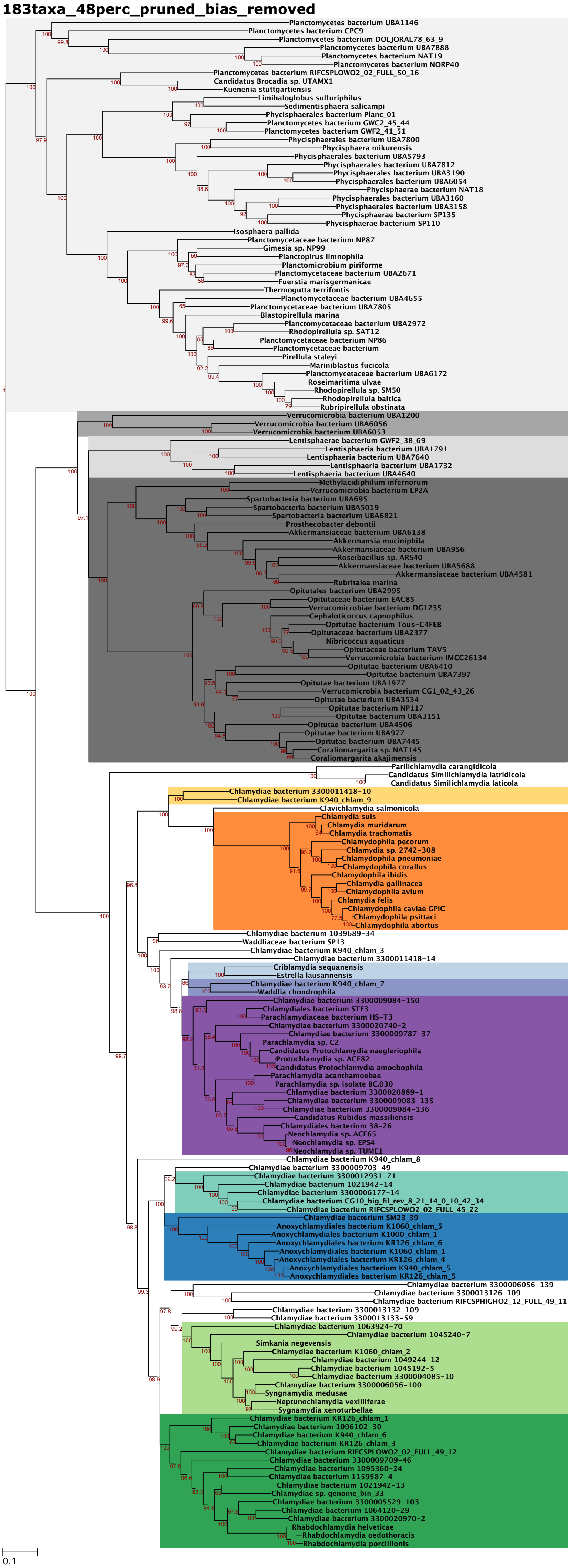

chain1

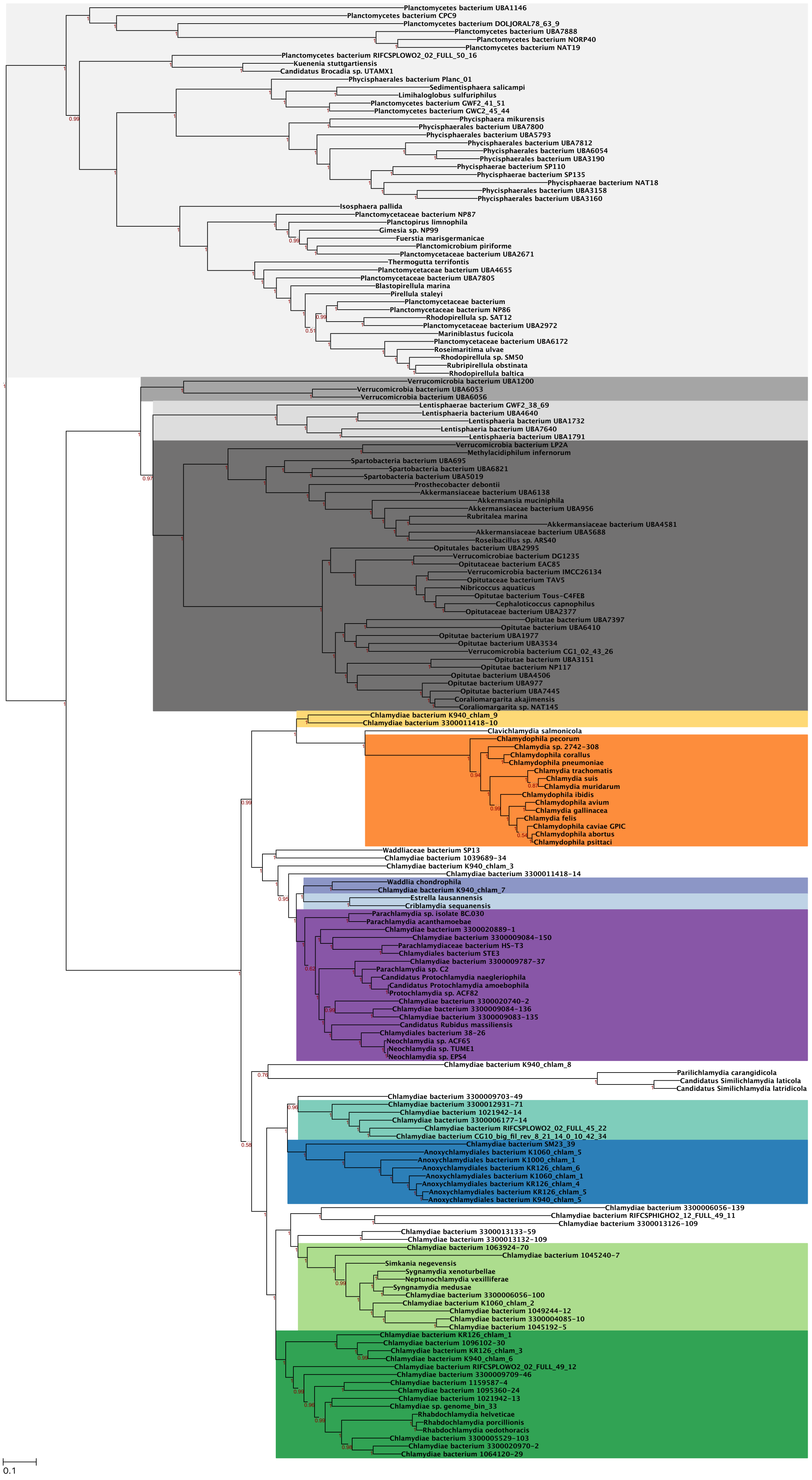

Planctomycetes bacterium UBA1146
Planctomycetes bacterium CPC9
Planctomycetes bacterium DOLJORAL78_63_9
Planctomycetes bacterium UBA7888
Planctomycetes bacterium NORP40
Planctomycetes bacterium NAT19
Planctomycetes bacterium RIFCSPLOWO2_02_FULL_50_16
Kuenenia stuttgartiensis
Candidatus Brocadia sp. UTAMX1
Phycisphaerales bacterium Planc_01
Sedimentisphaera salicampi
Limihaloglobus sulfuriphilus
Planctomycetes bacterium GWF2_41_51
Planctomycetes bacterium GWC2_45_44
Phycisphaera mikurensis
Phycisphaerales bacterium UBA7800
Phycisphaerales bacterium UBA5793
Phycisphaerales bacterium UBA7812
Phycisphaerales bacterium UBA6054
Phycisphaerales bacterium UBA3190
Phycisphaerae bacterium SP110
Phycisphaerae bacterium SP135
Phycisphaerae bacterium NAT18
Phycisphaerales bacterium UBA3158
Phycisphaerales bacterium UBA3160
Isosphaera pallida
Planctomycetaceae bacterium NP87
Planctopirus limnophila
Gimesia sp. NP99
Fuerstia marisgermanicae
Planctomicrobium piriforme
Planctomycetaceae bacterium UBA2671
Thermogutta terrifontis
Planctomycetaceae bacterium UBA4655
Planctomycetaceae bacterium UBA7805
Blastopirellula marina
Pirellula staleyi
Planctomycetaceae bacterium
Planctomycetaceae bacterium NP86
Rhodopirellula sp. SAT12
Planctomycetaceae bacterium UBA2972
Mariniblastus fucicola
Planctomycetaceae bacterium UBA6172
Roseimaritima ulvae
Rhodopirellula sp. SM50
Rubripirellula obstinata
Rhodopirellula baltica
Verrucomicrobia bacterium UBA1200
Verrucomicrobia bacterium UBA6053
Verrucomicrobia bacterium UBA6056
Lentisphaerae bacterium GWF2_38_69
Lentisphaeria bacterium UBA4640
Lentisphaeria bacterium UBA1732
Lentisphaeria bacterium UBA7640
Lentisphaeria bacterium UBA1791
Verrucomicrobia bacterium LP2A
Methylacidiphilum infernorum
Spartobacteria bacterium UBA695
Spartobacteria bacterium UBA6821
Spartobacteria bacterium UBA5019
Prosthecobacter debontii
Akkermansiaceae bacterium UBA6138
Akkermansia muciniphila
Akkermansiaceae bacterium UBA956
Rubritalea marina
Akkermansiaceae bacterium UBA4581
Akkermansiaceae bacterium UBA5688
Roseibacillus sp. ARS40
Opitutales bacterium UBA2995
Verrucomicrobiae bacterium DG1235
Opitutaceae bacterium EAC85
Verrucomicrobia bacterium IMCC26134
Opitutaceae bacterium TAV5
Nibricoccus aquaticus
Opitutae bacterium Tous-C4FEB
Cephaloticoccus capnophilus
Opitutaceae bacterium UBA2377
Opitutae bacterium UBA7397
Opitutae bacterium UBA6410
Opitutae bacterium UBA1977
Opitutae bacterium UBA3534
Verrucomicrobia bacterium CG1_02_43_26
Opitutae bacterium UBA3151
Opitutae bacterium NP117
Opitutae bacterium UBA4506
Opitutae bacterium UBA977
Opitutae bacterium UBA7445
Coraliomargarita akajimensis
Coraliomargarita sp. NAT145
Chlamydiae bacterium K940_chlam_9
Chlamydiae bacterium 3300011418-10
Clavichlamydia salmonicola
Chlamydophila pecorum
Chlamydia sp. 2742-308
Chlamydophila corallus
Chlamydophila pneumoniae
Chlamydia trachomatis
Chlamydia suis
Chlamydia muridarum
Chlamydophila ibidis
Chlamydophila avium
Chlamydia gallinacea
Chlamydia felis
Chlamydophila caviae GPIC
Chlamydophila abortus
Chlamydophila psittaci
Waddliaceae bacterium SP13
Chlamydiae bacterium 1039689-34
Chlamydiae bacterium K940_chlam_3
Chlamydiae bacterium 3300011418-14
Waddlia chondrophila
Chlamydiae bacterium K940_chlam_7
Estrella lausannensis
Criblamydia sequanensis
Parachlamydia sp. isolate BC.030
Parachlamydia acanthamoebae
Chlamydiae bacterium 3300020889-1
Chlamydiae bacterium 3300009084-150
Parachlamydiaceae bacterium HS-T3
Chlamydiales bacterium STE3
Chlamydiae bacterium 3300009787-37
Parachlamydia sp. C2
Candidatus Protochlamydia naegleriophila
Candidatus Protochlamydia amoebophila
Protochlamydia sp. ACF82
Chlamydiae bacterium 3300020740-2
Chlamydiae bacterium 3300009084-136
Chlamydiae bacterium 3300009083-135
Candidatus Rubidus massiliensis
Chlamydiales bacterium 38-26
Neochlamydia sp. ACF65
Neochlamydia sp. TUME1
Neochlamydia sp. EPS4
Chlamydiae bacterium K940_chlam_8
Parilichlamydia carangidicola
Candidatus Similichlamydia laticola
Candidatus Similichlamydia latridicola
Chlamydiae bacterium 3300009703-49
Chlamydiae bacterium 3300012931-71
Chlamydiae bacterium 1021942-14
Chlamydiae bacterium 3300006177-14
Chlamydiae bacterium RIFCSPLOWO2_02_FULL_45_22
Chlamydiae bacterium CG10_big_fil_rev_8_21_14_0_10_42_34
Chlamydiae bacterium SM23_39
Anoxychlamydiales bacterium K1060_chlam_5
Anoxychlamydiales bacterium K1000_chlam_1
Anoxychlamydiales bacterium KR126_chlam_6
Anoxychlamydiales bacterium K1060_chlam_1
Anoxychlamydiales bacterium KR126_chlam_4
Anoxychlamydiales bacterium KR126_chlam_5
Anoxychlamydiales bacterium K940_chlam_5
Chlamydiae bacterium 3300006056-139
Chlamydiae bacterium RIFCSPHIGHO2_12_FULL_49_11
Chlamydiae bacterium 3300013126-109
Chlamydiae bacterium 3300013133-59
Chlamydiae bacterium 3300013132-109
Chlamydiae bacterium 1063924-70
Chlamydiae bacterium 1045240-7
Simkania negevensis
Sygnamydia xenoturbellae
Neptunochlamydia vexilliferae
Syngnamydia medusae
Chlamydiae bacterium 3300006056-100
Chlamydiae bacterium K1060_chlam_2
Chlamydiae bacterium 1049244-12
Chlamydiae bacterium 3300004085-10
Chlamydiae bacterium 1045192-5
Chlamydiae bacterium KR126_chlam_1
Chlamydiae bacterium 1096102-30
Chlamydiae bacterium KR126_chlam_3
Chlamydiae bacterium K940_chlam_6
Chlamydiae bacterium RIFCSPLOWO2_02_FULL_49_12
Chlamydiae bacterium 3300009709-46
Chlamydiae bacterium 1159587-4
Chlamydiae bacterium 1095360-24
Chlamydiae bacterium 1021942-13
Chlamydia sp. genome_bin_33
Rhabdochlamydia helveticae
Rhabdochlamydia porcellionis
Rhabdochlamydia oedothoracis
Chlamydiae bacterium 3300005529-103
Chlamydiae bacterium 3300020970-2
Chlamydiae bacterium 1064120-29
0.1

chain2

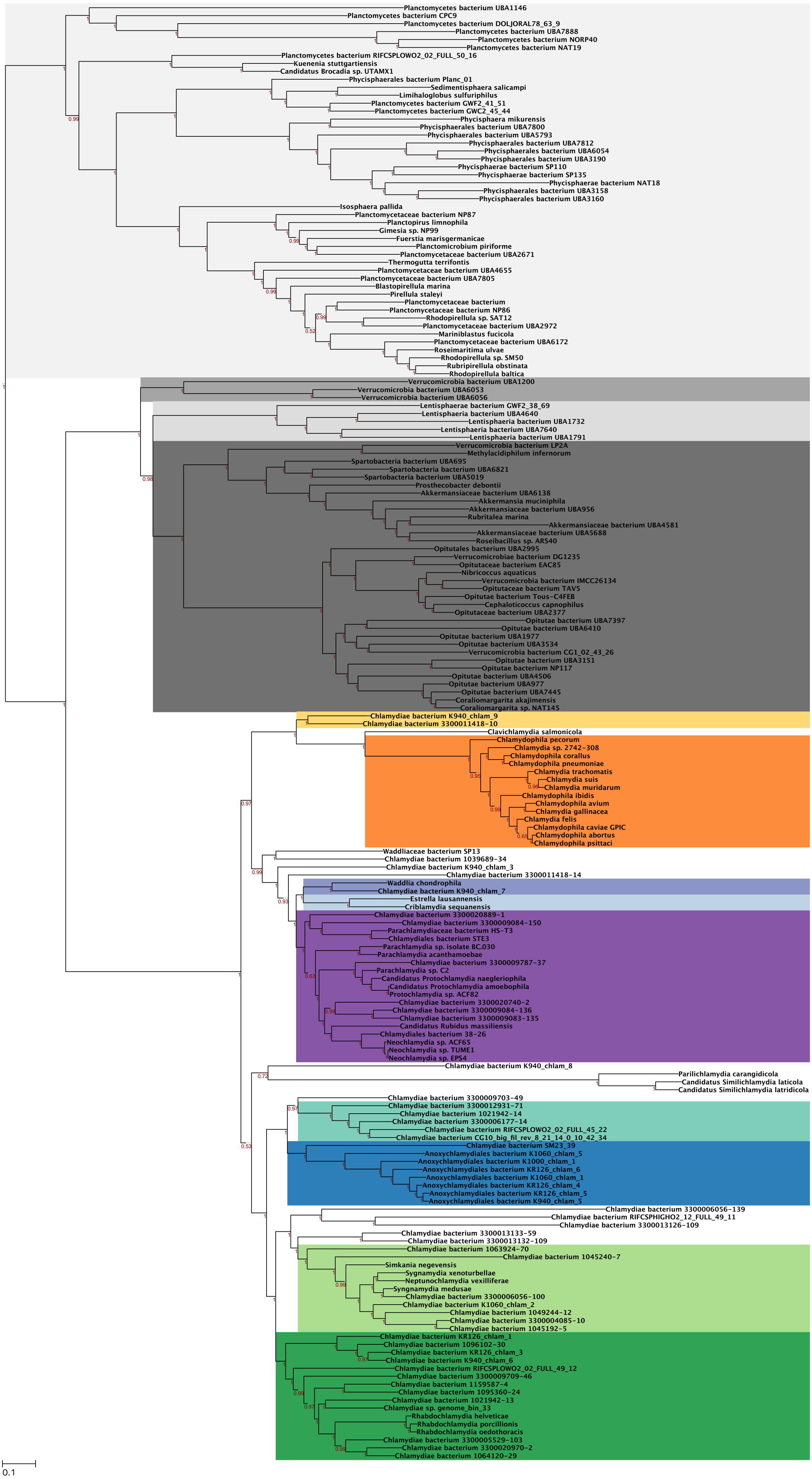
Planctomycetes bacterium UBA1146
Planctomycetes bacterium CPC9
Planctomycetes bacterium DOLJORAL78_63_9
Planctomycetes bacterium UBA7888
Planctomycetes bacterium NORP40
Planctomycetes bacterium NAT19
Planctomycetes bacterium RIFCSPLOWO2_02_FULL_50_16
Kuenenia stuttgartiensis
Candidatus Brocadia sp. UTAMX1
Phycisphaerales bacterium Planc_01
Sedimentisphaera salicampi
Limihaloglobus sulfuriphilus
Planctomycetes bacterium GWF2_41_51
Planctomycetes bacterium GWC2_45_44
Phycisphaera mikurensis
Phycisphaerales bacterium UBA7800
Phycisphaerales bacterium UBA5793
Phycisphaerales bacterium UBA7812
Phycisphaerales bacterium UBA6054
Phycisphaerales bacterium UBA3190
Phycisphaerae bacterium SP110
Phycisphaerae bacterium SP135
Phycisphaerae bacterium NAT18
Phycisphaerales bacterium UBA3158
Phycisphaerales bacterium UBA3160
Isosphaera pallida
Planctomycetaceae bacterium NP87
Planctopirus limnophila
Gimesia sp. NP99
Fuerstia marisgermanicae
Planctomicrobium piriforme
Planctomycetaceae bacterium UBA2671
Thermogutta terrifontis
Planctomycetaceae bacterium UBA4655
Planctomycetaceae bacterium UBA7805
Blastopirellula marina
Pirellula staleyi
Planctomycetaceae bacterium
Planctomycetaceae bacterium NP86
Rhodopirellula sp. SAT12
Planctomycetaceae bacterium UBA2972
Mariniblastus fucicola
Planctomycetaceae bacterium UBA6172
Roseimaritima ulvae
Rhodopirellula sp. SM50
Rubripirellula obstinata
Rhodopirellula baltica
Verrucomicrobia bacterium UBA1200
Verrucomicrobia bacterium UBA6053
Verrucomicrobia bacterium UBA6056
Lentisphaerae bacterium GWF2_38_69
Lentisphaeria bacterium UBA4640
Lentisphaeria bacterium UBA1732
Lentisphaeria bacterium UBA7640
Lentisphaeria bacterium UBA1791
Verrucomicrobia bacterium LP2A
Methylacidiphilum infernorum
Spartobacteria bacterium UBA695
Spartobacteria bacterium UBA6821
Spartobacteria bacterium UBA5019
Prosthecobacter debontii
Akkermansiaceae bacterium UBA6138
Akkermansia muciniphila
Akkermansiaceae bacterium UBA956
Rubritalea marina
Akkermansiaceae bacterium UBA4581
Akkermansiaceae bacterium UBA5688
Roseibacillus sp. ARS40
Opitutales bacterium UBA2995
Verrucomicrobiae bacterium DG1235
Opitutaceae bacterium EAC85
Nibricoccus aquaticus
Verrucomicrobia bacterium IMCC26134
Opitutaceae bacterium TAV5
Opitutae bacterium Tous-C4FEB
Cephaloticoccus capnophilus
Opitutaceae bacterium UBA2377
Opitutae bacterium UBA7397
Opitutae bacterium UBA6410
Opitutae bacterium UBA1977
Opitutae bacterium UBA3534
Verrucomicrobia bacterium CG1_02_43_26
Opitutae bacterium UBA3151
Opitutae bacterium NP117
Opitutae bacterium UBA4506
Opitutae bacterium UBA977
Opitutae bacterium UBA7445
Coraliomargarita akajimensis
Coraliomargarita sp. NAT145
Chlamydiae bacterium K940_chlam_9
Chlamydiae bacterium 3300011418-10
Clavichlamydia salmonicola
Chlamydophila pecorum
Chlamydia sp. 2742-308
Chlamydophila corallus
Chlamydophila pneumoniae
Chlamydia trachomatis
Chlamydia suis
Chlamydia muridarum
Chlamydophila ibidis
Chlamydophila avium
Chlamydia gallinacea
Chlamydia felis
Chlamydophila caviae GPIC
Chlamydophila abortus
Chlamydophila psittaci
Waddliaceae bacterium SP13
Chlamydiae bacterium 1039689-34
Chlamydiae bacterium K940_chlam_3
Chlamydiae bacterium 3300011418-14
Waddlia chondrophila
Chlamydiae bacterium K940_chlam_7
Estrella lausannensis
Criblamydia sequanensis
Chlamydiae bacterium 3300020889-1
Chlamydiae bacterium 3300009084-150
Parachlamydiaceae bacterium HS-T3
Chlamydiales bacterium STE3
Parachlamydia sp. isolate BC.030
Parachlamydia acanthamoebae
Chlamydiae bacterium 3300009787-37
Parachlamydia sp. C2
Candidatus Protochlamydia naegleriophila
Candidatus Protochlamydia amoebophila
Protochlamydia sp. ACF82
Chlamydiae bacterium 3300020740-2
Chlamydiae bacterium 3300009084-136
Chlamydiae bacterium 3300009083-135
Candidatus Rubidus massiliensis
Chlamydiales bacterium 38-26
Neochlamydia sp. ACF65
Neochlamydia sp. TUME1
Neochlamydia sp. EPS4
Chlamydiae bacterium K940_chlam_8
Parilichlamydia carangidicola
Candidatus Similichlamydia laticola
Candidatus Similichlamydia latridicola
Chlamydiae bacterium 3300009703-49
Chlamydiae bacterium 3300012931-71
Chlamydiae bacterium 1021942-14
Chlamydiae bacterium 3300006177-14
Chlamydiae bacterium RIFCSPLOWO2_02_FULL_45_22
Chlamydiae bacterium CG10_big_fil_rev_8_21_14_0_10_42_34
Chlamydiae bacterium SM23_39
Anoxychlamydiales bacterium K1060_chlam_5
Anoxychlamydiales bacterium K1000_chlam_1
Anoxychlamydiales bacterium KR126_chlam_6
Anoxychlamydiales bacterium K1060_chlam_1
Anoxychlamydiales bacterium KR126_chlam_4
Anoxychlamydiales bacterium KR126_chlam_5
Anoxychlamydiales bacterium K940_chlam_5
Chlamydiae bacterium 3300006056-139
Chlamydiae bacterium RIFCSPHIGHO2_12_FULL_49_11
Chlamydiae bacterium 3300013126-109
Chlamydiae bacterium 3300013133-59
Chlamydiae bacterium 3300013132-109
Chlamydiae bacterium 1063924-70
Chlamydiae bacterium 1045240-7
Simkania negevensis
Sygnamydia xenoturbellae
Neptunochlamydia vexilliferae
Syngnamydia medusae
Chlamydiae bacterium 3300006056-100
Chlamydiae bacterium K1060_chlam_2
Chlamydiae bacterium 1049244-12
Chlamydiae bacterium 3300004085-10
Chlamydiae bacterium 1045192-5
Chlamydiae bacterium KR126_chlam_1
Chlamydiae bacterium 1096102-30
Chlamydiae bacterium KR126_chlam_3
Chlamydiae bacterium K940_chlam_6
Chlamydiae bacterium RIFCSPLOWO2_02_FULL_49_12
Chlamydiae bacterium 3300009709-46
Chlamydiae bacterium 1159587-4
Chlamydiae bacterium 1095360-24
Chlamydiae bacterium 1021942-13
Chlamydiae sp. genome_bin_33
Rhabdochlamydia helveticae
Rhabdochlamydia porcillionis
Rhabdochlamydia oedothoracis
Chlamydiae bacterium 3300005529-103
Chlamydiae bacterium 3300020970-2
Chlamydiae bacterium 1064120-29
0.1

chain3

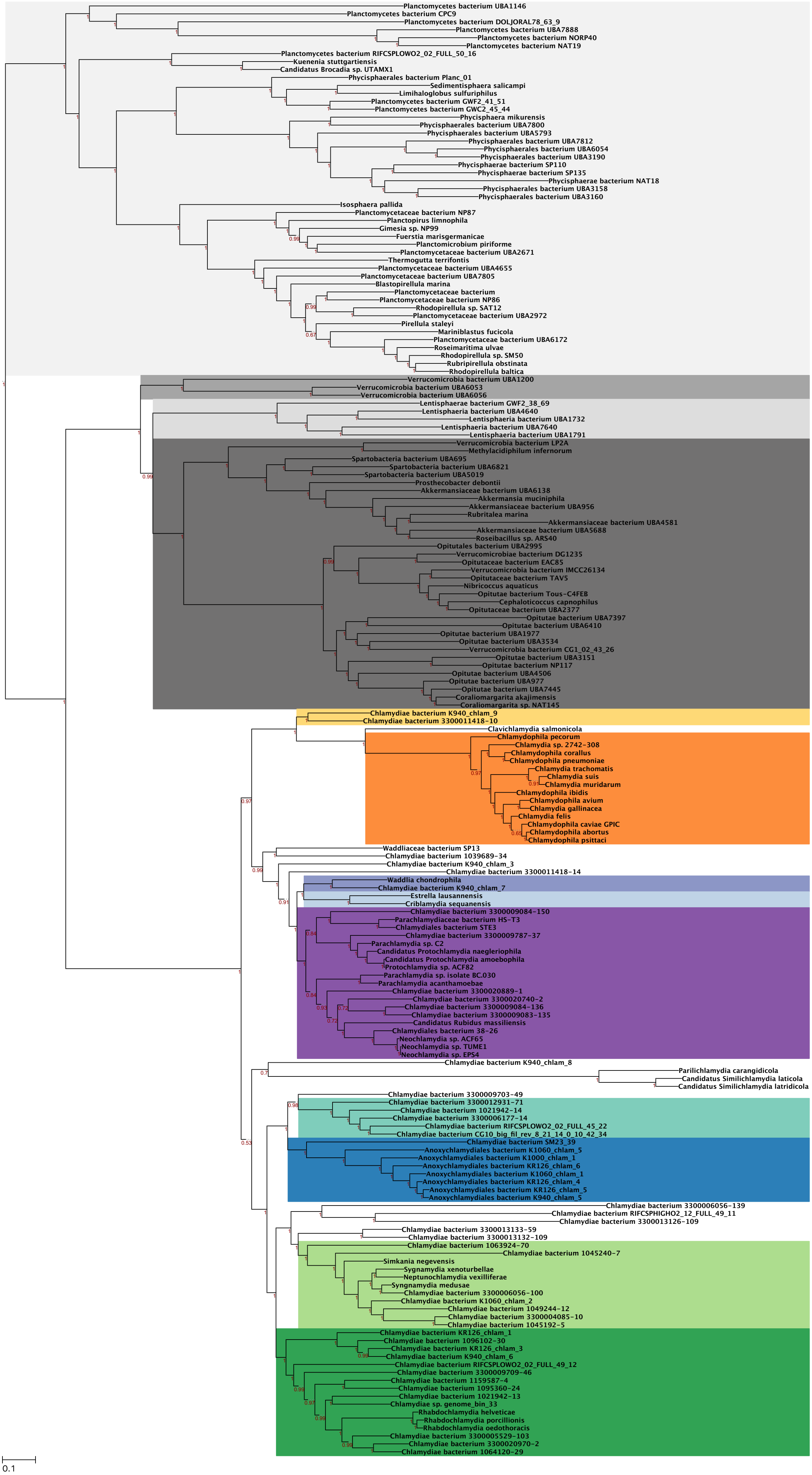
Planctomycetes bacterium UBA1146
Planctomycetes bacterium CPC9
Planctomycetes bacterium DOLJORAL78_63_9
Planctomycetes bacterium UBA7888
Planctomycetes bacterium NORP40
Planctomycetes bacterium NAT19
Planctomycetes bacterium RIFCSPLOWO2_02_FULL_50_16
Kuenenia stuttgartiensis
Candidatus Brocadia sp. UTAMX1
Phycisphaerales bacterium Planc_01
Sedimentisphaera salicampi
Limihaloglobus sulfuriphilus
Planctomycetes bacterium GWF2_41_51
Planctomycetes bacterium GWC2_45_44
Phycisphaera mikurensis
Phycisphaerales bacterium UBA7800
Phycisphaerales bacterium UBA5793
Phycisphaerales bacterium UBA7812
Phycisphaerales bacterium UBA6054
Phycisphaerales bacterium UBA3190
Phycisphaerae bacterium SP110
Phycisphaerae bacterium SP135
Phycisphaerae bacterium NAT18
Phycisphaerales bacterium UBA3158
Phycisphaerales bacterium UBA3160
Isosphaera pallida
Planctomycetaceae bacterium NP87
Planctopirus limnophila
Gimesia sp. NP99
Fuerstia marisgermanicae
Planctomicrobium piriforme
Planctomycetaceae bacterium UBA2671
Thermogutta terrifontis
Planctomycetaceae bacterium UBA4655
Planctomycetaceae bacterium UBA7805
Blastopirellula marina
Planctomycetaceae bacterium
Planctomycetaceae bacterium NP86
Rhodopirellula sp. SAT12
Planctomycetaceae bacterium UBA2972
Pirellula staleyi
Mariniblastus fucicola
Planctomycetaceae bacterium UBA6172
Roseimaritima ulvae
Rhodopirellula sp. SM50
Rubripirellula obstinata
Rhodopirellula baltica
Verrucomicrobia bacterium UBA1200
Verrucomicrobia bacterium UBA6053
Verrucomicrobia bacterium UBA6056
Lentisphaerae bacterium GWF2_38_69
Lentisphaeria bacterium UBA4640
Lentisphaeria bacterium UBA1732
Lentisphaeria bacterium UBA7640
Lentisphaeria bacterium UBA1791
Verrucomicrobia bacterium LP2A
Methylacidiphilum infernorum
Spartobacteria bacterium UBA695
Spartobacteria bacterium UBA6821
Spartobacteria bacterium UBA5019
Prosthecobacter debontii
Akkermansiaceae bacterium UBA6138
Akkermansia muciniphila
Akkermansiaceae bacterium UBA956
Rubritalea marina
Akkermansiaceae bacterium UBA4581
Akkermansiaceae bacterium UBA5688
Roseibacillus sp. ARS40
Opitutales bacterium UBA2995
Verrucomicrobiae bacterium DG1235
Opitutaceae bacterium EAC85
Verrucomicrobia bacterium IMCC26134
Opitutaceae bacterium TAV5
Nibricoccus aquaticus
Opitutae bacterium Tous-C4FEB
Cephaloticoccus capnophilus
Opitutaceae bacterium UBA2377
Opitutae bacterium UBA7397
Opitutae bacterium UBA6410
Opitutae bacterium UBA1977
Opitutae bacterium UBA3534
Verrucomicrobia bacterium CG1_02_43_26
Opitutae bacterium UBA3151
Opitutae bacterium NP117
Opitutae bacterium UBA4506
Opitutae bacterium UBA977
Opitutae bacterium UBA7445
Coraliomargarita akajimensis
Coraliomargarita sp. NAT145
Chlamydiae bacterium K940_chlam_9
Chlamydiae bacterium 3300011418-10
Clavichlamydia salmonicola
Chlamydophila pecorum
Chlamydia sp. 2742-308
Chlamydophila corallus
Chlamydophila pneumoniae
Chlamydia trachomatis
Chlamydia suis
Chlamydia muridarum
Chlamydophila ibidis
Chlamydophila avium
Chlamydia gallinacea
Chlamydia felis
Chlamydophila caviae GPIC
Chlamydophila abortus
Chlamydophila psittaci
Waddliaceae bacterium SP13
Chlamydiae bacterium 1039689-34
Chlamydiae bacterium K940_chlam_3
Chlamydiae bacterium 3300011418-14
Waddlia chondrophila
Chlamydiae bacterium K940_chlam_7
Estrella lausannensis
Criblamydia sequanensis
Chlamydiae bacterium 3300009084-150
Parachlamydiaceae bacterium HS-T3
Chlamydiales bacterium STE3
Chlamydiae bacterium 3300009787-37
Parachlamydia sp. C2
Candidatus Protochlamydia naegleriophila
Candidatus Protochlamydia amoebophila
Protochlamydia sp. ACF82
Parachlamydia sp. isolate BC.030
Parachlamydia acanthamoebae
Chlamydiae bacterium 3300020889-1
Chlamydiae bacterium 3300020740-2
Chlamydiae bacterium 3300009084-136
Chlamydiae bacterium 3300009083-135
Candidatus Rubidus massiliensis
Chlamydiales bacterium 38-26
Neochlamydia sp. ACF65
Neochlamydia sp. TUME1
Neochlamydia sp. EPS4
Chlamydiae bacterium K940_chlam_8
Parilichlamydia carangidicola
Candidatus Similichlamydia laticola
Candidatus Similichlamydia latridicola
Chlamydiae bacterium 3300009703-49
Chlamydiae bacterium 3300012931-71
Chlamydiae bacterium 1021942-14
Chlamydiae bacterium 3300006177-14
Chlamydiae bacterium RIFCSPLOWO2_02_FULL_45_22
Chlamydiae bacterium CG10_big_fil_rev_8_21_14_0_10_42_34
Chlamydiae bacterium SM23_39
Anoxychlamydiales bacterium K1060_chlam_5
Anoxychlamydiales bacterium K1000_chlam_1
Anoxychlamydiales bacterium KR126_chlam_6
Anoxychlamydiales bacterium K1060_chlam_1
Anoxychlamydiales bacterium KR126_chlam_4
Anoxychlamydiales bacterium KR126_chlam_5
Anoxychlamydiales bacterium K940_chlam_5
Chlamydiae bacterium 3300006056-139
Chlamydiae bacterium RIFCSPHIGHO2_12_FULL_49_11
Chlamydiae bacterium 3300013126-109
Chlamydiae bacterium 3300013133-59
Chlamydiae bacterium 3300013132-109
Chlamydiae bacterium 1063924-70
Chlamydiae bacterium 1045240-7
Simkania negevensis
Sygnamydia xenoturbellae
Neptunochlamydia vexilliferae
Syngnamydia medusae
Chlamydiae bacterium 3300006056-100
Chlamydiae bacterium K1060_chlam_2
Chlamydiae bacterium 1049244-12
Chlamydiae bacterium 3300004085-10
Chlamydiae bacterium 1045192-5
Chlamydiae bacterium KR126_chlam_1
Chlamydiae bacterium 1096102-30
Chlamydiae bacterium KR126_chlam_3
Chlamydiae bacterium K940_chlam_6
Chlamydiae bacterium RIFCSPLOWO2_02_FULL_49_12
Chlamydiae bacterium 3300009709-46
Chlamydiae bacterium 1159587-4
Chlamydiae bacterium 1095360-24
Chlamydiae bacterium 1021942-13
Chlamydiae sp. genome_bin_33
Rhabdochlamydia helveticae
Rhabdochlamydia porcellionis
Rhabdochlamydia oedothoracis
Chlamydiae bacterium 3300005529-103
Chlamydiae bacterium 3300020970-2
Chlamydiae bacterium 1064120-29
0.1

chain4

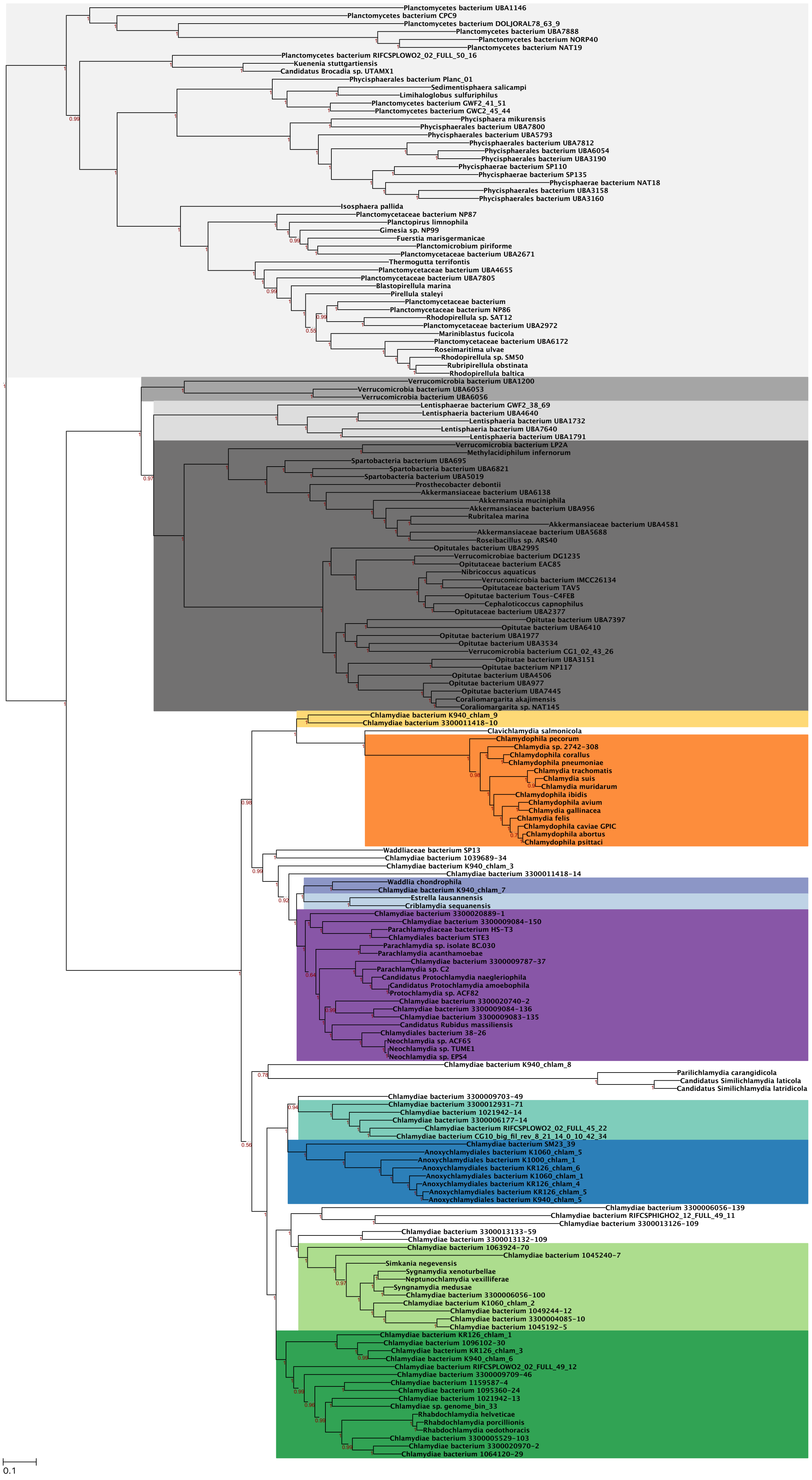
Planctomycetes bacterium UBA1146
Planctomycetes bacterium CPC9
Planctomycetes bacterium DOLJORAL78_63_9
Planctomycetes bacterium UBA7888
Planctomycetes bacterium NORP40
Planctomycetes bacterium NAT19
Planctomycetes bacterium RIFCSPLOWO2_02_FULL_50_16
Kuenenia stuttgartiensis
Candidatus Brocadia sp. UTAMX1
Phycisphaerales bacterium Planc_01
Sedimentisphaera salicampi
Limihaloglobus sulfuriphilus
Planctomycetes bacterium GWF2_41_51
Planctomycetes bacterium GWC2_45_44
Phycisphaera mikurensis
Phycisphaerales bacterium UBA7800
Phycisphaerales bacterium UBA5793
Phycisphaerales bacterium UBA7812
Phycisphaerales bacterium UBA6054
Phycisphaerales bacterium UBA3190
Phycisphaerae bacterium SP110
Phycisphaerae bacterium SP135
Phycisphaerae bacterium NAT18
Phycisphaerales bacterium UBA3158
Phycisphaerales bacterium UBA3160
Isosphaera pallida
Planctomycetaceae bacterium NP87
Planctopirus limnophila
Gimesia sp. NP99
Fuerstia marisgermanicae
Planctomicrobium piriforme
Planctomycetaceae bacterium UBA2671
Thermogutta terrifontis
Planctomycetaceae bacterium UBA4655
Planctomycetaceae bacterium UBA7805
Blastopirellula marina
Pirellula staleyi
Planctomycetaceae bacterium
Planctomycetaceae bacterium NP86
Rhodopirellula sp. SAT12
Planctomycetaceae bacterium UBA2972
Mariniblastus fucicola
Planctomycetaceae bacterium UBA6172
Roseimaritima ulvae
Rhodopirellula sp. SM50
Rubripirellula obstinata
Rhodopirellula baltica
Verrucomicrobia bacterium UBA1200
Verrucomicrobia bacterium UBA6053
Verrucomicrobia bacterium UBA6056
Lentisphaerae bacterium GWF2_38_69
Lentisphaeria bacterium UBA4640
Lentisphaeria bacterium UBA1732
Lentisphaeria bacterium UBA7640
Lentisphaeria bacterium UBA1791
Verrucomicrobia bacterium LP2A
Methylacidiphilum infernorum
Spartobacteria bacterium UBA695
Spartobacteria bacterium UBA6821
Spartobacteria bacterium UBA5019
Prosthecobacter debontii
Akkermansiaceae bacterium UBA6138
Akkermansia muciniphila
Akkermansiaceae bacterium UBA956
Rubritalea marina
Akkermansiaceae bacterium UBA4581
Akkermansiaceae bacterium UBA5688
Roseibacillus sp. ARS40
Opitutales bacterium UBA2995
Verrucomicrobiae bacterium DG1235
Opitutaceae bacterium EAC85
Nibricoccus aquaticus
Verrucomicrobia bacterium IMCC26134
Opitutaceae bacterium TAV5
Opitutae bacterium Tous-C4FEB
Cephaloticoccus capnophilus
Opitutaceae bacterium UBA2377
Opitutae bacterium UBA7397
Opitutae bacterium UBA6410
Opitutae bacterium UBA1977
Opitutae bacterium UBA3534
Verrucomicrobia bacterium CG1_02_43_26
Opitutae bacterium UBA3151
Opitutae bacterium NP117
Opitutae bacterium UBA4506
Opitutae bacterium UBA977
Opitutae bacterium UBA7445
Coraliomargarita akajimensis
Coraliomargarita sp. NAT145
Chlamydiae bacterium K940_chlam_9
Chlamydiae bacterium 3300011418-10
Clavichlamydia salmonicola
Chlamydophila pecorum
Chlamydia sp. 2742-308
Chlamydophila corallus
Chlamydophila pneumoniae
Chlamydia trachomatis
Chlamydia suis
Chlamydia muridarum
Chlamydophila ibidis
Chlamydophila avium
Chlamydia gallinacea
Chlamydia felis
Chlamydophila caviae GPIC
Chlamydophila abortus
Chlamydophila psittaci
Waddliaceae bacterium SP13
Chlamydiae bacterium 1039689-34
Chlamydiae bacterium K940_chlam_3
Chlamydiae bacterium 3300011418-14
Waddlia chondrophila
Chlamydiae bacterium K940_chlam_7
Estrella lausannensis
Criblamydia sequanensis
Chlamydiae bacterium 3300020889-1
Chlamydiae bacterium 3300009084-150
Parachlamydiaceae bacterium HS-T3
Chlamydiales bacterium STE3
Parachlamydia sp. isolate BC.030
Parachlamydia acanthamoebae
Chlamydiae bacterium 3300009787-37
Parachlamydia sp. C2
Candidatus Protochlamydia naegleriophila
Candidatus Protochlamydia amoebophila
Protochlamydia sp. ACF82
Chlamydiae bacterium 3300020740-2
Chlamydiae bacterium 3300009084-136
Chlamydiae bacterium 3300009083-135
Candidatus Rubidus massiliensis
Chlamydiales bacterium 38-26
Neochlamydia sp. ACF65
Neochlamydia sp. TUME1
Neochlamydia sp. EPS4
Chlamydiae bacterium K940_chlam_8
Parilichlamydia carangidicola
Candidatus Similichlamydia laticola
Candidatus Similichlamydia latridicola
Chlamydiae bacterium 3300009703-49
Chlamydiae bacterium 3300012931-71
Chlamydiae bacterium 1021942-14
Chlamydiae bacterium 3300006177-14
Chlamydiae bacterium RIFCSPLOWO2_02_FULL_45_22
Chlamydiae bacterium CG10_big_fil_rev_8_21_14_0_10_42_34
Chlamydiae bacterium SM23_39
Anoxychlamydiales bacterium K1060_chlam_5
Anoxychlamydiales bacterium K1000_chlam_1
Anoxychlamydiales bacterium KR126_chlam_6
Anoxychlamydiales bacterium K1060_chlam_1
Anoxychlamydiales bacterium KR126_chlam_4
Anoxychlamydiales bacterium KR126_chlam_5
Anoxychlamydiales bacterium K940_chlam_5
Chlamydiae bacterium 3300006056-139
Chlamydiae bacterium RIFCSPHIGHO2_12_FULL_49_11
Chlamydiae bacterium 3300013126-109
Chlamydiae bacterium 3300013133-59
Chlamydiae bacterium 3300013132-109
Chlamydiae bacterium 1063924-70
Chlamydiae bacterium 1045240-7
Simkania negevensis
Sygnamydia xenoturbellae
Neptunochlamydia vexilliferae
Syngnamydia medusae
Chlamydiae bacterium 3300006056-100
Chlamydiae bacterium K1060_chlam_2
Chlamydiae bacterium 1049244-12
Chlamydiae bacterium 3300004085-10
Chlamydiae bacterium 1045192-5
Chlamydiae bacterium KR126_chlam_1
Chlamydiae bacterium 1096102-30
Chlamydiae bacterium KR126_chlam_3
Chlamydiae bacterium K940_chlam_6
Chlamydiae bacterium RIFCSPLOWO2_02_FULL_49_12
Chlamydiae bacterium 3300009709-46
Chlamydiae bacterium 1159587-4
Chlamydiae bacterium 1095360-24
Chlamydiae bacterium 1021942-13
Chlamydiae sp. genome_bin_33
Rhabdochlamydia helveticae
Rhabdochlamydia porcellionis
Rhabdochlamydia oedothoracis
Chlamydiae bacterium 3300005529-103
Chlamydiae bacterium 3300020970-2
Chlamydiae bacterium 1064120-29
0.1

convergence_chains_1234

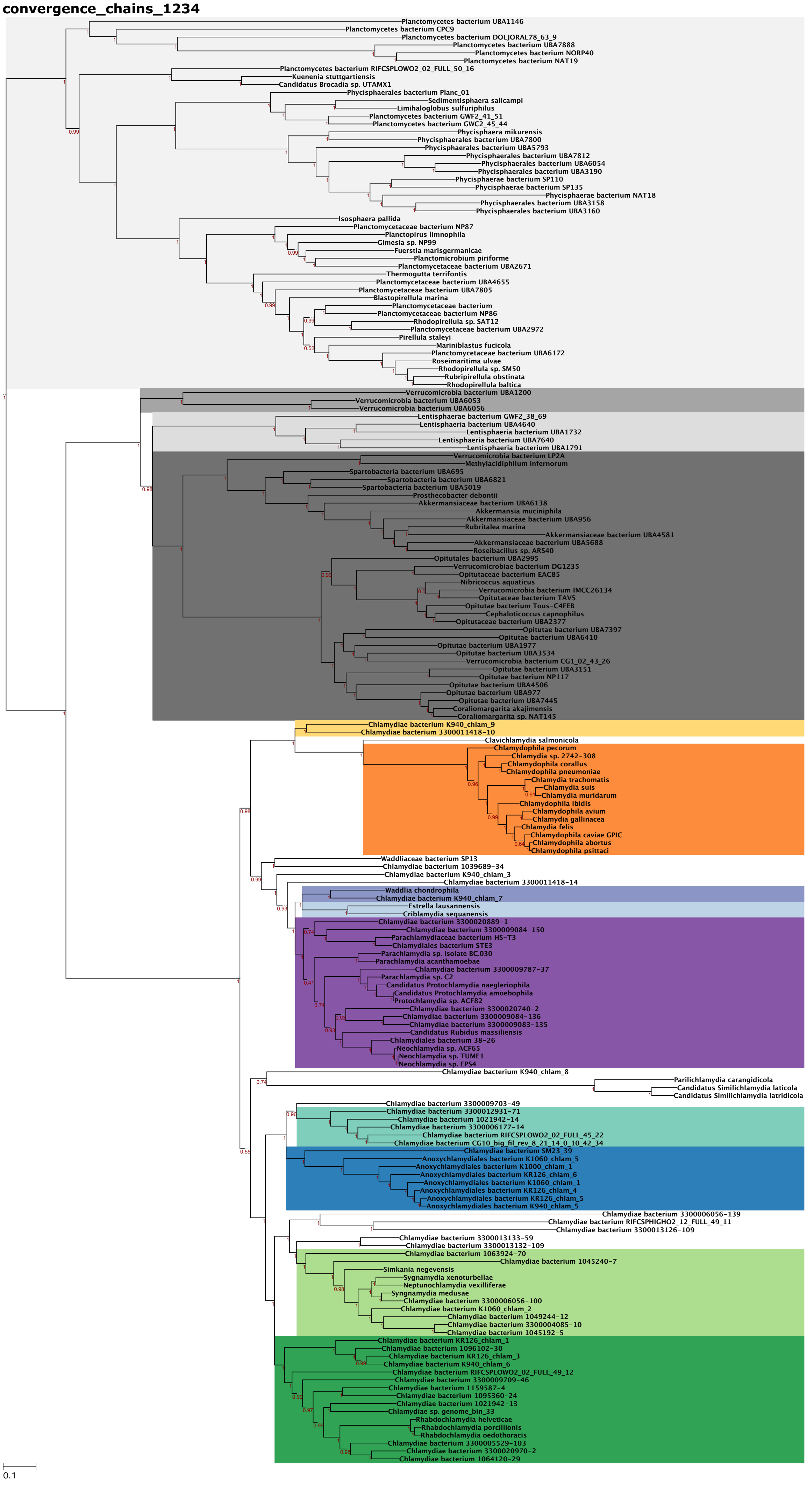

convergence_chains_24

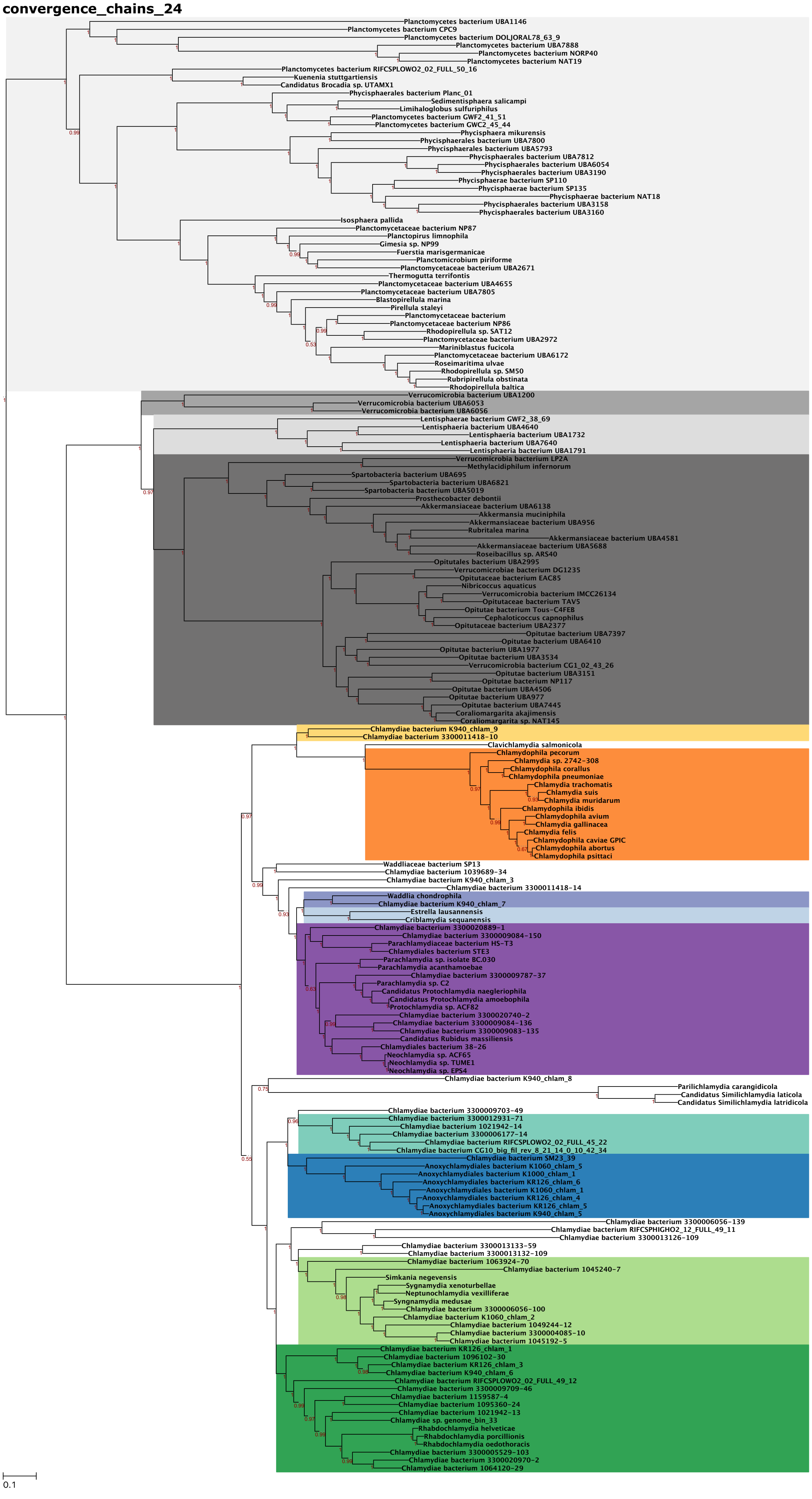

183taxa_50perc_pruned

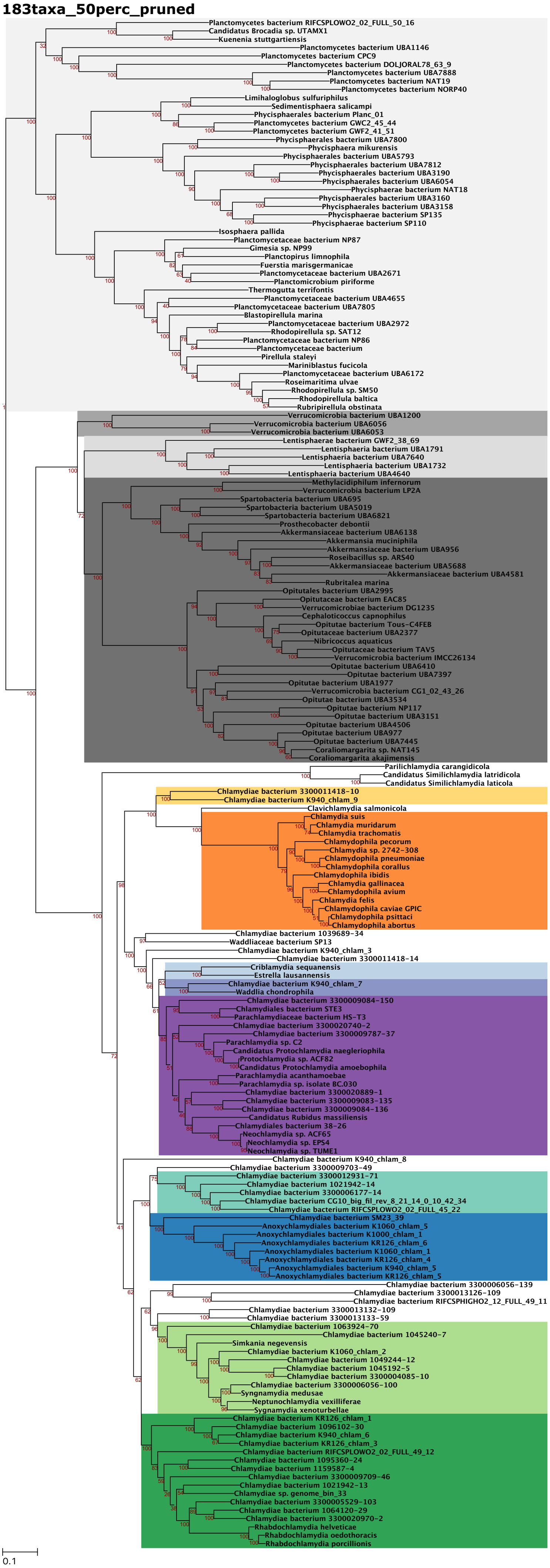

184taxa_0perc_pruned_original

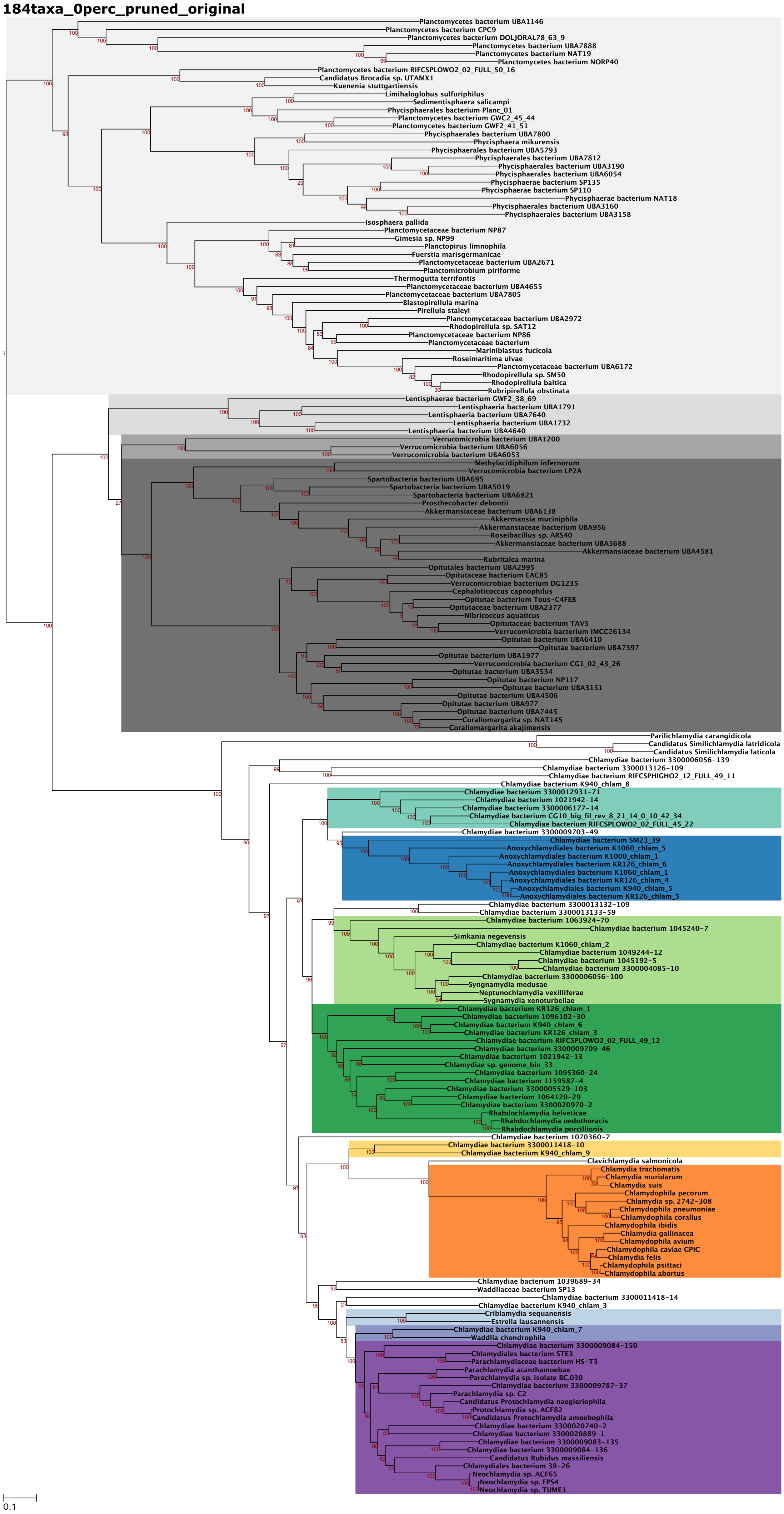

184taxa_0perc_pruned_original

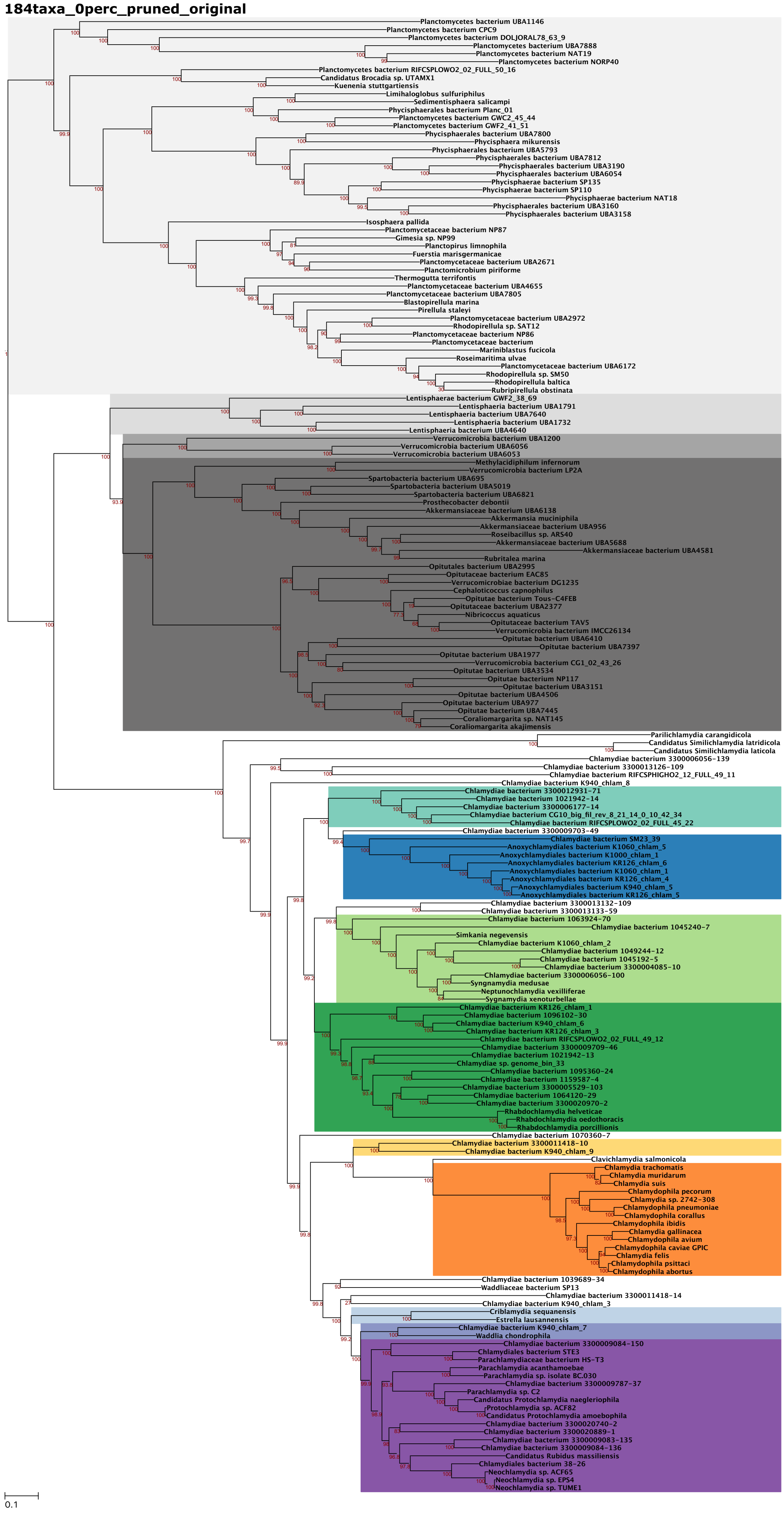

chain1

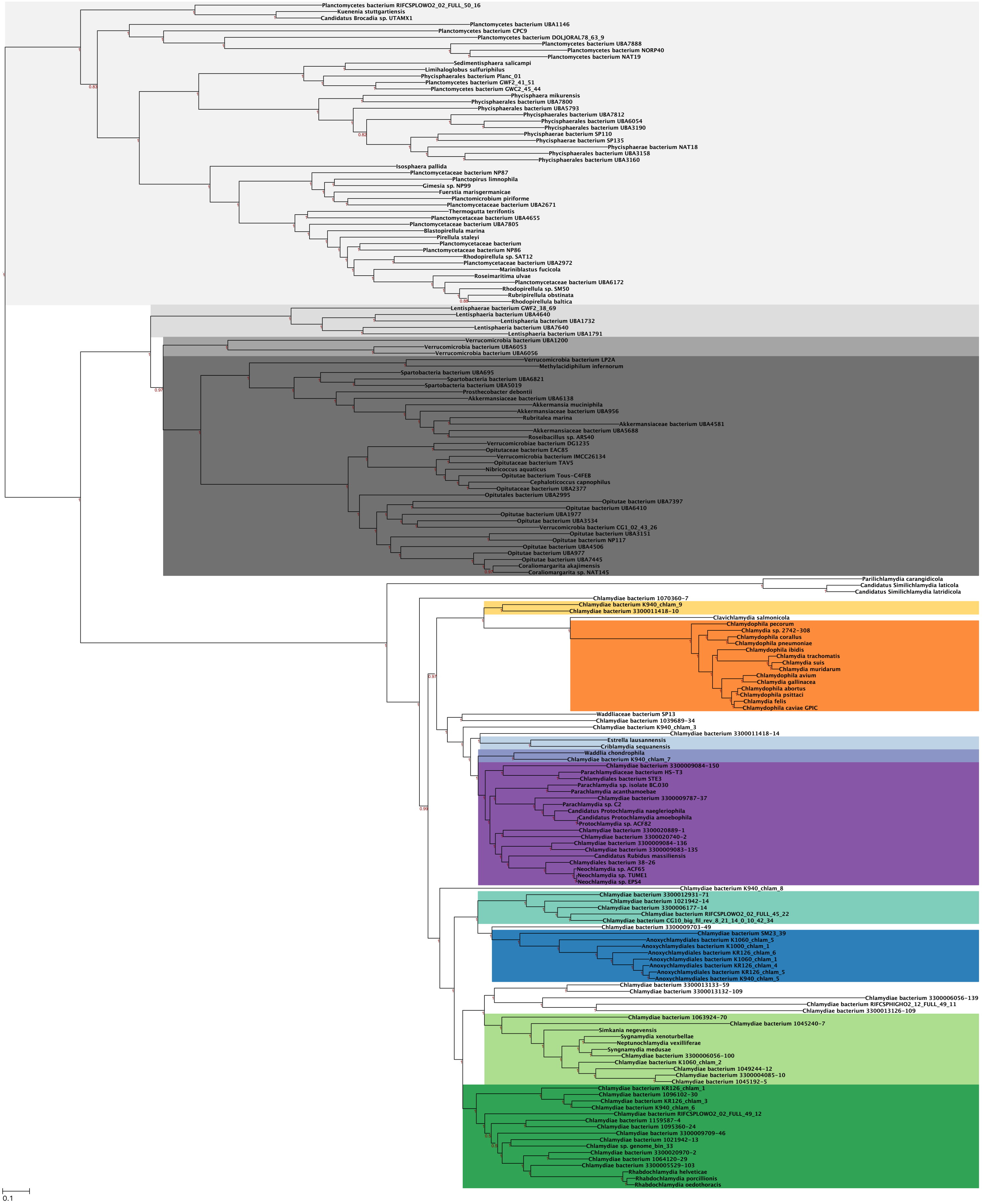
Planctomycetes bacterium RIFCSPLOWO2_02_FULL_50_16
Kuenenia stuttgartiensis
Candidatus Brocadia sp. UTAMX1
Planctomycetes bacterium UBA1146
Planctomycetes bacterium CPC9
Planctomycetes bacterium DOLJORAL78_63_9
Planctomycetes bacterium UBA7888
Planctomycetes bacterium NORP40
Planctomycetes bacterium NAT19
Sedimentisphaera salicampi
Limihaloglobus sulfuriphilus
Phycisphaerales bacterium Planc_01
Planctomycetes bacterium GWF2_41_51
Planctomycetes bacterium GWC2_45_44
Phycisphaera mikurensis
Phycisphaerales bacterium UBA7800
Phycisphaerales bacterium UBA5793
Phycisphaerales bacterium UBA7812
Phycisphaerales bacterium UBA6054
Phycisphaerales bacterium UBA3190
Phycisphaerae bacterium SP110
Phycisphaerae bacterium SP135
Phycisphaerae bacterium NAT18
Phycisphaerales bacterium UBA3158
Phycisphaerales bacterium UBA3160
Isosphaera pallida
Planctomycetaceae bacterium NP87
Planctopirus limnophila
Gimesia sp. NP99
Fuerstia marisgermanicae
Planctomicrobium piriforme
Planctomycetaceae bacterium UBA2671
Thermogutta terrifontis
Planctomycetaceae bacterium UBA4655
Planctomycetaceae bacterium UBA7805
Blastopirellula marina
Pirellula staleyi
Planctomycetaceae bacterium
Planctomycetaceae bacterium NP86
Rhodopirellula sp. SAT12
Planctomycetaceae bacterium UBA2972
Mariniblastus fucicola
Roseimaritima ulvae
Planctomycetaceae bacterium UBA6172
Rhodopirellula sp. SM50
Rubripirellula obstinata
Rhodopirellula baltica
Lentisphaerae bacterium GWF2_38_69
Lentisphaeria bacterium UBA4640
Lentisphaeria bacterium UBA1732
Lentisphaeria bacterium UBA7640
Lentisphaeria bacterium UBA1791
Verrucomicrobia bacterium UBA1200
Verrucomicrobia bacterium UBA6053
Verrucomicrobia bacterium UBA6056
Verrucomicrobia bacterium LP2A
Methylacidiphilum infernorum
Spartobacteria bacterium UBA695
Spartobacteria bacterium UBA6821
Spartobacteria bacterium UBA5019
Prosthecobacter debontii
Akkermansiaceae bacterium UBA6138
Akkermansia muciniphila
Akkermansiaceae bacterium UBA956
Rubritalea marina
Akkermansiaceae bacterium UBA4581
Akkermansiaceae bacterium UBA5688
Roseibacillus sp. ARS40
Verrucomicrobiae bacterium DG1235
Opitutaceae bacterium EAC85
Verrucomicrobia bacterium IMCC26134
Opitutaceae bacterium TAV5
Nibricoccus aquaticus
Opitutae bacterium Tous-C4FEB
Cephaloticoccus capnophilus
Opitutaceae bacterium UBA2377
Opitutales bacterium UBA2995
Opitutae bacterium UBA7397
Opitutae bacterium UBA6410
Opitutae bacterium UBA1977
Opitutae bacterium UBA3534
Verrucomicrobia bacterium CG1_02_43_26
Opitutae bacterium UBA3151
Opitutae bacterium NP117
Opitutae bacterium UBA4506
Opitutae bacterium UBA977
Opitutae bacterium UBA7445
Coraliomargarita akajimensis
Coraliomargarita sp. NAT145
Parilichlamydia carangidicola
Candidatus Similichlamydia laticola
Candidatus Similichlamydia latridicola
Chlamydiae bacterium 1070360-7
Chlamydiae bacterium K940_chlam_9
Chlamydiae bacterium 3300011418-10
Clavichlamydia salmonicola
Chlamydophila pecorum
Chlamydia sp. 2742-308
Chlamydophila corallus
Chlamydophila pneumoniae
Chlamydophila ibidis
Chlamydia trachomatis
Chlamydia suis
Chlamydia muridarum
Chlamydophila avium
Chlamydia gallinacea
Chlamydophila abortus
Chlamydophila psittaci
Chlamydia felis
Chlamydophila caviae GPIC
Waddliaceae bacterium SP13
Chlamydiae bacterium 1039689-34
Chlamydiae bacterium K940_chlam_3
Chlamydiae bacterium 3300011418-14
Estrella lausannensis
Criblamydia sequanensis
Waddlia chondrophila
Chlamydiae bacterium K940_chlam_7
Chlamydiae bacterium 3300009084-150
Parachlamydiaceae bacterium HS-T3
Chlamydiales bacterium STE3
Parachlamydia sp. isolate BC.030
Parachlamydia acanthamoebae
Chlamydiae bacterium 3300009787-37
Parachlamydia sp. C2
Candidatus Protochlamydia naegleriophila
Candidatus Protochlamydia amoebophila
Protochlamydia sp. ACF82
Chlamydiae bacterium 3300020889-1
Chlamydiae bacterium 3300020740-2
Chlamydiae bacterium 3300009084-136
Chlamydiae bacterium 3300009083-135
Candidatus Rubidus massiliensis
Chlamydiales bacterium 38-26
Neochlamydia sp. ACF65
Neochlamydia sp. TUME1
Neochlamydia sp. EPS4
Chlamydiae bacterium K940_chlam_8
Chlamydiae bacterium 3300012931-71
Chlamydiae bacterium 1021942-14
Chlamydiae bacterium 3300006177-14
Chlamydiae bacterium RIFCSPLOWO2_02_FULL_45_22
Chlamydiae bacterium CG10_big_fil_rev_8_21_14_0_10_42_34
Chlamydiae bacterium 3300009703-49
Chlamydiae bacterium SM23_39
Anoxychlamydiales bacterium K1060_chlam_5
Anoxychlamydiales bacterium K1000_chlam_1
Anoxychlamydiales bacterium KR126_chlam_6
Anoxychlamydiales bacterium K1060_chlam_1
Anoxychlamydiales bacterium KR126_chlam_4
Anoxychlamydiales bacterium KR126_chlam_5
Anoxychlamydiales bacterium K940_chlam_5
Chlamydiae bacterium 3300013133-59
Chlamydiae bacterium 3300013132-109
Chlamydiae bacterium 3300006056-139
Chlamydiae bacterium RIFCSPHIGHO2_12_FULL_49_11
Chlamydiae bacterium 3300013126-109
Chlamydiae bacterium 1063924-70
Chlamydiae bacterium 1045240-7
Simkania negevensis
Syngnamydia xenoturbellae
Neptunochlamydia vexilliferae
Syngnamydia medusae
Chlamydiae bacterium 3300006056-100
Chlamydiae bacterium K1060_chlam_2
Chlamydiae bacterium 1049244-12
Chlamydiae bacterium 3300004085-10
Chlamydiae bacterium 1045192-5
Chlamydiae bacterium KR126_chlam_1
Chlamydiae bacterium 1096102-30
Chlamydiae bacterium KR126_chlam_3
Chlamydiae bacterium K940_chlam_6
Chlamydiae bacterium RIFCSPLOWO2_02_FULL_49_12
Chlamydiae bacterium 1159587-4
Chlamydiae bacterium 1095360-24
Chlamydiae bacterium 3300009709-46
Chlamydiae bacterium 1021942-13
Chlamydia sp. genome_bin_33
Chlamydiae bacterium 3300020970-2
Chlamydiae bacterium 1064120-29
Chlamydiae bacterium 3300005529-103
Rhabdochlamydia helveticae
Rhabdochlamydia porcellionis
Rhabdochlamydia oedothoracis
0.1

chain2

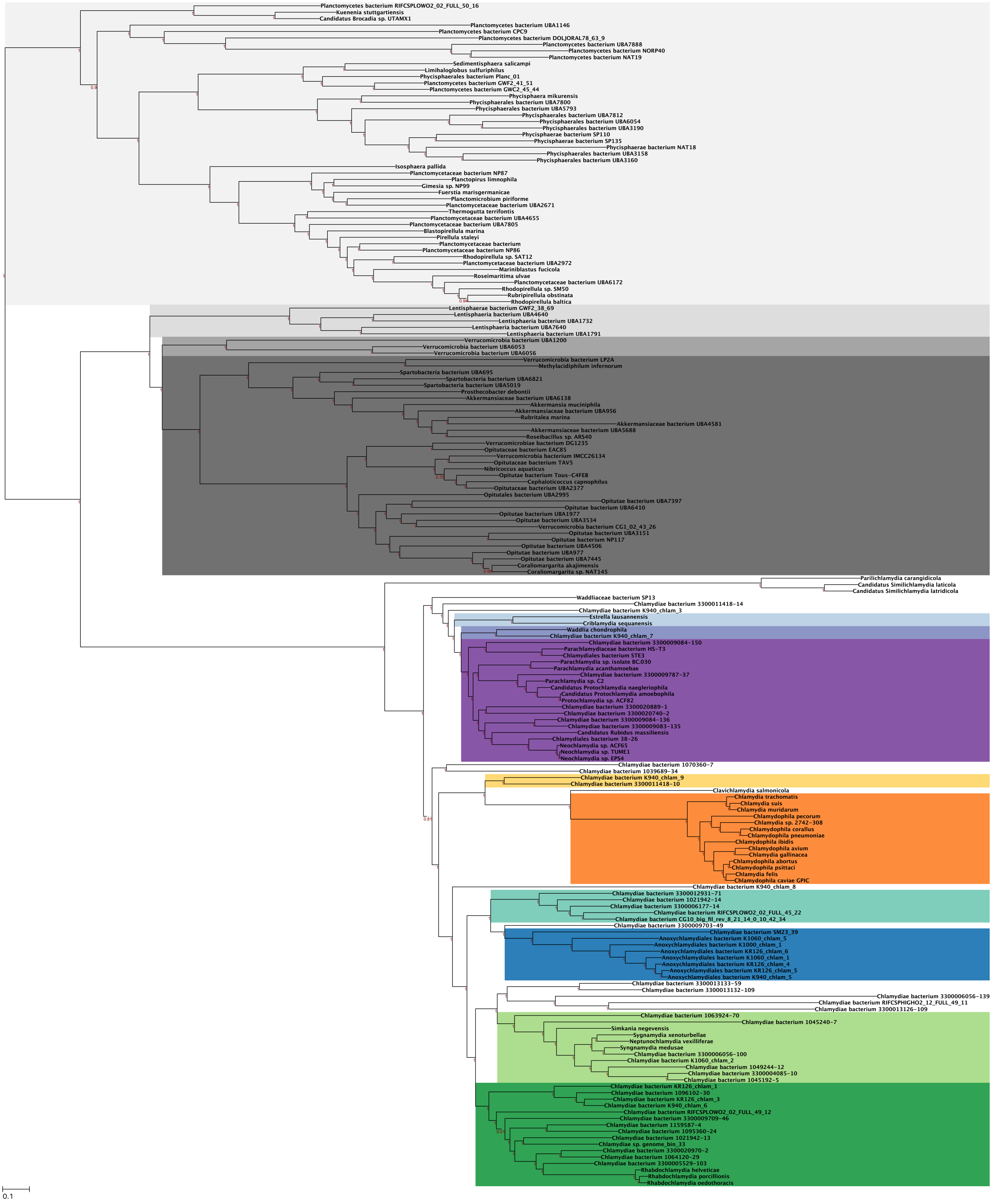

Planctomycetes bacterium RIFCSPLOWO2_02_FULL_50_16
Kuenenia stuttgartiensis
Candidatus Brocadia sp. UTAMX1
Planctomycetes bacterium UBA1146
Planctomycetes bacterium CPC9
Planctomycetes bacterium DOLJORAL78_63_9
Planctomycetes bacterium UBA7888
Planctomycetes bacterium NORP40
Planctomycetes bacterium NAT19
Sedimentisphaera salicampi
Limihaloglobus sulfuriphilus
Phycisphaerales bacterium Planc_01
Planctomycetes bacterium GWF2_41_51
Planctomycetes bacterium GWC2_45_44
Phycisphaera mikurensis
Phycisphaerales bacterium UBA7800
Phycisphaerales bacterium UBA5793
Phycisphaerales bacterium UBA7812
Phycisphaerales bacterium UBA6054
Phycisphaerales bacterium UBA3190
Phycisphaerae bacterium SP110
Phycisphaerae bacterium SP135
Phycisphaerae bacterium NAT18
Phycisphaerales bacterium UBA3158
Phycisphaerales bacterium UBA3160
Isosphaera pallida
Planctomycetaceae bacterium NP87
Planctopirus limnophila
Gimesia sp. NP99
Fuerstia marisgermanicae
Planctomicrobium piriforme
Planctomycetaceae bacterium UBA2671
Thermogutta terrifontis
Planctomycetaceae bacterium UBA4655
Planctomycetaceae bacterium UBA7805
Blastopirellula marina
Pirellula staleyi
Planctomycetaceae bacterium
Planctomycetaceae bacterium NP86
Rhodopirellula sp. SAT12
Planctomycetaceae bacterium UBA2972
Mariniblastus fucicola
Roseimaritima ulvae
Planctomycetaceae bacterium UBA6172
Rhodopirellula sp. SM50
Rubripirellula obstinata
Rhodopirellula baltica
Lentisphaerae bacterium GWF2_38_69
Lentisphaeria bacterium UBA4640
Lentisphaeria bacterium UBA1732
Lentisphaeria bacterium UBA7640
Lentisphaeria bacterium UBA1791
Verrucomicrobia bacterium UBA1200
Verrucomicrobia bacterium UBA6053
Verrucomicrobia bacterium UBA6056
Verrucomicrobia bacterium LP2A
Methylacidiphilum infernorum
Spartobacteria bacterium UBA695
Spartobacteria bacterium UBA6821
Spartobacteria bacterium UBA5019
Prosthecobacter debontii
Akkermansiaceae bacterium UBA6138
Akkermansia muciniphila
Akkermansiaceae bacterium UBA956
Rubritalea marina
Akkermansiaceae bacterium UBA4581
Akkermansiaceae bacterium UBA5688
Roseibacillus sp. ARS40
Verrucomicrobiae bacterium DG1235
Opitutaceae bacterium EAC85
Verrucomicrobia bacterium IMCC26134
Opitutaceae bacterium TAV5
Nibricoccus aquaticus
Opitutae bacterium Tous-C4FEB
Cephaloticoccus capnophilus
Opitutaceae bacterium UBA2377
Opitutales bacterium UBA2995
Opitutae bacterium UBA7397
Opitutae bacterium UBA6410
Opitutae bacterium UBA1977
Opitutae bacterium UBA3534
Verrucomicrobia bacterium CG1_02_43_26
Opitutae bacterium UBA3151
Opitutae bacterium NP117
Opitutae bacterium UBA4506
Opitutae bacterium UBA977
Opitutae bacterium UBA7445
Coraliomargarita akajimensis
Coraliomargarita sp. NAT145
Parilichlamydia carangidicola
Candidatus Similichlamydia laticola
Candidatus Similichlamydia latridicola
Waddliaceae bacterium SP13
Chlamydiae bacterium 3300011418-14
Chlamydiae bacterium K940_chlam_3
Estrella lausannensis
Criblamydia sequanensis
Waddlia chondrophila
Chlamydiae bacterium K940_chlam_7
Chlamydiae bacterium 3300009084-150
Parachlamydiaceae bacterium HS-T3
Chlamydiales bacterium STE3
Parachlamydia sp. isolate BC.030
Parachlamydia acanthamoebae
Chlamydiae bacterium 3300009787-37
Parachlamydia sp. C2
Candidatus Protochlamydia naegleriophila
Candidatus Protochlamydia amoebophila
Protochlamydia sp. ACF82
Chlamydiae bacterium 3300020889-1
Chlamydiae bacterium 3300020740-2
Chlamydiae bacterium 3300009084-136
Chlamydiae bacterium 3300009083-135
Candidatus Rubidus massiliensis
Chlamydiales bacterium 38-26
Neochlamydia sp. ACF65
Neochlamydia sp. TUME1
Neochlamydia sp. EPS4
Chlamydiae bacterium 1070360-7
Chlamydiae bacterium 1039689-34
Chlamydiae bacterium K940_chlam_9
Chlamydiae bacterium 3300011418-10
Clavichlamydia salmonicola
Chlamydia trachomatis
Chlamydia suis
Chlamydia muridarum
Chlamydophila pecorum
Chlamydia sp. 2742-308
Chlamydophila corallus
Chlamydophila pneumoniae
Chlamydophila ibidis
Chlamydophila avium
Chlamydia gallinacea
Chlamydophila abortus
Chlamydophila psittaci
Chlamydia felis
Chlamydophila caviae GPIC
Chlamydiae bacterium K940_chlam_8
Chlamydiae bacterium 3300012931-71
Chlamydiae bacterium 1021942-14
Chlamydiae bacterium 3300006177-14
Chlamydiae bacterium RIFCSPLOWO2_02_FULL_45_22
Chlamydiae bacterium CG10_big_fil_rev_8_21_14_0_10_42_34
Chlamydiae bacterium 3300009703-49
Chlamydiae bacterium SM23_39
Anoxychlamydiales bacterium K1060_chlam_5
Anoxychlamydiales bacterium K1000_chlam_1
Anoxychlamydiales bacterium KR126_chlam_6
Anoxychlamydiales bacterium K1060_chlam_1
Anoxychlamydiales bacterium KR126_chlam_4
Anoxychlamydiales bacterium KR126_chlam_5
Anoxychlamydiales bacterium K940_chlam_5
Chlamydiae bacterium 3300013133-59
Chlamydiae bacterium 3300013132-109
Chlamydiae bacterium 3300006056-139
Chlamydiae bacterium RIFCSPHIGHO2_12_FULL_49_11
Chlamydiae bacterium 3300013126-109
Chlamydiae bacterium 1063924-70
Chlamydiae bacterium 1045240-7
Simkania negevensis
Sygnamydia xenoturbellae
Neptunochlamydia vexilliferae
Syngnamydia medusae
Chlamydiae bacterium 3300006056-100
Chlamydiae bacterium K1060_chlam_2
Chlamydiae bacterium 1049244-12
Chlamydiae bacterium 3300004085-10
Chlamydiae bacterium 1045192-5
Chlamydiae bacterium KR126_chlam_1
Chlamydiae bacterium 1096102-30
Chlamydiae bacterium KR126_chlam_3
Chlamydiae bacterium K940_chlam_6
Chlamydiae bacterium RIFCSPLOWO2_02_FULL_49_12
Chlamydiae bacterium 3300009709-46
Chlamydiae bacterium 1159587-4
Chlamydiae bacterium 1095360-24
Chlamydiae bacterium 1021942-13
Chlamydiae sp. genome_bin_33
Chlamydiae bacterium 3300020970-2
Chlamydiae bacterium 1064120-29
Chlamydiae bacterium 3300005529-103
Rhabdochlamydia helveticae
Rhabdochlamydia porcillionis
Rhabdochlamydia oedothoracis
0.1

chain3

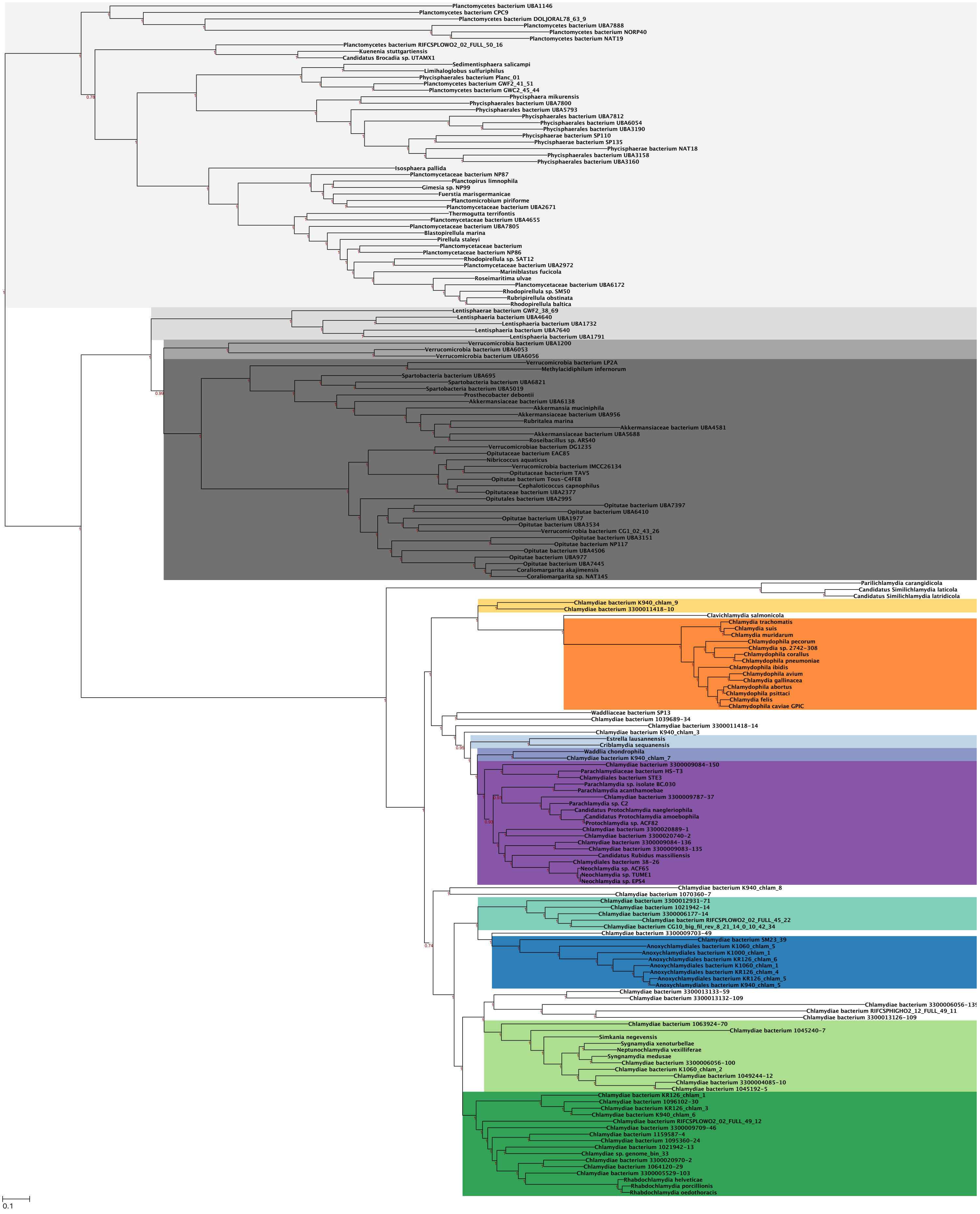

Planctomycetes bacterium UBA1146
Planctomycetes bacterium CPC9
Planctomycetes bacterium DOLJORAL78_63_9
Planctomycetes bacterium UBA7888
Planctomycetes bacterium NORP40
Planctomycetes bacterium NAT19
Planctomycetes bacterium RIFCSPLOWO2_02_FULL_50_16
Kuenenia stuttgartiensis
Candidatus Brocadia sp. UTAMX1
Sedimentisphaera salicampi
Limihaloglobus sulfuriphilus
Phycisphaerales bacterium Planc_01
Planctomycetes bacterium GWF2_41_51
Planctomycetes bacterium GWC2_45_44
Phycisphaera mikurensis
Phycisphaerales bacterium UBA7800
Phycisphaerales bacterium UBA5793
Phycisphaerales bacterium UBA7812
Phycisphaerales bacterium UBA6054
Phycisphaerales bacterium UBA3190
Phycisphaerae bacterium SP110
Phycisphaerae bacterium SP135
Phycisphaerae bacterium NAT18
Phycisphaerales bacterium UBA3158
Phycisphaerales bacterium UBA3160
Isosphaera pallida
Planctomycetaceae bacterium NP87
Planctopirus limnophila
Gimesia sp. NP99
Fuerstia marisgermanicae
Planctomicrobium piriforme
Planctomycetaceae bacterium UBA2671
Thermogutta terrifontis
Planctomycetaceae bacterium UBA4655
Planctomycetaceae bacterium UBA7805
Blastopirellula marina
Pirellula staleyi
Planctomycetaceae bacterium
Planctomycetaceae bacterium NP86
Rhodopirellula sp. SAT12
Planctomycetaceae bacterium UBA2972
Mariniblastus fucicola
Roseimaritima ulvae
Planctomycetaceae bacterium UBA6172
Rhodopirellula sp. SM50
Rubripirellula obstinata
Rhodopirellula baltica
Lentisphaerae bacterium GWF2_38_69
Lentisphaeria bacterium UBA4640
Lentisphaeria bacterium UBA1732
Lentisphaeria bacterium UBA7640
Lentisphaeria bacterium UBA1791
Verrucomicrobia bacterium UBA1200
Verrucomicrobia bacterium UBA6053
Verrucomicrobia bacterium UBA6056
Verrucomicrobia bacterium LP2A
Methylacidiphilum infernorum
Spartobacteria bacterium UBA695
Spartobacteria bacterium UBA6821
Spartobacteria bacterium UBA5019
Prosthecobacter debontii
Akkermansiaceae bacterium UBA6138
Akkermansia muciniphila
Akkermansiaceae bacterium UBA956
Rubritalea marina
Akkermansiaceae bacterium UBA4581
Akkermansiaceae bacterium UBA5688
Roseibacillus sp. ARS40
Verrucomicrobiae bacterium DG1235
Opitutaceae bacterium EAC85
Nibricoccus aquaticus
Verrucomicrobia bacterium IMCC26134
Opitutaceae bacterium TAV5
Opitutae bacterium Tous-C4FEB
Cephaloticoccus capnophilus
Opitutaceae bacterium UBA2377
Opitutales bacterium UBA2995
Opitutae bacterium UBA7397
Opitutae bacterium UBA6410
Opitutae bacterium UBA1977
Opitutae bacterium UBA3534
Verrucomicrobia bacterium CG1_02_43_26
Opitutae bacterium UBA3151
Opitutae bacterium NP117
Opitutae bacterium UBA4506
Opitutae bacterium UBA977
Opitutae bacterium UBA7445
Coraliomargarita akajimensis
Coraliomargarita sp. NAT145
Parilichlamydia carangidicola
Candidatus Similichlamydia laticola
Candidatus Similichlamydia latridicola
Chlamydiae bacterium K940_chlam_9
Chlamydiae bacterium 3300011418-10
Clavichlamydia salmonicola
Chlamydia trachomatis
Chlamydia suis
Chlamydia muridarum
Chlamydophila pecorum
Chlamydia sp. 2742-308
Chlamydophila corallus
Chlamydophila pneumoniae
Chlamydophila ibidis
Chlamydophila avium
Chlamydia gallinacea
Chlamydophila abortus
Chlamydophila psittaci
Chlamydia felis
Chlamydophila caviae GPIC
Waddliaceae bacterium SP13
Chlamydiae bacterium 1039689-34
Chlamydiae bacterium 3300011418-14
Chlamydiae bacterium K940_chlam_3
Estrella lausannensis
Criblamydia sequanensis
Waddlia chondrophila
Chlamydiae bacterium K940_chlam_7
Chlamydiae bacterium 3300009084-150
Parachlamydiaceae bacterium HS-T3
Chlamydiales bacterium STE3
Parachlamydia sp. isolate BC.030
Parachlamydia acanthamoebae
Chlamydiae bacterium 3300009787-37
Parachlamydia sp. C2
Candidatus Protochlamydia naegleriophila
Candidatus Protochlamydia amoebophila
Protochlamydia sp. ACF82
Chlamydiae bacterium 3300020889-1
Chlamydiae bacterium 3300020740-2
Chlamydiae bacterium 3300009084-136
Chlamydiae bacterium 3300009083-135
Candidatus Rubidus massiliensis
Chlamydiales bacterium 38-26
Neochlamydia sp. ACF65
Neochlamydia sp. TUME1
Neochlamydia sp. EPS4
Chlamydiae bacterium K940_chlam_8
Chlamydiae bacterium 1070360-7
Chlamydiae bacterium 3300012931-71
Chlamydiae bacterium 1021942-14
Chlamydiae bacterium 3300006177-14
Chlamydiae bacterium RIFCSPLOWO2_02_FULL_45_22
Chlamydiae bacterium CG10_big_fil_rev_8_21_14_0_10_42_34
Chlamydiae bacterium 3300009703-49
Chlamydiae bacterium SM23_39
Anoxychlamydiales bacterium K1060_chlam_5
Anoxychlamydiales bacterium K1000_chlam_1
Anoxychlamydiales bacterium KR126_chlam_6
Anoxychlamydiales bacterium K1060_chlam_1
Anoxychlamydiales bacterium KR126_chlam_4
Anoxychlamydiales bacterium KR126_chlam_5
Anoxychlamydiales bacterium K940_chlam_5
Chlamydiae bacterium 3300013133-59
Chlamydiae bacterium 3300013132-109
Chlamydiae bacterium 3300006056-13
Chlamydiae bacterium RIFCSPHIGHO2_12_FULL_49_11
Chlamydiae bacterium 3300013126-109
Chlamydiae bacterium 1063924-70
Chlamydiae bacterium 1045240-7
Simkania negevensis
Sygnamydia xenoturbellae
Neptunochlamydia vexilliferae
Syngnamydia medusae
Chlamydiae bacterium 3300006056-100
Chlamydiae bacterium K1060_chlam_2
Chlamydiae bacterium 1049244-12
Chlamydiae bacterium 3300004085-10
Chlamydiae bacterium 1045192-5
Chlamydiae bacterium KR126_chlam_1
Chlamydiae bacterium 1096102-30
Chlamydiae bacterium KR126_chlam_3
Chlamydiae bacterium K940_chlam_6
Chlamydiae bacterium RIFCSPLOWO2_02_FULL_49_12
Chlamydiae bacterium 3300009709-46
Chlamydiae bacterium 1159587-4
Chlamydiae bacterium 1095360-24
Chlamydiae bacterium 1021942-13
Chlamydiae sp. genome_bin_33
Chlamydiae bacterium 3300020970-2
Chlamydiae bacterium 1064120-29
Chlamydiae bacterium 3300005529-103
Rhabdochlamydia helveticae
Rhabdochlamydia porcellionis
Rhabdochlamydia oedothoracis
0.1

chain4

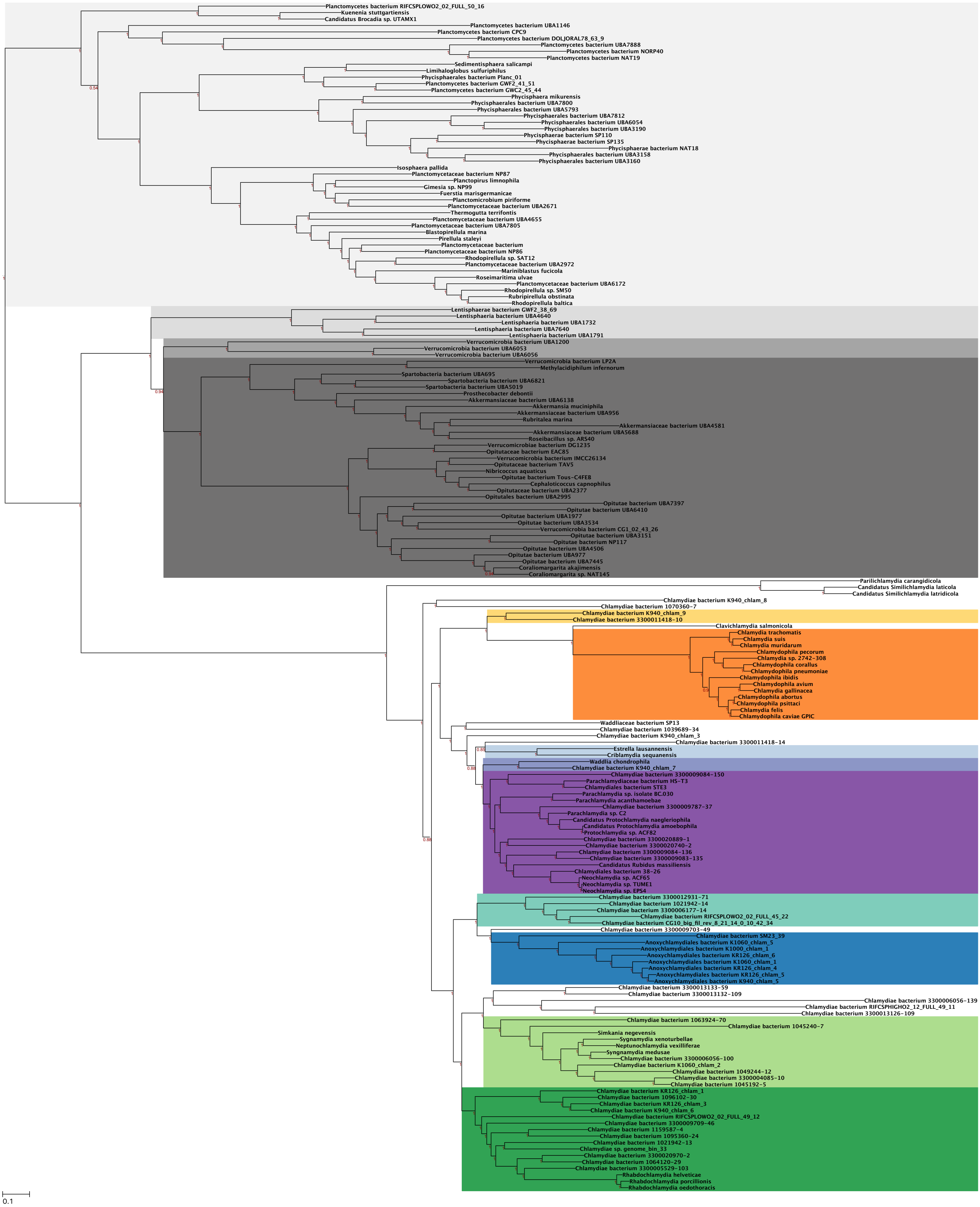

Planctomycetes bacterium RIFCSPLOWO2_02_FULL_50_16
Kuenenia stuttgartiensis
Candidatus Brocadia sp. UTAMX1
Planctomycetes bacterium UBA1146
Planctomycetes bacterium CPC9
Planctomycetes bacterium DOLJORAL78_63_9
Planctomycetes bacterium UBA7888
Planctomycetes bacterium NORP40
Planctomycetes bacterium NAT19
Sedimentisphaera salicampi
Limihaloglobus sulfuriphilus
Phycisphaerales bacterium Planc_01
Planctomycetes bacterium GWF2_41_51
Planctomycetes bacterium GWC2_45_44
Phycisphaera mikurensis
Phycisphaerales bacterium UBA7800
Phycisphaerales bacterium UBA5793
Phycisphaerales bacterium UBA7812
Phycisphaerales bacterium UBA6054
Phycisphaerales bacterium UBA3190
Phycisphaerae bacterium SP110
Phycisphaerae bacterium SP135
Phycisphaerae bacterium NAT18
Phycisphaerales bacterium UBA3158
Phycisphaerales bacterium UBA3160
Isosphaera pallida
Planctomycetaceae bacterium NP87
Planctopirus limnophila
Gimesia sp. NP99
Fuerstia marisgermanicae
Planctomicrobium piriforme
Planctomycetaceae bacterium UBA2671
Thermogutta terrifontis
Planctomycetaceae bacterium UBA4655
Planctomycetaceae bacterium UBA7805
Blastopirellula marina
Pirellula staleyi
Planctomycetaceae bacterium
Planctomycetaceae bacterium NP86
Rhodopirellula sp. SAT12
Planctomycetaceae bacterium UBA2972
Mariniblastus fucicola
Roseimaritima ulvae
Planctomycetaceae bacterium UBA6172
Rhodopirellula sp. SM50
Rubripirellula obstinata
Rhodopirellula baltica
Lentisphaerae bacterium GWF2_38_69
Lentisphaeria bacterium UBA4640
Lentisphaeria bacterium UBA1732
Lentisphaeria bacterium UBA7640
Lentisphaeria bacterium UBA1791
Verrucomicrobia bacterium UBA1200
Verrucomicrobia bacterium UBA6053
Verrucomicrobia bacterium UBA6056
Verrucomicrobia bacterium LP2A
Methylacidiphilum infernorum
Spartobacteria bacterium UBA695
Spartobacteria bacterium UBA6821
Spartobacteria bacterium UBA5019
Prosthecobacter debontii
Akkermansiaceae bacterium UBA6138
Akkermansia muciniphila
Akkermansiaceae bacterium UBA956
Rubritalea marina
Akkermansiaceae bacterium UBA4581
Akkermansiaceae bacterium UBA5688
Roseibacillus sp. ARS40
Verrucomicrobiae bacterium DG1235
Opitutaceae bacterium EAC85
Verrucomicrobia bacterium IMCC26134
Opitutaceae bacterium TAV5
Nibricoccus aquaticus
Opitutae bacterium Tous-C4FEB
Cephaloticoccus capnophilus
Opitutaceae bacterium UBA2377
Opitutales bacterium UBA2995
Opitutae bacterium UBA7397
Opitutae bacterium UBA6410
Opitutae bacterium UBA1977
Opitutae bacterium UBA3534
Verrucomicrobia bacterium CG1_02_43_26
Opitutae bacterium UBA3151
Opitutae bacterium NP117
Opitutae bacterium UBA4506
Opitutae bacterium UBA977
Opitutae bacterium UBA7445
Coraliomargarita akajimensis
Coraliomargarita sp. NAT145
Parilichlamydia carangidicola
Candidatus Similichlamydia laticola
Candidatus Similichlamydia latridicola
Chlamydiae bacterium K940_chlam_8
Chlamydiae bacterium 1070360-7
Chlamydiae bacterium K940_chlam_9
Chlamydiae bacterium 3300011418-10
Clavichlamydia salmonicola
Chlamydia trachomatis
Chlamydia suis
Chlamydia muridarum
Chlamydophila pecorum
Chlamydia sp. 2742-308
Chlamydophila corallus
Chlamydophila pneumoniae
Chlamydophila ibidis
Chlamydophila avium
Chlamydia gallinacea
Chlamydophila abortus
Chlamydophila psittaci
Chlamydia felis
Chlamydophila caviae GPIC
Waddliaceae bacterium SP13
Chlamydiae bacterium 1039689-34
Chlamydiae bacterium K940_chlam_3
Chlamydiae bacterium 3300011418-14
Estrella lausannensis
Criblamydia sequanensis
Waddlia chondrophila
Chlamydiae bacterium K940_chlam_7
Chlamydiae bacterium 3300009084-150
Parachlamydiaceae bacterium HS-T3
Chlamydiales bacterium STE3
Parachlamydia sp. isolate BC.030
Parachlamydia acanthamoebae
Chlamydiae bacterium 3300009787-37
Parachlamydia sp. C2
Candidatus Protochlamydia naegleriophila
Candidatus Protochlamydia amoebophila
Protochlamydia sp. ACF82
Chlamydiae bacterium 3300020889-1
Chlamydiae bacterium 3300020740-2
Chlamydiae bacterium 3300009084-136
Chlamydiae bacterium 3300009083-135
Candidatus Rubidus massiliensis
Chlamydiales bacterium 38-26
Neochlamydia sp. ACF65
Neochlamydia sp. TUME1
Neochlamydia sp. EPS4
Chlamydiae bacterium 3300012931-71
Chlamydiae bacterium 1021942-14
Chlamydiae bacterium 3300006177-14
Chlamydiae bacterium RIFCSPLOWO2_02_FULL_45_22
Chlamydiae bacterium CG10_big_fil_rev_8_21_14_0_10_42_34
Chlamydiae bacterium 3300009703-49
Chlamydiae bacterium SM23_39
Anoxychlamydiales bacterium K1060_chlam_5
Anoxychlamydiales bacterium K1000_chlam_1
Anoxychlamydiales bacterium KR126_chlam_6
Anoxychlamydiales bacterium K1060_chlam_1
Anoxychlamydiales bacterium KR126_chlam_4
Anoxychlamydiales bacterium KR126_chlam_5
Anoxychlamydiales bacterium K940_chlam_5
Chlamydiae bacterium 3300013133-59
Chlamydiae bacterium 3300013132-109
Chlamydiae bacterium 3300006056-139
Chlamydiae bacterium RIFCSPHIGHO2_12_FULL_49_11
Chlamydiae bacterium 3300013126-109
Chlamydiae bacterium 1063924-70
Chlamydiae bacterium 1045240-7
Simkania negevensis
Sygnamydia xenoturbellae
Neptunochlamydia vexilliferae
Syngnamydia medusae
Chlamydiae bacterium 3300006056-100
Chlamydiae bacterium K1060_chlam_2
Chlamydiae bacterium 1049244-12
Chlamydiae bacterium 3300004085-10
Chlamydiae bacterium 1045192-5
Chlamydiae bacterium KR126_chlam_1
Chlamydiae bacterium 1096102-30
Chlamydiae bacterium KR126_chlam_3
Chlamydiae bacterium K940_chlam_6
Chlamydiae bacterium RIFCSPLOWO2_02_FULL_49_12
Chlamydiae bacterium 3300009709-46
Chlamydiae bacterium 1159587-4
Chlamydiae bacterium 1095360-24
Chlamydiae bacterium 1021942-13
Chlamydiae sp. genome_bin_33
Chlamydiae bacterium 3300020970-2
Chlamydiae bacterium 1064120-29
Chlamydiae bacterium 3300005529-103
Rhabdochlamydia helveticae
Rhabdochlamydia porcellionis
Rhabdochlamydia oedothoracis
0.1

convergence_1_2_3_4

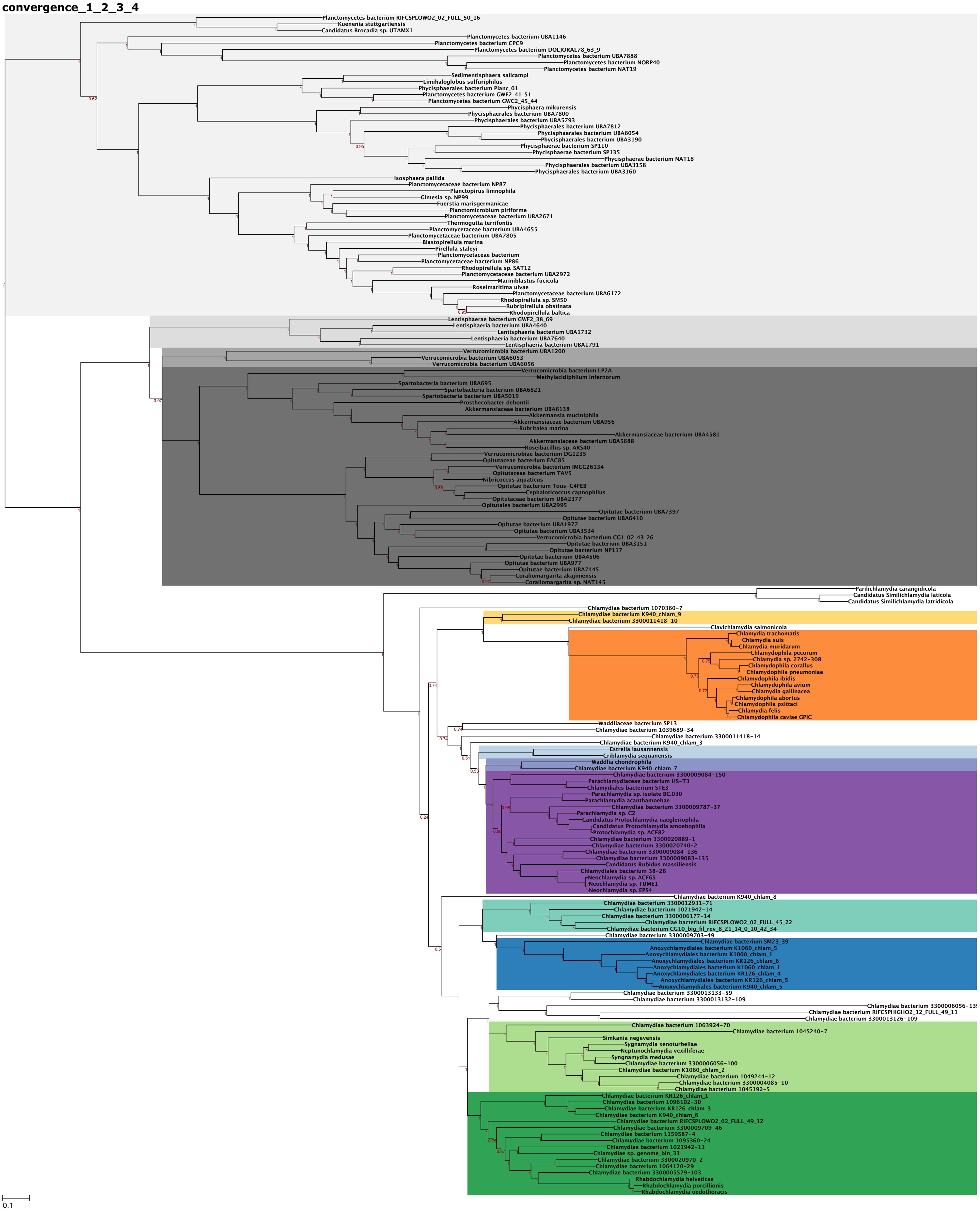

184taxa_10perc_pruned

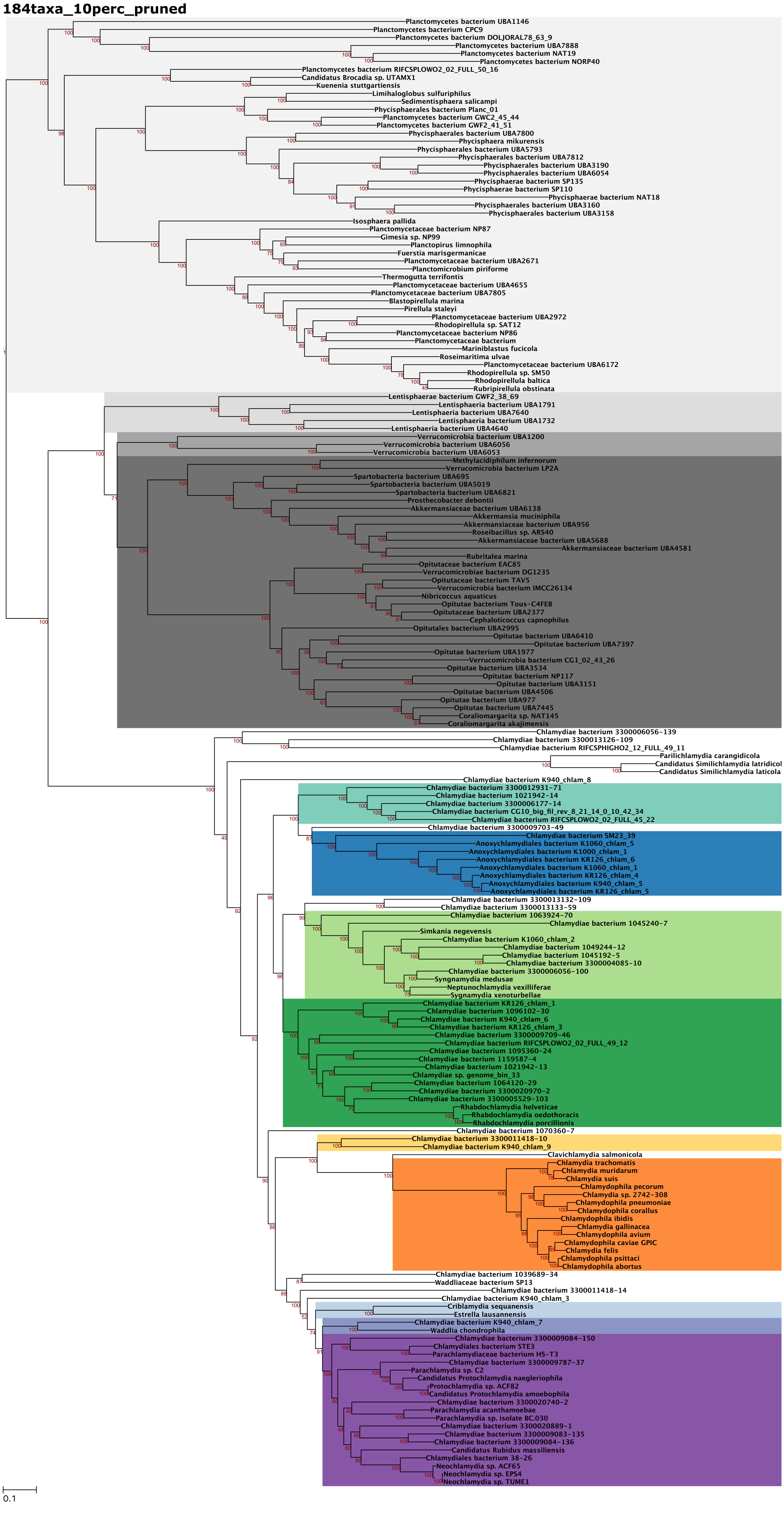

184taxa_20perc_pruned

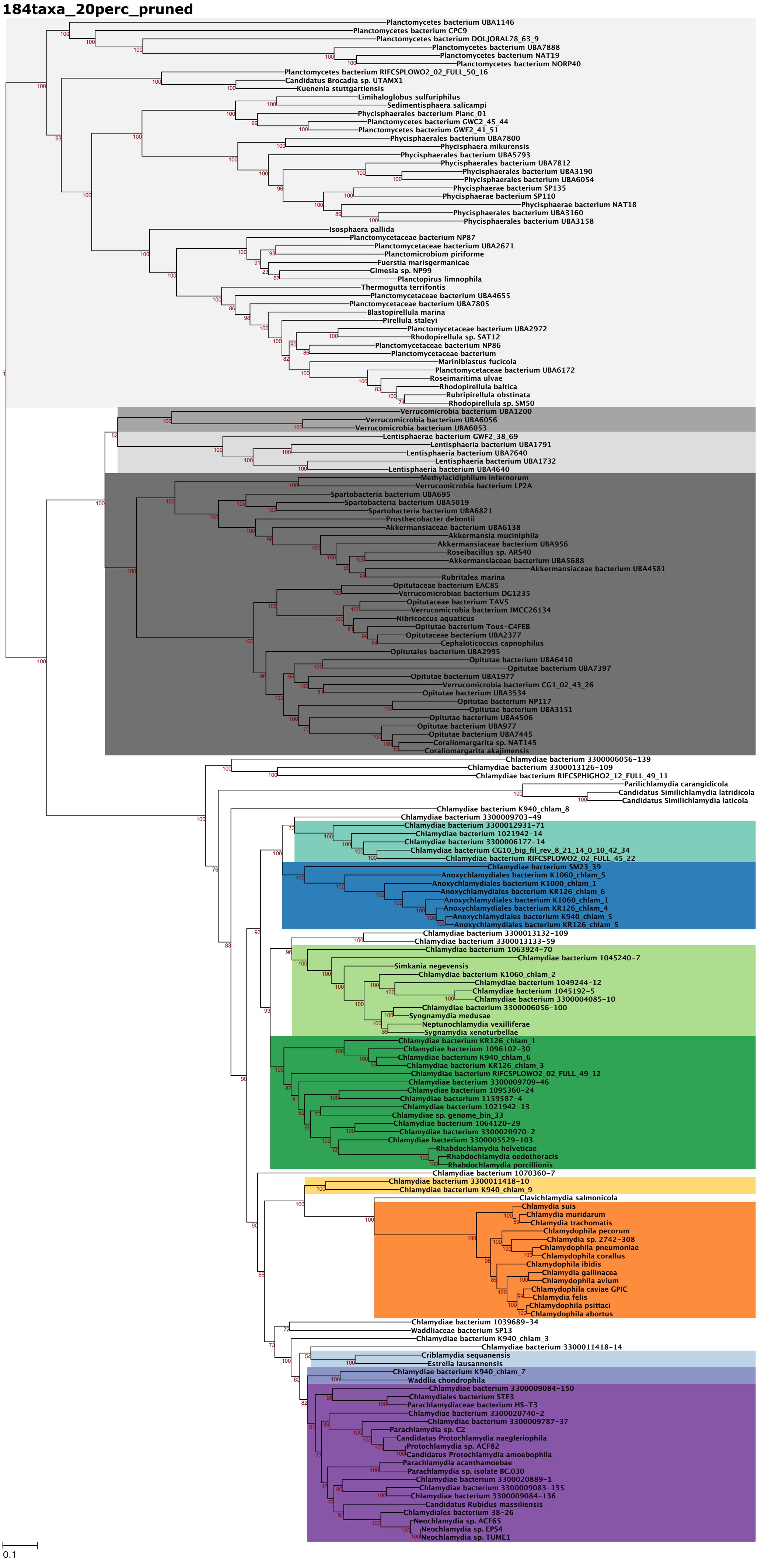

184taxa_30perc_pruned

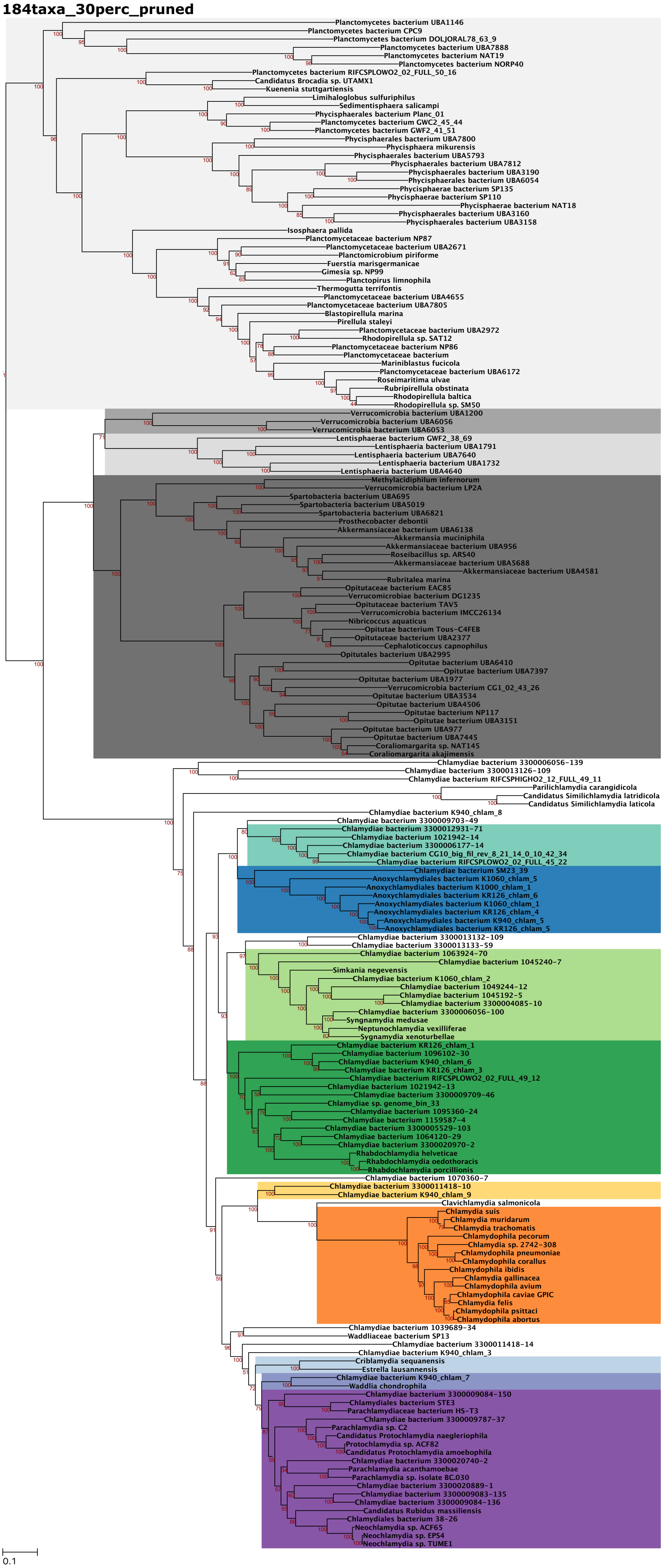

184taxa_40perc_pruned

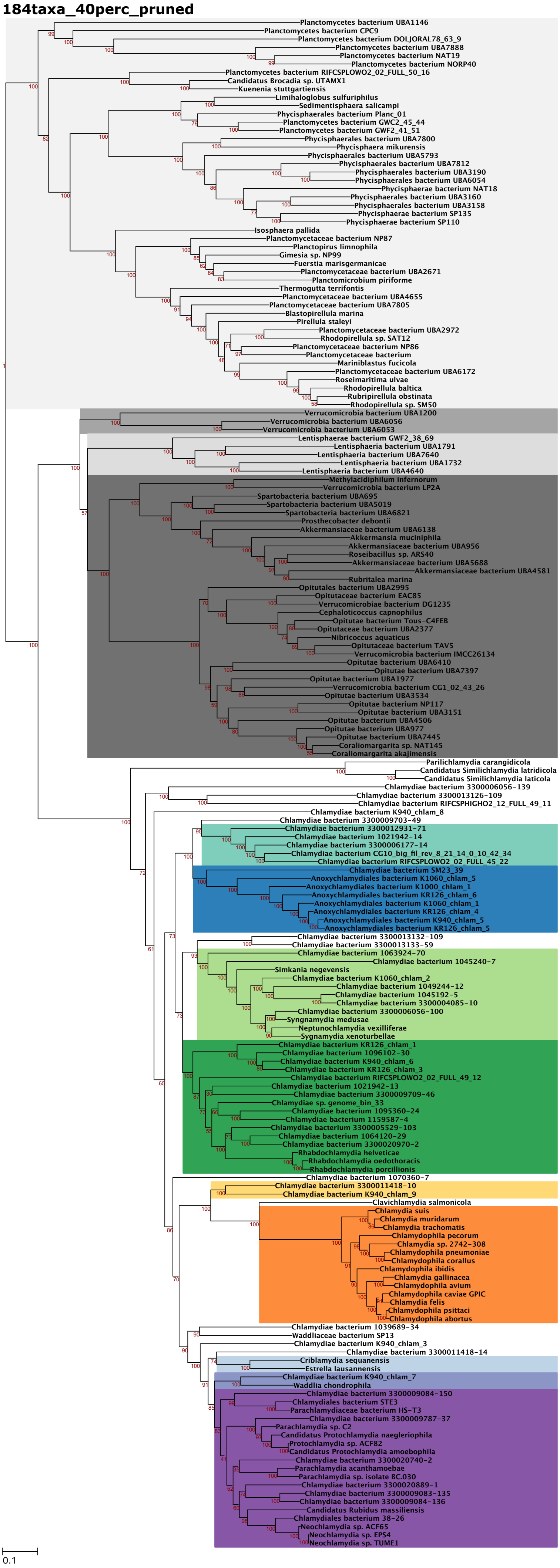

184taxa_50perc_pruned

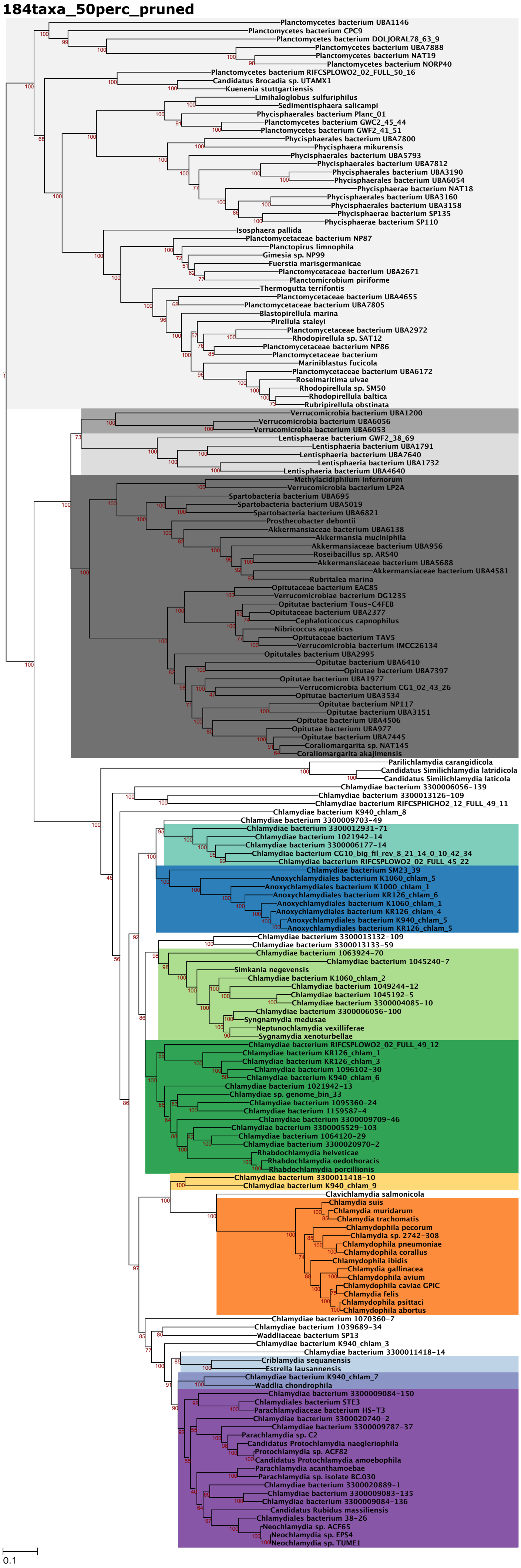

184taxa_51perc_pruned_bias_removed

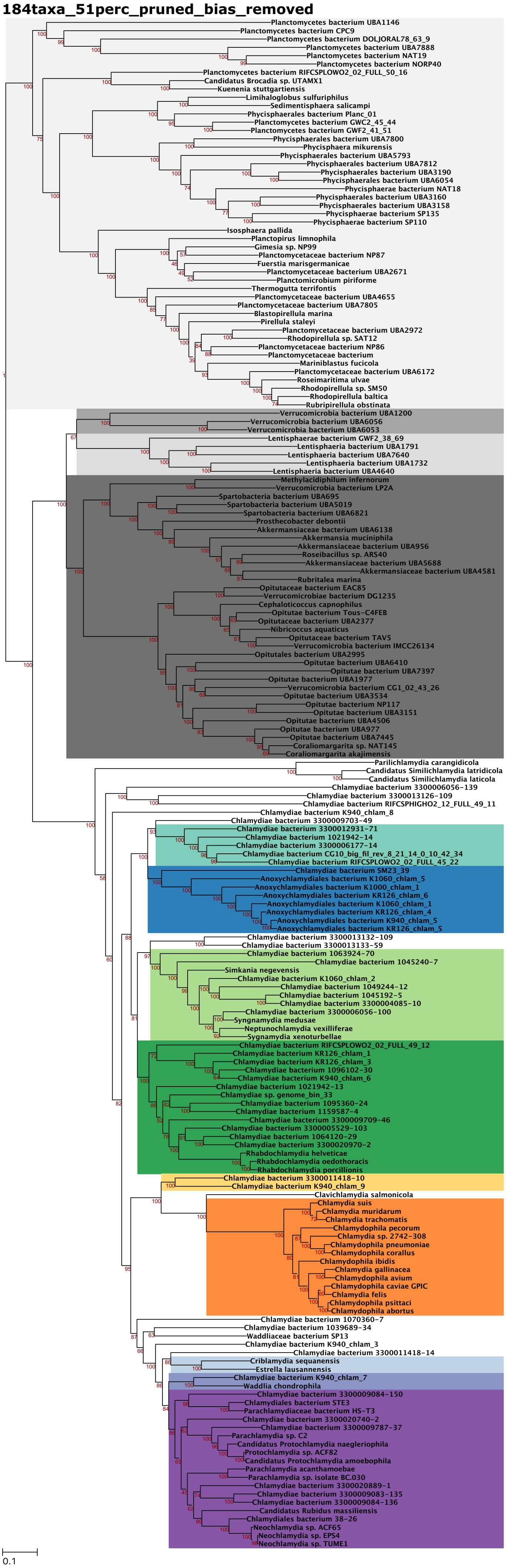

184taxa_51perc_pruned_bias_removed

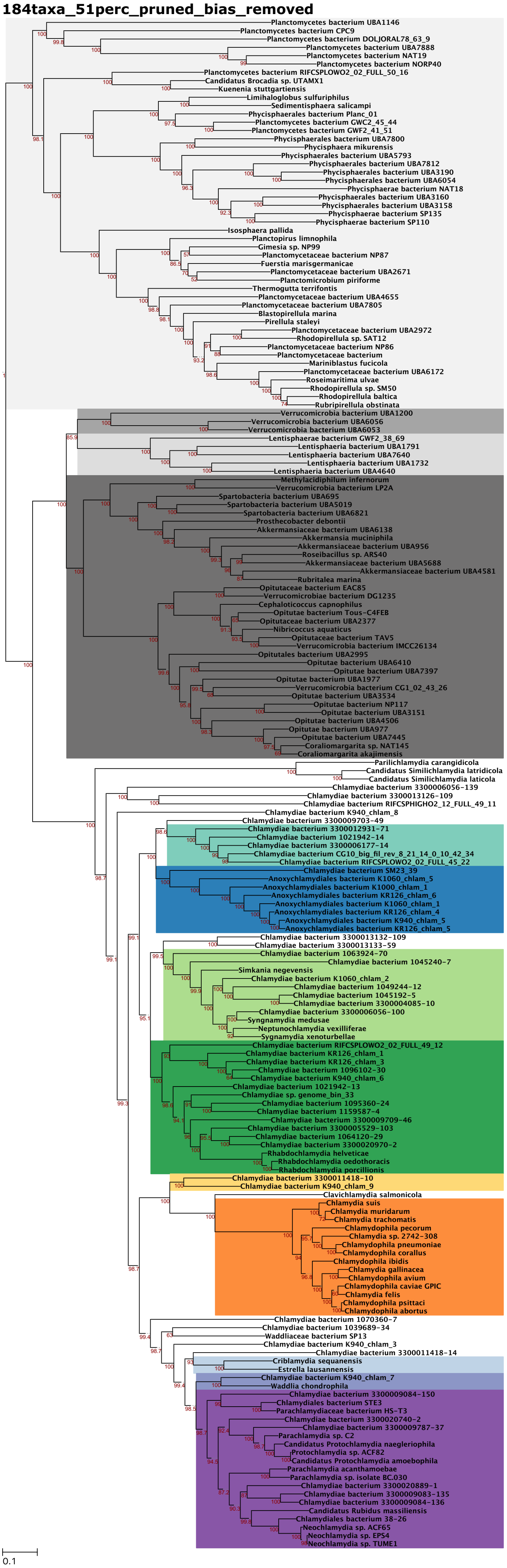

chain1

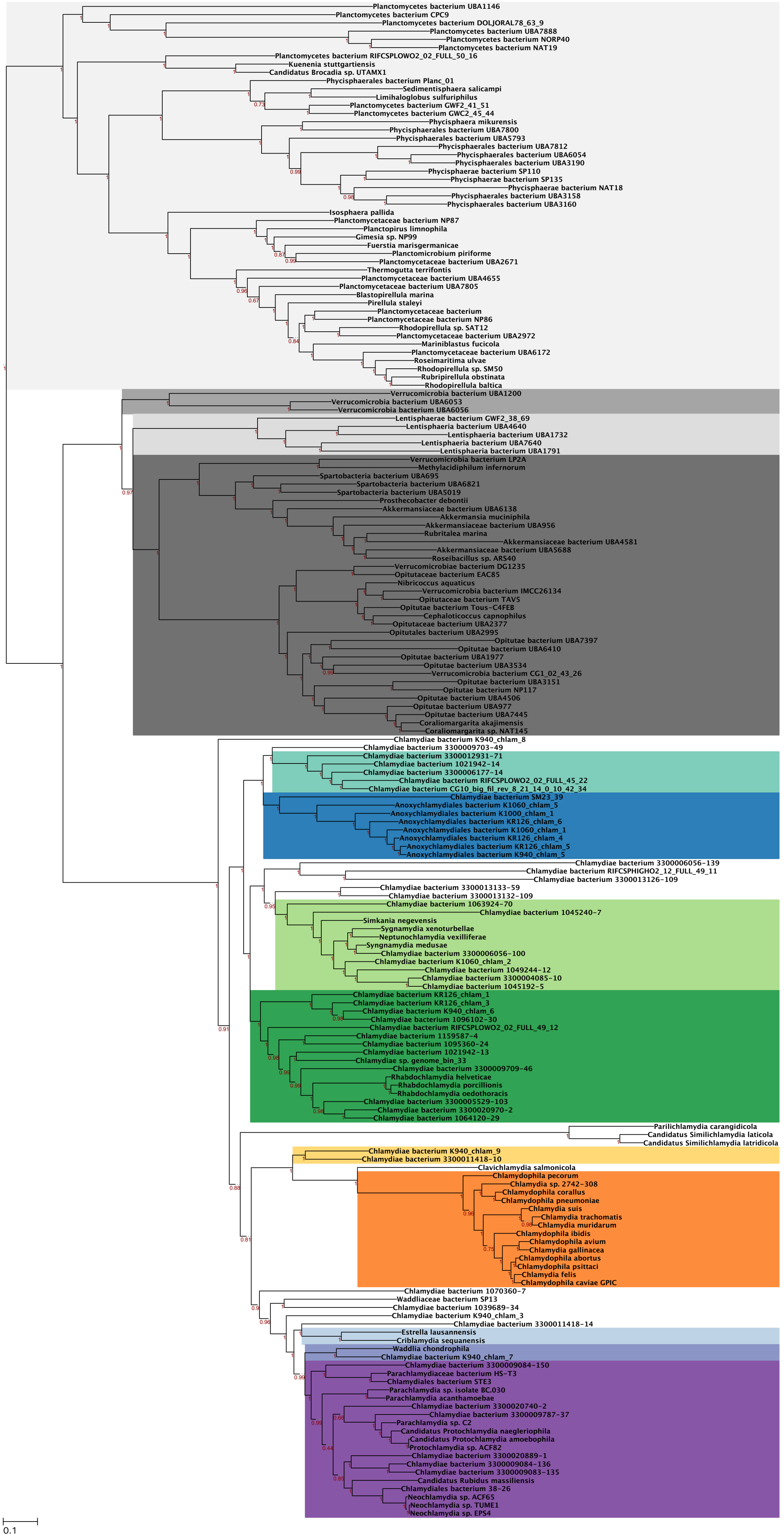

Planctomycetes bacterium UBA1146
Planctomycetes bacterium CPC9
Planctomycetes bacterium DOLJORAL78_63_9
Planctomycetes bacterium UBA7888
Planctomycetes bacterium NORP40
Planctomycetes bacterium NAT19
Planctomycetes bacterium RIFCSPLOWO2_02_FULL_50_16
Kuenenia stuttgartiensis
Candidatus Brocadia sp. UTAMX1
Phycisphaerales bacterium Planc_01
Sedimentisphaera salicampi
Limihaloglobus sulfuriphilus
Planctomycetes bacterium GWF2_41_51
Planctomycetes bacterium GWC2_45_44
Phycisphaera mikurensis
Phycisphaerales bacterium UBA7800
Phycisphaerales bacterium UBA5793
Phycisphaerales bacterium UBA7812
Phycisphaerales bacterium UBA6054
Phycisphaerales bacterium UBA3190
Phycisphaerae bacterium SP110
Phycisphaerae bacterium SP135
Phycisphaerae bacterium NAT18
Phycisphaerales bacterium UBA3158
Phycisphaerales bacterium UBA3160
Isosphaera pallida
Planctomycetaceae bacterium NP87
Planctopirus limnophila
Gimesia sp. NP99
Fuerstia marisgermanicae
Planctomicrobium piriforme
Planctomycetaceae bacterium UBA2671
Thermogutta terrifontis
Planctomycetaceae bacterium UBA4655
Planctomycetaceae bacterium UBA7805
Blastopirellula marina
Pirellula staleyi
Planctomycetaceae bacterium
Planctomycetaceae bacterium NP86
Rhodopirellula sp. SAT12
Planctomycetaceae bacterium UBA2972
Mariniblastus fucicola
Planctomycetaceae bacterium UBA6172
Roseimaritima ulvae
Rhodopirellula sp. SM50
Rubripirellula obstinata
Rhodopirellula baltica
Verrucomicrobia bacterium UBA1200
Verrucomicrobia bacterium UBA6053
Verrucomicrobia bacterium UBA6056
Lentisphaerae bacterium GWF2_38_69
Lentisphaeria bacterium UBA4640
Lentisphaeria bacterium UBA1732
Lentisphaeria bacterium UBA7640
Lentisphaeria bacterium UBA1791
Verrucomicrobia bacterium LP2A
Methylacidiphilum infernorum
Spartobacteria bacterium UBA695
Spartobacteria bacterium UBA6821
Spartobacteria bacterium UBA5019
Prosthecobacter debontii
Akkermansiaceae bacterium UBA6138
Akkermansia muciniphila
Akkermansiaceae bacterium UBA956
Rubritalea marina
Akkermansiaceae bacterium UBA4581
Akkermansiaceae bacterium UBA5688
Roseibacillus sp. ARS40
Verrucomicrobiae bacterium DG1235
Opitutaceae bacterium EAC85
Nibricoccus aquaticus
Verrucomicrobia bacterium IMCC26134
Opitutaceae bacterium TAV5
Opitutae bacterium Tous-C4FEB
Cephaloticoccus capnophilus
Opitutaceae bacterium UBA2377
Opitutales bacterium UBA2995
Opitutae bacterium UBA7397
Opitutae bacterium UBA6410
Opitutae bacterium UBA1977
Opitutae bacterium UBA3534
Verrucomicrobia bacterium CG1_02_43_26
Opitutae bacterium UBA3151
Opitutae bacterium NP117
Opitutae bacterium UBA4506
Opitutae bacterium UBA977
Opitutae bacterium UBA7445
Coraliomargarita akajimensis
Coraliomargarita sp. NAT145
Chlamydiae bacterium K940_chlam_8
Chlamydiae bacterium 3300009703-49
Chlamydiae bacterium 3300012931-71
Chlamydiae bacterium 1021942-14
Chlamydiae bacterium 3300006177-14
Chlamydiae bacterium RIFCSPLOWO2_02_FULL_45_22
Chlamydiae bacterium CG10_big_fil_rev_8_21_14_0_10_42_34
Chlamydiae bacterium SM23_39
Anoxychlamydiales bacterium K1060_chlam_5
Anoxychlamydiales bacterium K1000_chlam_1
Anoxychlamydiales bacterium KR126_chlam_6
Anoxychlamydiales bacterium K1060_chlam_1
Anoxychlamydiales bacterium KR126_chlam_4
Anoxychlamydiales bacterium KR126_chlam_5
Anoxychlamydiales bacterium K940_chlam_5
Chlamydiae bacterium 3300006056-139
Chlamydiae bacterium RIFCSPHIGHO2_12_FULL_49_11
Chlamydiae bacterium 3300013126-109
Chlamydiae bacterium 3300013133-59
Chlamydiae bacterium 3300013132-109
Chlamydiae bacterium 1063924-70
Chlamydiae bacterium 1045240-7
Simkania negevensis
Sygnamydia xenoturbellae
Neptunochlamydia vexilliferae
Syngnamydia medusae
Chlamydiae bacterium 3300006056-100
Chlamydiae bacterium K1060_chlam_2
Chlamydiae bacterium 1049244-12
Chlamydiae bacterium 3300004085-10
Chlamydiae bacterium 1045192-5
Chlamydiae bacterium KR126_chlam_1
Chlamydiae bacterium KR126_chlam_3
Chlamydiae bacterium K940_chlam_6
Chlamydiae bacterium 1096102-30
Chlamydiae bacterium RIFCSPLOWO2_02_FULL_49_12
Chlamydiae bacterium 1159587-4
Chlamydiae bacterium 1095360-24
Chlamydiae bacterium 1021942-13
Chlamydiae sp. genome_bin_33
Chlamydiae bacterium 3300009709-46
Rhabdochlamydia helveticae
Rhabdochlamydia porcellionis
Rhabdochlamydia oedothoracis
Chlamydiae bacterium 3300005529-103
Chlamydiae bacterium 3300020970-2
Chlamydiae bacterium 1064120-29
Parilichlamydia carangidicola
Candidatus Similichlamydia laticola
Candidatus Similichlamydia latridicola
Chlamydiae bacterium K940_chlam_9
Chlamydiae bacterium 3300011418-10
Clavichlamydia salmonicola
Chlamydophila pecorum
Chlamydia sp. 2742-308
Chlamydophila corallus
Chlamydophila pneumoniae
Chlamydia suis
Chlamydia trachomatis
Chlamydia muridarum
Chlamydophila ibidis
Chlamydophila avium
Chlamydia gallinacea
Chlamydophila abortus
Chlamydophila psittaci
Chlamydia felis
Chlamydophila caviae GPIC
Chlamydiae bacterium 1070360-7
Waddliaceae bacterium SP13
Chlamydiae bacterium 1039689-34
Chlamydiae bacterium K940_chlam_3
Chlamydiae bacterium 3300011418-14
Estrella lausannensis
Criblamydia sequanensis
Waddlia chondrophila
Chlamydiae bacterium K940_chlam_7
Chlamydiae bacterium 3300009084-150
Parachlamydiaceae bacterium HS-T3
Chlamydiales bacterium STE3
Parachlamydia sp. isolate BC.030
Parachlamydia acanthamoebae
Chlamydiae bacterium 3300020740-2
Chlamydiae bacterium 3300009787-37
Parachlamydia sp. C2
Candidatus Protochlamydia naegleriophila
Candidatus Protochlamydia amoebophila
Protochlamydia sp. ACF82
Chlamydiae bacterium 3300020889-1
Chlamydiae bacterium 3300009084-136
Chlamydiae bacterium 3300009083-135
Candidatus Rubidus massiliensis
Chlamydiales bacterium 38-26
Neochlamydia sp. ACF65
Neochlamydia sp. TUME1
Neochlamydia sp. EPS4
0.1

chain2

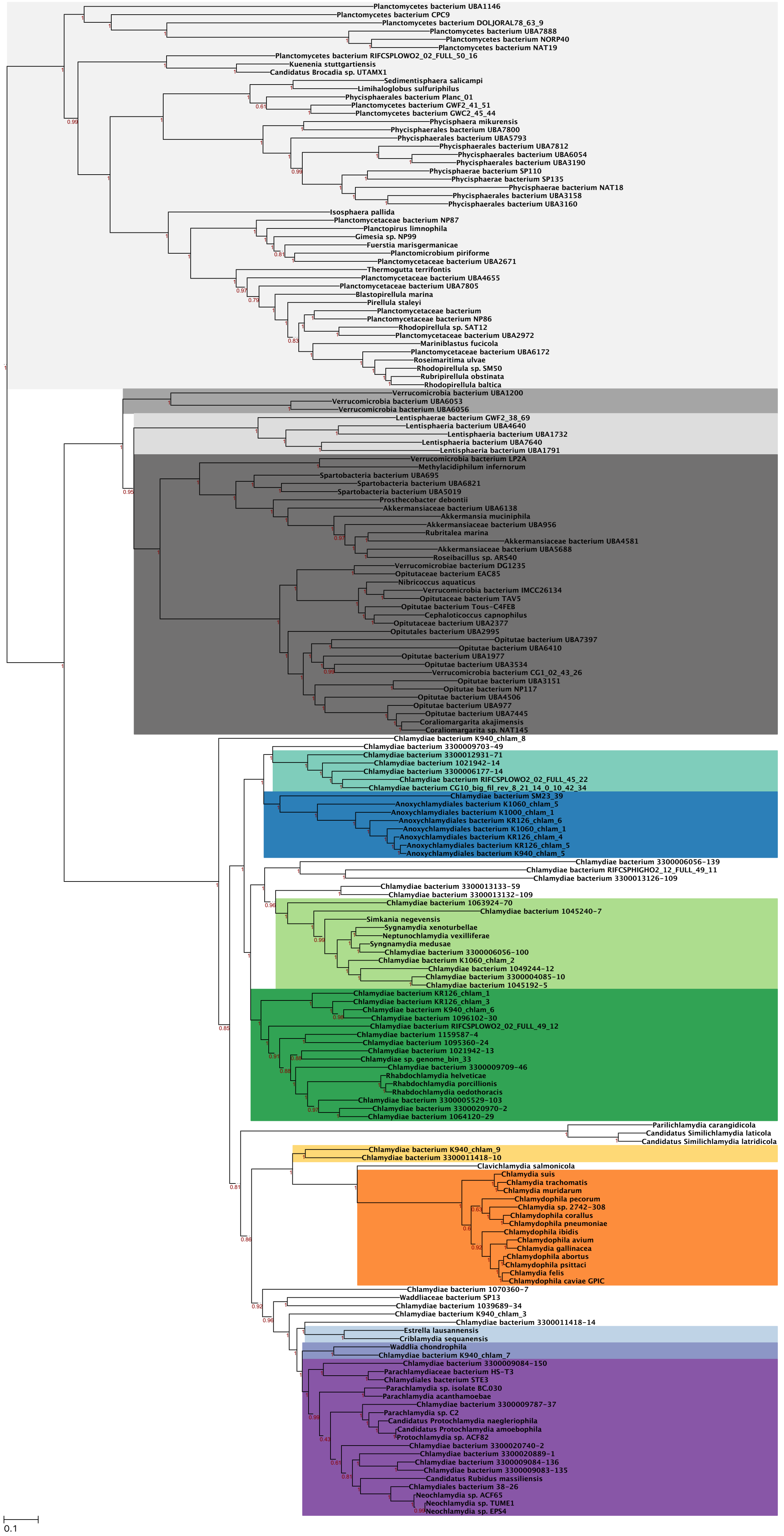
Planctomycetes bacterium UBA1146
Planctomycetes bacterium CPC9
Planctomycetes bacterium DOLJORAL78_63_9
Planctomycetes bacterium UBA7888
Planctomycetes bacterium NORP40
Planctomycetes bacterium NAT19
Planctomycetes bacterium RIFCSPLOWO2_02_FULL_50_16
Kuenenia stuttgartiensis
Candidatus Brocadia sp. UTAMX1
Sedimentisphaera salicampi
Limihaloglobus sulfuriphilus
Phycisphaerales bacterium Planc_01
Planctomycetes bacterium GWF2_41_51
Planctomycetes bacterium GWC2_45_44
Phycisphaera mikurensis
Phycisphaerales bacterium UBA7800
Phycisphaerales bacterium UBA5793
Phycisphaerales bacterium UBA7812
Phycisphaerales bacterium UBA6054
Phycisphaerales bacterium UBA3190
Phycisphaerae bacterium SP110
Phycisphaerae bacterium SP135
Phycisphaerae bacterium NAT18
Phycisphaerales bacterium UBA3158
Phycisphaerales bacterium UBA3160
Isosphaera pallida
Planctomycetaceae bacterium NP87
Planctopirus limnophila
Gimesia sp. NP99
Fuerstia marisgermanicae
Planctomicrobium piriforme
Planctomycetaceae bacterium UBA2671
Thermogutta terrifontis
Planctomycetaceae bacterium UBA4655
Planctomycetaceae bacterium UBA7805
Blastopirellula marina
Pirellula staleyi
Planctomycetaceae bacterium
Planctomycetaceae bacterium NP86
Rhodopirellula sp. SAT12
Planctomycetaceae bacterium UBA2972
Mariniblastus fucicola
Planctomycetaceae bacterium UBA6172
Roseimaritima ulvae
Rhodopirellula sp. SM50
Rubripirellula obstinata
Rhodopirellula baltica
Verrucomicrobia bacterium UBA1200
Verrucomicrobia bacterium UBA6053
Verrucomicrobia bacterium UBA6056
Lentisphaerae bacterium GWF2_38_69
Lentisphaeria bacterium UBA4640
Lentisphaeria bacterium UBA1732
Lentisphaeria bacterium UBA7640
Lentisphaeria bacterium UBA1791
Verrucomicrobia bacterium LP2A
Methylacidiphilum infernorum
Spartobacteria bacterium UBA695
Spartobacteria bacterium UBA6821
Spartobacteria bacterium UBA5019
Prosthecobacter debontii
Akkermansiaceae bacterium UBA6138
Akkermansia muciniphila
Akkermansiaceae bacterium UBA956
Rubritalea marina
Akkermansiaceae bacterium UBA4581
Akkermansiaceae bacterium UBA5688
Roseibacillus sp. ARS40
Verrucomicrobiae bacterium DG1235
Opitutaceae bacterium EAC85
Nibricoccus aquaticus
Verrucomicrobia bacterium IMCC26134
Opitutaceae bacterium TAV5
Opitutae bacterium Tous-C4FEB
Cephaloticoccus capnophilus
Opitutaceae bacterium UBA2377
Opitutales bacterium UBA2995
Opitutae bacterium UBA7397
Opitutae bacterium UBA6410
Opitutae bacterium UBA1977
Opitutae bacterium UBA3534
Verrucomicrobia bacterium CG1_02_43_26
Opitutae bacterium UBA3151
Opitutae bacterium NP117
Opitutae bacterium UBA4506
Opitutae bacterium UBA977
Opitutae bacterium UBA7445
Coraliomargarita akajimensis
Coraliomargarita sp. NAT145
Chlamydiae bacterium K940_chlam_8
Chlamydiae bacterium 3300009703-49
Chlamydiae bacterium 3300012931-71
Chlamydiae bacterium 1021942-14
Chlamydiae bacterium 3300006177-14
Chlamydiae bacterium RIFCSPLOWO2_02_FULL_45_22
Chlamydiae bacterium CG10_big_fil_rev_8_21_14_0_10_42_34
Chlamydiae bacterium SM23_39
Anoxychlamydiales bacterium K1060_chlam_5
Anoxychlamydiales bacterium K1000_chlam_1
Anoxychlamydiales bacterium KR126_chlam_6
Anoxychlamydiales bacterium K1060_chlam_1
Anoxychlamydiales bacterium KR126_chlam_4
Anoxychlamydiales bacterium KR126_chlam_5
Anoxychlamydiales bacterium K940_chlam_5
Chlamydiae bacterium 3300006056-139
Chlamydiae bacterium RIFCSPHIGHO2_12_FULL_49_11
Chlamydiae bacterium 3300013126-109
Chlamydiae bacterium 3300013133-59
Chlamydiae bacterium 3300013132-109
Chlamydiae bacterium 1063924-70
Chlamydiae bacterium 1045240-7
Simkania negevensis
Sygnamydia xenoturbellae
Neptunochlamydia vexilliferae
Syngnamydia medusae
Chlamydiae bacterium 3300006056-100
Chlamydiae bacterium K1060_chlam_2
Chlamydiae bacterium 1049244-12
Chlamydiae bacterium 3300004085-10
Chlamydiae bacterium 1045192-5
Chlamydiae bacterium KR126_chlam_1
Chlamydiae bacterium KR126_chlam_3
Chlamydiae bacterium K940_chlam_6
Chlamydiae bacterium 1096102-30
Chlamydiae bacterium RIFCSPLOWO2_02_FULL_49_12
Chlamydiae bacterium 1159587-4
Chlamydiae bacterium 1095360-24
Chlamydiae bacterium 1021942-13
Chlamydiae sp. genome_bin_33
Chlamydiae bacterium 3300009709-46
Rhabdochlamydia helveticae
Rhabdochlamydia porcellionis
Rhabdochlamydia oedothoracis
Chlamydiae bacterium 3300005529-103
Chlamydiae bacterium 3300020970-2
Chlamydiae bacterium 1064120-29
Parilichlamydia carangidicola
Candidatus Similichlamydia laticola
Candidatus Similichlamydia latridicola
Chlamydiae bacterium K940_chlam_9
Chlamydiae bacterium 3300011418-10
Clavichlamydia salmonicola
Chlamydia suis
Chlamydia trachomatis
Chlamydia muridarum
Chlamydophila pecorum
Chlamydia sp. 2742-308
Chlamydophila corallus
Chlamydophila pneumoniae
Chlamydophila ibidis
Chlamydophila avium
Chlamydia gallinacea
Chlamydophila abortus
Chlamydophila psittaci
Chlamydia felis
Chlamydophila caviae GPIC
Chlamydiae bacterium 1070360-7
Waddliaceae bacterium SP13
Chlamydiae bacterium 1039689-34
Chlamydiae bacterium K940_chlam_3
Chlamydiae bacterium 3300011418-14
Estrella lausannensis
Criblamydia sequanensis
Waddlia chondrophila
Chlamydiae bacterium K940_chlam_7
Chlamydiae bacterium 3300009084-150
Parachlamydiaceae bacterium HS-T3
Chlamydiales bacterium STE3
Parachlamydia sp. isolate BC.030
Parachlamydia acanthamoebae
Chlamydiae bacterium 3300009787-37
Parachlamydia sp. C2
Candidatus Protochlamydia naegleriophila
Candidatus Protochlamydia amoebophila
Protochlamydia sp. ACF82
Chlamydiae bacterium 3300020740-2
Chlamydiae bacterium 3300020889-1
Chlamydiae bacterium 3300009084-136
Chlamydiae bacterium 3300009083-135
Candidatus Rubidus massiliensis
Chlamydiales bacterium 38-26
Neochlamydia sp. ACF65
Neochlamydia sp. TUME1
Neochlamydia sp. EPS4
0.1

chain3

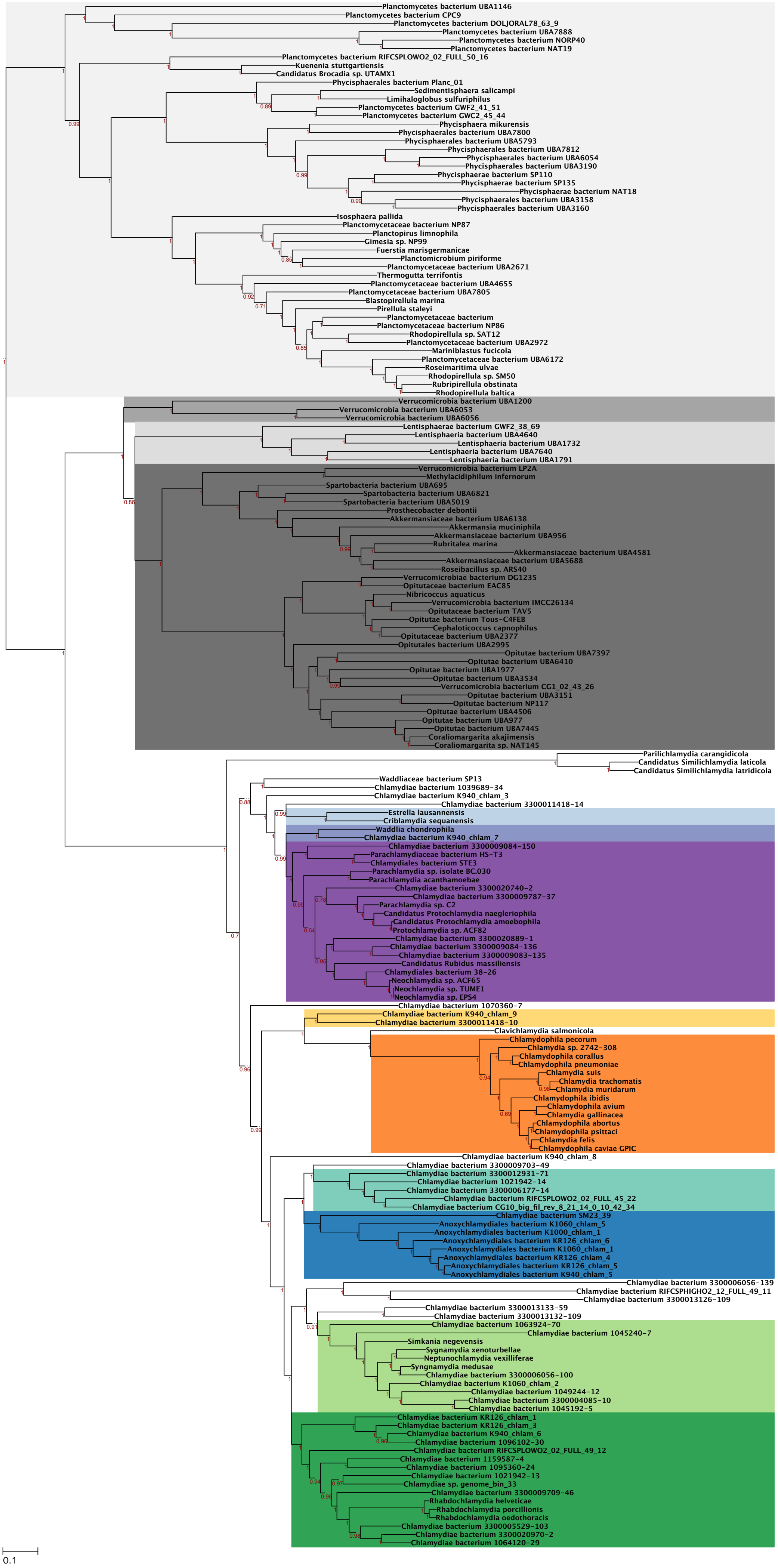
Planctomycetes bacterium UBA1146
Planctomycetes bacterium CPC9
Planctomycetes bacterium DOLJORAL78_63_9
Planctomycetes bacterium UBA7888
Planctomycetes bacterium NORP40
Planctomycetes bacterium NAT19
Planctomycetes bacterium RIFCSPLOWO2_02_FULL_50_16
Kuenenia stuttgartiensis
Candidatus Brocadia sp. UTAMX1
Phycisphaerales bacterium Planc_01
Sedimentisphaera salicampi
Limihaloglobus sulfuriphilus
Planctomycetes bacterium GWF2_41_51
Planctomycetes bacterium GWC2_45_44
Phycisphaera mikurensis
Phycisphaerales bacterium UBA7800
Phycisphaerales bacterium UBA5793
Phycisphaerales bacterium UBA7812
Phycisphaerales bacterium UBA6054
Phycisphaerales bacterium UBA3190
Phycisphaerae bacterium SP110
Phycisphaerae bacterium SP135
Phycisphaerae bacterium NAT18
Phycisphaerales bacterium UBA3158
Phycisphaerales bacterium UBA3160
Isosphaera pallida
Planctomycetaceae bacterium NP87
Planctopirus limnophila
Gimesia sp. NP99
Fuerstia marisgermanicae
Planctomicrobium piriforme
Planctomycetaceae bacterium UBA2671
Thermogutta terrifontis
Planctomycetaceae bacterium UBA4655
Planctomycetaceae bacterium UBA7805
Blastopirellula marina
Pirellula staleyi
Planctomycetaceae bacterium
Planctomycetaceae bacterium NP86
Rhodopirellula sp. SAT12
Planctomycetaceae bacterium UBA2972
Mariniblastus fucicola
Planctomycetaceae bacterium UBA6172
Roseimaritima ulvae
Rhodopirellula sp. SM50
Rubripirellula obstinata
Rhodopirellula baltica
Verrucomicrobia bacterium UBA1200
Verrucomicrobia bacterium UBA6053
Verrucomicrobia bacterium UBA6056
Lentisphaerae bacterium GWF2_38_69
Lentisphaeria bacterium UBA4640
Lentisphaeria bacterium UBA1732
Lentisphaeria bacterium UBA7640
Lentisphaeria bacterium UBA1791
Verrucomicrobia bacterium LP2A
Methylacidiphilum infernorum
Spartobacteria bacterium UBA695
Spartobacteria bacterium UBA6821
Spartobacteria bacterium UBA5019
Prosthecobacter debontii
Akkermansiaceae bacterium UBA6138
Akkermansia muciniphila
Akkermansiaceae bacterium UBA956
Rubritalea marina
Akkermansiaceae bacterium UBA4581
Akkermansiaceae bacterium UBA5688
Roseibacillus sp. ARS40
Verrucomicrobiae bacterium DG1235
Opitutaceae bacterium EAC85
Nibricoccus aquaticus
Verrucomicrobia bacterium IMCC26134
Opitutaceae bacterium TAV5
Opitutae bacterium Tous-C4FEB
Cephaloticoccus capnophilus
Opitutaceae bacterium UBA2377
Opitutales bacterium UBA2995
Opitutae bacterium UBA7397
Opitutae bacterium UBA6410
Opitutae bacterium UBA1977
Opitutae bacterium UBA3534
Verrucomicrobia bacterium CG1_02_43_26
Opitutae bacterium UBA3151
Opitutae bacterium NP117
Opitutae bacterium UBA4506
Opitutae bacterium UBA977
Opitutae bacterium UBA7445
Coraliomargarita akajimensis
Coraliomargarita sp. NAT145
Parilichlamydia carangidicola
Candidatus Similichlamydia laticola
Candidatus Similichlamydia latridicola
Waddliaceae bacterium SP13
Chlamydiae bacterium 1039689-34
Chlamydiae bacterium K940_chlam_3
Chlamydiae bacterium 3300011418-14
Estrella lausannensis
Criblamydia sequanensis
Waddlia chondrophila
Chlamydiae bacterium K940_chlam_7
Chlamydiae bacterium 3300009084-150
Parachlamydiaceae bacterium HS-T3
Chlamydiales bacterium STE3
Parachlamydia sp. isolate BC.030
Parachlamydia acanthamoebae
Chlamydiae bacterium 3300020740-2
Chlamydiae bacterium 3300009787-37
Parachlamydia sp. C2
Candidatus Protochlamydia naegleriophila
Candidatus Protochlamydia amoebophila
Protochlamydia sp. ACF82
Chlamydiae bacterium 3300020889-1
Chlamydiae bacterium 3300009084-136
Chlamydiae bacterium 3300009083-135
Candidatus Rubidus massiliensis
Chlamydiales bacterium 38-26
Neochlamydia sp. ACF65
Neochlamydia sp. TUME1
Neochlamydia sp. EPS4
Chlamydiae bacterium 1070360-7
Chlamydiae bacterium K940_chlam_9
Chlamydiae bacterium 3300011418-10
Clavichlamydia salmonicola
Chlamydophila pecorum
Chlamydia sp. 2742-308
Chlamydophila corallus
Chlamydophila pneumoniae
Chlamydia suis
Chlamydia trachomatis
Chlamydia muridarum
Chlamydophila ibidis
Chlamydophila avium
Chlamydia gallinacea
Chlamydophila abortus
Chlamydophila psittaci
Chlamydia felis
Chlamydophila caviae GPIC
Chlamydiae bacterium K940_chlam_8
Chlamydiae bacterium 3300009703-49
Chlamydiae bacterium 3300012931-71
Chlamydiae bacterium 1021942-14
Chlamydiae bacterium 3300006177-14
Chlamydiae bacterium RIFCSPLOWO2_02_FULL_45_22
Chlamydiae bacterium CG10_big_fil_rev_8_21_14_0_10_42_34
Chlamydiae bacterium SM23_39
Anoxychlamydiales bacterium K1060_chlam_5
Anoxychlamydiales bacterium K1000_chlam_1
Anoxychlamydiales bacterium KR126_chlam_6
Anoxychlamydiales bacterium K1060_chlam_1
Anoxychlamydiales bacterium KR126_chlam_4
Anoxychlamydiales bacterium KR126_chlam_5
Anoxychlamydiales bacterium K940_chlam_5
Chlamydiae bacterium 3300006056-139
Chlamydiae bacterium RIFCSPHIGHO2_12_FULL_49_11
Chlamydiae bacterium 3300013126-109
Chlamydiae bacterium 3300013133-59
Chlamydiae bacterium 3300013132-109
Chlamydiae bacterium 1063924-70
Chlamydiae bacterium 1045240-7
Simkania negevensis
Sygnamydia xenoturbellae
Neptunochlamydia vexilliferae
Syngnamydia medusae
Chlamydiae bacterium 3300006056-100
Chlamydiae bacterium K1060_chlam_2
Chlamydiae bacterium 1049244-12
Chlamydiae bacterium 3300004085-10
Chlamydiae bacterium 1045192-5
Chlamydiae bacterium KR126_chlam_1
Chlamydiae bacterium KR126_chlam_3
Chlamydiae bacterium K940_chlam_6
Chlamydiae bacterium 1096102-30
Chlamydiae bacterium RIFCSPLOWO2_02_FULL_49_12
Chlamydiae bacterium 1159587-4
Chlamydiae bacterium 1095360-24
Chlamydiae bacterium 1021942-13
Chlamydiae sp. genome_bin_33
Chlamydiae bacterium 3300009709-46
Rhabdochlamydia helveticae
Rhabdochlamydia porcellionis
Rhabdochlamydia oedothoracis
Chlamydiae bacterium 3300005529-103
Chlamydiae bacterium 3300020970-2
Chlamydiae bacterium 1064120-29
0.1

chain4

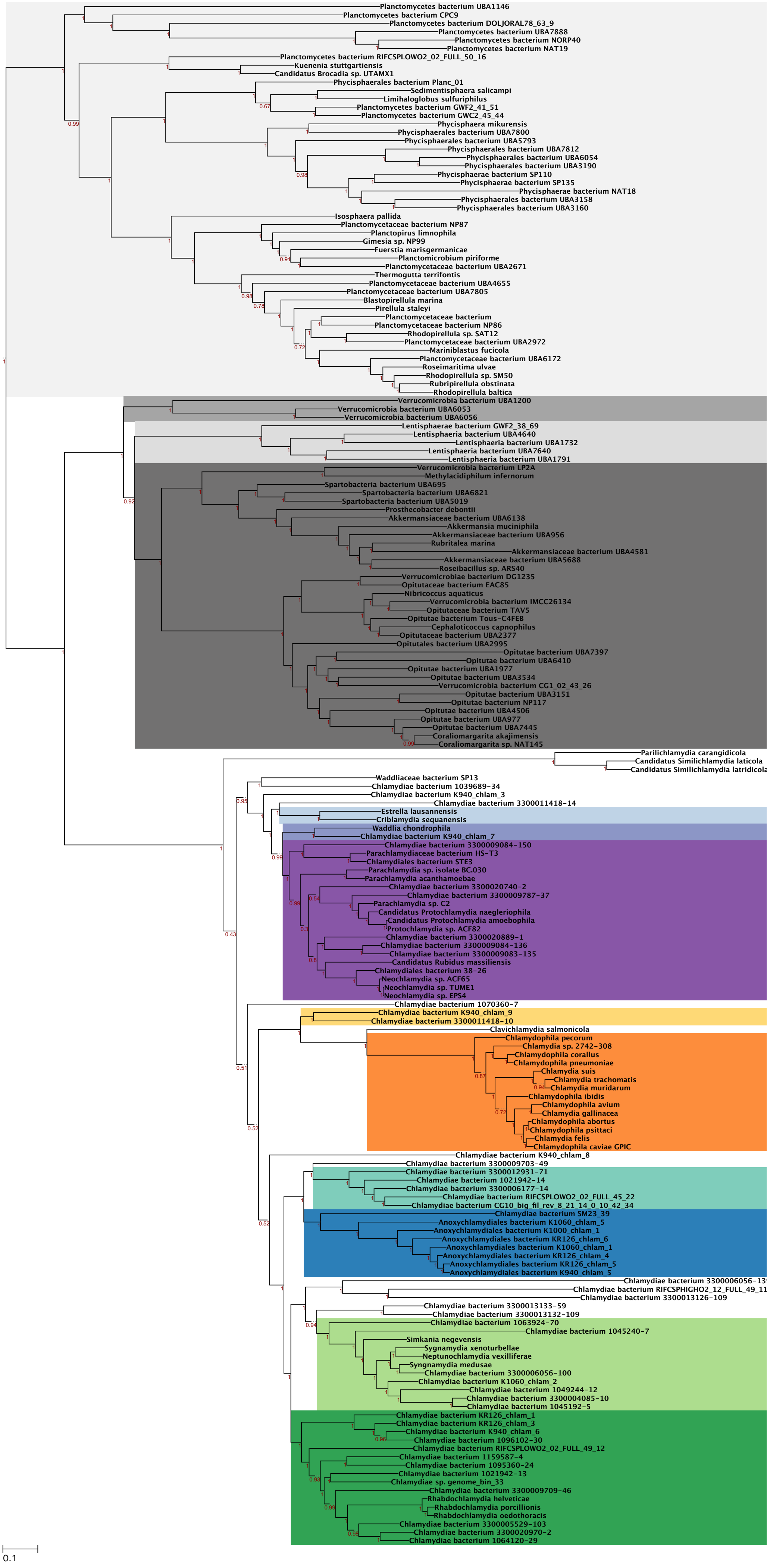
Planctomycetes bacterium UBA1146
Planctomycetes bacterium CPC9
Planctomycetes bacterium DOLJORAL78_63_9
Planctomycetes bacterium UBA7888
Planctomycetes bacterium NORP40
Planctomycetes bacterium NAT19
Planctomycetes bacterium RIFCSPLOWO2_02_FULL_50_16
Kuenenia stuttgartiensis
Candidatus Brocadia sp. UTAMX1
Phycisphaerales bacterium Planc_01
Sedimentisphaera salicampi
Limihaloglobus sulfuriphilus
Planctomycetes bacterium GWF2_41_51
Planctomycetes bacterium GWC2_45_44
Phycisphaera mikurensis
Phycisphaerales bacterium UBA7800
Phycisphaerales bacterium UBA5793
Phycisphaerales bacterium UBA7812
Phycisphaerales bacterium UBA6054
Phycisphaerales bacterium UBA3190
Phycisphaerae bacterium SP110
Phycisphaerae bacterium SP135
Phycisphaerae bacterium NAT18
Phycisphaerales bacterium UBA3158
Phycisphaerales bacterium UBA3160
Isosphaera pallida
Planctomycetaceae bacterium NP87
Planctopirus limnophila
Gimesia sp. NP99
Fuerstia marisgermanicae
Planctomicrobium piriforme
Planctomycetaceae bacterium UBA2671
Thermogutta terrifontis
Planctomycetaceae bacterium UBA4655
Planctomycetaceae bacterium UBA7805
Blastopirellula marina
Pirellula staleyi
Planctomycetaceae bacterium
Planctomycetaceae bacterium NP86
Rhodopirellula sp. SAT12
Planctomycetaceae bacterium UBA2972
Mariniblastus fucicola
Planctomycetaceae bacterium UBA6172
Roseimaritima ulvae
Rhodopirellula sp. SM50
Rubripirellula obstinata
Rhodopirellula baltica
Verrucomicrobia bacterium UBA1200
Verrucomicrobia bacterium UBA6053
Verrucomicrobia bacterium UBA6056
Lentisphaerae bacterium GWF2_38_69
Lentisphaeria bacterium UBA4640
Lentisphaeria bacterium UBA1732
Lentisphaeria bacterium UBA7640
Lentisphaeria bacterium UBA1791
Verrucomicrobia bacterium LP2A
Methylacidiphilum infernorum
Spartobacteria bacterium UBA695
Spartobacteria bacterium UBA6821
Spartobacteria bacterium UBA5019
Prosthecobacter debontii
Akkermansiaceae bacterium UBA6138
Akkermansia muciniphila
Akkermansiaceae bacterium UBA956
Rubritalea marina
Akkermansiaceae bacterium UBA4581
Akkermansiaceae bacterium UBA5688
Roseibacillus sp. ARS40
Verrucomicrobiae bacterium DG1235
Opitutaceae bacterium EAC85
Nibricoccus aquaticus
Verrucomicrobia bacterium IMCC26134
Opitutaceae bacterium TAV5
Opitutae bacterium Tous-C4FEB
Cephaloticoccus capnophilus
Opitutaceae bacterium UBA2377
Opitutales bacterium UBA2995
Opitutae bacterium UBA7397
Opitutae bacterium UBA6410
Opitutae bacterium UBA1977
Opitutae bacterium UBA3534
Verrucomicrobia bacterium CG1_02_43_26
Opitutae bacterium UBA3151
Opitutae bacterium NP117
Opitutae bacterium UBA4506
Opitutae bacterium UBA977
Opitutae bacterium UBA7445
Coraliomargarita akajimensis
Coraliomargarita sp. NAT145
Parilichlamydia carangidicola
Candidatus Similichlamydia laticola
Candidatus Similichlamydia latridicola
Waddliaceae bacterium SP13
Chlamydiae bacterium 1039689-34
Chlamydiae bacterium K940_chlam_3
Chlamydiae bacterium 3300011418-14
Estrella lausannensis
Criblamydia sequanensis
Waddlia chondrophila
Chlamydiae bacterium K940_chlam_7
Chlamydiae bacterium 3300009084-150
Parachlamydiaceae bacterium HS-T3
Chlamydiales bacterium STE3
Parachlamydia sp. isolate BC.030
Parachlamydia acanthamoebae
Chlamydiae bacterium 3300020740-2
Chlamydiae bacterium 3300009787-37
Parachlamydia sp. C2
Candidatus Protochlamydia naegleriophila
Candidatus Protochlamydia amoebophila
Protochlamydia sp. ACF82
Chlamydiae bacterium 3300020889-1
Chlamydiae bacterium 3300009084-136
Chlamydiae bacterium 3300009083-135
Candidatus Rubidus massiliensis
Chlamydiales bacterium 38-26
Neochlamydia sp. ACF65
Neochlamydia sp. TUME1
Neochlamydia sp. EPS4
Chlamydiae bacterium 1070360-7
Chlamydiae bacterium K940_chlam_9
Chlamydiae bacterium 3300011418-10
Clavichlamydia salmonicola
Chlamydophila pecorum
Chlamydia sp. 2742-308
Chlamydophila corallus
Chlamydophila pneumoniae
Chlamydia suis
Chlamydia trachomatis
Chlamydia muridarum
Chlamydophila ibidis
Chlamydophila avium
Chlamydia gallinacea
Chlamydophila abortus
Chlamydophila psittaci
Chlamydia felis
Chlamydophila caviae GPIC
Chlamydiae bacterium K940_chlam_8
Chlamydiae bacterium 3300009703-49
Chlamydiae bacterium 3300012931-71
Chlamydiae bacterium 1021942-14
Chlamydiae bacterium 3300006177-14
Chlamydiae bacterium RIFCSPLOWO2_02_FULL_45_22
Chlamydiae bacterium CG10_big_fil_rev_8_21_14_0_10_42_34
Chlamydiae bacterium SM23_39
Anoxychlamydiales bacterium K1060_chlam_5
Anoxychlamydiales bacterium K1000_chlam_1
Anoxychlamydiales bacterium KR126_chlam_6
Anoxychlamydiales bacterium K1060_chlam_1
Anoxychlamydiales bacterium KR126_chlam_4
Anoxychlamydiales bacterium KR126_chlam_5
Anoxychlamydiales bacterium K940_chlam_5
Chlamydiae bacterium 3300006056-13
Chlamydiae bacterium RIFCSPHIGHO2_12_FULL_49_11
Chlamydiae bacterium 3300013126-109
Chlamydiae bacterium 3300013133-59
Chlamydiae bacterium 3300013132-109
Chlamydiae bacterium 1063924-70
Chlamydiae bacterium 1045240-7
Simkania negevensis
Syngnamydia xenoturbellae
Neptunochlamydia vexilliferae
Syngnamydia medusae
Chlamydiae bacterium 3300006056-100
Chlamydiae bacterium K1060_chlam_2
Chlamydiae bacterium 1049244-12
Chlamydiae bacterium 3300004085-10
Chlamydiae bacterium 1045192-5
Chlamydiae bacterium KR126_chlam_1
Chlamydiae bacterium KR126_chlam_3
Chlamydiae bacterium K940_chlam_6
Chlamydiae bacterium 1096102-30
Chlamydiae bacterium RIFCSPLOWO2_02_FULL_49_12
Chlamydiae bacterium 1159587-4
Chlamydiae bacterium 1095360-24
Chlamydiae bacterium 1021942-13
Chlamydiae sp. genome_bin_33
Chlamydiae bacterium 3300009709-46
Rhabdochlamydia helveticae
Rhabdochlamydia porcellionis
Rhabdochlamydia oedothoracis
Chlamydiae bacterium 3300005529-103
Chlamydiae bacterium 3300020970-2
Chlamydiae bacterium 1064120-29
0.1

convergence_chains_1234

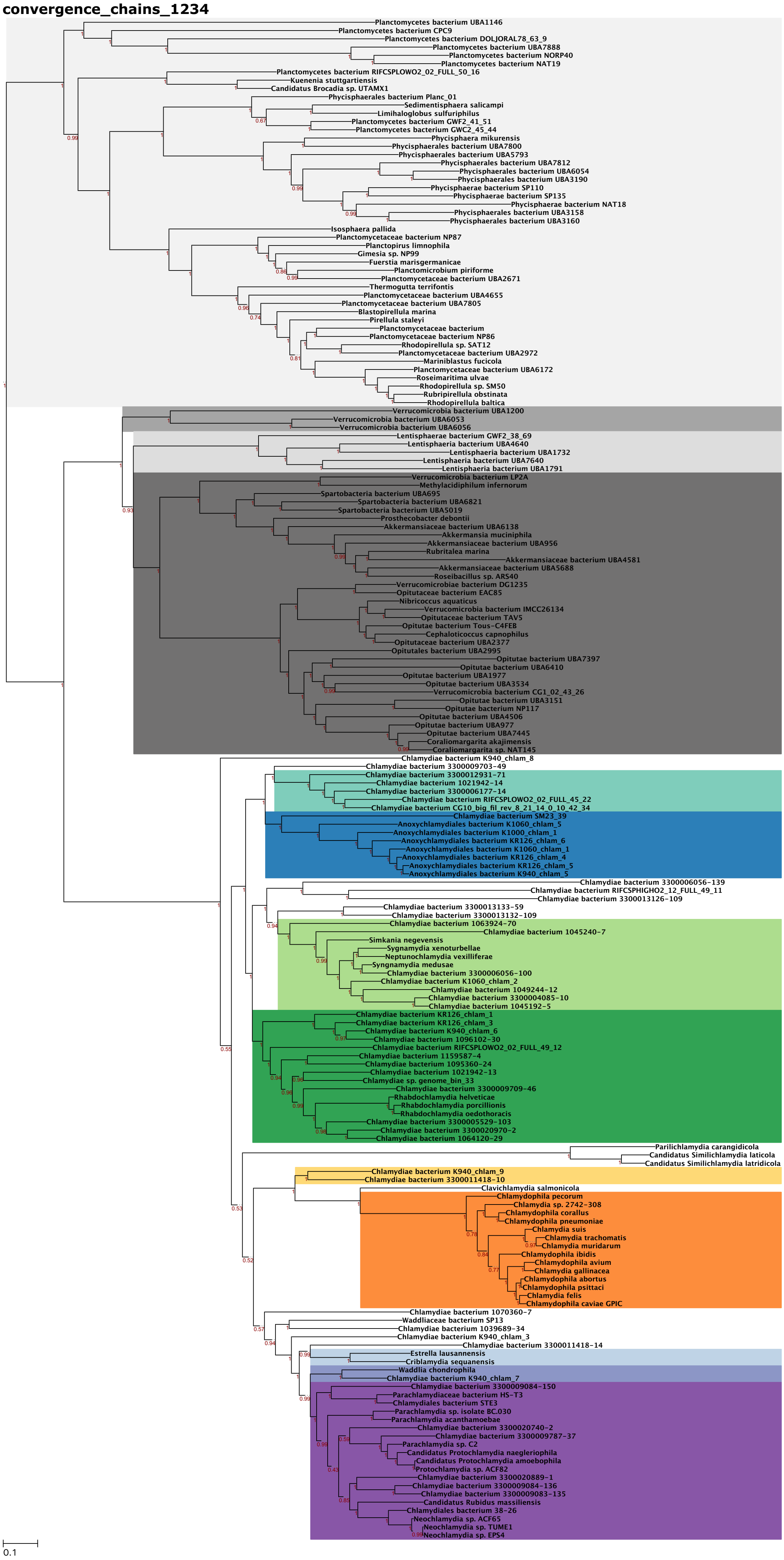

COG0056
atpA
K02111
F-type H+/Na+-transporting ATPase subunit alpha

PF00006: ATP synthase alpha/beta family, nucleotide-binding domain
PF00306: ATP synthase alpha/beta chain, C terminal domain
PF02874: ATP synthase alpha/beta family, beta-barrel domain
PF00231: ATP synthase
PF00213: ATP synthase delta (OSCP) subunit

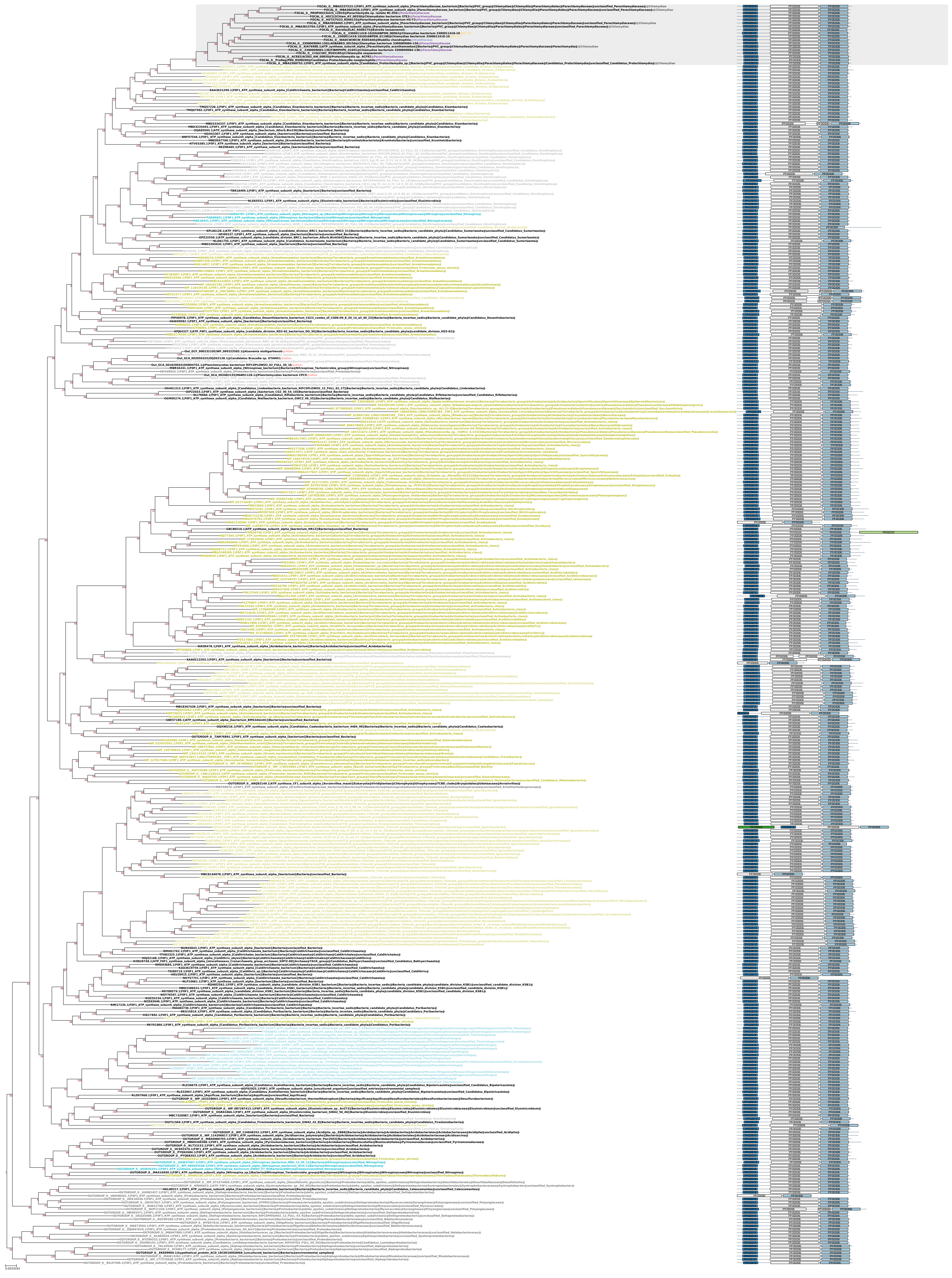

COG0356
atpB
K02108
F-type H+-transporting ATPase subunit a

PF00119: ATP synthase A chain

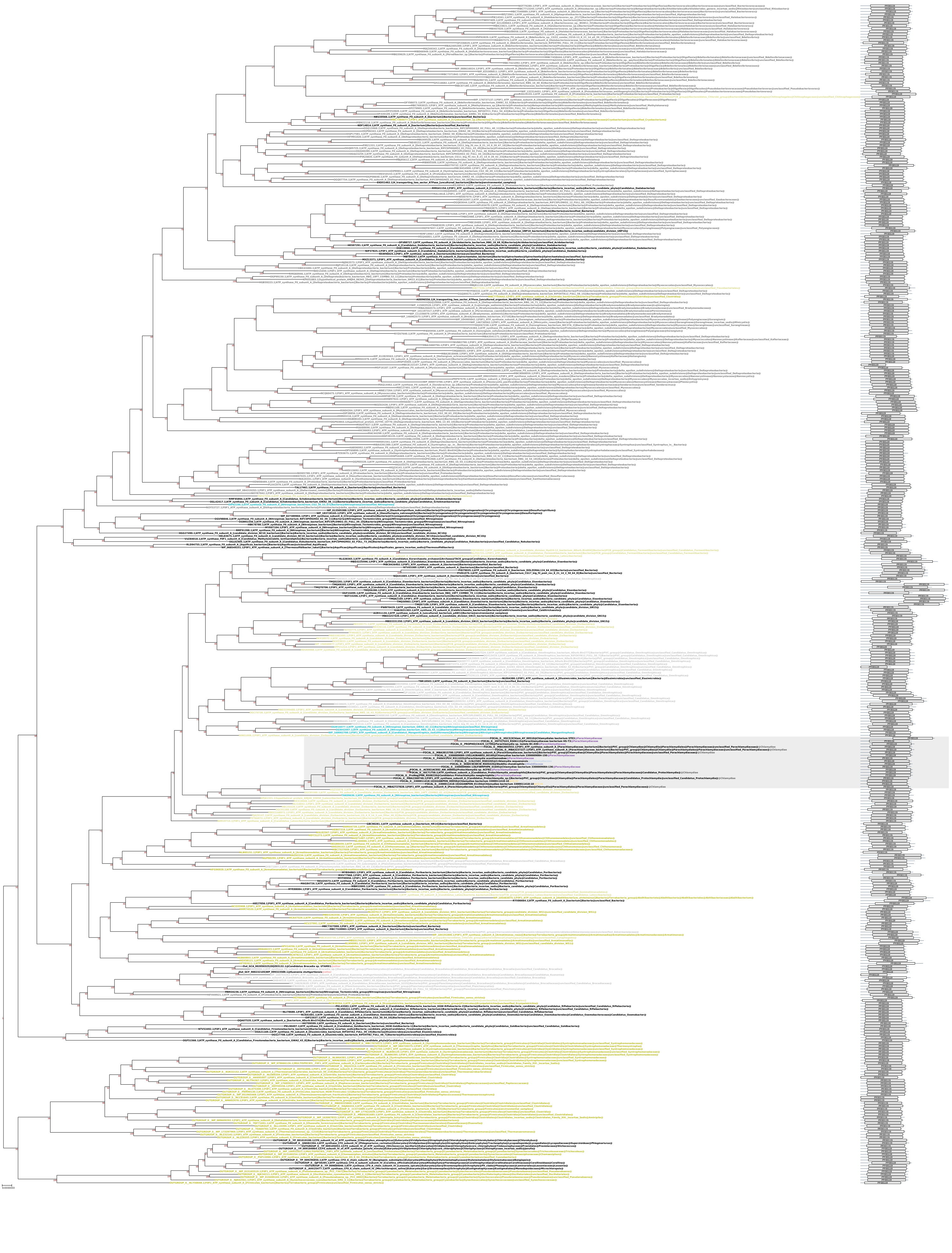

11YZG
atpC
K02114
F-type H+-transporting ATPase subunit epsilon

PF00401: ATP synthase, Delta/Epsilon chain, long alpha-helix domain
PF02823: ATP synthase, Delta/Epsilon chain, beta-sandwich domain

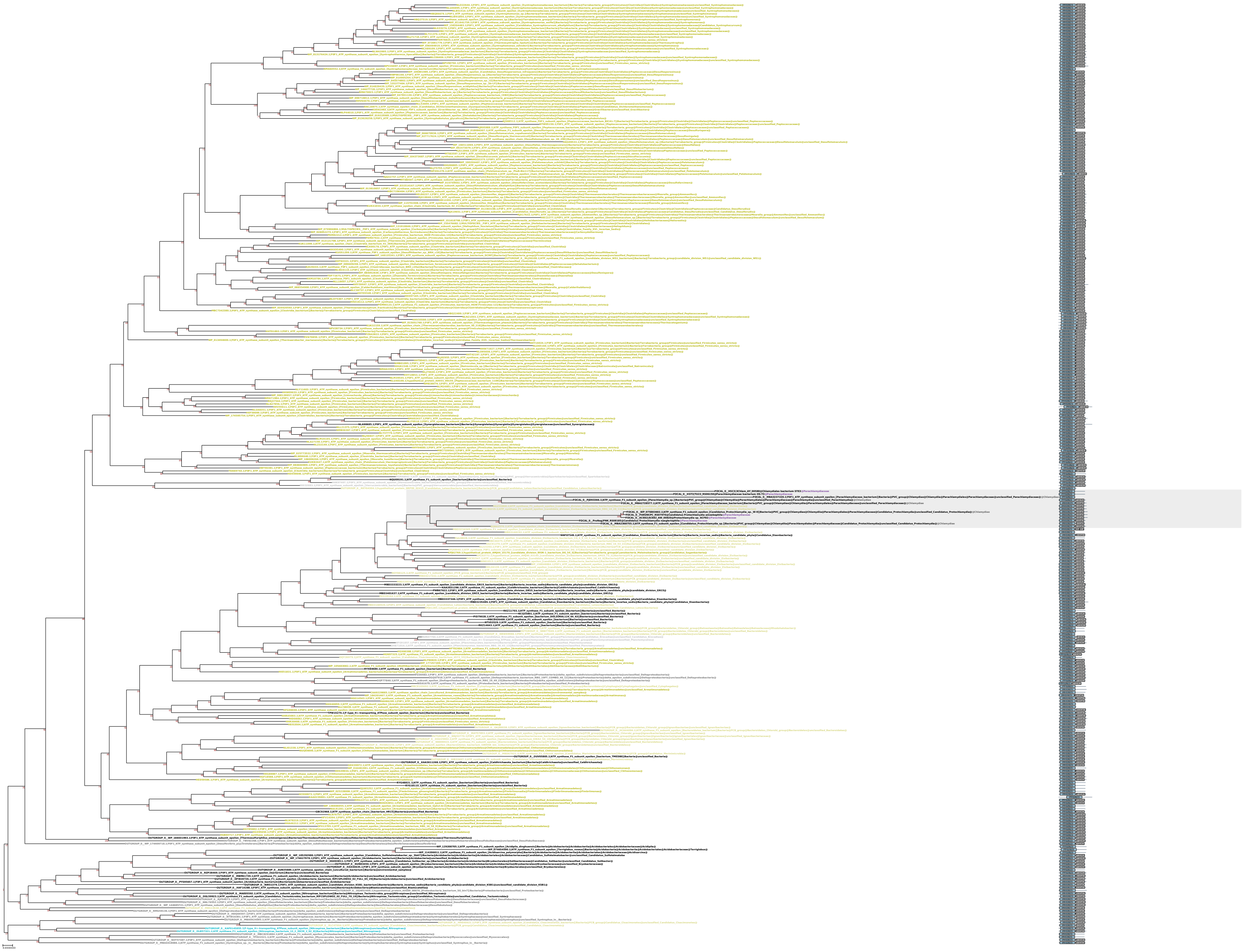

COG0055
atpD
K02112
F-type H+/Na+-transporting ATPase subunit beta

PF00006: ATP synthase alpha/beta family, nucleotide-binding domain
PF02874: ATP synthase alpha/beta family, beta-barrel domain
PF00231: ATP synthase
PF18269: T3SS EscN ATPase C-terminal domain

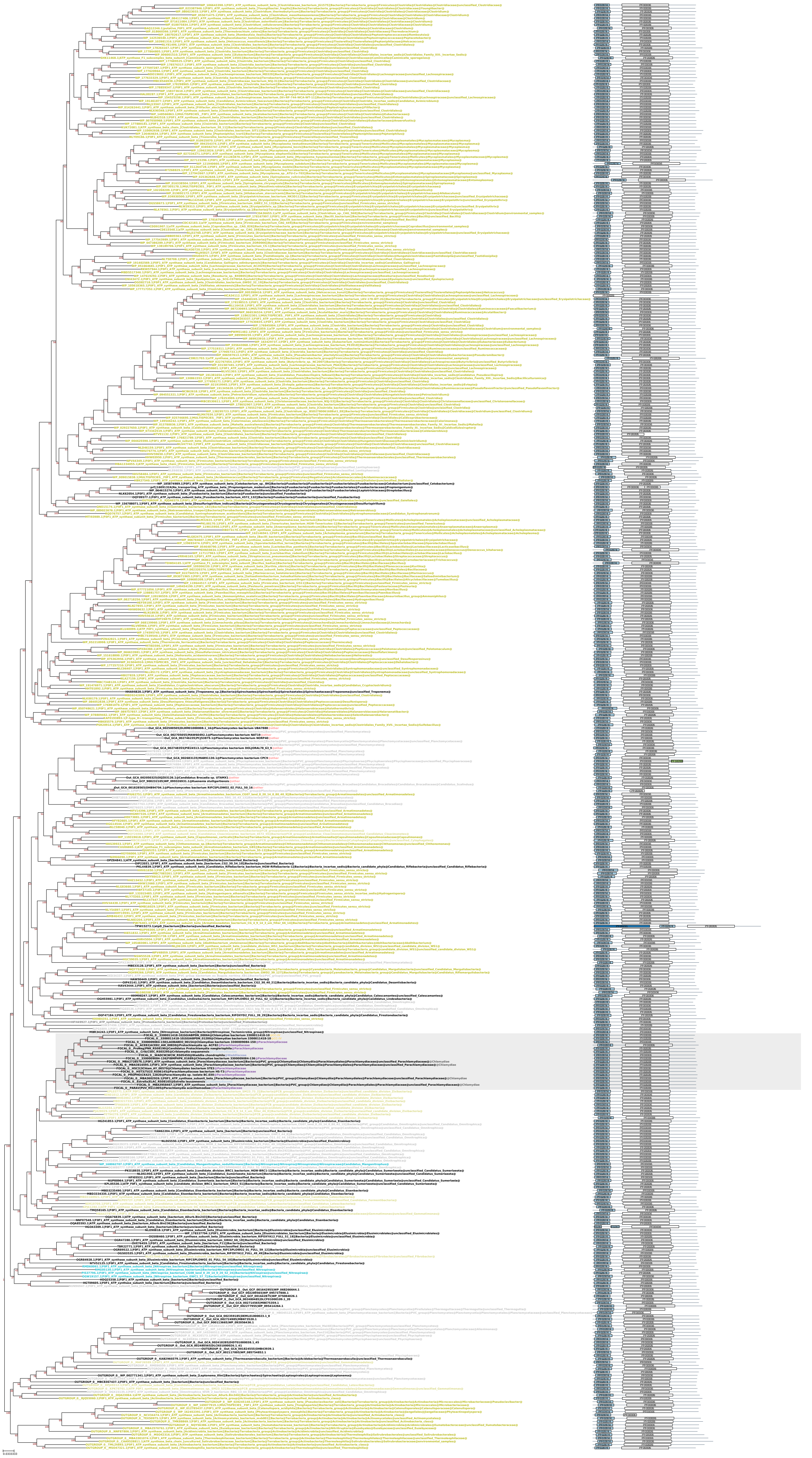

0ZXI6
atpE
K02110
F-type H+-transporting ATPase subunit c

PF00137: ATP synthase subunit C
PF09134: Invasin, domain 3

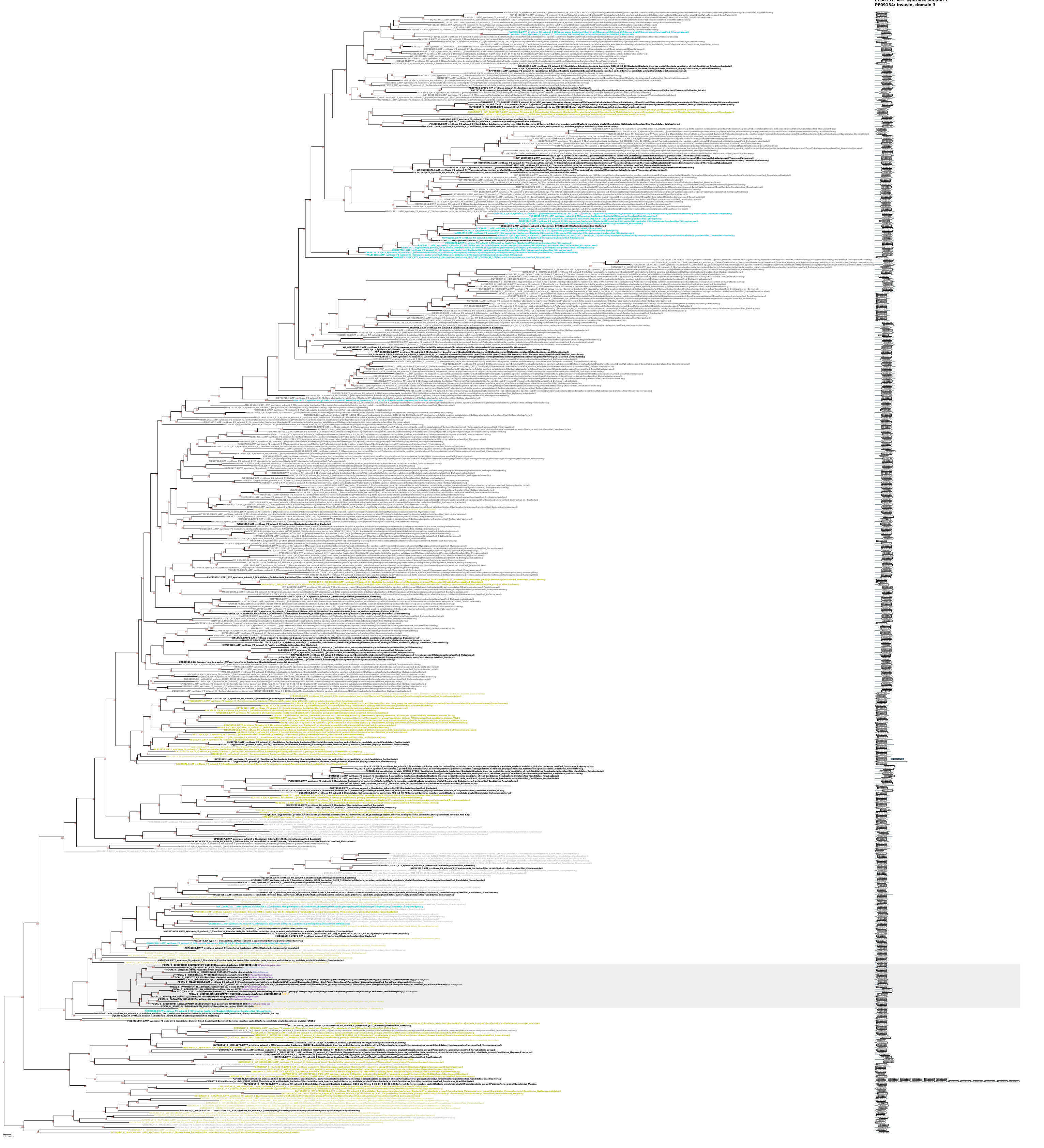

COG0711
atpF
K02109
F-type H+-transporting ATPase subunit b

PF00430: ATP synthase B/B' CF(0)
PF00213: ATP synthase delta (OSCP) subunit
PF04389: Peptidase family M28

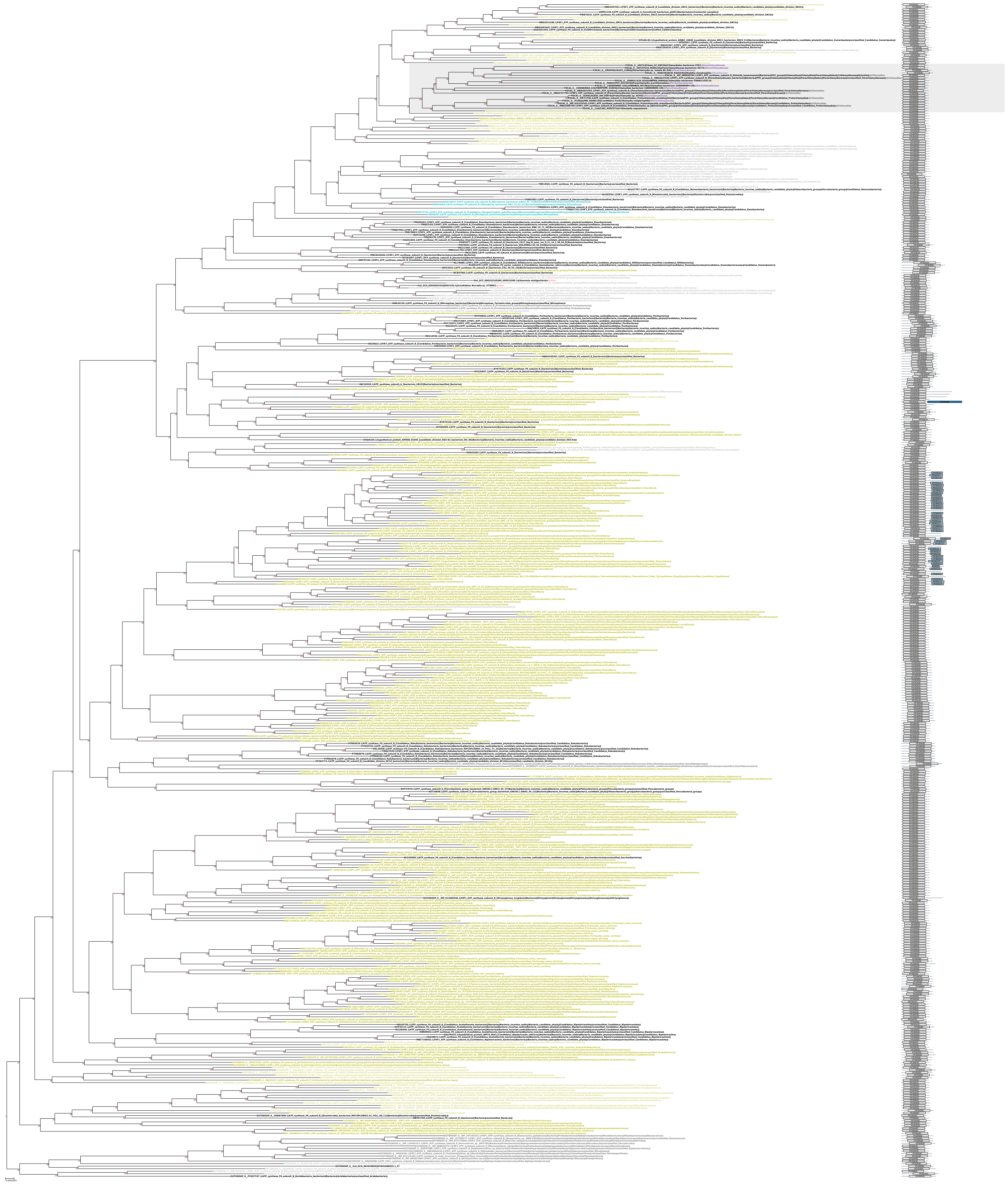

COG0712
atpH
K02113
F-type H+-transporting ATPase subunit delta

PF00213: ATP synthase delta (OSCP) subunit
PF02874: ATP synthase alpha/beta family, beta-barrel domain
PF00006: ATP synthase alpha/beta family, nucleotide-binding domain
PF00306: ATP synthase alpha/beta chain, C terminal domain

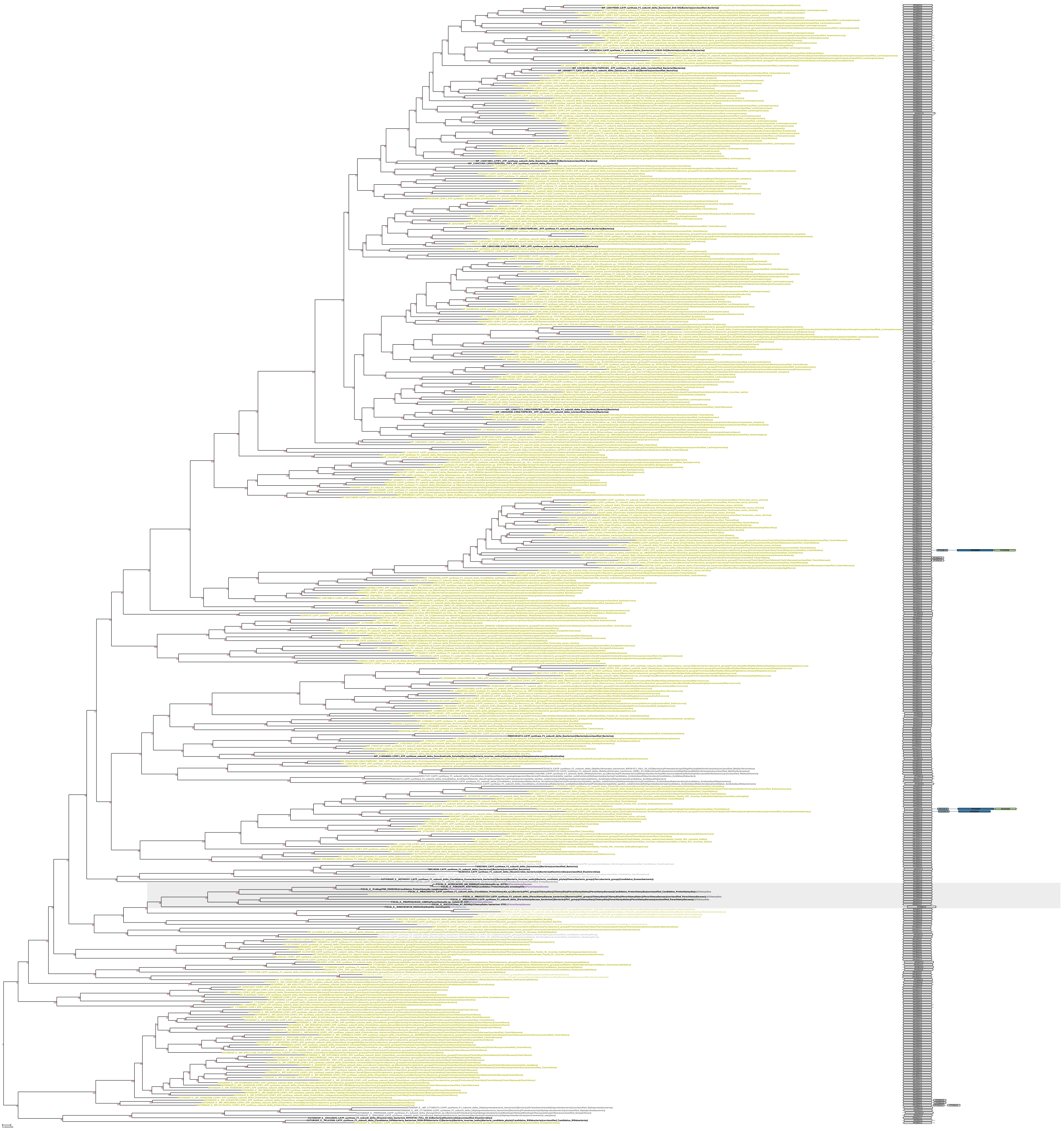

COG1622
cyoA
K02297
cytochrome o ubiquinol oxidase subunit II

PF06481: COX Aromatic Rich Motif
PF02790: Cytochrome C oxidase subunit II, transmembrane domain
PF00116: Cytochrome C oxidase subunit II, periplasmic domain

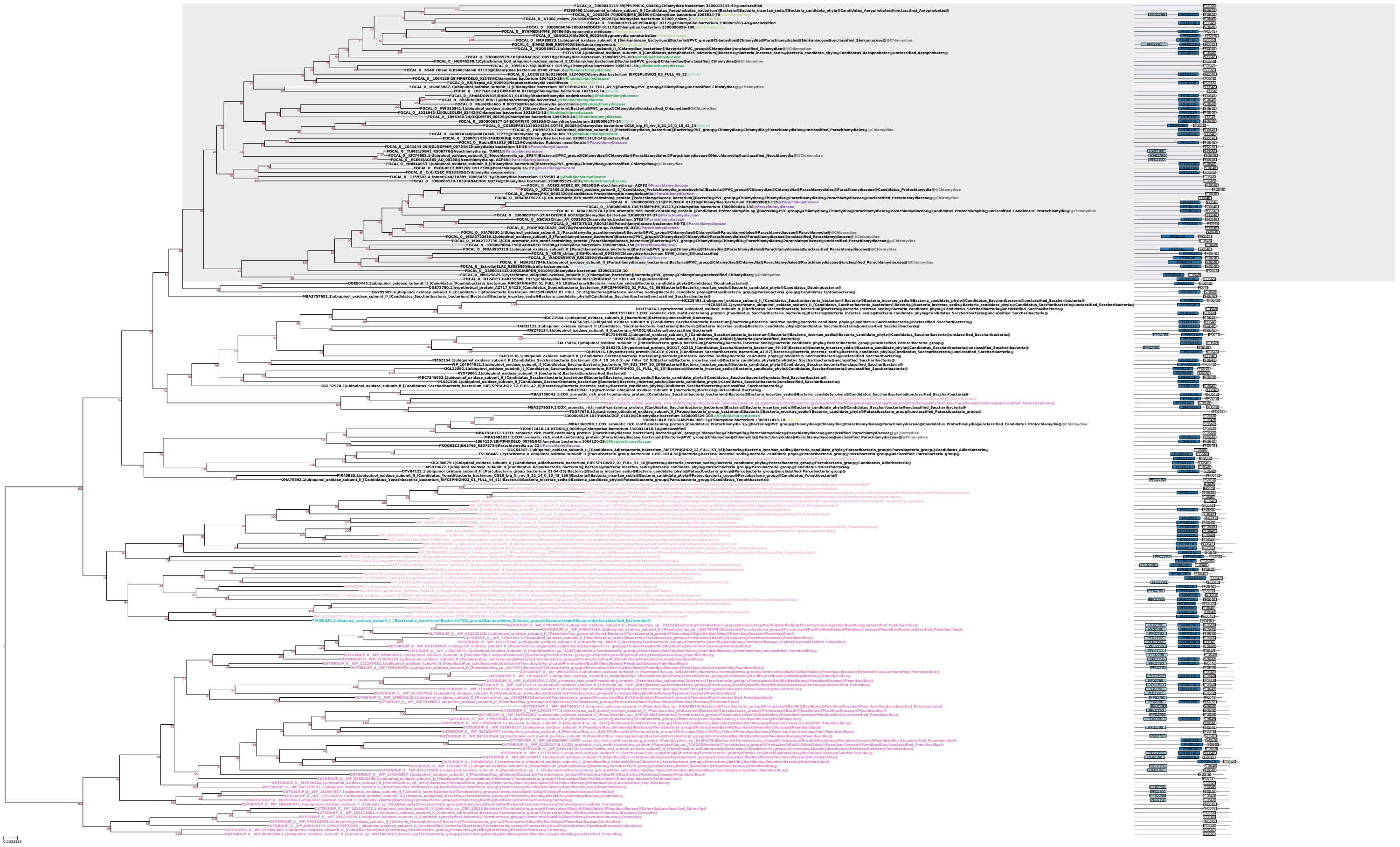

COG0843
cyoB
K02298
cytochrome o ubiquinol oxidase subunit I

PF00115: Cytochrome C and Quinol oxidase polypeptide I
PF12270: Cytochrome c oxidase subunit IV
PF00510: Cytochrome c oxidase subunit III
PF06481: COX Aromatic Rich Motif

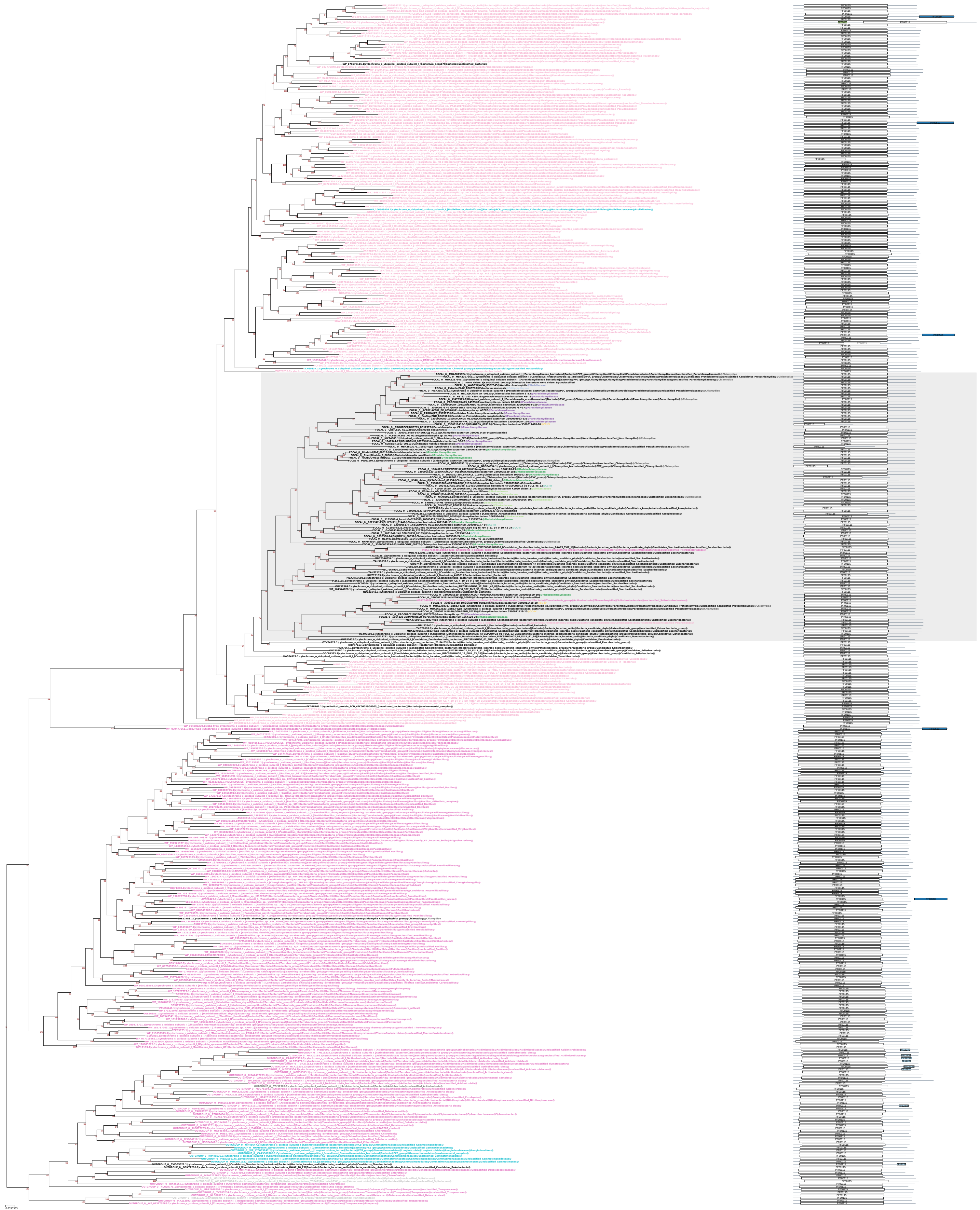

COG1845
cyoC
K02299
cytochrome o ubiquinol oxidase subunit III

PF00510: Cytochrome c oxidase subunit III
PF03626: Prokaryotic Cytochrome C oxidase subunit IV

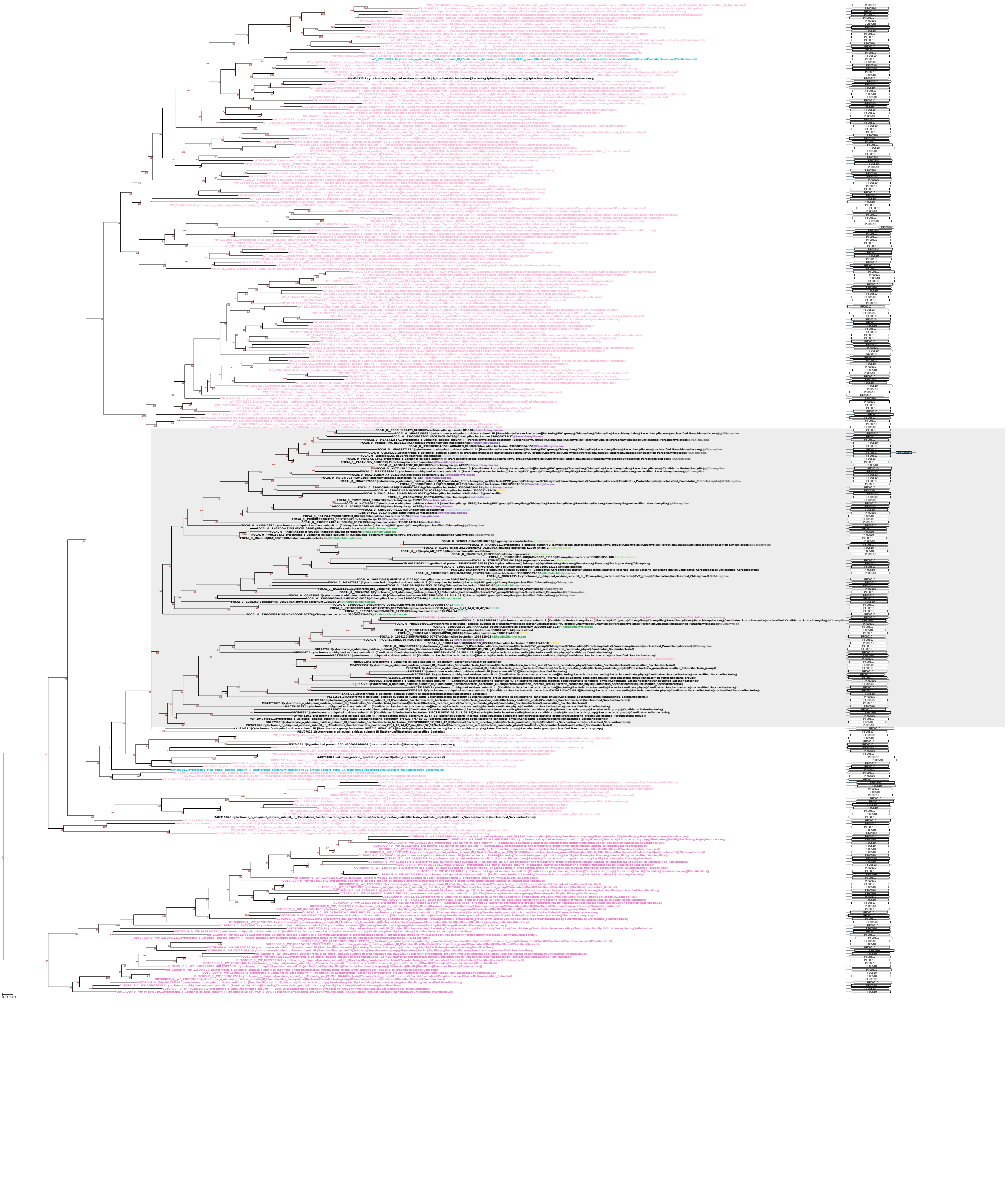

COG3125
cyoD
K02300
cytochrome o ubiquinol oxidase subunit IV

PF03626: Prokaryotic Cytochrome C oxidase subunit IV

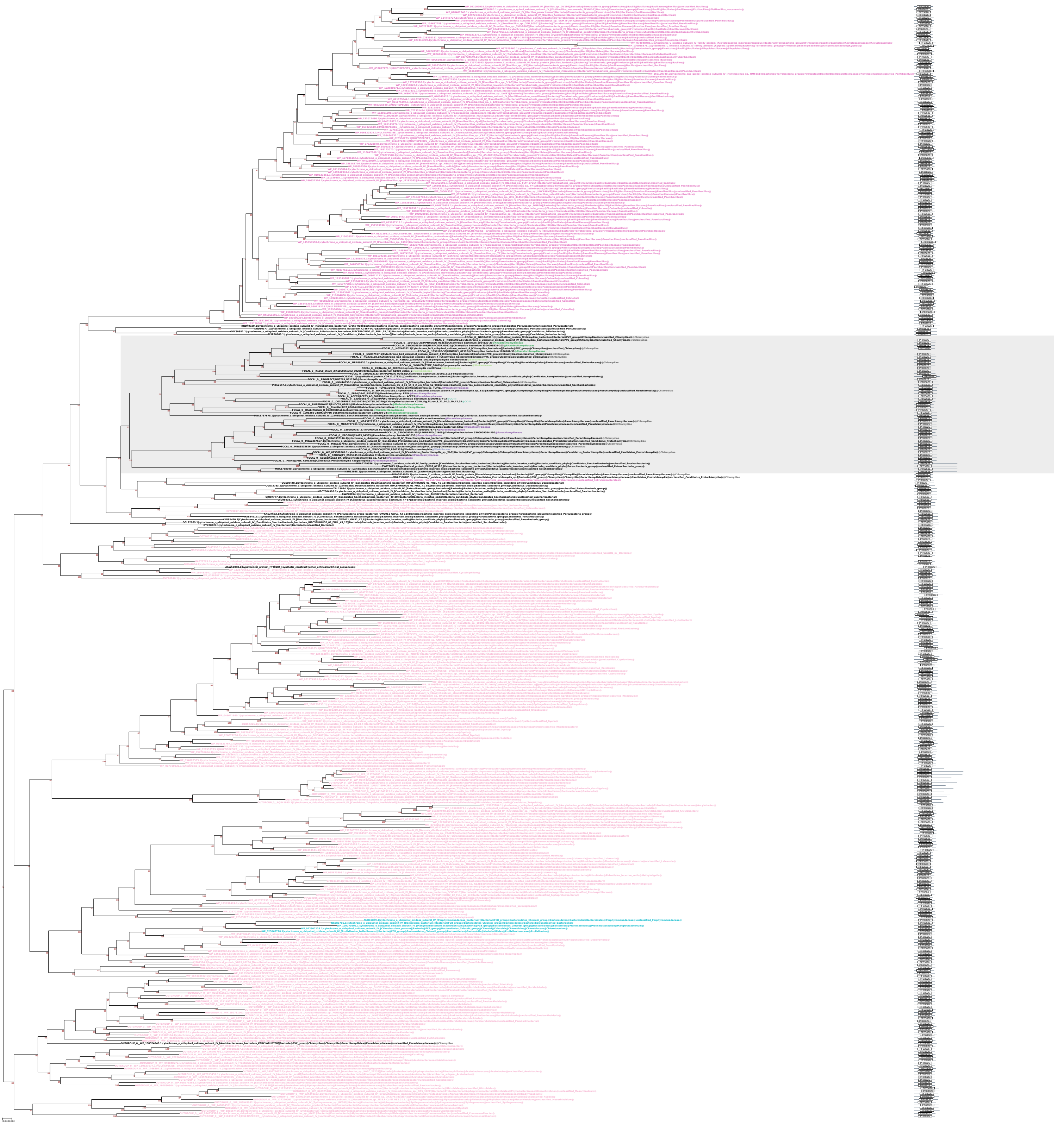

COG0838
nuoA
K00330
NADH-quinone oxidoreductase subunit A

PF00507: NADH-ubiquinone/plastoquinone oxidoreductase, chain 3

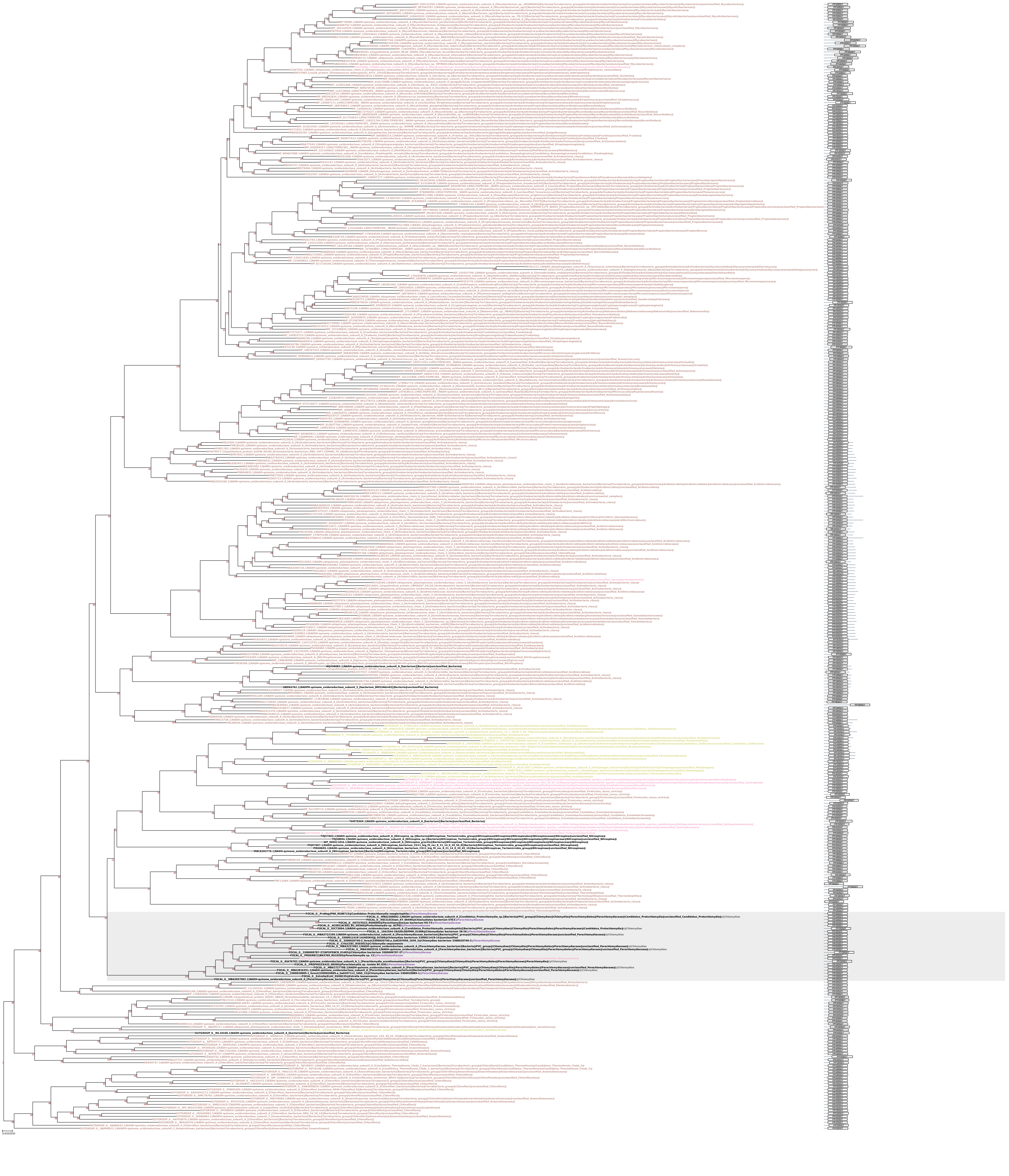

COG0377
nuoB
K00331
NADH-quinone oxidoreductase subunit B

PF01058: NADH ubiquinone oxidoreductase, 20 Kd subunit
PF00507: NADH-ubiquinone/plastoquinone oxidoreductase, chain 3
PF00329: Respiratory-chain NADH dehydrogenase, 30 Kd subunit
PF00346: Respiratory-chain NADH dehydrogenase, 49 Kd subunit
PF15975: Flotillin
PF01145: SPFH domain / Band 7 family

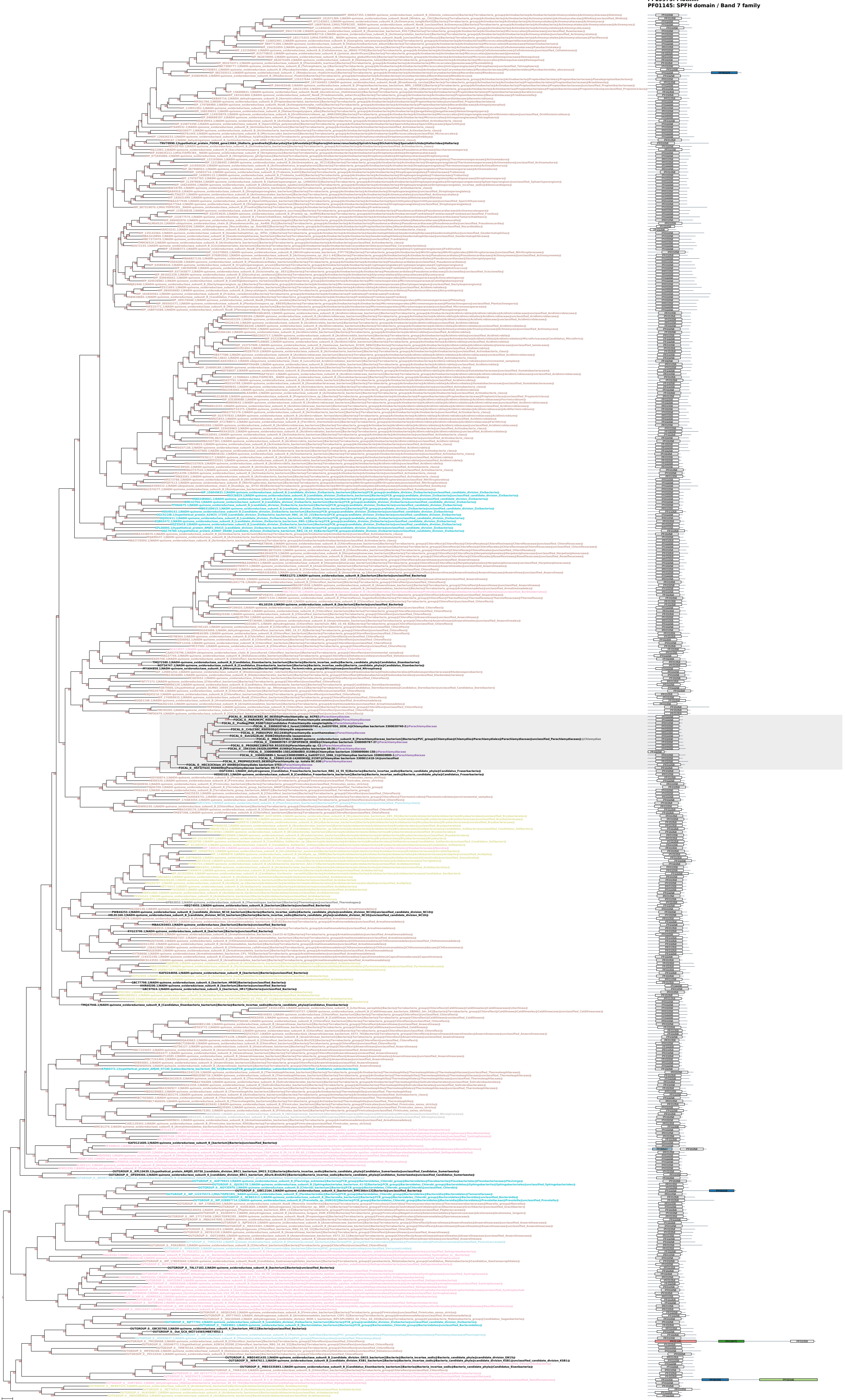

COG0852
nuoC
K00332
NADH-quinone oxidoreductase subunit C

PF00329: Respiratory-chain NADH dehydrogenase, 30 Kd subunit
PF00346: Respiratory-chain NADH dehydrogenase, 49 Kd subunit
PF00374: Nickel-dependent hydrogenase
PF01257: Thioredoxin-like [2Fe-2S] ferredoxin

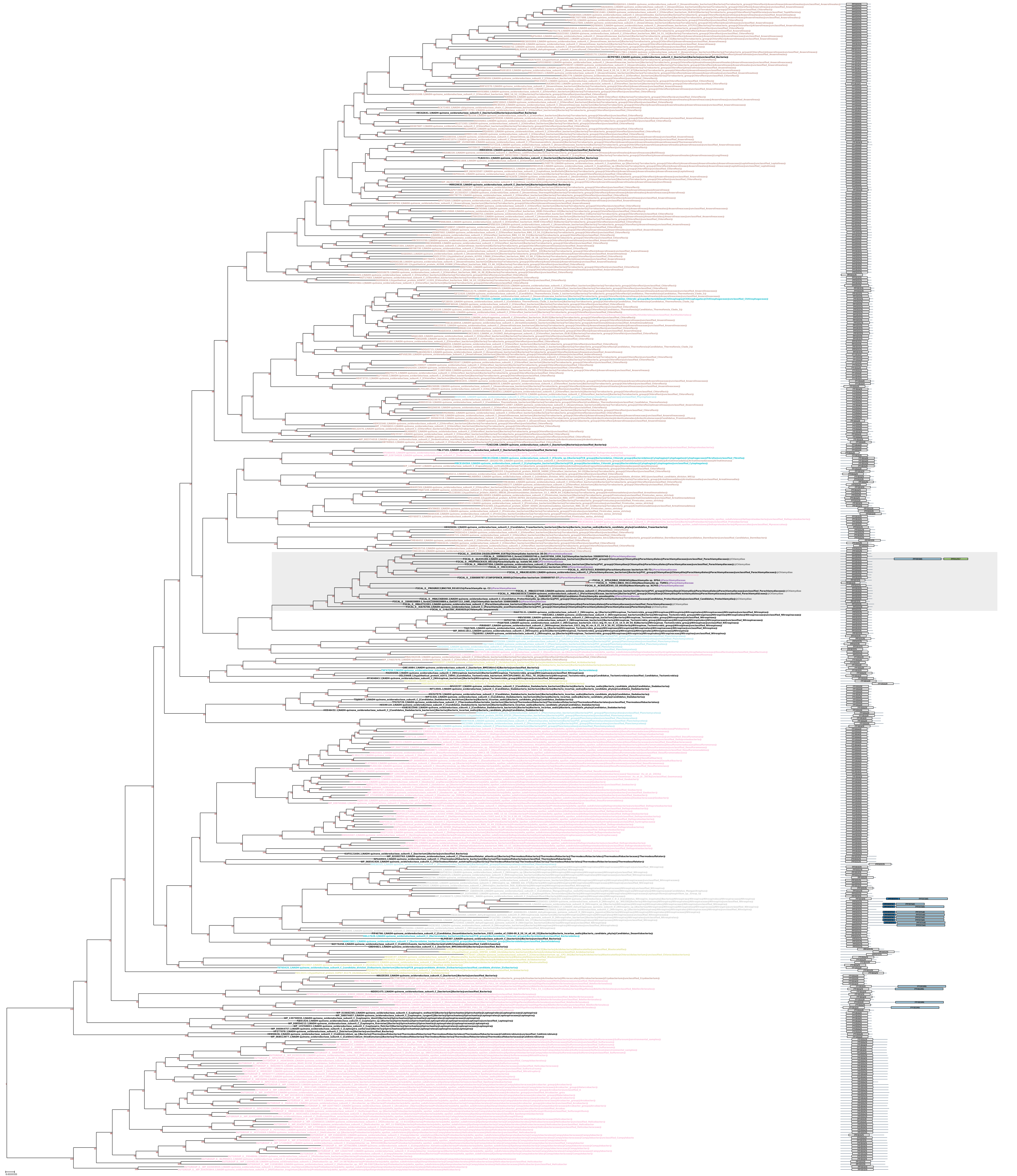

COG0649
nuoD
K00333
NADH-quinone oxidoreductase subunit D

PF00346: Respiratory-chain NADH dehydrogenase, 49 Kd subunit
PF01257: Thioredoxin-like [2Fe-2S] ferredoxin
PF00329: Respiratory-chain NADH dehydrogenase, 30 Kd subunit

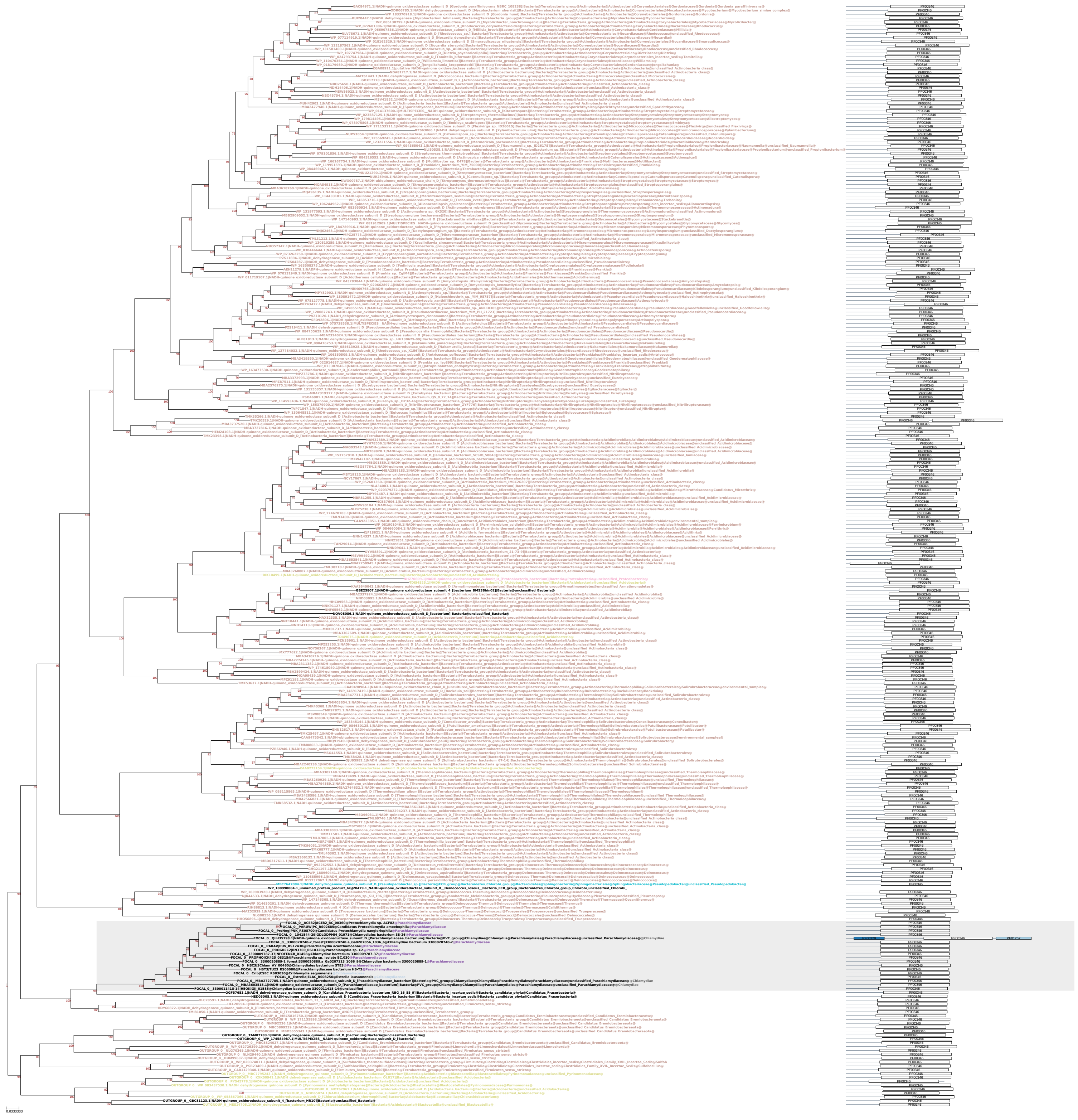

COG1905
nuoE
K00334
NADH-quinone oxidoreductase subunit E

PF01257: Thioredoxin-like [2Fe-2S] ferredoxin
PF01512: Respiratory-chain NADH dehydrogenase 51 Kd subunit
PF10531: SLBB domain
PF10589: NADH-ubiquinone oxidoreductase-F iron-sulfur binding region
PF00329: Respiratory-chain NADH dehydrogenase, 30 Kd subunit
PF00346: Respiratory-chain NADH dehydrogenase, 49 Kd subunit

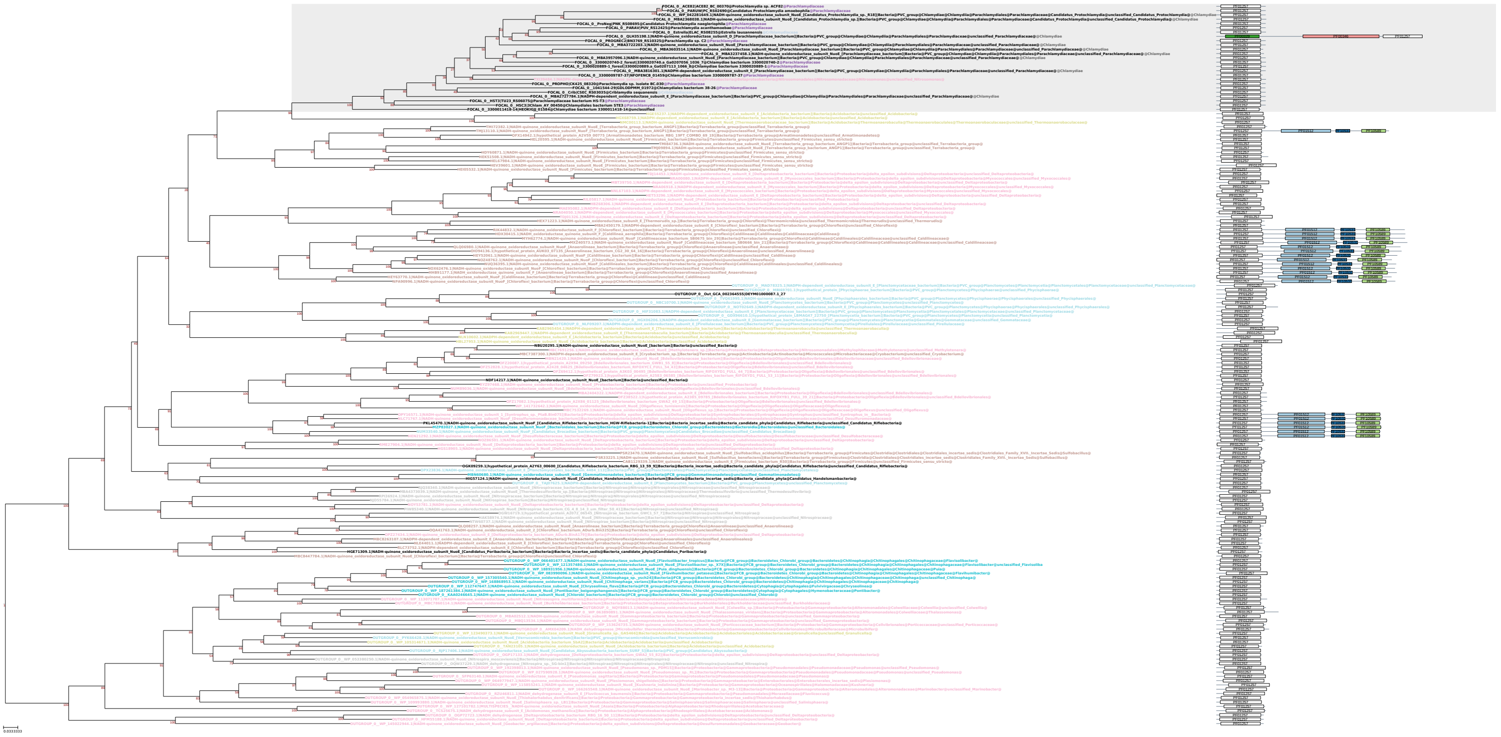

COG1894
nuoF
K00335
NADH-quinone oxidoreductase subunit F

PF10589: NADH-ubiquinone oxidoreductase-F iron-sulfur binding region
PF01512: Respiratory-chain NADH dehydrogenase 51 Kd subunit
PF10531: SLBB domain
PF01257: Thioredoxin-like [2Fe-2S] ferredoxin
PF00072: Response regulator receiver domain

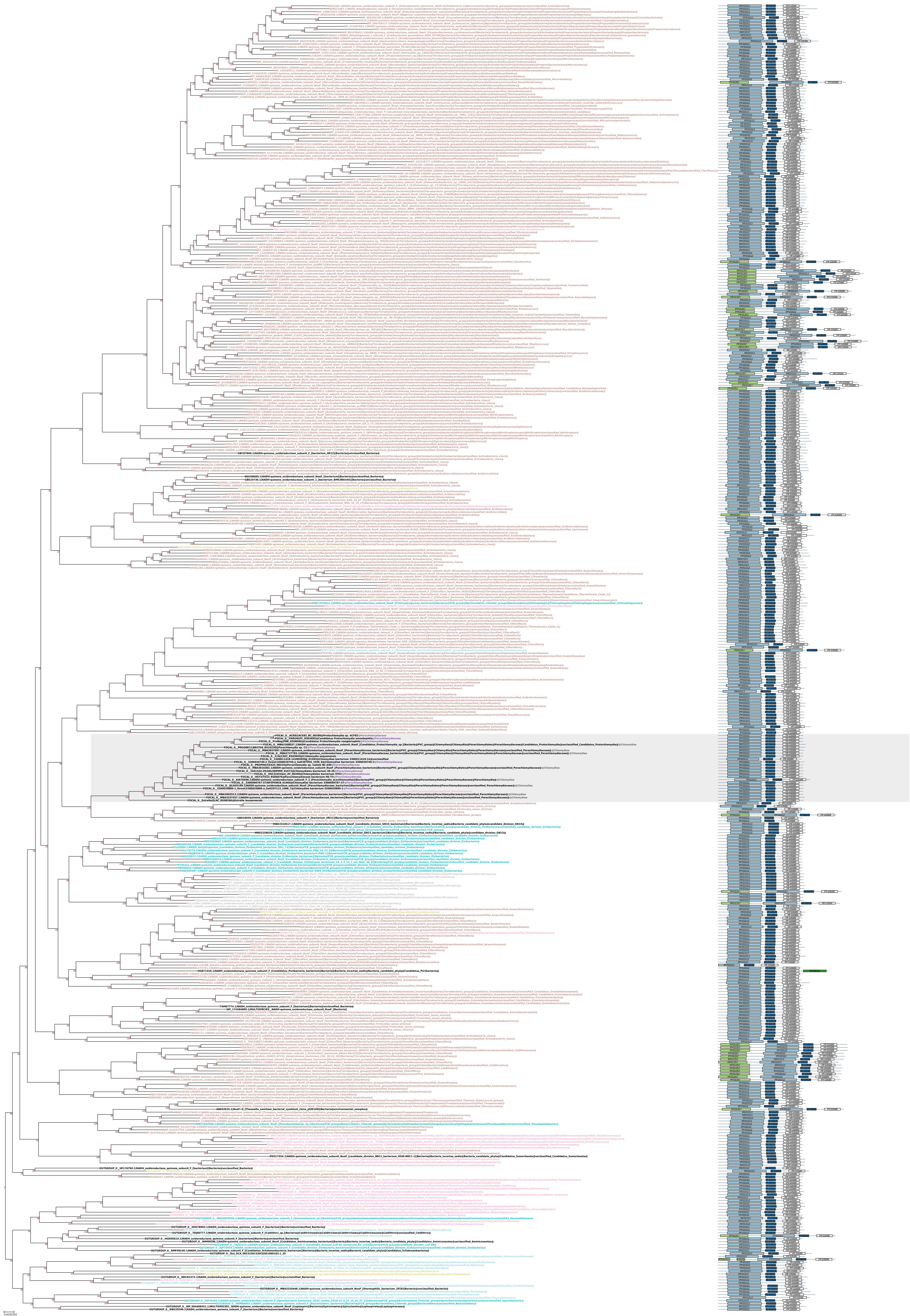

COG3383
nuoG
K00336
NADH-quinone oxidoreductase subunit G

PF00384: Molybdopterin oxidoreductase
PF10588: NADH-ubiquinone oxidoreductase-G iron-sulfur binding region
PF04879: Molybdopterin oxidoreductase Fe4S4 domain
PF13510: 2Fe-2S iron-sulfur cluster binding domain
PF01568: Molydopterin dinucleotide binding domain

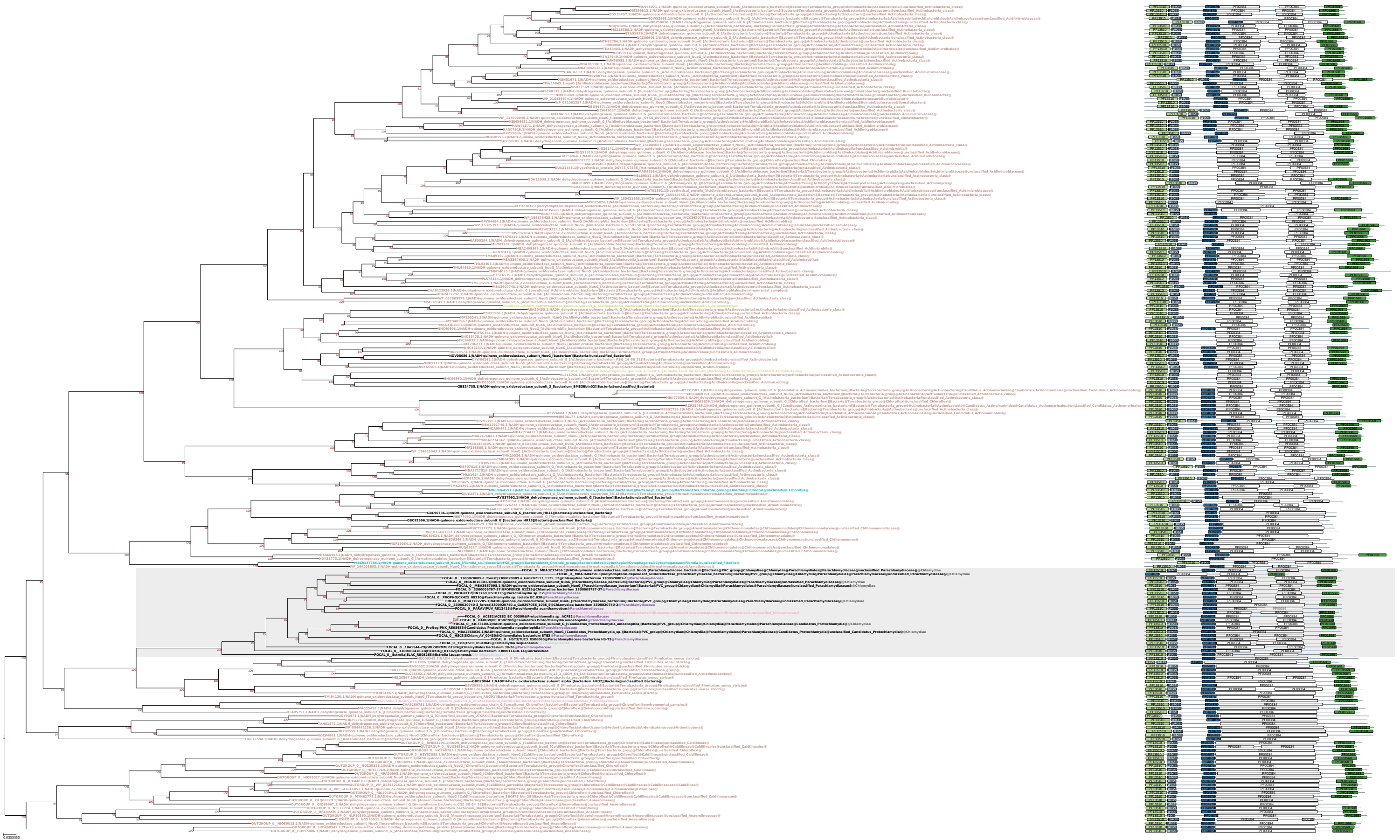

COG1005
nuoH
K00337
NADH-quinone oxidoreductase subunit H

PF00146: NADH dehydrogenase
PF00384: Molybdopterin oxidoreductase
PF05745: Chlamydia 15 kDa cysteine-rich outer membrane protein (CRPA)

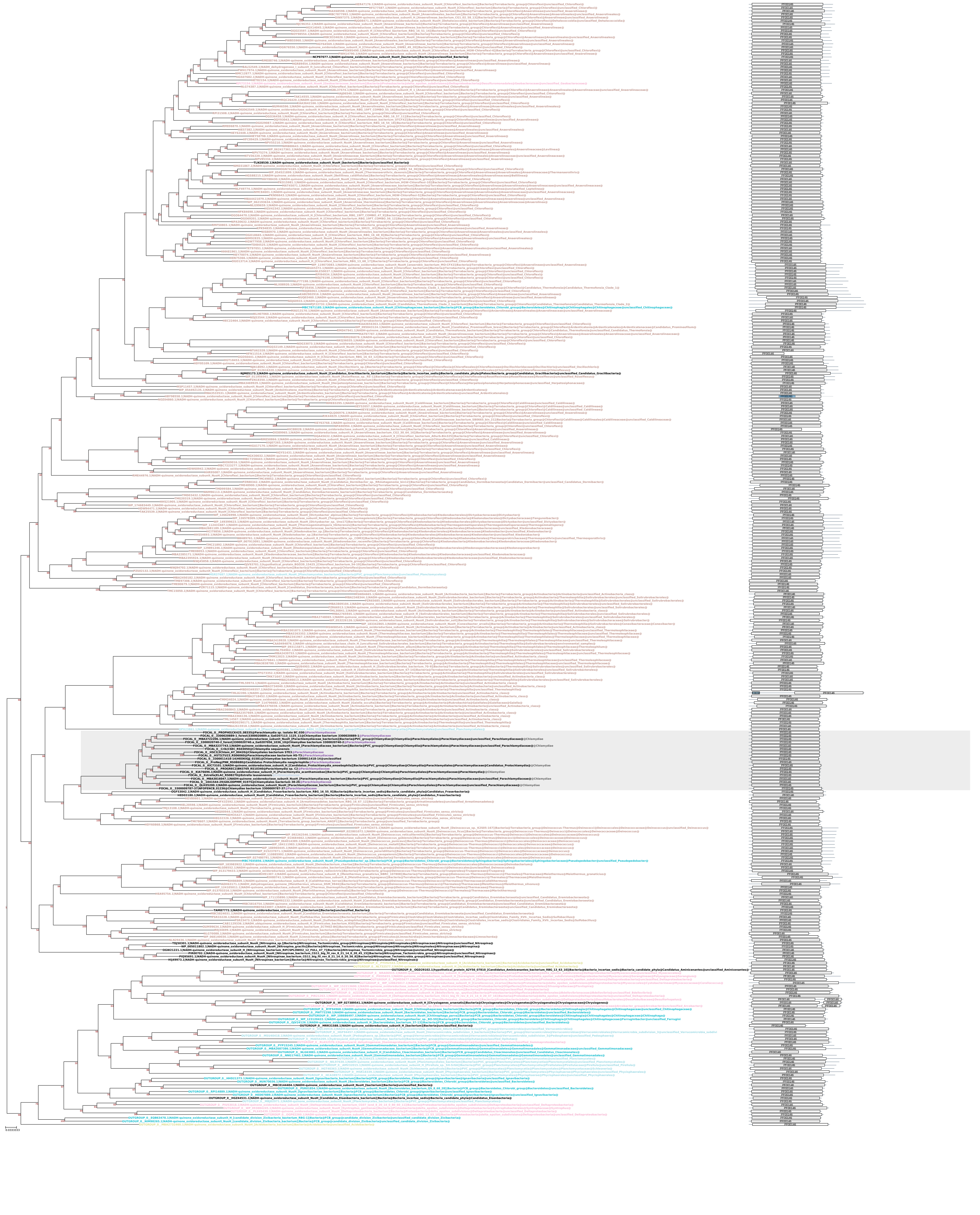

COG1143
nuoI
K00338
NADH-quinone oxidoreductase subunit I

PF12838: 4Fe-4S dicluster domain
PF00037: 4Fe-4S binding domain

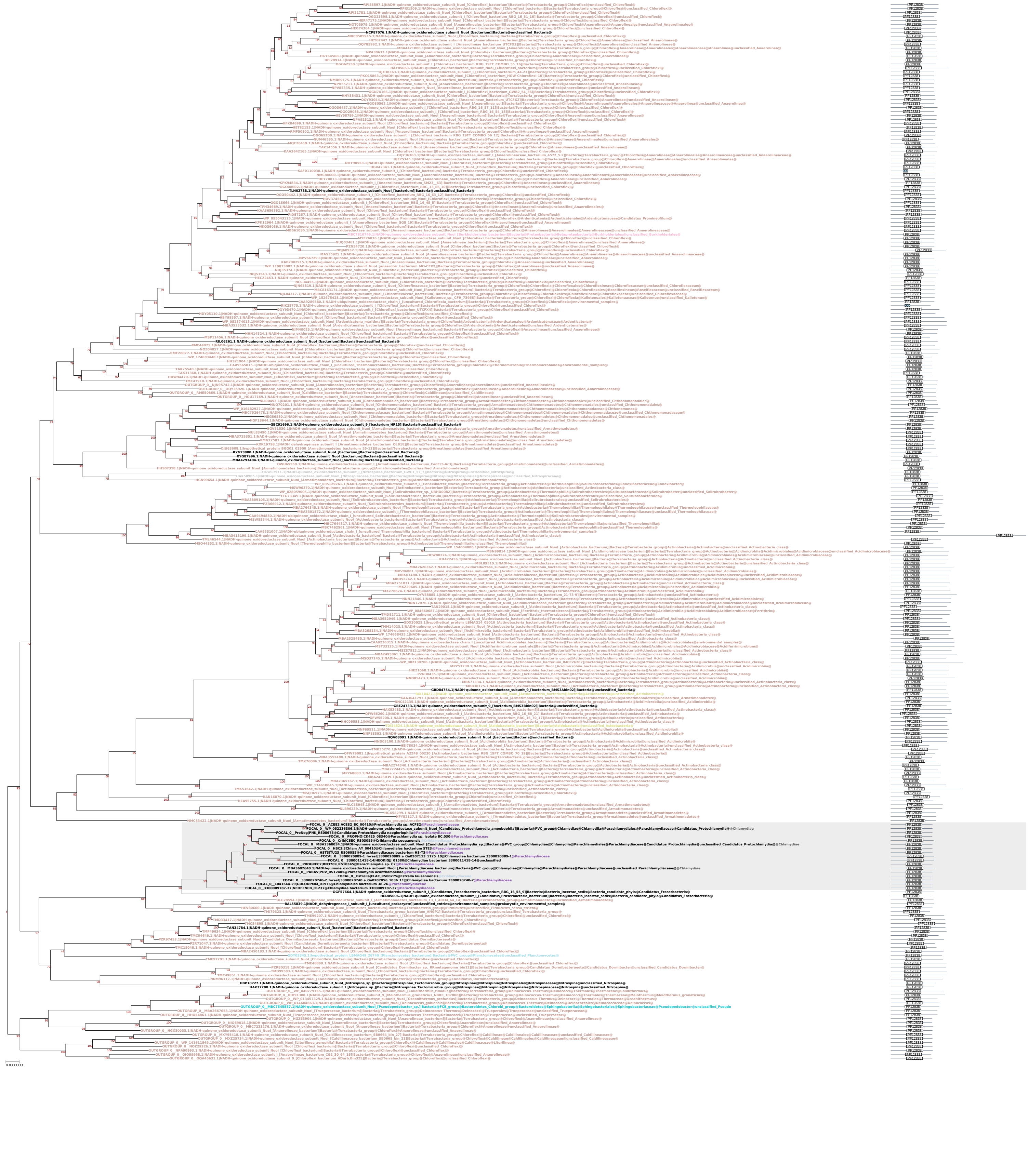

COG0839
nuoJ
K00339
NADH-quinone oxidoreductase subunit J

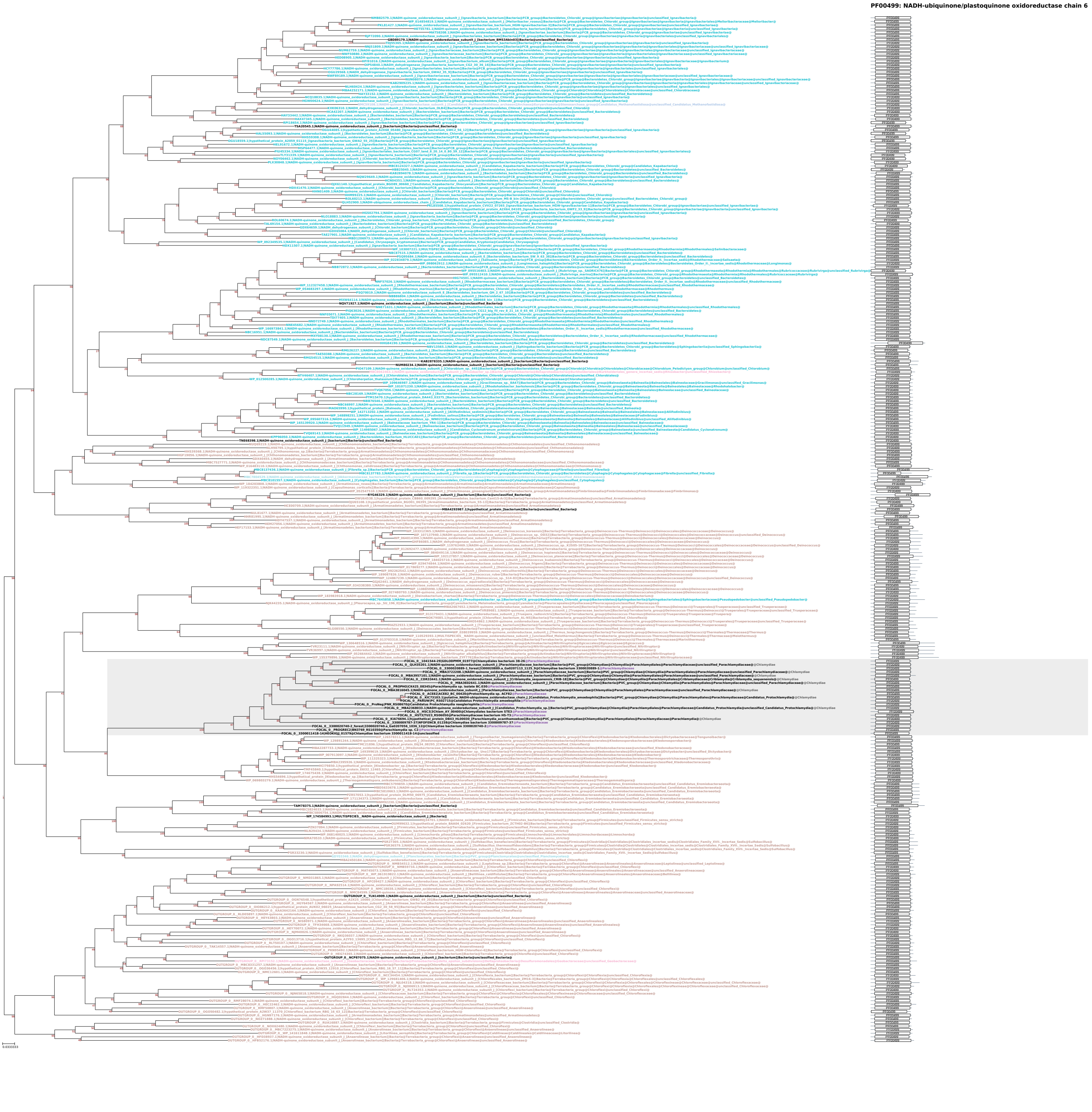

COG0713
nuoK
K00340
NADH-quinone oxidoreductase subunit K

PF00420: NADH-ubiquinone/plastoquinone oxidoreductase chain 4L
PF13385: Concanavalin A-like lectin/glucanases superfamily

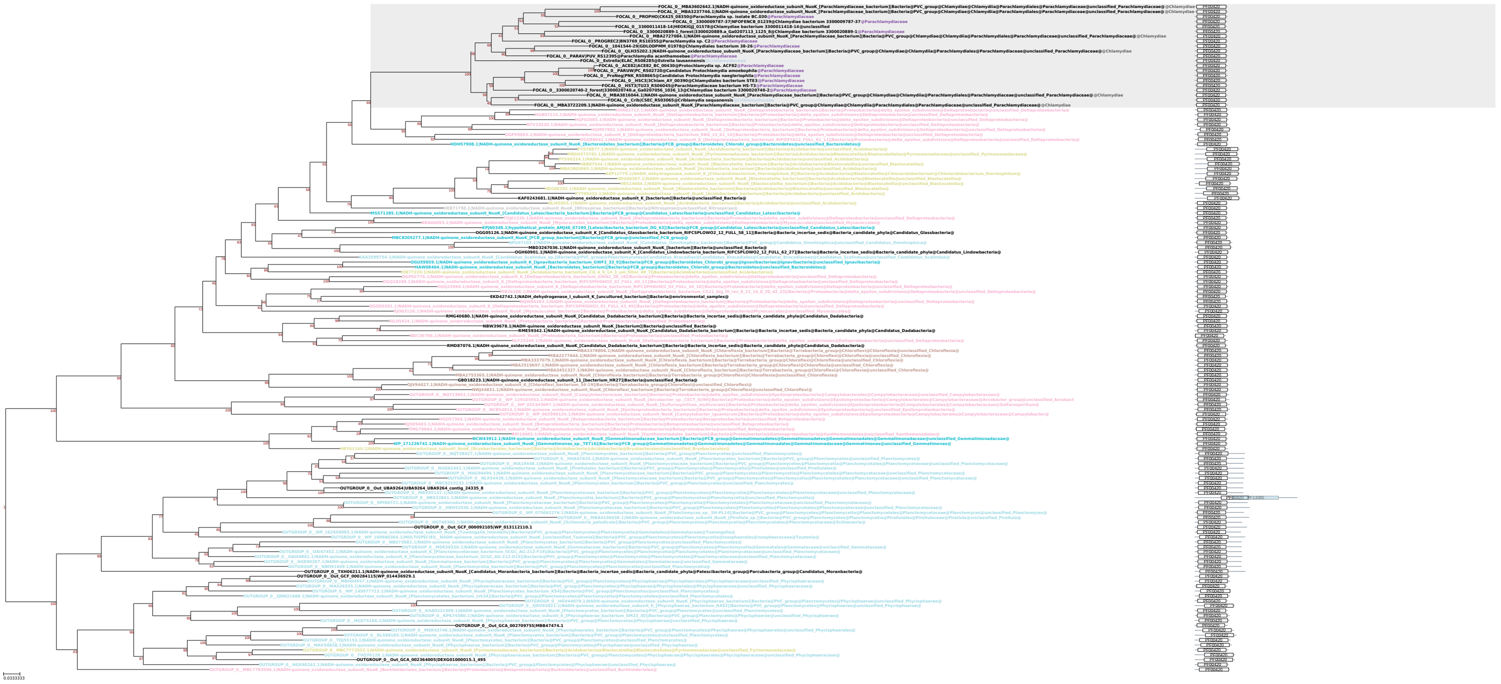

COG1009
nuoL
K00341
NADH-quinone oxidoreductase subunit L

PF00662: NADH-Ubiquinone oxidoreductase (complex I), chain 5 N-terminus
PF00361: Proton-conducting membrane transporter
PF00420: NADH-ubiquinone/plastoquinone oxidoreductase chain 4L
PF01010: NADH-dehyrogenase subunit F, TMs, (complex I) C-terminus

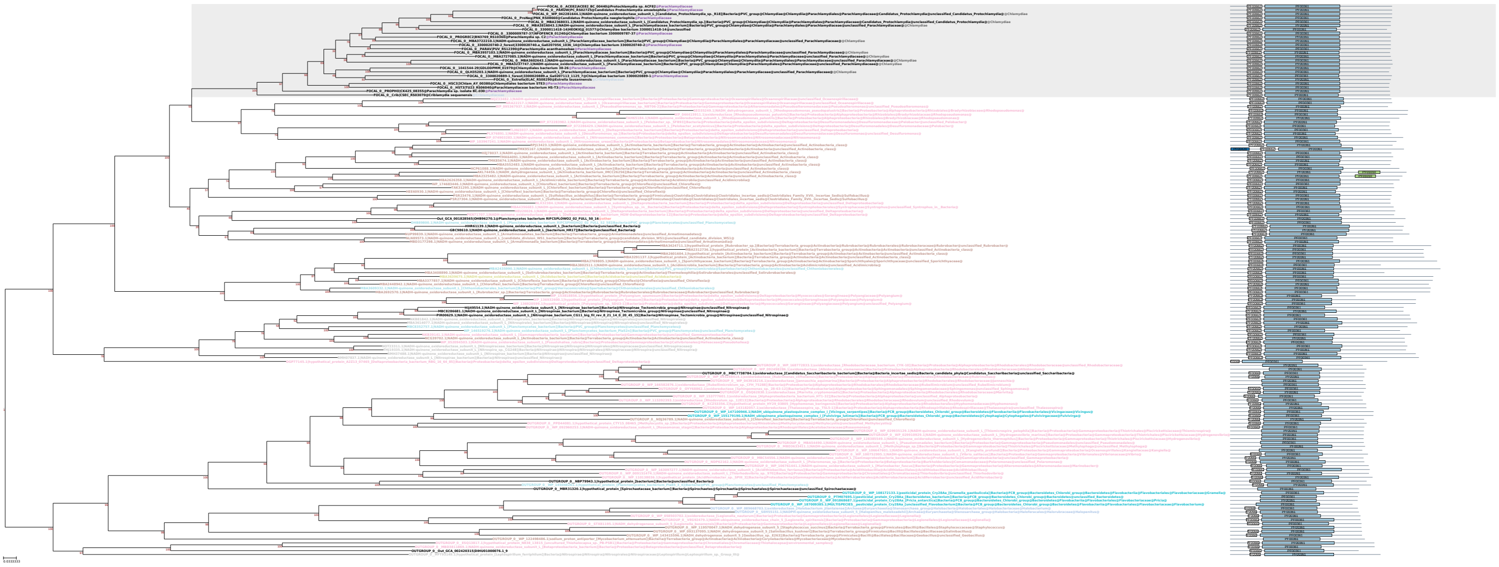

COG1008
nuoM
K00342
NADH-quinone oxidoreductase subunit M

PF00361: Proton-conducting membrane transporter
PF01059: NADH-ubiquinone oxidoreductase chain 4, amino terminus
PF00662: NADH-Ubiquinone oxidoreductase (complex I), chain 5 N-terminus

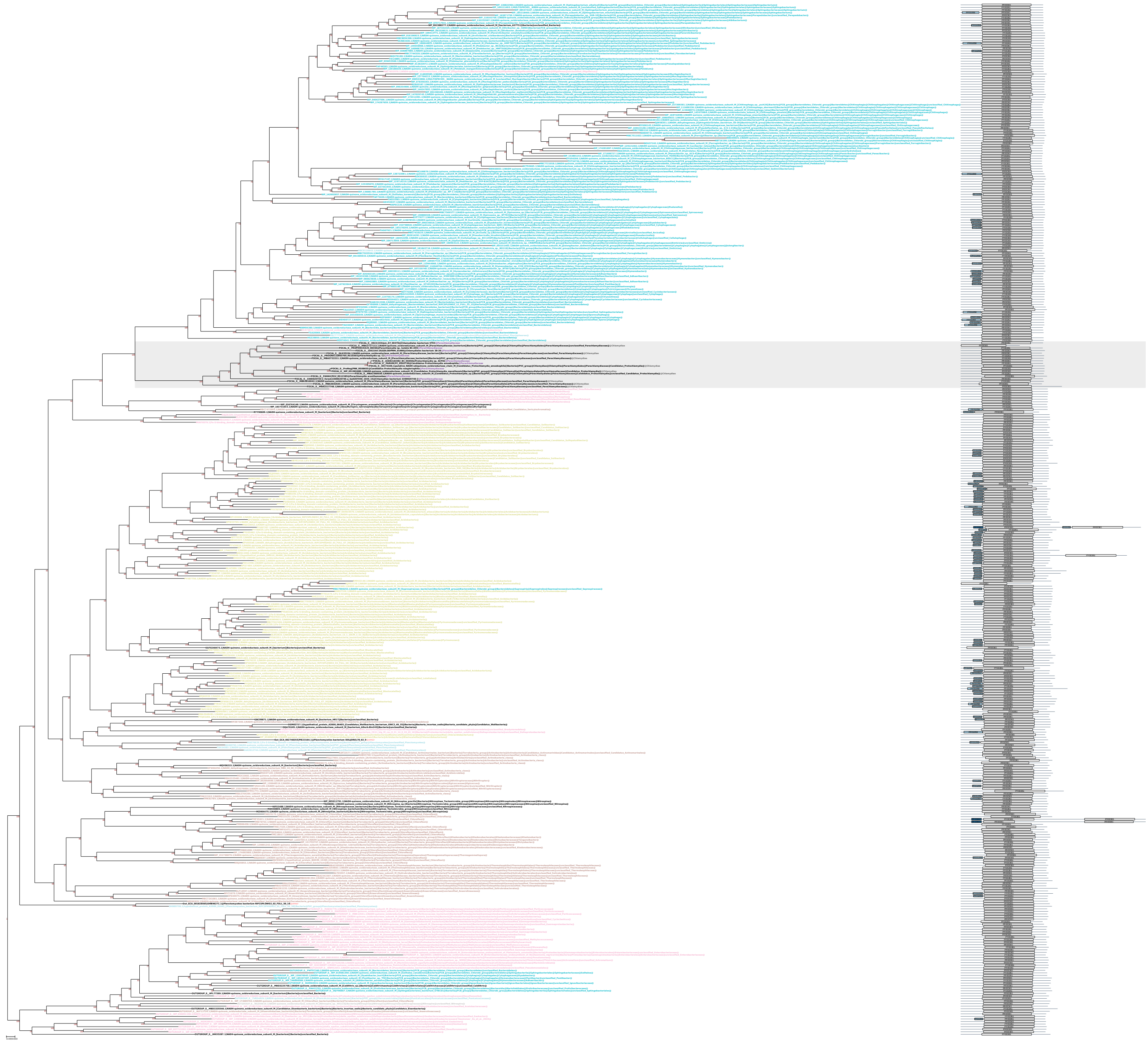

COG1007
nuoN
K00343
NADH-quinone oxidoreductase subunit N

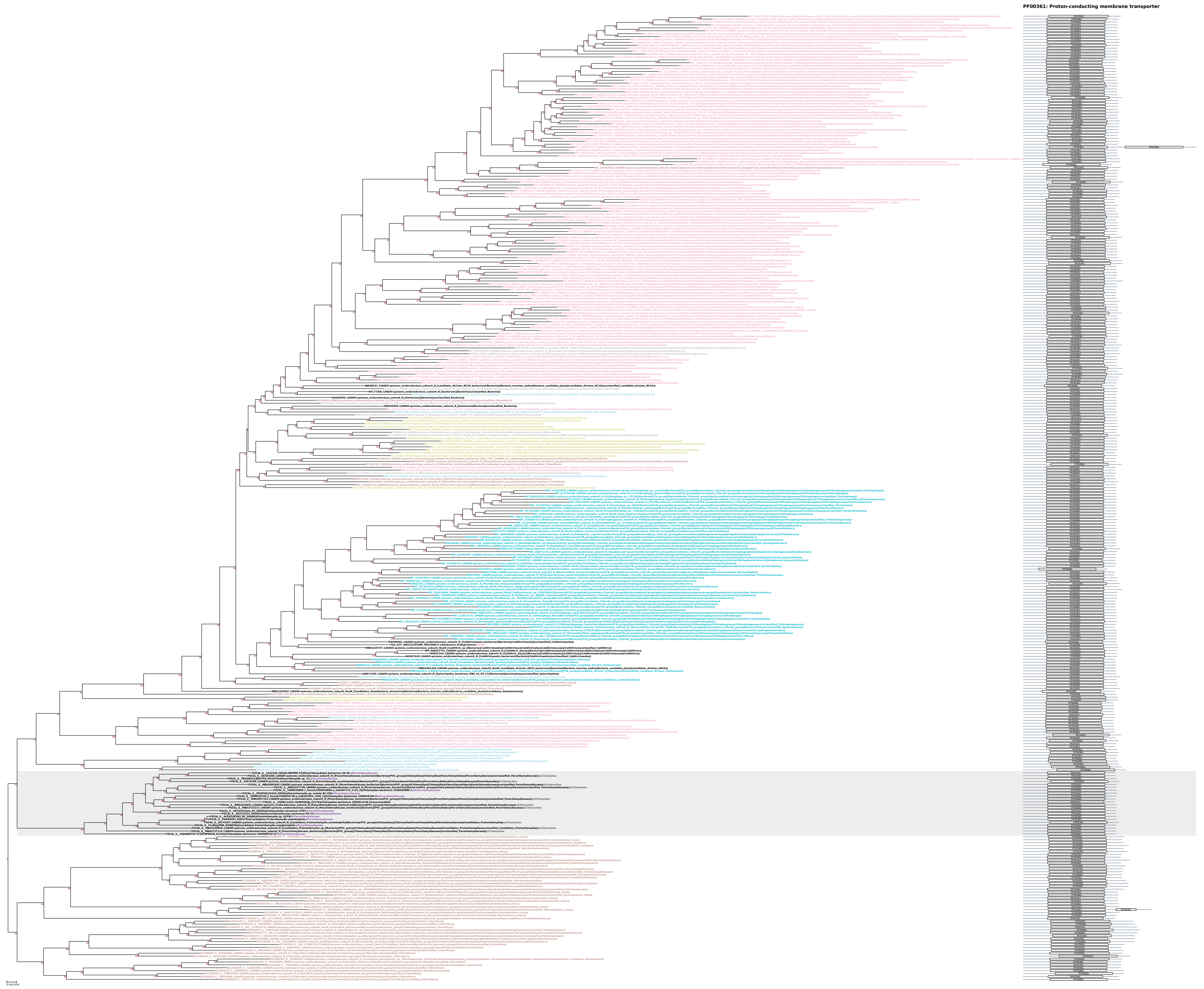

Supplement: Supplementary file 5 — Supplementary Data 6 as outlined in Supplementary Information. [file 41564_2022_1284_MOESM5_ESM.pdf]
