# Supplementary material for: Gene gain facilitated endosymbiotic evolution of Chlamydiae
Source: Nat Microbiol. 2023 Jan 5;8(1):40–54. doi: 10.1038/s41564-022-01284-9 (PMC9816063; doi:10.1038/s41564-022-01284-9)
Supplement: Supplementary file 3 — Peer Review File [file 41564_2022_1284_MOESM3_ESM.doc]

Peer Review Information

**Journal: Nature Microbiology**

**Manuscript Title:**  Gene gain facilitated endosymbiotic evolution of Chlamydiae

**Corresponding author name(s): Professor Thijs Ettema**

**Reviewer Comments & Decisions:**

| **Decision Letter, initial version:** | | | |
| --- | --- | --- | --- |
|  | **Message:** | 28th January 2022  *Please ensure you delete the link to your author homepage in this e-mail if you wish to forward it to your co-authors.  Dear Thijs,  Thank you again for your patience while your manuscript "Gene gain facilitated the origin and diversification of an ancient phylum of symbionts" was under peer-review at Nature Microbiology. Please accept once more my apologies for the delay in getting back to you with a decision, but unfortunately some of the referees were quite delayed in returning their comments to us. Nonetheless, the paper has now been seen by 4 referees, whose expertise and comments you will find at the end of this email. As you will see from the reports, although they find your work of potential interest, they have raised a number of concerns that will need to be addressed before we can consider publication of the work in Nature Microbiology.  In particular, you'll see that while referees #1 and #3, who work more closely with PVC/Chlamydia, are quite enthusiastic about the topic, the other 2 reviewers point out several aspects that will need to be revised in order to better support the main claims and to broaden the appeal of the piece. For example, referee #4 points out the need to revise the introduction and the discussion so that the present findings are better placed with the broader symbiosis literature. Referee #2 provides a very detailed report that, while raising some potential issues linked to the analyses, also provides many suggestions for how to better handle potential biases of the methods used, particularly those employed to infer ancestral gene content. Should further experimental data and text modifications allow you to address these criticisms, we would be happy to look at a revised manuscript.  We are committed to providing a fair and constructive peer-review process so please do not hesitate to contact us if there are specific requests from the reviewers that you believe are technically impossible or unlikely to yield a meaningful outcome.  We strongly support public availability of data. Please place the data used in your paper into a public data repository, if one exists, or alternatively, present the data as Source Data or Supplementary Information. If data can only be shared on request, please explain why in your Data Availability Statement, and also in the correspondence with your editor. For some data types, deposition in a public repository is mandatory - more information on our data deposition policies and available repositories can be found at https://www.nature.com/nature-research/editorial-policies/reporting-standards#availability-of-data.  Please include a data availability statement as a separate section after Methods but before references, under the heading "Data Availability”. This section should inform readers about the availability of the data used to support the conclusions of your study. This information includes accession codes to public repositories (data banks for protein, DNA or RNA sequences, microarray, proteomics data etc…), references to source data published alongside the paper, unique identifiers such as URLs to data repository entries, or data set DOIs, and any other statement about data availability. At a minimum, you should include the following statement: “The data that support the findings of this study are available from the corresponding author upon request”, mentioning any restrictions on availability. If DOIs are provided, we also strongly encourage including these in the Reference list (authors, title, publisher (repository name), identifier, year). For more guidance on how to write this section please see: http://www.nature.com/authors/policies/data/data-availability-statements-data-citations.pdf  When revising your manuscript:  * Include a “Response to referees” document detailing, point-by-point, how you addressed each referee comment. If no action was taken to address a point, you must provide a compelling argument. This response will be sent back to the referees along with the revised manuscript.  * If you have not done so already we suggest that you begin to revise your manuscript so that it conforms to our Article format instructions at http://www.nature.com/nmicrobiol/info/final-submission. Refer also to any guidelines provided in this letter.  * Include a revised version of any required reporting checklist. It will be available to referees (and, potentially, statisticians) to aid in their evaluation if the manuscript goes back for peer review. A revised checklist is essential for re-review of the paper.   When submitting the revised version of your manuscript, please pay close attention to our href="https://www.nature.com/nature-research/editorial-policies/image-integrity">Digital Image Integrity Guidelines.</a> and to the following points below:  -- that unprocessed scans are clearly labelled and match the gels and western blots presented in figures. -- that control panels for gels and western blots are appropriately described as loading on sample processing controls -- all images in the paper are checked for duplication of panels and for splicing of gel lanes.  Finally, please ensure that you retain unprocessed data and metadata files after publication, ideally archiving data in perpetuity, as these may be requested during the peer review and production process or after publication if any issues arise.   [redacted] Nature Microbiology is committed to improving transparency in authorship. As part of our efforts in this direction, we are now requesting that all authors identified as ‘corresponding author’ on published papers create and link their Open Researcher and Contributor Identifier (ORCID) with their account on the Manuscript Tracking System (MTS), prior to acceptance. This applies to primary research papers only. ORCID helps the scientific community achieve unambiguous attribution of all scholarly contributions. You can create and link your ORCID from the home page of the MTS by clicking on ‘Modify my Springer Nature account’. For more information please visit please visit <a href="http://www.springernature.com/orcid">www.springernature.com/orcid</a>.  If you wish to submit a suitably revised manuscript we would hope to receive it within 6 months. If you cannot send it within this time, please let us know. We will be happy to consider your revision, even if a similar study has been accepted for publication at Nature Microbiology or published elsewhere (up to a maximum of 6 months).  In the meantime we hope that you find our referees' comments helpful.  [redacted]  ***************************************************** Reviewer Expertise:  Referee #1: Chlamydia genomics Referee #2: Microbial genome evolution Referee #3: PVC biology Referee #4: Microbial symbiosis; evolution  ***************************************************** Reviewer Comments:  Reviewer #1 (Remarks to the Author):  Having now read the full manuscript, I feel that I am probably not a suitable reviewer. The manuscript is highly dependent on understanding and validating the complex phylogenomics methods that have been used. I am not able to do this as I am not sufficiently expert in the area of advanced bacterial phylogenomics. The authors have done an excellent job of interpreting and extending the evolutionary hypotheses. However, without having confidence in the methodologies used, any interpretation as not valid.  The question addressed in this manuscript though is very relevant and intriguing. Chlamydia is an extremely interesting and important group of organisms with a unique adaptation to intracellular symbiosis. The topic of the manuscript therefore is extremely novel and interesting and will be of broad interest.   ***************************************************** Reviewer #2 (Remarks to the Author):  This manuscript describes an extensive analysis of genomes of Chlamydiae (and other in the broader PVC clade) in view of understanding the evolution of gene repertoires from a presumably free-living ancestor to an obligatory intracellular pathogen. This clade contains bacteria that are obligate pathogens and others that are environmental. It has spurred significant interest because it provides an interesting group to understand the evolution of intracellular pathogenesis using mechanisms that have probably evolved to protect (or infect) protozoa. Here, the authors use phylogenetic inference and reconstruction of ancestral states to track the evolution of the genes in the clade (in terms of presence/acquisition). The authors draw three major conclusions. 1) That the process of evolution was characterized not just by extensive loss, but also by genome expansion by horizontal transfer. 2) that the ancestor gained the ability to infect eukaryotic hosts over a billion years ago. 3) That this corresponded to transitions between types of energy metabolism. Some of the results associated with claim #1 may be the result of a reconstruction artifact. The claim #2 does not seem very novel relative to previous works of the authors (confirmatory). The extensive and careful analyses behind claim #3 are interesting and important for the researchers in the field. The paper is well written. I have one very strong criticism, a few major suggestions and several minor ones. Most of these concern claim #1. Major problem: The reconstruction of ancestral states was done using state-of-the-art tools. But the authors seem unaware that this type of analyses allows to infer the presence of genes in the ancestor, but not the size of genomes (in terms of the number of genes). This results in an artifact that challenges several major results supporting claim #1. The major problem is that if one takes extant genomes and one reconstructs the gene set of ancestors one always gets a biased downwards estimate of the number of genes. This means that the size of the gene set is smaller than it should be. This affects the conclusions drawn from figs 3 and 5. The reason is well known, although possibly not sufficiently advertised: because some proteins evolve fast and especially because some protein families are prone to a lot of transfer and loss, they are not inferred to be present back in the tree. Let's take an example: many species have many transposable elements. Each family of these elements is usually present in a few strains of a species. But most genomes have at least one family. The inference of ancestral states in such cases is that all these elements are absent from the ancestors. This is not just a problem of accuracy, there is a systematic bias that is true for any method (ML, Bayesian, etc). The problem of the bias in inferring gains and losses is clear in Fig 3. For a clade that is supposed to have endured genome reduction it's odd that all but one branch reveal more gains than losses. This is a problem, because this bias may explain some of the claims that the clade has had a more complex evolution of gene repertoires than expected. This is of course also a huge problem for the inference of the rates of horizontal transfer, because it means that the transfer of the most transferred families of genes is heavily under-estimated. The resulting rates of transfer are very conservative and, more problematic, they are downwards biased and more so for ancient events: the more one goes back in time the most likely it is that they are lower than the real value. This has a deeper and even more problematic effect when the authors take the inferred sets of ancestral genes as a proxy of the size of gene repertoires in the ancestors. This is used to make assumptions about reductive evolution, etc. The problem is that, once again, the more one goes back in time the worst is this proxy. And the more biased it is (the effect is that genomes sizes get more and more underestimated even if genome sizes were unchanged). If one takes the example above, the ancestral genomes will lack most transposable elements and therefore will be inferred as being smaller than they actually were. Fortunately, in the specific case of genome size there is a way of estimating how much is lost in the analysis. The authors should take the tree and the values of genome size (or the number of protein coding genes) and infer ancestral sizes in internal nodes in the tree. Then they should compute the difference between the number of genes inferred in their analysis and the number of genes in the genome inferred by this new analysis. This will reveal and quantify the bias. Other major issues: - The problems mentioned above in terms of identification of horizontal gene transfer are amplified by the use of MAGs, which are well known to miss many mobile elements. This makes the analysis of horizontal transfer even less accurate. - Several clades were removed from the analysis because they had extreme genomes or were hard to place. Reconstruction of ancestral states is very sensitive to non-random sampling, especially when clades have heterogeneous distributions of traits, as it is the case in this study. I would have liked to know how the most important results are affected by the inclusion of these clades. I realize that some may produce instabilities in the tree, but the use of Bayesian methods allows to account for that. It is important to understand the effect of these decisions on the major results. -The use of gene species-species trees reconstructions to infer the rates of transfer has recently been criticized as producing biased estimates and this should be commented (Tria, GBE, 21). Minor issues: -The inaccuracy of using MAGs is amplified by the fact that they are not randomly dispersed in the tree, but instead they cluster in poorly known clades. I do realize that this is necessary, MAGs are used exactly because they are in poorly sampled parts of the tree. But a note of caution is needed. - L52. It seems to me that the claim " the Chlamydiae ancestor had already obtained key genomic features associated with a symbiotic lifestyle" has been done several times for other traits by the same group. Maybe I missed the point. If not, this should be specified and the claim in the abstract could be toned down. -It is not always clear to me why the manuscript has so many different ways of doing seemingly similar analyses. I would understand that the CAT model can't be used in very single case, but some analyses are done with complex ML methods, whereas others are done using very simplified (and not very accurate) methods. For example, fig S1. Also, sometimes trimming is done with BMGE and in others with chi2 trimming (none of which is among the best in the benchmarks). -L116. Here and elsewhere the use of LCCA is ambiguous. It sometimes refers to its gene repertoires, which is fine. But sometimes is used as the genes acquired by LCCA. I assume these are the genes acquired in the branch between this node and the one above in the tree and for which we have evidence in LCCA. The difference between past genomes and past lineages should be clarified. -L243. The indication of figure 2 as evidence for the claim is insufficient, since S2 as no data. -L512 "except COG3119 and COG0457 due to computational infeasibility. " please clarify. -L519. " We created dummy phylogenetic trees for the remaining 91,705 gene families with less than 4 members. " Clarify this point. Why was this done if there is no information ? Are there consequences?. -L553. The sentence is long and confusing.   ***************************************************** Reviewer #3 (Remarks to the Author):  The Article of Dharamshi et al. ‘Gene gain facilitated the origin and diversification of an ancient phylum of symbionts’ deals with the evolution of the bacterial phylum Chlamydia, mostly by applying computational methods such as large-scale phylogenomic analysis. Being not a bioinformation, I will focus on microbiological aspects of this work and do not touch technical aspects.  The subject of research, the phylum Chlamydia, is a very important branch of the so-called PVC-superphylm that consists besides Chlamydia of Planctomycetes, Verrucomicrobia and others. All these phyla are of particular interest given their environmental role including Chlamydia, that are best known as human pathogens. Thus, the topic of the work is highly relevant to a broad audience and absolutely fits to the journal scope.  The main and unexpected finding of the authors is that horizontal gene transfer (HGT) and genome expansion played an important role in Chlamydia evolution. This contradicts current textbook knowledge that during evolution, pathogens tend to lose more genes to streamline their genome as they face only constant host conditions. In particular, the authors demonstrate that during the evolution of Amoebachlamydiales gene gains led to a genome expansion. The ancestor of this linages comprises 1,783 genes compared with the last common ancestors of Chlamydia group 1 (1,285 genes) and group 2 (1,469 genes). Parachlamydiaceae even comprise more than 3,200 genes in their genome, almost double the amount of the ancestor of Chlamydia group 1 (1,285 genes). This finding is very surprising and interesting. The authors could find a good explanation in terms of adaptation to different oxygen conditions and subsequent metabolic adaptations. Their findings challenge the paradigm of long-term genome reduction during evolution in obligate endosymbionts. In contrast, endosymbiont genome complexity can be maintained or increased over evolutionary time scales. This is surprising as one would assume that endosymbionts are based on their lifestyle somehow limited in gene exchange. However, co-infection of a eukaryotic host with gene donors might explain this finding. Congratulations to the authors: once more PVC bacteria require textbooks to be rewritten.  The only criticism, if any, is some wording issues that readers unfamiliar with the topic might find difficult to understand. For example, in the abstract the authors write in L4-5 of unknown environmental roles. This is per se not wrong as the term ‘environmental Chlamydia’ which is frequently used but might be misleading for some readers. The authors nicely demonstrated that the last Chlamydial common ancestor was already adapted to an intracellular life-style. Truly environment includes intracellular life as well, but this might be misunderstood by readers, assume that some Chlamydia are free living. This potential confusion is further supported by sentences such as (P3L68-69) ‘…and have unknown lifestyles’. Sure, the lifestyle could not be observed with the methods applied, but in the light of the lex parsimoniae they are very-likely intracellular symbionts. It is not a requirement, but I suggest that the authors reword the manuscript -and maybe figure legend 1- to prevent such misunderstanding.  Minor thing:  The finding of peptidoglycan in all analyzed strains is just briefly mentioned in figure legend 2. I suggest adding 2-3 sentences to the manuscript on the PG issue in PVC bacteria as the authors’ finding is an important aspect in this regard. P12L245-258: I agree that the biphasic lifestyle might connect Chlamydia and Planctomycetes. However, the deep-branching Planctomycetes such as anammox species seem to lack such a biphasic life-style, that might have emerged only later in the ‘higher’ Planctomycetes. Maybe adding half a sentence mention this could present an alternative -although less likely- explanation.  Recommendation: accept for publication. Suggested changes need not to be made if authors disagree.  Many congrats for this great work! [redacted]   ***************************************************** Reviewer #4 (Remarks to the Author):  The overarching story of the manuscript revolves around symbiosis, but there was not much discussion on Chlamydiae and their hosts in nature. For example, what do the symbionts typically do for their hosts? I assume they are not all pathogenic as there is mention of Chlamydiae pathogens and symbionts, so are there any benefits that the symbionts confer? Or is “symbionts” being used here as microbes that have a long-term association with hosts, regardless of whether they are harmful or beneficial—it was not clear in the manuscript.  Moreover, the introduction is lacking any grounding in symbiosis literature in general. The first two sentences of the introduction and the last sentence in the manuscript (discussion) make sweeping statements without any regard for the general symbiosis literature or other systems. This risks the findings being specialist to the system and not of general interest. Really throughout the manuscript, there should be more reference to symbiosis literature.   It is a neat finding that Chlamydiae underwent genome reduction and expansion. The intracellular symbiont literature has many examples of metabolic complementarity between host and bacteria as a result of genome reduction, and these are usually found in invertebrates, mainly insects. Are all of the vertebrate-associated Chlamydiae pathogenic (Figure 1)? If not, what might this mean for the generality of symbiont genome reduction across host types? Is there evidence of metabolic complementarity (this relates back to the previous question on the role of the symbiont)?   Was the last common ancestor already host-associated? Or free-living with the potential to be host-associated? If the latter, it might suggest that some bacteria are more prone to become host-associated (if they already have genes that facilitate an endosymbiotic lifestyle as a free-living microbe), which has implications for the evolution of symbiosis in general.  Specific comments:  Line 19. Cite Drew et al. 2021. Nat Rev Microbiol. Line 180. How likely is extinction? Figure 5. More thorough description of results needed. This figure is complication but very illuminating. Line 334. References needed on that last sentence. What theoretical papers discuss genome reduction or is there evidence from other systems of genome reduction? Line 342. Horizontal transmission and HGT are increasingly being recongised as key to the maintenance of other inherited endosymbionts (e.g., Wolbachia, Arsenophonus). Can the authors discuss their findings in light of findings in other symbioses? Line 346. How would coinfection occur? Is it common? Do these organisms occupy the same host niche? Line 374. References needed. |  |

**Author Rebuttal to Initial comments**

# Response to Reviews

Please find enclosed our response to reviewer comments on our manuscript entitled “*Gene gain facilitated the origin and diversification of an ancient phylum of symbionts*”. We would like to begin by thanking the reviewers for taking the time to review our manuscript and for their constructive comments. We have largely implemented reviewer suggestions and used their feedback to further improve the manuscript. In particular, the reviewers identified two overarching points: **1.** Whether our analyses accurately reconstruct ancient patterns in genome evolution dynamics and can thus be used to infer ancestral proteome sizes and gene transfer rates. **2.** How our results showing that the chlamydiae ancestor was capable of an endosymbiotic lifestyle relate to previous knowledge and fit into the larger symbiosis literature. We have addressed these as follows:

1. Relative to other available methods, gene-tree aware ancestral state reconstruction (ASR) can more reliably infer ancestral proteome sizes and transfer rates, and it has previously been applied to study these specific questions (*e.g.*, Szöllősi *et al.* 2015, Philos. Trans. R. Soc.; Williams *et al.* 2017, PNAS; Sheridan *et al.* 2020, Nature Commun; Coleman *et al.* 2021, Science). Nevertheless, we recognize that there are biases that can be further addressed. In response to the reviewer concerns we have taken the following steps (see **R2.2**-**R2.5**):
   - Previously, we had only included events inferred with high-confidence, and we continue to do so for reconstructing the presence of specific genes across ancestors. However, for inferring genome dynamics we have instead switched to using total event frequencies, which include lower confidence events and are thus less conservative towards more ancient events. This resulted in more losses and transfers being inferred in ancient branches (Figure 3 and Extended Data Figure 7), but there were no differences in inferred ancestor proteome size patterns.
   - Reconstructed proteome sizes had been corrected for using gene extinction probability rates in our original analysis. We had not made this clear in the manuscript and have now added this information.
   - We now include a comparison between reconstructed proteome sizes of chlamydial family ancestors and extant members using both gene-tree aware (Figure 5b) and gene-tree unaware (Figure S12b) methods. In the latter approach, ancestral proteome sizes are indeed underestimated relative to extant members (Figure S12b), as was the concern of reviewer 2. Importantly, this is not the case with the gene-tree aware approach that we have used in our analysis (Figure 5b). Regardless, in both cases a relative increase in Amoebachlamydiales ancestor proteome size is clearly observed.
   - Extant Amoebachlamydiales have greater metabolic diversity and larger proteome sizes than other chlamydiae (Omsland *et al.*, 2014). It was previously thought that LCCA would have resembled modern Amoebachlamydiales and that other chlamydial groups had instead undergone genome reduction. This was based on our presumption that endosymbionts primarily evolve through gene loss and metabolic streamlining. Instead, we found that a large suite of metabolic genes was gained during Amoebachlamydiales evolution, causing this group to increase in metabolic complexity relative to LCCA and counter to our expectations for endosymbionts. This fact remains regardless of whether

ancestor proteome size is considered. We have taken steps to better emphasize this important piece of corroborating evidence throughout the manuscript.

2. We agree that a discussion of our work within a larger symbiosis context was not part of the manuscript. In addition, we had not clearly outlined our expected results, and how the reconstruction of LCCA and Chlamydiae genome evolution differed from this. We have now taken the following steps to accommodate this (see **R2.10** and **R4.1**-**R4.2** for further details):
   - We now explicitly outline prior knowledge on the nature of the chlamydial ancestor and better state how our expectations for Chlamydiae ASR differ from results. Since endosymbiosis-related genes are widespread in extant chlamydiae it was expected that LCCA was inferred to be an endosymbiont as well, equipped with a somewhat similar gene repertoire as extant Amoebachlamydiales. However, importantly, no large-scale reconstruction of Chlamydiae genome evolution had previously been done to confirm this, and the genetic makeup of LCCA was unknown. Furthermore, we found several unexpected yet important findings: some genes involved in the chlamydial lifestyle were already gained prior to the LVCCA; genes for endosymbiosis were gained prior to LCCA alongside a reduction of pathways involved in *de novo* biosynthesis, together suggesting obligate host-association; and LCCA was reconstructed as a motile facultative anaerobe but without the extensive aerobic metabolism found in modern Amoebachlamydiales. We have adjusted the text to improve the clarity of these points.
   - While keeping in mind space limitations, we have made efforts to better place our results within the wider symbiosis and chlamydial literature by adding 27 additional references. We have now defined symbiosis in our context, outlined knowledge on chlamydial host range and effects, and included additional information on prior knowledge about HGT and genome evolution trajectories in endosymbionts. With these additions, we think our findings are now better placed within the larger context of microbial symbiosis.

Thanks to changes implemented based on reviewer comments we feel that the strength of the manuscript has further improved. We appreciate the efforts of the reviewers in helping us see where additional clarity was warranted. Please find below a detailed response (in purple font) to each individual comment raised by the reviewers (in black font). Line numbers are indicated where corresponding sections of the text have been altered. We have labeled changes in the accompanying manuscript in colours corresponding to each reviewers comments as follows: Reviewer #2 in green, Reviewer #3 in orange, and Reviewer #4 in blue.

# Detailed response to reviewer comments (in purple)

*******************************

# Reviewer #1

**R1.1:** Remarks to the Author

Having now read the full manuscript, I feel that I am probably not a suitable reviewer. The manuscript is highly dependent on understanding and validating the complex phylogenomics methods that have been used. I am not able to do this as I am not sufficiently expert in the area of advanced bacterial phylogenomics. The authors have done an excellent job of interpreting and extending the evolutionary hypotheses. However, without having confidence in the methodologies used, any interpretation as not valid.

The question addressed in this manuscript though is very relevant and intriguing. Chlamydia is an extremely interesting and important group of organisms with a unique adaptation to intracellular symbiosis. The topic of the manuscript therefore is extremely novel and interesting and will be of broad interest.

We would like to thank the reviewer for the positive assessment of the key questions and topics addressed in the manuscript.

*******************************

# Reviewer #2 (in green)

**R2.1:** This manuscript describes an extensive analysis of genomes of Chlamydiae (and other in the broader PVC clade) in view of understanding the evolution of gene repertoires from a presumably free-living ancestor to an obligatory intracellular pathogen. This clade contains bacteria that are obligate pathogens and others that are environmental. It has spurred significant interest because it provides an interesting group to understand the evolution of intracellular pathogenesis using mechanisms that have probably evolved to protect (or infect) protozoa. Here, the authors use phylogenetic inference and reconstruction of ancestral states to track the evolution of the genes in the clade (in terms of presence/acquisition). The authors draw three major conclusions. 1) That the process of evolution was characterized not just by extensive loss, but also by genome expansion by horizontal transfer. 2) that the ancestor gained the ability to infect eukaryotic hosts over a billion years ago. 3) That this corresponded to transitions between types of energy metabolism.

Some of the results associated with claim #1 may be the result of a reconstruction artifact. The claim #2 does not seem very novel relative to previous works of the authors (confirmatory). The extensive and careful analyses behind claim #3 are interesting and important for the researchers in the field. The paper is well written. I have one very strong criticism, a few major suggestions and several minor ones. Most of these concern claim #1.

We want to thank the reviewer for emphasizing that our findings are important for researchers in the field and for their feedback that the paper is well written. In terms of concerns regarding our

claims #1 and #2, we have worked the manuscript to clarify and address the present issues and ambiguities in the text. With the help of the reviewer’s feedback, we feel we have improved the clarity of the manuscript. As with any study aiming to infer deep evolutionary events, our work also potentially includes biases affecting our results and the interpretation thereof; we would like to thank the reviewer for pointing them out. We originally had already made efforts to account for these biases, but have now taken additional steps to further address these concerns, to better emphasize corroborating evidence, and to clearly state the methods that were used to address them. Please see below for more specific details in response to each point raised.

# Major problem:

**R2.2:** The reconstruction of ancestral states was done using state-of-the-art tools. But the authors seem unaware that this type of analyses allows to infer the presence of genes in the ancestor, but not the size of genomes (in terms of the number of genes). This results in an artifact that challenges several major results supporting claim #1. The major problem is that if one takes extant genomes and one reconstructs the gene set of ancestors one always gets a biased downwards estimate of the number of genes. This means that the size of the gene set is smaller than it should be. This affects the conclusions drawn from figs 3 and 5. The reason is well known, although possibly not sufficiently advertised: because some proteins evolve fast and especially because some protein families are prone to a lot of transfer and loss, they are not inferred to be present back in the tree. Let's take an example: many species have many transposable elements. Each family of these elements is usually present in a few strains of a species. But most genomes have at least one family. The inference of ancestral states in such cases is that all these elements are absent from the ancestors. This is not just a problem of accuracy, there is a systematic bias that is true for any method (ML, Bayesian, etc).

While it is certainly true that any ASR method is in principle subjected to systematic biases, gene- tree aware approaches have been shown to better account for ancient events, and the specific method we used has previously been applied to specifically study this question (*e.g.*, Szöllősi et al. 2015, Sheridan et al. 2020). However, there are indeed caveats to inferring ancestral gene content, issues that we are aware of and that we have also outlined in our manuscript (lines 143- 145). In addition, we have now added a statement further outlining this and that our main finding stems from the larger patterns in gene content evolution rather than the exact number of genes in ancestral genomes:

[lines 408-413] - “Although we cannot accurately reconstruct the evolution of all genes, such as those that have since gone extinct or are fast-evolving, by examining relative reconstructed proteome sizes (*i.e.*, protein-coding gene copy number) we can investigate larger patterns. Using multiple methods, our reconstructions of gene content indicate a shift towards larger proteome sizes leading to the LCWPA (Figures 5a and S12a).”

We have also taken steps to better account for potentially biased downwards estimates of the number of protein-coding genes by including events inferred with lower confidence when estimating genome evolution dynamics (see R2.3 for more details). Additionally, we included a

comparative analysis showing that the gene-tree aware ASR approach did not not result in biased downwards estimates of proteome sizes when comparing ancestors and extant members within chlamydial families (Figure 5b), while a gene-tree unaware approach did as was the concern of the reviewer (Figure S12b; see R2.5 for more details). In the manuscript text, we have also now explicitly indicated that gene extinction rates were taken into account when inferring ancestor proteome sizes. We better outlined the corroborating evidence that gene gain through HGT led to the larger proteome sizes seen in extant Amoebachlamydiales, and we placed greater emphasis on relative patterns and the increase in Amoebachlamydiales metabolic complexity relative to LCCA (see R2.5 and R2.6 for more details).

**R2.3:** The problem of the bias in inferring gains and losses is clear in Fig 3. For a clade that is supposed to have endured genome reduction it's odd that all but one branch reveal more gains than losses. This is a problem, because this bias may explain some of the claims that the clade has had a more complex evolution of gene repertoires than expected.

We initially only used high-confidence events (over 0.3 reconciliation frequency and rounded up to 1) across all analyses in the manuscript for consistency, and since this gene set represented what was used for reconstructing and inferring the metabolic capabilities of the ancestors. We had tested and specifically chosen this reconciliation cut-off (see Figure S8-S9 for details), and it has previously been used in other studies as well (Martijn *et al.* 2020, Huang *et al.* 2021). However, doing so does result in potential underestimation of transfers and losses, due to many lower likelihood events (Figure S8), especially in early ancestors. We have thus switched to reporting results based on overall event frequencies in the text and figures when discussing larger patterns in gene content evolution (*e.g.*, in Figure 3 and 5, and Extended Data Figure 7). We have also added this information to Data S7. However, we have retained the use of only high- confidence events with the more conservative reconciliation cut-off of 0.3 for all cases involving ancestral gene content reconstruction (*e.g.*, in Figure 2 and Extended Data Figure 2). By including lower likelihood events in the reconstruction, more losses and transfers are now inferred in ancient nodes (Figure 3) than previously (Figure S11), while estimated proteome sizes have remained stable. Thus, including these events has allowed us to more accurately reconstruct more ancient genome evolution dynamics. We have adjusted the text to reflect these additional methods as follows

[lines 140-142] - “Frequencies of inferred events were then used to assess overall patterns in gene content evolution and took into account estimated gene extinction rates. However, only genes inferred as present with high confidence were used to reconstruct ancestral gene content.”

[lines 253-256] - “Figure 3 Raw gene copy and event numbers with reconciliation frequencies

were summed up per ancestral node depicted. Terminal nodes represent chlamydial family ancestors, collapsed into triangles, with the corresponding node events shown to the right. Orphan lineages are excluded from the schematic. See Figure S11 for gene copy and event numbers based on reconciliation frequencies using a frequency cutoff of 0.3 (see Methods) ”

[lines 733-735! # $%&' &()&'*+ ,-./'0 12 31,*',*-* )',' 24.(5('* 1/+4(,'6 7(+& )','#+0'' 4740' 4,6 -,4740' .'+&16* 74* 0'43&'6 7(+& 4 +&0'*&156 12 89: ;<()-0' =>4?9 @4*'6 1, +&(* 4,45A*(*

4,6 +&' 'B',+ 6',*(+A 6(*+0(/-+(1,*C 7' *'5'3+'6 4 20'D-',3A 3-+122 12 89: 210 (,2'00(,) 'B15-+(1,40A

'B',+* (, 1-0 E=F 4,45A*(* ;<()-0'* =G#=>?9 H',' 24.(5('* 7'0' +&-* (,2'00'6 4* I0'*',+ 7&',

0'31,*+0-3+(,) 4,3'*+10 )',' 31,+',+ (2 +&'A &46 4 31IA 20'D-',3A 12 4+ 5'4*+ 89:9 J, 466(+(1,C 7'

-*'6 +&(* 3-+122 +1 3453-54+' 31,2(6',+ 'B',+ 20'D-',3('*9 %&(* .'4,+ +&4+ 4, 'B',+ 20'D-',3A K

89: 4,6 L M9: 74* 31-,+'6 4* MC 4 20'D-',3A K M9: 4,6 L N9: 74* 31-,+'6 4* NC 4,6 *1 1,9 %&'*'

31,2(6',+ 'B',+ 20'D-',3('* 3100'*I1,6 +1 +&' )',' 31,+',+ 4,6 'B',+* -*'6 (, 1-0 4,3'*+045

0'31,*+0-3+(1,* ;<()-0'* NC OC 4,6 =M8#=MNC PQ+',6'6 R4+4 <()-0' N 4,6 S?9 T17'B'0C 210 '*+(.4+(,) )',1.' 31,+',+ 'B15-+(1, 6A,4.(3* 4,6 4,3'*+045 I01+'1.' *(U'C 047 0'31,3(5(4+(1,

20'D-',3('* 7'0' -*'6 +1 4B1(6 4 I1+',+(45 -,6'0'*+(.4+(1, 12 +04,*2'0* 4,6 51**'* ;(9'9C <()-0'*

: 4,6 VC PQ+',6'6 R4+4 <()-0'* V#W?9X

**R2.4:** This is of course also a huge problem for the inference of the rates of horizontal transfer, because it means that the transfer of the most transferred families of genes is heavily under- estimated. The resulting rates of transfer are very conservative and, more problematic, they are downwards biased and more so for ancient events: the more one goes back in time the most likely it is that they are lower than the real value.

We agree with the author that overall transfer rates could be underestimated due to fast evolving proteins like for example, as mentioned by the reviewer, transposases that might not be recovered within one gene family due to their fast divergence. We tried to minimize such bias by using the evolutionary broad eggNOG gene families at the bacterial level and supplementing with a *de novo* clustering for unmapped proteins that was calibrated to retain proteins with the same TIGRFAM assignment in the same gene family (see Methods and Figure S7). However, we focused our analysis on the comparison of transfer rates between ancestors of extant chlamydial families that are sufficiently sampled (Extended Data Figure 7c). In this analysis, a potential underestimation of (ancient) transfer events should equally affect all groups compared. It should thus only have a minor influence on our general conclusions regarding the marked differences in gene transfer rates between extant chlamydial families.

**R2.5:** This has a deeper and even more problematic effect when the authors take the inferred sets of ancestral genes as a proxy of the size of gene repertoires in the ancestors. This is used to make assumptions about reductive evolution, etc. The problem is that, once again, the more one goes back in time the worst is this proxy. And the more biased it is (the effect is that genomes sizes get more and more underestimated even if genome sizes were unchanged). If one takes the example above, the ancestral genomes will lack most transposable elements and therefore will be inferred as being smaller than they actually were. Fortunately, in the specific case of

genome size there is a way of estimating how much is lost in the analysis. The authors should take the tree and the values of genome size (or the number of protein coding genes) and infer ancestral sizes in internal nodes in the tree. Then they should compute the difference between the number of genes inferred in their analysis and the number of genes in the genome inferred by this new analysis. This will reveal and quantify the bias.

The major concerns of the reviewer about accurately inferring ancestor proteome size relate to: inferring fast-evolving and extinct genes in ancestors and biased downward estimates of ancestor gene repertoires further back in time. We agree that this bias could be an issue and have now taken steps to outline how we have addressed it as suggested by the reviewer. We have also directly pointed out this potential bias in the text:

[lines 408-411] - “Although we cannot accurately reconstruct the evolution of all genes, such as those that have since gone extinct or are fast-evolving, by examining relative reconstructed proteome sizes (*i.e.*, the number of protein-coding genes) we can investigate larger patterns.”

The ancestral proteome size estimates reported in our manuscript were in fact based on inferring ancestral sizes at internal nodes in the tree using the gene-tree aware ASR approach. However, we have now included two comparisons to help reveal and quantify potential bias in our analysis. In Figure 5a, we now only show deeper chlamydial ancestor proteome sizes scaled to relative evolutionary divergence. We have now added panel b comparing proteome sizes inferred for ancestral nodes and extant members within each chlamydial family. We found that inferred ancestral proteome sizes are within a similar range to extant members, indicating that we are not severely underestimating ancestral proteome sizes. In the legend of Figure 5 we state this as follows:

[lines 428-437] - “Figure 5. The proteome size of Amoebachlamydiales ancestors increased relative to other chlamydiae. Proteome size (i.e., the sum of inferred gene copies corrected for by gene extinction probability; see Methods; Data S7) inferred across chlamydial ancestors using gene-tree species-tree reconciliation, and protein-coding gene copies present in extant chlamydial genomes. a. Inferred proteome size of early chlamydial ancestors scaled to the relative evolutionary divergence (RED) metric. The RED metric provides an approximation of relative time from a given common ancestor (LPVCCA, RED=0) to extant taxa (RED=1). b. Comparison of proteome size between ancestors and extant members from each chlamydial family. Chlamydiae from families without in-family ancestors are grouped together in the grey boxplot (i.e., Criblamydiaceae, Waddliaceae, Chlamydiae Clade IV, and unclassified). Nodes and branches are coloured by family assignment according to the legend. Abbreviations of key ancestors are labeled as in Figure 3 (see Data S7).”

In addition, we have completed the same analysis using the gene-tree unaware method implemented in Count (new Figure S12). In contrast to the gene-tree aware approach used in our work, here we do see a clear underestimation of proteome size inferred for ancestors from the same family as was the reviewers concern and have added this information to the text:

[lines 413-415] - “By using gene-tree aware ancestral reconstruction, which are able to estimate and take into account gene extinction rates, we can more accurately capture ancient events.”

[lines 421-424] - “Within each chlamydial family, ancestors were reconstructed as having proteome sizes that resemble extant members (Figure 5b). However, in a reconstruction using only gene presence and absence, ancestor proteome sizes were underestimated relative to extant members within each chlamydial family (Figure S12b).”

In addition, our results demonstrate that using an independent (tree-unaware) method, even when clearly underestimating ancestral proteome sizes, the same pattern of a shift towards larger proteome sizes among Amoebachlamydiales is evident (Figures 5a and S12a). We thus added the following statement:

[lines 411-413] - “Using multiple methods, our reconstructions of gene content indicate a shift towards larger proteome sizes leading to the LCWPA (Figures 5a and S12a).”

While it is true that we cannot infer the true number of genes in an ancestor, we specifically employed methods that account for lineage-specific gene extinction. We have now clarified in the methods that proteome size (or gene copy number) was corrected for using extinction probabilities in ALE. We would also like to point out that there are limits to how much ancestral genome sizes can be underestimated.

[lines 716-718] - “In addition, the specific implementation of ALE corrects ancestor gene copy number estimates using modeled gene extinction probability rates (Szöllősi et al., 2013), as has previously been employed to estimate ancestral proteome sizes (Williams et al., 2017).”

Importantly, in our context when discussing “genome expansion” we are not only referring to changes in gene content (protein-coding) size, but also to a large number of HGTs that led to increased metabolic complexity in Amoebachlamydiales since LCCA. It was originally presumed, including by authors here, that the metabolic versatility characteristic of Amoebachlamydiales must have been present in LCCA, since this follows thinking on endosymbiosis leading to a trajectory of reductive genome evolution. Instead, we found clear evidence that these metabolic genes were gained later through extensive HGT. Here, we put a particular emphasis on the evolution of energy metabolism, work which Reviewer 2 summarized as “extensive and careful analyses”. We have now placed greater focus on how Amoebachlamydiales evolved through an increase in metabolic versatility through gene gain counter to expectations, and that this is what led to the larger proteome size in this specific group. This is now outlined in the text as follows:

[lines 12-15] - “Unexpectedly for strict endosymbionts, our reconstruction revealed extensive gene gain through horizontal gene transfer during Chlamydiae evolution. In particular, we found that protist-infecting chlamydiae gained characteristic metabolic and aerobic genes, such as electron transport chain complexes, after divergence from the Chlamydiae ancestor.”

[lines 462-463] - “This stands in large contrast to our findings that extensive gene gain led to the larger proteome sizes found today in the Amoebachlamydiales”

[lines 52-55] - “It was also expected that the chlamydial ancestor would have most closely resembled extant protist-infecting lineages, which have greater coding potential and metabolic versatility, with other chlamydiae having lost these genes through genome reduction processes.”

[lines 403-408] - “The gain of aerobiosis-related genes in the Amoebachlamydiales was accompanied by other prominent gene gains related to metabolic potential (Extended Data Figure 3), in line with the extended metabolic capabilities and larger gene repertoires of extant members relative to other chlamydiae. Unexpectedly, our results provide evidence that many of these key genes were not present in the LCCA but were instead gained through HGT during Amoebachlamydiales evolution (Extended Data Figure 4) leading to their characteristic metabolic complexity. ”

[lines 415-421] - “The Amoebachlamydiales ancestor was reconstructed with 1,691 protein- coding genes, relative to 1,225 and 1,408 genes reconstructed in LG1CA and LG2CA, respectively (Figure 3). Further large gene influxes through HGT led to increased metabolic complexity and gene content expansion in Amoebachlamydiales (Figures 5a and Extended Data Figure 4). The last common ancestor of the Criblamydiaceae, Waddliaceae, and Parachlamydiaceae (LCWPCA) was reconstructed with a proteome size of 2,560 genes as having doubled gene content since LG2CA (Figure 3).”

[lines 71-72] - “Counter to our expectations for genome streamlining during endosymbiont evolution, HGT led to expanded metabolic potential in protist-infecting chlamydial groups.”

[lines 538-540] - “Our results demonstrate that protist-infecting chlamydiae have increased in metabolic complexity through rampant HGT during their evolution, counter to expectations for obligate endosymbionts.”

[lines 471-475] - “However, genome expansion had not previously been demonstrated in obligate endosymbionts. Unexpectedly, our results demonstrate that extensive gene gain in Amoebachlamydiales lead to the larger proteome sizes and increased metabolic complexity in extant members of this group (Figure 5). This finding suggests that obligate endosymbionts can evolve counter to genome reduction processes throughout evolutionary time.”

# Other major issues:

**R2.6:** - The problems mentioned above in terms of identification of horizontal gene transfer are amplified by the use of MAGs, which are well known to miss many mobile elements. This makes the analysis of horizontal transfer even less accurate.

We accounted for the incompleteness of metagenome assembled genomes (MAGs) (with only high-quality MAGs of >90% completeness included) within ALE’s probabilistic approach using the genome completeness estimates. We have now better outlined these points as follows:

[lines 699-702! # $%1 )4(, 4 .10' 31.I5'+' I'0*I'3+(B' 1, YZ[ )',1.' 'B15-+(1,C 7' -*'6 +&'

31.I5'+' )',1.' 64+4*'+ 1-+5(,'6 4/1B'C 7&(3& (,35-6'* \EH* 201. -,3-5+-0'6 5(,'4)'* +&4+

71-56 1+&'07(*' /' .(**'69 T17'B'0C +&(* 74* 0'*+0(3+'6 +1 &()&#D-45(+A \EH* 4* 1-+5(,'6 4/1B'

;31.I5'+','** K >8] 4,6 31,+4.(,4+(1, ^ N]?9X

**R2.7:** - Several clades were removed from the analysis because they had extreme genomes or were hard to place. Reconstruction of ancestral states is very sensitive to non-random sampling, especially when clades have heterogeneous distributions of traits, as it is the case in this study. I would have liked to know how the most important results are affected by the inclusion of these clades. I realize that some may produce instabilities in the tree, but the use of Bayesian methods allows to account for that. It is important to understand the effect of these decisions on the major results.

As outlined in the methods, the sampling of PVC bacteria genomes included only those with high quality and was performed randomly with a representative of each species included for Chlamydiae and a representative of each genus for other PVC bacteria (Figure S1). We only subsequently removed four MAGs belonging to two clades as a result of phylogenomic reconstructions that revealed they could not be reliably placed within the chlamydial species tree,

*i.e.* the three Parilichlamydiaceae MAGs and another chlamydiae MAG, Chlamydiae bacterium 1070360-7.

In general we tried to include as much high-quality chlamydial genome information as possible, which is why we included MAGs of high quality in addition to complete closed genomes. As described in the manuscript, we initially used Bayesian phylogenetic methods to obtain a stable phylogenomic tree, yet we could not reliably place these chlamydial lineages as multiple potential positions were reconstructed in chlamydial species trees, none of which were sufficiently supported. Our extensive effort in trying to resolve this is summarized in Figures S3-S4, S6, and Supplementary Discussion 1. However, due to the large computational resource demands of the analyses we performed, it was not feasible to investigate all different scenarios, and doing so would introduce unnecessary complexity to the results. As Parilichlamydiaceae and 1070360-7 are orphan lineages on long branches, the various positions we observed are likely artifacts introduced by biases such as long-branch attraction and heterogenous amino acid composition. In addition, the Parilichlamydiaceae in particular have divergent genome composition and consistently have long-branches in individual gene trees. As our aim was to infer a high-quality and reliable reconstruction of chlamydiae evolution with current taxon sampling we decided to exclude these lineages due to the likely biases and lack of resolution they would introduce in both the species and gene trees and thus subsequent reconstruction. We added the following line to the manuscript to clarify this:

[lines 92-95] - “However, four genomes belonging to two long-branching chlamydial lineages were removed due to unstable positions across species phylogenies and divergent genome compositions that could result in reconstruction artifacts (Figures S3-S4; Supplementary Discussion 1).”

**R2.8:** -The use of gene species-species trees reconstructions to infer the rates of transfer has recently been criticized as producing biased estimates and this should be commented (Tria, GBE, 21).

We now calculated the per genome t/d ratio we reconstructed with ALE for our study and obtained an average value of **9.25**. When keeping in mind that in our study, we dereplicated at the species and genus level for Chlamydiae and non-chlamydial PVC genomes, respectively, we retrieve a mean t/d value that is comparable to what Tria and Martin display in Figure 2 for inter-genus comparisons with >=2 genomes per group. Furthermore, given that the focus of our study is obligate endosymbionts, we also expect a lower t/d ratio in the first place due to the lower exposure to ‘foreign’ DNA in an intracellular niche. Finally, we would like to point out that while Tria and Martin criticize reconciliation methods, they actually do not show that their own approach performs better. We feel that performing a detailed comparison between both methods is beyond the scope of the current study.

# Minor issues:

**R2.9:** -The inaccuracy of using MAGs is amplified by the fact that they are not randomly dispersed in the tree, but instead they cluster in poorly known clades. I do realize that this is necessary, MAGs are used exactly because they are in poorly sampled parts of the tree. But a note of caution is needed.

As pointed out by the reviewer, indeed MAGs tend to be incomplete and can include contaminating sequences. To minimize the issues that arise with missing or contaminant “alien” gene content we applied strict quality standards for our selection criteria. Specifically, MAGs had to have an estimated completeness of at least 90% and a maximum redundancy of 2%. Furthermore, the estimated fraction of missing gene content for all genomes were supplied to ALE, which uses these values to correct for potentially missing data in the reconstruction. To better inform the reader of this potential caveat and to clarify the methods we have employed to account for it, we added the following to the text:

[lines 714-718] - “Furthermore, the estimated fraction of missing gene content per genome was provided to ALE as it uses this to correct for potentially missing data, i.e. in the MAGs included. In addition, the specific implementation of ALE corrects ancestor gene copy number estimates using modeled gene extinction probability rates (Szöllősi et al., 2013), as has previously been employed to estimate ancestral proteome sizes (Williams et al., 2017).”

**R2.10:** - L52. It seems to me that the claim " the Chlamydiae ancestor had already obtained key genomic features associated with a symbiotic lifestyle" has been done several times for other traits by the same group. Maybe I missed the point. If not, this should be specified and the claim in the abstract could be toned down.

The notion that the chlamydial ancestor already had features of a symbiotic lifestyle was so far based on comparative genomics of a limited number of chlamydial genomes and phylogenetic analysis of key genes associated with this lifestyle. However, importantly, no large-scale reconstruction of Chlamydiae genome evolution had previously been done to support this. Furthermore, we found several unexpected key findings. First, some genes involved in the chlamydial lifestyle were already gained prior to the LVCCA. Second, we found that genes for endosymbiosis were gained prior to LCCA alongside a reduction of pathways involved in *de novo* biosynthesis, which together suggest obligate host-association. Third, we reconstructed LCCA as a motile facultative anaerobe but without the extensive aerobic metabolism found in modern Amoebachlamydiales. We have adjusted the text to improve the clarity of these points.

[lines 52-55] - “It was also expected that the chlamydial ancestor would have most closely resembled extant protist-infecting lineages, which have greater coding potential and metabolic versatility, with other chlamydiae having lost these genes through genome reduction processes.”

[lines 232-235] - “Earlier hypotheses for Chlamydiae genome evolution were predominantly based on gene presence patterns across extant taxa and included limited genetic diversity. Our results provide the previously missing support that LCCA already had the genetic toolkit for an endosymbiotic and biphasic lifecycle that has been maintained throughout chlamydial evolution.”

[lines 8-10] - “We found that endosymbiotic genes were gained prior to the Chlamydiae ancestor, providing further evidence that endosymbiosis has prevailed throughout chlamydial evolution.”

[lines 235-238] - “We could further show that some genes involved in the chlamydial lifestyle were already gained prior to the LVCCA and that LCCA evolved through both the gain of additional genes for endosymbiosis and a reduction of pathways involved in *de novo* biosynthesis.”

[lines 333-335] - “Together, we propose that LCCA was a motile facultative anaerobe. Contrary to our expectations and previous hypotheses, the extensive aerobic and energy metabolism found in modern Amoebachlamydiales was not reconstructed in LCCA.”

**R2.11:** -It is not always clear to me why the manuscript has so many different ways of doing seemingly similar analyses. I would understand that the CAT model can't be used in very single case, but some analyses are done with complex ML methods, whereas others are done using very simplified (and not very accurate) methods. For example, fig S1.

As the reviewer has pointed out, we used different phylogenetic methods for reconstructing phylogenomic trees. In general we used ML with complex models alone for estimating the general robustness of species tree topologies with the different genome datasets and the effect of removal

of heterogeneous sites presented in Figures S3-5, and both ML and Bayesian methods for the final species tree in Figure 1 (keep in mind that Bayesian analyses of these large datasets are computationally extremely demanding). The species tree in Figure S1 was indeed reconstructed using simplified methods (*i.e.* FastTree) but is purely meant for representation purposes. The underlying analysis for genus-level selection of representative genomes was performed based on GTDB taxonomic ranks and the depicted tree had no effect on this.

**R2.12:** Also, sometimes trimming is done with BMGE and in others with chi2 trimming (none of which is among the best in the benchmarks).

We always filtered poorly aligned sites from multiple sequence alignments by trimming with BMGE for all alignments related to the ancestral state reconstruction (i.e., marker gene trees for inferring the species trees and single gene trees of all protein families). In other cases we used trimAL (16S rRNA gene tree and single gene trees to identify HGT events for gene originations). We are not sure which benchmark the reviewer is referring to, but in a recent benchmark of trimming methods (PMID: 33264284) it was shown that BMGE with an increased entropy threshold (here we used 0.6 instead of the default 0.5) and trimAL ‘-gappyout’ perform well on single gene tree alignments of empirical datasets, while reducing the computational complexity. The benchmark further states that the effect of either trimming method compared to no trimming was negligible for concatenated alignments.

Chi2 trimming was specifically used to sequentially remove compositionally heterogeneous sites from the already trimmed and concatenated protein alignments used for the species trees.

**R2.13:** -L116. Here and elsewhere the use of LCCA is ambiguous. It sometimes refers to its gene repertoires, which is fine. But sometimes is used as the genes acquired by LCCA. I assume these are the genes acquired in the branch between this node and the one above in the tree and for which we have evidence in LCCA. The difference between past genomes and past lineages should be clarified.

Thanks for pointing this out. We have taken steps to address this ambiguity throughout the text and now use words such as “prior to” or “reconstructed in” to make it clear that the genes were acquired on the branch leading to the relevant ancestor, rather than by the ancestor itself (see adjustments in green in the main text).

**R2.14:** -L243. The indication of figure 2 as evidence for the claim is insufficient, since S2 as no data.

We thank the reviewer for pointing out this missing reference to supplementary file Data S9. We went through the manuscript and added references to the supporting supplementary data where missing.

**R2.15:** -L512 "except COG3119 and COG0457 due to computational infeasibility. " please clarify.

We have now clarified more specifically why these two gene families were excluded:

[lines 685-691] - “Two specific gene families were later excluded (COG3119 and COG0457) due to poor alignment (likely caused by repeat regions) and subsequent difficulties inferring a ML tree, bootstraps from which are required for ALE. These gene families primarily occur in non- Chlamydiae PVC members and thus should not impact chlamydial ancestor proteome sizes.”

**R2.16:** -L519. " We created dummy phylogenetic trees for the remaining 91,705 gene families with less than 4 members. " Clarify this point. Why was this done if there is no information ? Are there consequences?.

These dummy phylogenetic trees were made because ALE requires this format for all protein families with more than one member. It only concerned protein clusters with 2 or 3 sequences, which come mostly from closely related taxa (shallow originations) or from distantly related taxa (rather clear cases of transfers, though direction remains unknown). However, with only 2 or 3 sequences one cannot infer a tree topology since, as stated by the reviewer, there is no information. Thus, there are no consequences for the inference, other than allowing these protein families to be included in the ALE reconstruction.

We have clarified this point in the methods:

[lines 689-691] - “The remaining 91,705 gene families had three or fewer sequences. Thus, phylogenetic trees could not be inferred. However, ALE requires gene families in a tree format, and we thus provided mock trees for those with two or three sequences.”

**R2.17:** -L553. The sentence is long and confusing.

We agree and have adjusted the sentence accordingly:

[lines 733-735] - “The highest number of consensus gene families obtained with gene-tree aware and unaware methods was reached with a threshold of 0.3 (Figure S9a). Based on this analysis and the event density distributions, we selected a frequency cutoff of 0.3 for inferring evolutionary events in our ASR analysis (Figures S8-S9). Gene families were thus inferred as present when reconstructing ancestor gene content if they had a copy frequency of at least 0.3.”

*******************************

# Reviewer #3 (in orange)

**R3.1:** The Article of Dharamshi et al. ‘Gene gain facilitated the origin and diversification of an ancient phylum of symbionts’ deals with the evolution of the bacterial phylum Chlamydia, mostly by applying computational methods such as large-scale phylogenomic analysis. Being not a bioinformation, I will focus on microbiological aspects of this work and do not touch technical aspects.

The subject of research, the phylum Chlamydia, is a very important branch of the so-called PVC- superphylm that consists besides Chlamydia of Planctomycetes, Verrucomicrobia and others. All these phyla are of particular interest given their environmental role including Chlamydia, that are best known as human pathogens. Thus, the topic of the work is highly relevant to a broad audience and absolutely fits to the journal scope.

The main and unexpected finding of the authors is that horizontal gene transfer (HGT) and genome expansion played an important role in Chlamydia evolution. This contradicts current textbook knowledge that during evolution, pathogens tend to lose more genes to streamline their genome as they face only constant host conditions. In particular, the authors demonstrate that during the evolution of Amoebachlamydiales gene gains led to a genome expansion. The ancestor of this linages comprises 1,783 genes compared with the last common ancestors of Chlamydia group 1 (1,285 genes) and group 2 (1,469 genes). Parachlamydiaceae even comprise more than 3,200 genes in their genome, almost double the amount of the ancestor of Chlamydia group 1 (1,285 genes). This finding is very surprising and interesting. The authors could find a good explanation in terms of adaptation to different oxygen conditions and subsequent metabolic adaptations. Their findings challenge the paradigm of long-term genome

reduction during evolution in obligate endosymbionts. In contrast, endosymbiont genome complexity can be maintained or increased over evolutionary time scales. This is surprising as one would assume that endosymbionts are based on their lifestyle somehow limited in gene exchange. However, co-infection of a eukaryotic host with gene donors might explain this finding. Congratulations to the authors: once more PVC bacteria require textbooks to be rewritten.

We would like to thank Reviewer #3 for their nice overview of our work and positive assessment of its importance and key findings.

**R3.2:** The only criticism, if any, is some wording issues that readers unfamiliar with the topic might find difficult to understand. For example, in the abstract the authors write in L4-5 of unknown environmental roles. This is per se not wrong as the term ‘environmental Chlamydia’ which is frequently used but might be misleading for some readers.

We would like to thank Reviewer #3 for pointing out this potential point of confusion for some readers. We have removed occurrences of “environmental chlamydiae”. Likewise, we have re- worded several sentences that include the word “environmental” to clarify our meaning:

[lines 4-5] - “This group of highly successful eukaryotic endosymbionts ranges from animal pathogens to relatives with protist hosts from diverse environments.”

[lines 46-48] - “The protist-infecting Parachlamydiaceae and Chlamydiaceae animal pathogens have large differences in genome size and content, with larger genomes and metabolic capacity in the former.”

[lines 513-514] - “The resulting transfer network reveals highways of gene transfer indicating likely shared environmental niches, such as shared hosts (Extended Data Figure 7).”

[lines 57-59] - “The recent rise of culture-independent genomics has led to the retrieval of numerous uncultured chlamydial lineages from various environments and with unknown hosts.”

[lines 109-112] - “Members of G1 are primarily represented by MAGs sequenced from diverse environments and thus have unknown hosts, although several distinct groups are likely animal- associated as they were obtained from marine and terrestrial invertebrate metagenomes (Figure 1).”

**R3.3:** The authors nicely demonstrated that the last Chlamydial common ancestor was already adapted to an intracellular life-style. Truly environment includes intracellular life as well, but this might be misunderstood by readers, assume that some Chlamydia are free living. This potential confusion is further supported by sentences such as (P3L68-69) ‘…and have unknown lifestyles’. Sure, the lifestyle could not be observed with the methods applied, but in the light of the lex parsimoniae they are very-likely intracellular symbionts. It is not a requirement, but I suggest that the authors reword the manuscript -and maybe figure legend 1- to

prevent such misunderstanding.

We are thankful to Reviewer #3 for pointing out that some readers may misunderstand “environmental'' to equate with “free-living”. We have replaced instances of “unknown lifestyles” with “unknown hosts” to clarify this and adjusted Figure 1 (*i.e.*, changed lifestyle to host- association, changed unknown to unknown host) and the corresponding legend accordingly. In addition we have added a statement specifying that there are no known free-living chlamydiae to the introduction:

[lines 61-62] - “Despite this, there are no known free-living chlamydiae and those isolated are obligately host-associated.”

[lines 83-85] - “Although some of these lineages are known to be host-associated, the majority are represented by metagenome-assembled genomes (MAGs) that have unknown hosts and physiology (Figure 1).”

[lines 126-127] - “Genome type, evidence for host-association, and the environment from which the genome or MAG was obtained, is indicated by coloured squares for each chlamydiae representative according to the legend.”

Minor thing:

**R3.4:** The finding of peptidoglycan in all analyzed strains is just briefly mentioned in figure legend

2. I suggest adding 2-3 sentences to the manuscript on the PG issue in PVC bacteria as the authors’ finding is an important aspect in this regard.

We agree and have added a few sentences to the manuscript outlining our findings of peptidoglycan biosynthesis genes in Chlamydiae and PVC bacteria ancestors, with some context about the PVC peptidoglycan issue:

[lines 223-231] - “Key peptidoglycan biosynthesis and cell division genes (e.g., murACDEFGJ, mreB, mraY, ftsKI, but not ftsAZ) were also reconstructed in both LCCA and LVCCA (Figure 2, Extended Data Figure 2, and Data S10). While peptidoglycan is critical for cell shape and division in most bacteria, Chlamydiae and other PVC bacteria, such as Planctomycetes, were initially thought to be an exception and to lack this essential structural molecule (Fuerst, 2013). Peptidoglycan has now been detected in diverse Chlamydiae (Pilhofer et al., 2013; Liechti et al., 2014) and in Planctomycetes (Jeske, 2015), although peptidoglycan biosynthesis genes are absent in some Planctomycetes (Wiegand, 2020). Our results provide evidence that peptidoglycan biosynthesis genes were present across key Chlamydiae and PVC bacteria ancestors (Data S10).”

**R3.5:** P12L245-258: I agree that the biphasic lifestyle might connect Chlamydia and Planctomycetes. However, the deep-branching Planctomycetes such as anammox species seem to lack such a biphasic life-style, that might have emerged only later in the ‘higher’ Planctomycetes. Maybe adding half a sentence mention this could present an alternative - although less likely- explanation.

As suggested we have clarified this point by rewording the relevant sentences to include the alternate scenario:

[lines 337-340] - “Some Planctomycetes lineages also employ two lifestages, a motile stage and a sessile reproductive stage. Biphasic lifecycles in PVC bacteria may have more ancient origins, or conversely evolved independently.”

**R3.6:** Recommendation: accept for publication. Suggested changes need not to be made if authors disagree. Many congrats for this great work! Christian Jogler

We agree with all suggested changes and have thus included them in the revised version of the manuscript. Thank you for the congratulations!

*******************************

# Reviewer #4 (in blue)

**R4.1:** The overarching story of the manuscript revolves around symbiosis, but there was not much discussion on Chlamydiae and their hosts in nature. For example, what do the symbionts typically do for their hosts? I assume they are not all pathogenic as there is mention of Chlamydiae pathogens and symbionts, so are there any benefits that the symbionts confer? Or is “symbionts” being used here as microbes that have a long-term association with hosts, regardless of whether they are harmful or beneficial—it was not clear in the manuscript.

We thank Reviewer #4 for pointing out that we should specifically define symbiosis so that our usage of the term is clear to all readers. Here we used “symbiosis'' in its definition to refer to sustained associations between different organisms regardless of the interaction along the mutualism-parasitism spectrum (De Bary, 1879). As interaction outcomes along this spectrum can be situation-specific and shift, it is difficult generally to define a symbiotic interaction as mutualistic or parasitic. We have added this definition to the introduction as follows:

[lines 21-22] - “Symbioses span the mutualism-parasitism spectrum and include all sustained interactions between different organisms.”

Generally, we would like to thank Reviewer #4 for their questions about Chlamydiae-host interactions, as we now see that this information was lacking and could be useful for readers to interpret our results in a wider context. We have therefore also added/adjusted the following sentences in the introduction:

[lines 40-46] - “Chlamydiae are ubiquitous in environmental samples and have been identified as endosymbionts of a wide range of both microbial and multicellular hosts from across the eukaryotic tree of life. However, apart from their roles as pathogens (e.g. Chlamydiaceae), chlamydial host-effects are understudied. Yet, chlamydial host interactions are not limited to pathogenicity and span the mutualism-parasitism spectrum. For example, chlamydiae infecting protists (e.g., Parachlamydiaceae) can be host mutualists and protect against co-infection with Legionella.”

**R4.2:** Moreover, the introduction is lacking any grounding in symbiosis literature in general. The first two sentences of the introduction and the last sentence in the manuscript (discussion) make sweeping statements without any regard for the general symbiosis literature or other systems. This risks the findings being specialist to the system and not of general interest. Really throughout the manuscript, there should be more reference to symbiosis literature.

We would like to thank reviewer #4 for their comments, which have helped us to improve the level of interest our findings will have to a wider audience.

We have added references throughout the manuscript to improve the grounding of the paper in the general symbiosis literature:

- Drew et al. 2021 - Microbial evolution and transitions along the parasite-mutualist continuum
- De Bary 1879 - Die Erscheinung der Symbiose
- McFall-Ngai et al. 2013 - Animals in a bacterial world, a new imperative for the life sciences
- Husnik, F. et al. 2021 - Bacterial and archaeal symbioses with protists
- López-García et al. 2017 - Symbiosis in eukaryotic evolution
- Sogin et al. 2020. - Chemosynthetic symbioses
- Wilkins et al. 2019 - Host-associated microbiomes drive structure and function of marine ecosystems
- McLean et al. 2016 - Insect symbionts in food webs
- Moran, 1996 - Accelerated evolution and Muller’s rachet in endosymbiotic bacteria
- Moran, 2002 - Microbial Minimalism: Genome Reduction in Bacterial Pathogens
- Wernegreen, 2005 - For better or worse: genomic consequences of intracellular mutualism and parasitism
- Toft and Andersson, 2010 - Evolutionary microbial genomics: insights into bacterial host adaptation
- McCutcheon, Boyd, Dale 2019 - The Life of an Insect Endosymbiont from the Cradle to the Grave
- Russell 2020 - Horizontal transmission and recombination maintain forever young bacterial symbiont genomes.
- Pinto-Carbó 2016 - Evidence of horizontal gene transfer between obligate leaf nodule symbionts.
- Bordenstein 2004 - Bacteriophage flux in endosymbionts (Wolbachia): infection frequency, lateral transfer, and recombination rates.
- Nakabachi 2013 - Horizontal Gene Acquisition of Liberibacter Plant Pathogens from a Bacteriome-Confined Endosymbiont of Their Psyllid Vector.
- Scholz 2020 - Large scale genome reconstructions illuminate Wolbachia evolution
- Perreau and Moran, 2022 - Genetic innovations in animal–microbe symbioses
- Lagkouvardos, Shen, Horn 2014 - Improved axenization method reveals complexity of symbiotic associations between bacteria and acanthamoebae

The text was adjusted with these references as follows:

# Extended:

[lines 22-23] - “Host-symbiont associations between eukaryotes and bacteria are ubiquitous, having evolved numerous times with both multicellular and microbial hosts (McFall-Ngai et al., 2013; Husnik, F. et al., 2021).”

[lines 23-25] - “These interactions and dependencies between eukaryotic hosts and their bacterial symbionts play essential roles, from ecosystem functioning to the evolution of biological complexity (McLean et al., 2016, López-García et al., Wilkins et al., 2019, 2017, Sogin et al., 2020).”

[lines 491-492] - “HGT is known to occur in endosymbionts (Pinto-Carbó, 2016; Bordenstein, 2004; Nakabachi, 2013, Scholz, 2020). While more prevalent during horizontal host transmission (Russell, 2020), HGT is also important for predominately vertically inherited endosymbionts (*e.g.*, Wolbachia) (Scholz, 2020; Bordenstein, 2004). However, it is expected that obligate endosymbionts, such as chlamydiae, would be limited in gene exchange in comparison to free- living bacteria (Moran, 2002). ”

# Added:

[lines 21-22] - “Symbioses span the mutualism-parasitism spectrum (Drew et al., 2021) and include all sustained interactions between different organisms (Bary and de Bary, 1879).”

[lines 25-27] - “Obligate endosymbionts tend to undergo genome reduction and metabolic streamlining, driven by small population sizes, lack of recombination, and host-dependence (Moran, 1996; Moran, 2002; Wernegreen, 2005; Toft and Andersson, 2010; McCutcheon, Boyd,

and Dale, 2019).”

[lines 458-460] - “Genome evolution in obligate endosymbionts is typically thought to occur through reductive processes (Wernegreen, 2005) and is what we generally observe in ancestors of the Chlamydiaceae, which are animal pathogens (Figure 5)”

[lines 469-471] - “Furthermore, in marine bacterial-bivalve endosymbionts it has been demonstrated that horizontal symbiont transmission together with recombination can maintain intermediate genome sizes (Russell et al., 2020).”

[lines 460-462] - “Here, small intracellular population sizes and genetic isolation over time lead to gene loss through genetic drift, the accumulation of slightly deleterious mutations, and a lack of recombination (Moran, 1996; Moran, 2002; Wernegreen, 2005; Toft and Andersson, 2010)”

[lines 509-510] - “Thus, the rates of gene exchange in some chlamydial groups do not differ from free-living PVC bacteria.”

**R4.3:** It is a neat finding that Chlamydiae underwent genome reduction and expansion. The intracellular symbiont literature has many examples of metabolic complementarity between host and bacteria as a result of genome reduction, and these are usually found in invertebrates, mainly insects. Are all of the vertebrate-associated Chlamydiae pathogenic (Figure 1)? If not, what might this mean for the generality of symbiont genome reduction across host types? Is there evidence of metabolic complementarity (this relates back to the previous question on the role of the symbiont)?

The vertebrate-associated Chlamydiaceae are indeed known as pathogens. *Waddlia chrondophila*, a member of the Amoebachlamydiales order, was also initially isolated from an aborted bovine fetus, and there is some literature associating it as an emerging animal pathogen (Taylor-Brown et al., 2015). However, it also infects amoeba (Collingro et al., 2020). The remaining vertebrate-associated chlamydiae are also known pathogens (e.g., *Clavichlamydia* sp.), specifically of fish (Taylor-Brown et al., 2015). In terms of chlamydiae infecting invertebrates, such as insects and marine worms, little is known about the outcomes of interactions between chlamydiae and these hosts. However, we can see from our results that chlamydial lineages infecting animals tend towards gene loss and genome reduction, and have fewer HGT events. This is particularly clear for Chlamydiaceae.

We have further added the following sentences and references outlining this finding and indicating what it might mean for the generality of symbiont genome reduction across host types:

[lines 471-486] - “However, genome expansion had not previously been demonstrated in obligate endosymbionts. Unexpectedly, our results demonstrate that extensive gene gain in Amoebachlamydiales lead to the larger proteome sizes and increased metabolic complexity in extant members of this group (Figure 5). This finding suggests that obligate endosymbionts can evolve counter to genome reduction processes throughout evolutionary time. We see the opposite pattern in animal-infecting chlamydiae, such as the Chlamydiaceae, which have undergone genome reduction. Across other chlamydial clades, gene content has been largely maintained (Figure 5). Thus, despite a conserved endosymbiotic lifestyle, divergent patterns in genome evolution are found across the phylum Chlamydiae. Genome reduction and maintenance are in- line with prior work on the effects of transmission mode and host variability on endosymbiont genome evolution (Husnik and Keeling, 2019; Perreau and Moran, 2022). Genome reduction is associated with vertical transmission and a strict host range, while genome maintenance is found in horizontally transmitted endosymbionts that infect a wider range of hosts and encounter co- infecting endosymbionts. However, Amoebachlamydiales genome evolution challenges overarching paradigms. Our study provides evidence that genomic and metabolic complexity in endosymbionts can even increase over evolutionary time scales on the order of a billion years.”

Unfortunately, little is also known about metabolic complementarity between chlamydiae and their hosts. Likely because host specificity, and hosts in nature, are often unclear. Metabolic host complementarity could be the case in some animal-infecting chlamydiae, but this needs to be investigated further and is outside the scope of the current study.

**R4.4:** Was the last common ancestor already host-associated? Or free-living with the potential to be host-associated? If the latter, it might suggest that some bacteria are more prone to become host-associated (if they already have genes that facilitate an endosymbiotic lifestyle as a free- living microbe), which has implications for the evolution of symbiosis in general.

Yes, given the inferred gene content of the last common ancestor of Chlamydiae and current knowledge of chlamydial biology and cultured representatives, we make the presumption that it already was an obligate endosymbiont. We would like to thank the reviewer for bringing this up as it could be stated more clearly/explicitly. We have thus added the following sentence:

[lines 238-241] - “Thus far, all cultured representatives of chlamydiae are obligate endosymbionts. Similar to extant chlamydiae, since we reconstructed endosymbiosis-related genes and a tendency towards auxotrophies in LCCA we thus propose that it was most likely an obligate endosymbiont.”

# Specific comments:

**R4.5:** Line 19. Cite Drew et al. 2021. Nat Rev Microbiol. We have now cited this reference here:

[lines 21-22] - “Symbioses span the mutualism-parasitism spectrum (Drew et al., 2021) and include all sustained interactions between different organisms (De Bary, 1879).”

**R4.6:** Line 180. How likely is extinction?

The exact likelihood of gene extinction is difficult to directly quantify and to state more generally. However, cross-lineage extinction probabilities are modeled by the ancestral reconstruction tool ALE. We used a modified version of ALE (https://github.com/maxemil/ALE-pipeline) where these extinction probabilities were used to correct ancestral proteome sizes accordingly, as has previously been done (Williams et al., 2017 PNAS, PMID: 28533395).

We have clarified this point in both the main text and methods as follows:

[lines 428-431] - “The proteome size of Amoebachlamydiales ancestors increased relative to other chlamydiae. Proteome size (i.e., the sum of inferred gene copies corrected for by gene extinction probability; see Methods; Data S7) inferred across chlamydial ancestors using gene-tree species- tree reconciliation, and protein-coding gene copies present in extant chlamydial genomes.”

[lines 716-718] - “In addition, the specific implementation of ALE corrects ancestor gene copy number estimates using modeled gene extinction probability rates (Szöllősi et al., 2013), as has previously been employed to estimate ancestral proteome sizes (Williams et al., 2017).”

**R4.7:** Figure 5. More thorough description of results needed. This figure is complication but very illuminating.

We have adjusted this figure so it is less complicated and hopefully easier to interpret. We have done so by adding a separate panel for within family proteome sizes (see 5b), and removed these nodes from the figure comparing ancestor proteome size to relative evolutionary divergence (see 5a). Here, the inferred ancestral and extent proteome sizes are directly compared for each family.

We have adjusted the Figure 5 legend as follows:

[lines 428-437] - “Figure 5. The proteome size of Amoebachlamydiales ancestors increased relative to other chlamydiae. Proteome size (i.e., the sum of inferred gene copies corrected for by gene extinction probability; see Methods; Data S7) inferred across chlamydial ancestors using gene-tree species-tree reconciliation, and protein-coding gene copies present in extant chlamydial genomes. a. Inferred proteome size of early chlamydial ancestors scaled to the relative evolutionary divergence (RED) metric87. The RED metric provides an approximation of relative time from a given common ancestor (LPVCCA, RED=0) to extant taxa (RED=1). b. Comparison of proteome size between ancestors and extant members from each chlamydial family. Chlamydiae from families without in-family ancestors are grouped together in the grey boxplot (i.e., Criblamydiaceae, Waddliaceae, Chlamydiae Clade IV, and unclassified). Nodes and branches are coloured by family assignment according to the legend. Abbreviations of key ancestors are labeled as in Figure 3 (see Data S7). ”

We have also expanded the main text to further describe these results:

[lines 403-424] - “The gain of aerobiosis-related genes in the Amoebachlamydiales was accompanied by other prominent gene gains related to metabolic potential (Extended Data Figure 3), in line with the extended metabolic capabilities and larger gene repertoires of extant members relative to other chlamydiae. Unexpectedly, our results provide evidence that many of these key genes were not present in the LCCA but were instead gained through HGT during Amoebachlamydiales evolution (Extended Data Figure 4) leading to their characteristic metabolic complexity. Although we cannot accurately reconstruct the evolution of all genes, such as those that have since gone extinct or are fast-evolving, by examining relative reconstructed proteome sizes (ie., the number of protein-coding genes) we can investigate larger patterns. Using multiple methods, our reconstructions of gene content indicate a shift towards larger proteome sizes leading to the LCWPA (Figures 5a and S12a). By using gene-tree aware ancestral reconstruction, which are able to estimate and take into account gene extinction rates, we can more accurately capture ancient events. The Amoebachlamydiales ancestor was reconstructed with 1,691 protein- coding genes, relative to 1,225 and 1,408 genes reconstructed in LG1CA and LG2CA, respectively (Figure 3). Further large gene influxes through HGT led to increased metabolic complexity and gene content expansion in Amoebachlamydiales (Figures 5a and Extended Data Figure 4). The last common ancestor of the Criblamydiaceae, Waddliaceae, and Parachlamydiaceae (LCWPCA) was reconstructed with a proteome size of 2,560 genes as having doubled gene content since LG2CA (Figure 3). Within each chlamydial family, ancestors were reconstructed as having proteome sizes that resemble extant members (Figure 5b). However, in a reconstruction using only gene presence and absence, ancestor proteome sizes were underestimated relative to extant members within each chlamydial family (Figure S12b).”

**R4.8:** Line 334. References needed on that last sentence. What theoretical papers discuss genome reduction or is there evidence from other systems of genome reduction?

We have reworded this sentence and added a separate one (see R4.3) outlining the theory and key papers for evidence of genome reduction in obligate endosymbionts:

[lines 481-484] - **“**Genome reduction is associated with vertical transmission and a strict host range, while genome maintenance is found in horizontally transmitted endosymbionts that infect a wider range of hosts and encounter co-infecting endosymbionts. However, Amoebachlamydiales genome evolution challenges overarching paradigms.**”**

**R4.9:** Line 342. Horizontal transmission and HGT are increasingly being recongised as key to the maintenance of other inherited endosymbionts (e.g., Wolbachia, Arsenophonus). Can the authors discuss their findings in light of findings in other symbioses?

We have extended the sentences around line 342 (as mentioned above) to discuss our findings in light of other symbioses:

[lines 491-495] - “HGT is known to occur in endosymbionts (Pinto-Carbó, 2016; Bordenstein, 2004; Nakabachi, 2013, Scholz, 2020). While more prevalent during horizontal transmission (Russell, 2020), HGT is also important for predominately vertically inherited endosymbionts (e.g., Wolbachia) (Scholz, 2020; Bordenstein, 2004). However, it is expected that obligate endosymbionts, such as chlamydiae, would be limited in gene exchange in comparison to free- living bacteria (Moran, 2002).”

**R4.10:** Line 346. How would coinfection occur? Is it common? Do these organisms occupy the same host niche?

For example, co-infecting symbionts are very commonly found in microbial eukaryotes. We had stated this earlier in the text in the section about proteome size increase (lines 465-467). However, we have now also reiterated this after the line in question.

[lines 500-502] - “Such HGT events could have been facilitated by symbiont co-occurrence or the presence of prey bacteria in phagocytosing hosts, both of which are common among microbial eukaryotes (Moliner et al., 2010; Wang & Wu, 2017; Bertelli & Greub, 2012; Lagkouvardos, Shi, and Horn, 2014).”

**R4.11:** Line 374. References needed.

We have adjusted this sentence and added references:

[lines 525-528] - “While HGT is well recognized in endosymbiotic bacteria (Pinto-Carbó, 2016; Bordenstein, 2004; Nakabachi, 2013, Scholz, 2020, Russell, 2020, Bordenstein, 2004), our study provides a systematic view on the pervasiveness of HGT across a phylum of endosymbionts. In particular, providing further evidence for a role of inter-symbiont HGT in long-term endosymbiont genome evolution.”

*******************************

**Final Decision Letter:**

| **Message:** | Our ref: NMICROBIOL-21112817A  24th August 2022  Dear Thijs,  Thank you for submitting your revised manuscript "Gene gain facilitated the origin and diversification of an ancient phylum of symbionts" (NMICROBIOL-21112817A). It has now been seen by the original referees and their comments are below. The reviewers find that the paper has improved in revision, and therefore we'll be happy in principle to publish it in Nature Microbiology, pending minor revisions to satisfy the referees' final requests and to comply with our editorial and formatting guidelines.  If the current version of your manuscript is in a PDF format, please email us a copy of the file in an editable format (Microsoft Word or LaTex)-- we can not proceed with PDFs at this stage.  We are now performing detailed checks on your paper and will send you a checklist detailing our editorial and formatting requirements in about a week. Please do not upload the final materials and make any revisions until you receive this additional information from us.  Thank you again for your interest in Nature Microbiology Please do not hesitate to contact me if you have any questions.  Sincerely,  [redacted]   Reviewer #2 (Remarks to the Author):  I've read carefully the answer of the authors to my major criticisms. I thank that for tackling them in a detailed way. Their analysis suggests that the bias I mentioned does not apply to the used methodology. I'm a bit surprised by that, since in theory, it should still exist. I suspect the effect is smaller than I feared. In which case, the authors have a good argument. We'll see the reception of the community. I have no further comments to this interesting study.   Reviewer #3 (Remarks to the Author):  All my (minor) concerns are addressed. Many congrats again, great work! Love to see it published, soon.   Reviewer #4 (Remarks to the Author):  This revision is a major improvement. All comments were addressed satisfactorily. Looking forward to seeing this amazing work published. |
| --- | --- |
